# Expedient synthesis of poly-aryl substituted allenylsilanes *via* silylation of 1,3-diaryl propargyl carbonates

**DOI:** 10.1039/d6ra04344e

**Published:** 2026-07-04

**Authors:** Shuxian Zhu, Weijie Luo, Yuan Zhang, Qingqing Xuan, Jinglong Chen

**Affiliations:** a College of Material Sciences and Engineering, Huaqiao University Xiamen Fujian 361021 China 314029268@qq.com; b College of Materials Science and Engineering, Fuzhou University Fuzhou Fujian 350108 China

## Abstract

Allenylsilanes serve as important and versatile intermediates in organic synthesis, finding widespread applications in medicinal chemistry and materials science. A mild and regioselective silylation of 1,3-diaryl propargyl carbonates using palladium and copper as co-catalysts is reported herein. Under this method various poly-aryl substituted allenylsilanes were obtained in mono-regioselectivities and excellent yields. Compared with the existing single-metal catalytic systems, this Pd/Cu cooperative activation mode exhibits superior substrate adaptability and reaction efficiency. This work not only enriches the synthetic methodology for allenylsilanes but also provides a new avenue for the synthesis of poly-aryl substituted allene derivatives.

## Abstract 2

A Pd/Cu bimetallic cooperative catalytic system enables highly regioselective γ-silylation of 1,3-diaryl propargyl carbonates, delivering poly-aryl substituted allenylsilanes in excellent yields with broad functional group tolerance.

## Graphical abstract

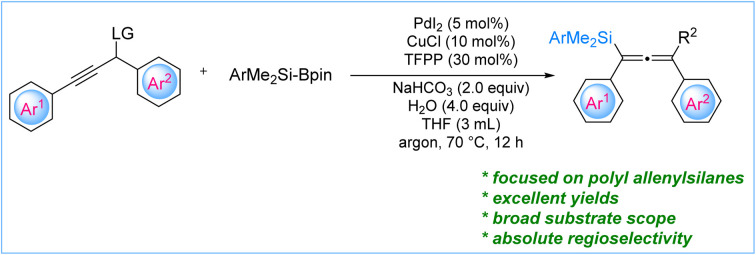

## Introduction

**Fig. 1:**
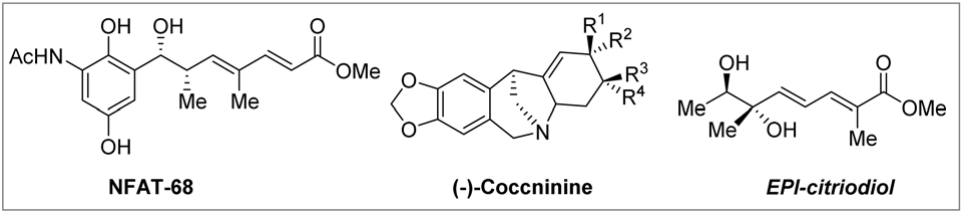
Natural products synthesized from allenylsilanes.

Allenylsilanes represent a class of versatile and valuable building blocks in organic synthesis, owing to their unique reactivity, stability and low toxicity.^1^ They have been extensively employed as key intermediates in the construction of complex natural products, pharmaceuticals, and functional materials (Fig. 1).^2^ Thus, the development of efficient and regio-selective methods for the synthesis of allenylsilanes with different structures has attracted sustainable interest from chemists.

**Scheme 1:**
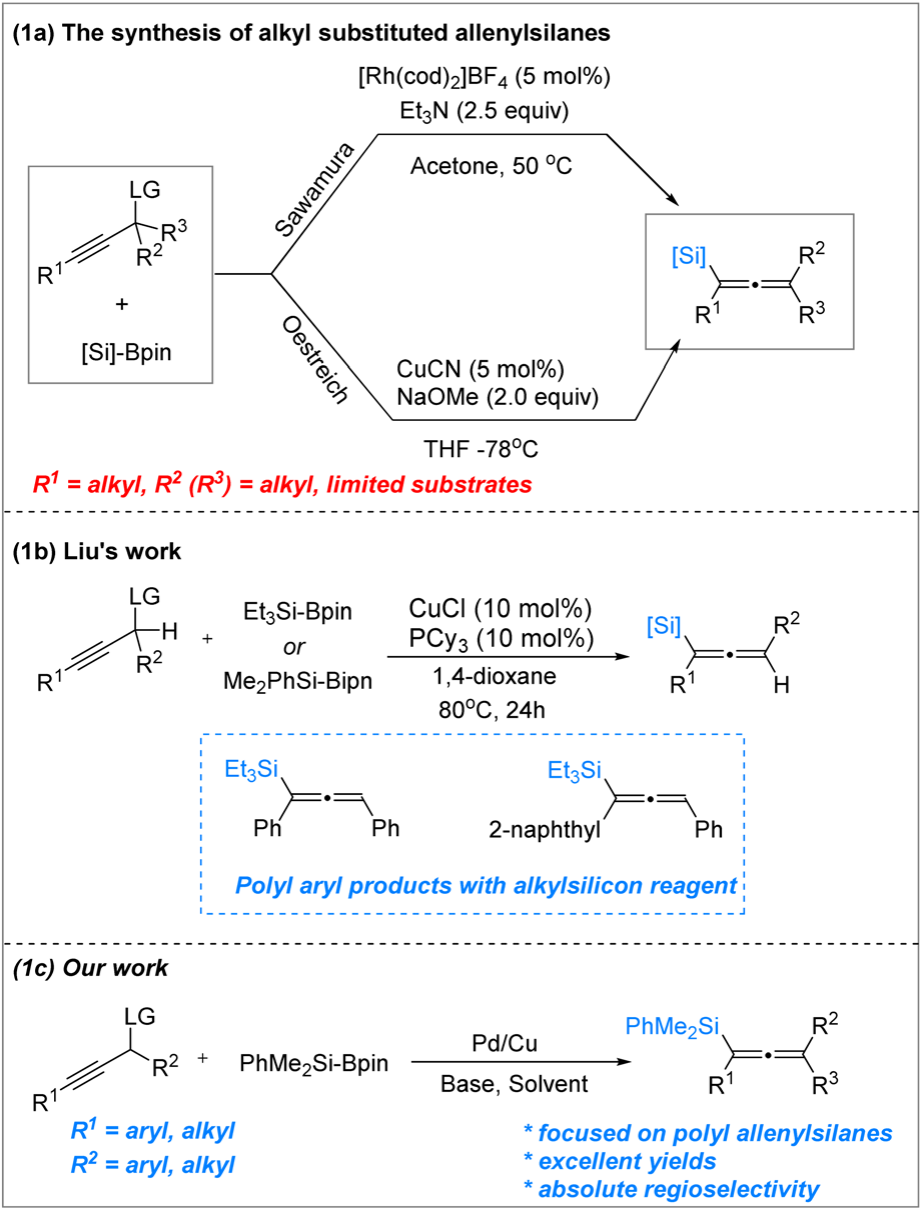
Transition-metal-catalyzed synthesis of allenylsilanes.

Traditional approaches for the synthesis of allenylsilanes typically involve the stoichiometric use of silyl-metal reagents with propargylic derivatives.^3^ However, these methods often suffer from limitations such as poor functional group tolerance, narrow substrate scope, and unsatisfactory regioselectivity. Recently, transition-metal-catalyzed silylation of propargylic derivatives with silyl element compounds (*e.g.* silylboronates) has emerged as a powerful and reliable strategy for the construction of allenylsilanes.^4^ In this context, Sawamura, Oestreich, and Liu *et al.* reported pioneering work on coupling of propargylic carbonates with silylboronates, offering a general and expedient approach to allenylsilanes (Scheme 1). In 2009, Sawamura and coworkers,^5^ reported the synthesis of allenylsilanes *via* silylation of propargylic carbonates under the catalyst of Rh-complex (Scheme 1a). In this creative work, many alkyl-substituted allenylsilanes were obtained, however, aryl-substituted propargylic carbonates failed to give the desired products. Later on, Oestreich and coworkers^6^ also reported similar work on the synthesis of allenylsilanes (Scheme 1a). In their work, although cheaper copper salts were employed as catalysts, the substrates scope was also limited. Recently, Liu and coworkers^7^ further studied the synthesis of allenylsilanes from propargylic derivatives (Scheme 1b). In their work, aryl-substituted allenylsilanes were got. Although, the substrates' scope was enlarged, substituents on silicon center were limited to alkyl group. Except for silylboranes, R1R2R3Si-H,^8^ R1R2R3Si-NTf2,^9^ R1R2R3Si-OTf^10^ regents were also employed to synthesis allenylsilanes. More importantly, several asymmetric synthesis methods were also developed.^11^ Fluorine-containing compounds and (trimethylsilyl)alkynes are also an important class of synthons,^12,4*e*^ and they can also be used to synthesize allenylsilanes. In 2015, Knochel and coworkers, employed benzylic (trimethylsilyl)alkynes as substrates to get the 1,3-diarylallenylsilanes. Despite these significant advances, the substrate scope in many existing protocols remains primarily focused on alkyl-substituted or terminal propargylic systems. The systematic investigation of 1,3-diaryl substituted propargylic carbonates, which would lead to poly-aryl substituted allenylsilanes with potential applications in materials science and pharmaceutical chemistry, has been largely unexplored. Inspired by these considerations and as part of our ongoing interest in the development of novel methods for organosilicon compounds, we envisioned that a palladium/copper bimetallic cooperative catalytic system might offer a unique solution for the silylation of 1,3-diaryl propargylic carbonates. We hypothesized that palladium could facilitate the activation of the propargylic carbonate, while copper could efficiently activate the silylboronate, enabling a highly regioselective γ-silylation under mild conditions. Herein, we report the successful implementation of this strategy, providing a general and efficient access to a diverse array of poly-aryl substituted allenylsilanes in excellent yields with exclusive regioselectivity (Scheme 1c). This work not only expands the synthetic toolbox for allenylsilanes but also highlights the potential of Pd/Cu cooperative catalysis in addressing challenging substrate classes.

## Results and discussion

To develop an efficient synthetic method for poly-aryl substituted allenylsilanes, the silylation of propargylic carbonate 1a with silylboronate 2a was conducted in THF under cocatalysts of PdCl_2_ and CuCl in the presence of KHCO_3_, and pleasingly, the desired product 3a was got in 51% yield with exclusive regioselectivity (Table 1, entry 1). Next, the investigations were focused on evaluating the necessity of a cooperative catalytic system. When PdCl_2_ or CuCl was employed alone as the catalyst, low yield of 3a was observed (Table 1, entry 2–3), indicating that a single-metal system was insufficient for this transformation. Subsequently, the effect of ligand was also studied (Table 1, entry 4–7). According to the results, 3a was failed to generate in the absence of a phosphine ligand, and among the various ligands examined, tris(4-fluorophenyl)phosphine (TFPP) was proved to be the most effective affording 3a in 80% yield. Then, various palladium catalysts were also tested, and PdI_2_ was proved to be the most effective and give 3a in 85% yield. We also screened the effects of base and when NaHCO_3_ was used instead of KHCO_3_, the yield of 3a could be increased to 94% (Table 1, entry 11). Finally, effect of temperature was also examined, it showed that higher yield of 3a was got when the reaction was carried out at 70 °C. Thus the optimal reaction condition was obtained as: Pd(OAc)_2_ (5 mol%), CuCl (10 mol%), TFPP (30 mol%), NaHCO_3_ (2.0 equiv.), H_2_O (4.0 equiv.) in THF at 60 °C stirred for 12 h under argon.

**Table 1:** Optimization reactions^a^

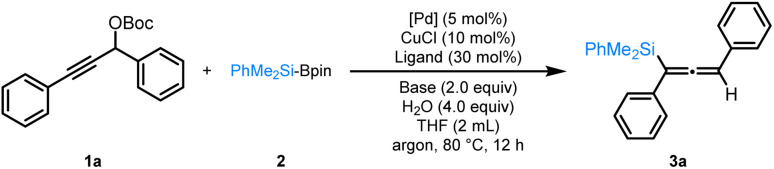					
Entry	[Pd]	[Cu]	Ligand	Base	Yield^b^ (%)
1	PdCl_2_	CuCl	PPh_3_	KHCO_3_	51
2	PdCl_2_	—	PPh_3_	KHCO_3_	6
3	—	CuCl	PPh_3_	KHCO_3_	Trace
4	PdCl_2_	CuCl	—	KHCO_3_	0
5	PdCl_2_	CuCl	P(*p*-tol)_3_	KHCO_3_	43
6	PdCl_2_	CuCl	TDMPP	KHCO_3_	0
7	PdCl_2_	CuCl	TFPP	KHCO_3_	80
8	Pdl_2_	CuCl	TFPP	KHCO_3_	85
9	[Pd(allyl)Cl)]_2_	CuCl	TFPP	KHCO_3_	10
10	Pd(PCy_3_)_2_Cl_2_	CuCl	TFPP	KHCO_3_	58
11	Pdl_2_	CuCl	TFPP	NaHCO_3_	94
12^c^	Pdl_2_	CuCl	TFPP	NaHCO_3_	98

a Unless otherwise noted, the reaction was run under the following reaction conditions: 1a (0.2 mmol), 2 (0.4 mmol), [Pd] (5 mol%), CuCl (10 mol%), ligand (30 mol%), base (2.0 equiv.) and H_2_O (4.0 equiv.) in THF (2 mL) at 80 °C for 12 h under an argon atmosphere.

b Isolated yield.

c At 70 °C.

**Scheme 2:**
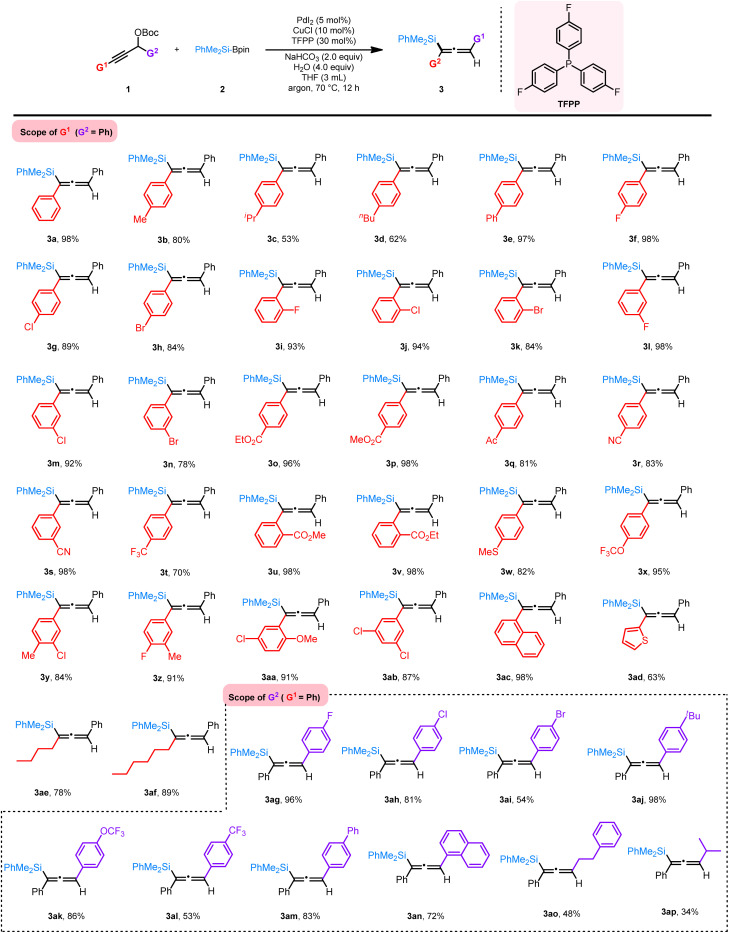
Substrate scope studies.

With the optimized reaction conditions in hand, the substrate scope of this Pd/Cu-catalyzed silylation was systematically investigated. The results were summarized in Scheme 2, which demonstrate the broad applicability and high regioselectivity of this cooperative catalytic system. Firstly, the scope of G1 was examined. The diverse substituents on the aryl rings attached to the sp-hybridized carbon were studied. Generally, substrates with alkyl groups (such as, –Me, –^*i*^Pr, –^*n*^Bu), phenyl group at the para position of the aryl rings underwent the reactions smoothly and gave the corresponding poly-aryl allenylsilanes in excellent yields (3b–3e). Then, substrates with halogens (such as –F, –Cl, –Br) at *para*, *ortho*, and *meta* position of the aryl rings were also examined, and all of them could afford the desired products in good to excellent yields (3f–3n). Following by, electron-withdrawing groups, such as esters, acetyl, –CN and –CF_3_ group at *para*, *ortho*, and *meta* position were also studied, and all of them could afford the desired products in excellent yields (3o–3v). Next, electron-donating groups (*e.g.*, –SMe, –OCF_3_) were also tested, and the corresponding products were also got in good yields (3w–3x). The results elucidated that electronic effects have a negligible impact on our reactions. Then, disubstituted substrates were also examined, the products were also got in excellent yields with absolute regioselectivity (3y–3ab). The results indicated that steric hindrance had little effect to our reactions. Then, 1-naphthyl and 2-thienyl substituted substrates could also go through the reactions smoothly and give the corresponding products with absolute regioselectivity (3ac–3ad). These examples highlight the potential of this method for the synthesis of heteroaryl-substituted allenes, which are of interest in medicinal chemistry and materials science. Alkyl substituted substrates could also give the corresponding allenylsilanes with exclusive γ-regioselectivity (3ae–3af). At last, the scope of G2 was also tested, various substituted phenyl groups, 1-naphthyl and alkyl groups were all tolerated and afforded the corresponding products with exclusive regioselectivity (3ag–3ap). Different with the reported references, alkyl groups derived substrates afforded relatively poor yields (3ao–3ap). In summary, the developed Pd/Cu bimetallic catalytic system exhibits exceptional substrate generality, accommodating a wide range of electronic and steric variations on both G1and G2 part, and various poly-aryl substituted allenylsilanes were synthesized successfully.

## Mechanism

**Scheme 3:**
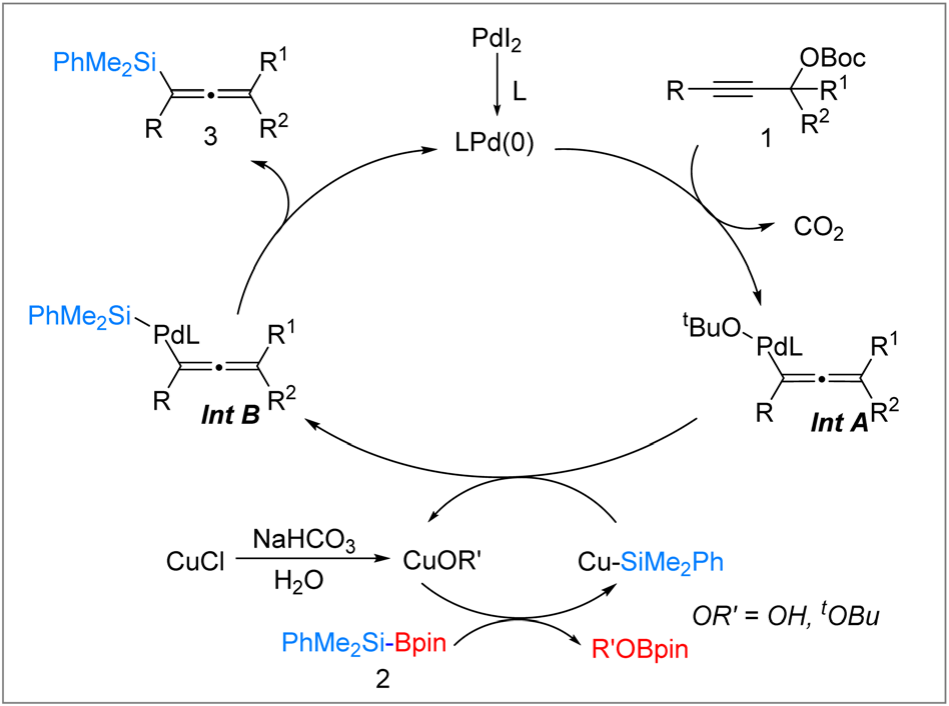
Proposed mechanism.

Based on the experimental observations and precedent literature, a plausible mechanism for the Pd/Cu-catalyzed γ-selective silylation of 1,3-diaryl propargyl carbonates is proposed as depicted in Scheme 3. The catalytic cycle is proposed to involve activation of the propargylic carbonate by palladium. Initially, the propargylic carbonate (1) undergoes oxidative addition to Pd(0) species, generated *in situ* from Pd(OAc)_2_ in the presence of TFPP, to form a Pd(ii)-allenyl Int A. This step is facilitated by the phosphine ligand (TFPP), which can well stabilizes the Pd(0) complex. Concurrently, the nucleophilic Cu-SiMe_2_Ph species is generated *via* transmetalation process between silylboronate and the copper(i) specie. This process is promoted by NaHCO_3_ and H_2_O. This step is crucial, as the copper catalyst alone cannot effectively activate the propargylic carbonate (Table 1, entry 3), and palladium alone cannot efficiently transfer the silyl group (entry 2), highlighting the necessity of the bimetallic system. Then, the other transmetalation process occurs between Cu-SiMe_2_Ph and Pd(ii)-allenyl Int A, affording Int B, which give the desired product 3*via* reductive elimination, releasing the Pd(0) and Cu(i) species to re-enter the catalytic cycle.

## Conclusions

In summary, we have developed a mild and highly regioselective Pd/Cu-catalyzed silylation of 1,3-diaryl propargylic carbonates for the efficient synthesis of poly-aryl substituted allenylsilanes. The cooperative bimetallic system is essential, as single-metal catalysts give much lower yields. The method features exceptional substrate generality, tolerating a wide range of electronic and steric variations, including halogens, electron-withdrawing/donating groups, disubstituted arenes, heteroaromatics, and even alkyl substituents, all with exclusive γ-regioselectivity and excellent yields. This work provides a practical and general route to structurally diverse allenylsilanes and demonstrates the power of Pd/Cu cooperative catalysis in addressing challenging substrate classes.

## Author contributions

Shuxian Zhu: methodology, investigation, formal analysis, writing – original draft, and writing – review & editing. Weijie Luo: conceptualization, conceptualization. Yuan Zhang: investigation, formal analysis, methodology. Jinglong Chen and Qingqing Xuan: project administration, funding acquisition, writing – review & editing.

## Conflicts of interest

There are no conflicts to declare.

## Supplementary Material

RA-OLF-D6RA04344E-s001

## Data Availability

The data supporting this communication have been included in the supplementary information (SI). Data for this paper, including [^1^H-NMR, ^13^C-NMR, HRMS] are available at spectroscopy. Supplementary information: the synthetic methods for the relevant substrates, the optimization process for the reaction conditions, and related data of ^1^H-NMR, ^13^C-NMR, HRMS. See DOI: https://doi.org/10.1039/d6ra04344e.
