# Supplementary material for: Expedient synthesis of poly-aryl substituted allenylsilanes via silylation of 1,3-diaryl propargyl carbonates
Source: RSC Adv. 2026 Jul 4;16(35):36764–8. doi: 10.1039/d6ra04344e (PMC13331679; doi:10.1039/d6ra04344e)
Supplement: RA-016-D6RA04344E-s001 [file RA-016-D6RA04344E-s001.pdf]

Supplementary Information (SI) for RSC Advances.
This journal is © The Royal Society of Chemistry 2026

Supporting Information

for

Expedient synthesis of poly-aryl substituted allenylsilanes via silylation of 1,3-diaryl propargyl carbonates

Shuxian Zhu,[a] Weijie Luo,[a] Yuan Zhang,[a] Qingqing Xuan*[a] and Jinglong Chen*[b]

Table of Contents

1. General Information 3

2. Procedures for the synthesis of substrates 4

2.1 General procedure A for the synthesis of 1a-1ad[1] 4

2.2 General procedure B for the synthesis of 1ae-1ap[2] 4

2.3 Procedure for the synthesis of 1,3-diphenylprop-2-yn-1-yl acetate 5

2.4 Procedure for the synthesis of 1,3-diphenylprop-2-yn-1-yl benzoate 5

2.5 Procedure for the synthesis of 1,3-diphenylprop-2-yn-1-yl pivalate 6

2.6 Procedure for the synthesis of Et_3Si-Bpin 7

3. Characterization of substrates 8

4. Optimization studies 29

5.1. General procedure C for the synthesis of products 3 35

5.2. Large scale experiment of synthesis of products 3a 35

6. General procedure D for the synthesis of products 4 36

7. Characterization of products 37

8. NMR spectra 59

8.1 NMR spectra of substrates 59

8.2 NMR spectra of products 114

9. References 166

1. General Information

All chemicals were purchased from Adamas Reagent, Ltd, Energy chemical company, J&K Scientific Ltd, Alfa Aesa chemical company and so forth. Anhydrous solvents are commercially available (energy) and stored in a glove box. Unless otherwise stated, all experiments were conducted in a seal tube under argon atmosphere. Reactions were monitored by TLC or GC-MS analysis. Flash column chromatography was performed over silica gel (200-300 mesh). ^{1}H-NMR and ^{13}C-NMR spectra were recorded in $CDCl_3$ on a Bruker Avance 500 spectrometer (500 MHz ^{1}H, 126 MHz ^{13}C, 471 MHz ^{19}F) at room temperature. Chemical shifts were reported in ppm on the scale relative to $CDCl_3$ (δ = 7.26 for ^{1}H-NMR, δ = 77.00 for ^{13}C-NMR) as an internal reference. High resolution mass spectra were recorded using Q-TOF time-of-flight mass spectrometer. Coupling constants (*J*) were reported in Hertz (Hz). Oil bath was used as heating source.

2. Procedures for the synthesis of substrates

2.1 General procedure A for the synthesis of 1a-1ad[1]

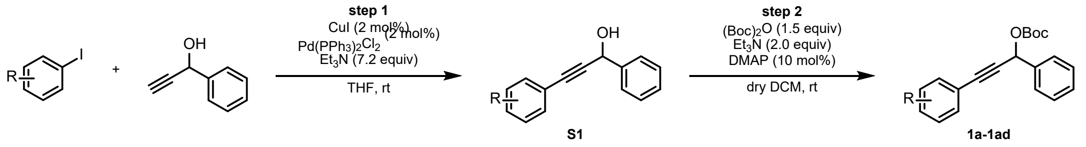

Step 1: CuI (11.4 mg, 0.06 mmol) and $Pd(PPh_3)_2Cl_2$ (42.1 mg, 0.06 mmol) were charged into a 25 mL Schlenk tube and the tube was refilled with argon. THF (3 mL) was added to the tube. Aryl iodides (3 mmol), 1-phenylprop-2-yn-1-ol (0.37 mL, 3 mmol) and triethylamine (3 mL, 21.6 mmol) were added to the mixture at room temperature. The reaction mixture was stirred at room temperature overnight. Saturated NH_4Cl solution (40 mL) was added to the mixture and the resulting aqueous phase was extracted three times with EtOAc. The combined organic phase was dried over anhydrous Na_2SO_4 and concentrated under reduced pressure. The residue was purified by silica gel column chromatography to obtain the desired aryl propargyl alcohol compounds **S1**.

Step 2: In a reaction tube, a mixture of $(Boc)_2O$ (1.5 equiv), Et_3N (2.0 equiv) and DMAP (10 mol%) in dry DCM (0.5 M) were stirred at room temperature, followed by dropwise addition of the propargyl alcohol derivative **S1** (1.0 equiv). The reaction was monitored by TLC and then quenched with aqueous NH_4Cl solution and extracted with DCM (x3). The organic phase was washed with brine after which it was dried over anhydrous Na_2SO_4, and concentrated under reduced pressure. The crude product was purified by silica gel column chromatography to give the desired tert-butyl propargyl carbonates **1a-1d**.

2.2 General procedure B for the synthesis of 1ae-1ap[2]

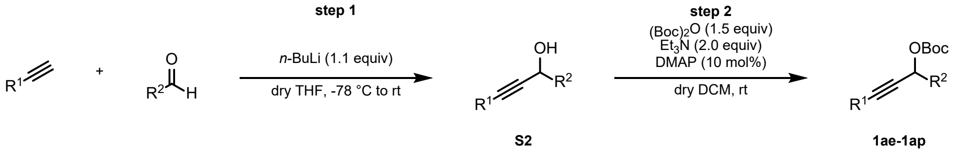

Step 1: *n*-BuLi (2.1 mL, 1.6 M in hexane, 3.3 mmol) was added to a stirred solution of alkyne compound (3.3 mmol, 1.1 equiv) in THF (4 mL) at -78 °C, and the mixture was stirred at -78 °C for 30 min. To the resulting solution was added aldehyde (3 mmol, 1.0 equiv) at -78 °C, and the mixture was gradually warmed to rt. The reaction was monitored by TLC and then quenched by the addition of water and extracted with DCM (x3). The organic phase was washed with brine after which it was dried over anhydrous Na_2SO_4, and concentrated under reduced pressure. The residue was purified

by silica gel column chromatography to obtain the desired propargyl alcohol compounds **S2**.

Step 2: The preparation of the subsequent tert-butyl propargyl carbonates **1ae-1ap** are the same as that of the **step 2** in the previous **general procedure A**.

2.3 Procedure for the synthesis of 1,3-diphenylprop-2-yn-1-yl acetate

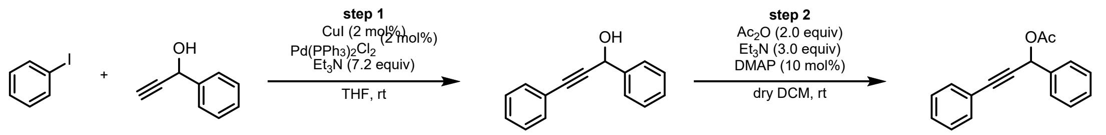

Step 1: CuI (11.4 mg, 0.06 mmol) and $Pd(PPh_3)_2Cl_2$ (42.1 mg, 0.06 mmol) were charged into a 25 mL Schlenk tube and the tube was refilled with argon. THF (3 mL) was added to the tube. Aryl iodides (3 mmol), 1-phenylprop-2-yn-1-ol (0.37 mL, 3 mmol) and triethylamine (3 mL, 21.6 mmol) were added to the mixture at room temperature. The reaction mixture was stirred at room temperature overnight. Saturated NH_4Cl solution (40 mL) was added to the mixture and the resulting aqueous phase was extracted three times with EtOAc. The combined organic phase was dried over anhydrous Na_2SO_4 and concentrated under reduced pressure. The residue was purified by silica gel column chromatography to obtain the desired 1,3-diphenylprop-2-yn-1-ol.

Step 2: In a reaction tube, a mixture of Ac_2O (2.0 equiv), Et_3N (3.0 equiv) and DMAP (10 mol%) in dry DCM (0.5 M) were stirred at room temperature, followed by dropwise addition of the 1,3-diphenylprop-2-yn-1-ol (1.0 equiv). The reaction was monitored by TLC and then quenched with aqueous NH_4Cl solution and extracted with DCM (x3). The organic phase was washed with brine after which it was dried over anhydrous Na_2SO_4, and concentrated under reduced pressure. The crude product was purified by silica gel column chromatography (PE: EA = 100:1, v/v) to give the desired acetate in 83% yield (622.8 mg, 2 steps) as a yellow oil. ^1H NMR (500 MHz, Chloroform-d): δ 7.64 – 7.60 (m, 2H), 7.51 – 7.48 (m, 2H), 7.44 – 7.38 (m, 3H), 7.35 – 7.30 (m, 3H), 6.72 (s, 1H), 2.15 (s, 3H). ^{13}C NMR (126 MHz, Chloroform-d): δ 169.9, 137.2, 132.0, 129.0, 128.9, 128.7, 128.3, 127.8, 122.1, 87.1, 85.6, 66.1, 21.2. HRMS (ESI) m/z: [M+H]+ Calcd. for $C_{17}H_{15}O_2$+ 251.1067; Found: 251.1067. ^1H and ^{13}C NMR are in agreement with previously reported literature values.[3]

2.4 Procedure for the synthesis of 1,3-diphenylprop-2-yn-1-yl benzoate

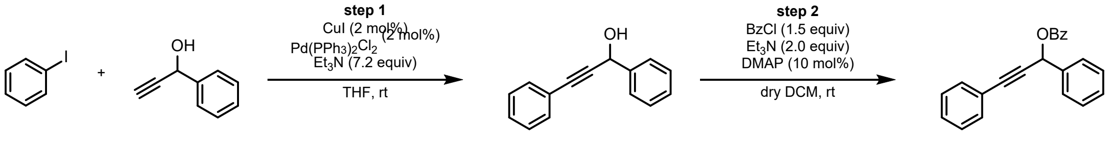

Step 1: CuI (11.4 mg, 0.06 mmol) and $Pd(PPh_3)_2Cl_2$ (42.1 mg, 0.06 mmol) were charged into a 25 mL Schlenk tube and the tube was refilled with argon. THF (3 mL) was added to the tube. Aryl iodides (3 mmol), 1-phenylprop-2-yn-1-ol (0.37 mL, 3 mmol) and triethylamine (3 mL, 21.6 mmol) were added to the mixture at room temperature. The reaction mixture was stirred at room temperature overnight. Saturated NH_4Cl solution (40 mL) was added to the mixture and the resulting aqueous phase was extracted three times with EtOAc. The combined organic phase was dried over anhydrous Na_2SO_4 and concentrated under reduced pressure. The residue was purified by silica gel column chromatography to obtain the desired 1,3-diphenylprop-2-yn-1-ol.

Step 2: In a reaction tube, a mixture of BzCl (1.5 equiv), Et_3N (2.0 equiv) and DMAP (10 mol%) in dry DCM (0.5 M) were stirred at room temperature, followed by dropwise addition of the 1,3-diphenylprop-2-yn-1-ol (1.0 equiv). The reaction was monitored by TLC and then quenched with aqueous NH_4Cl solution and extracted with DCM (x3). The organic phase was washed with brine after which it was dried over anhydrous Na_2SO_4, and concentrated under reduced pressure. The crude product was purified by silica gel column chromatography (PE: EA = 100:1, v/v) to give the desired benzoate in 80% yield (748.9 mg, 2 steps) as a yellow oil. ^1H NMR (500 MHz, Chloroform-d): δ 8.18 – 8.13 (m, 2H), 7.76 – 7.73 (m, 2H), 7.58 (t, J = 7.4 Hz, 1H), 7.54 (dt, J = 7.8, 2.6 Hz, 2H), 7.49 – 7.40 (m, 5H), 7.38 – 7.32 (m, 3H), 7.02 (s, 1H). ^{13}C NMR (126 MHz, Chloroform-d): δ 165.5, 137.3, 133.3, 132.0, 130.0, 129.9, 129.0, 128.9, 128.8, 128.5, 128.4, 127.9, 122.2, 87.4, 85.7, 66.7. HRMS (ESI) m/z: [M+H]+ Calcd. for $C_{22}H_{17}O_2^+$ 313.1223; Found: 313.1222. ^1H and ^{13}C NMR are in agreement with previously reported literature values.[4]

2.5 Procedure for the synthesis of 1,3-diphenylprop-2-yn-1-yl pivalate

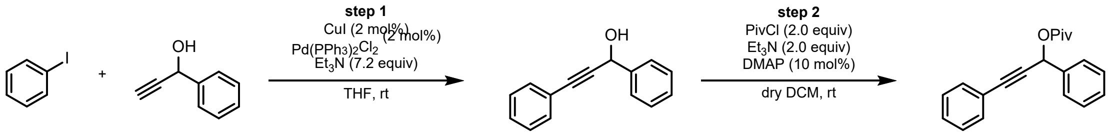

Step 1: CuI (11.4 mg, 0.06 mmol) and $Pd(PPh_3)_2Cl_2$ (42.1 mg, 0.06 mmol) were charged into a 25 mL Schlenk tube and the tube was refilled with argon. THF (3 mL) was added to the tube. Aryl iodides (3 mmol), 1-phenylprop-2-yn-1-ol (0.37 mL, 3 mmol) and triethylamine (3 mL, 21.6 mmol) were added to the mixture at room temperature. The reaction mixture was stirred at room temperature overnight. Saturated NH_4Cl solution (40 mL) was added to the mixture and the resulting aqueous phase was extracted three times with EtOAc. The combined organic phase was dried over anhydrous Na_2SO_4 and concentrated under reduced pressure. The residue was purified by silica gel column chromatography to obtain the desired 1,3-diphenylprop-2-yn-1-ol.

Step 2: In a reaction tube, a mixture of PivCl (2.0 equiv), Et_3N (2.0 equiv) and DMAP (10

mol%) in dry DCM (0.5 M) were stirred at room temperature, followed by dropwise addition of the 1,3-diphenylprop-2-yn-1-ol (1.0 equiv). The reaction was monitored by TLC and then quenched with aqueous NH_4Cl solution and extracted with DCM (x3). The organic phase was washed with brine after which it was dried over anhydrous Na_2SO_4, and concentrated under reduced pressure. The crude product was purified by silica gel column chromatography (PE: EA = 100:1, v/v) to give the desired pivalate in 72% yield (632.9 mg, 2 steps) as a yellow oil. ^{1}H NMR (500 MHz, Chloroform-d): δ 7.62 – 7.59 (m, 2H), 7.51 – 7.48 (m, 2H), 7.45 – 7.41 (m, 2H), 7.40 – 7.36 (m, 1H), 7.36 – 7.30 (m, 3H), 6.72 (s, 1H), 1.27 (s, 9H). ^{13}C NMR (126 MHz, Chloroform-d): δ 177.3, 137.6, 132.0, 128.8, 128.74, 128.67, 128.3, 127.5, 122.3, 86.8, 85.9, 65.9, 38.9, 27.1. HRMS (ESI) m/z: [M+H]+ Calcd. for $C_{20}H_{21}O_2^+$ 293.1536; Found: 293.1538. ^{1}H and ^{13}C NMR are in agreement with previously reported literature values.[5]

2.6 Procedure for the synthesis of Et_3Si-Bpin

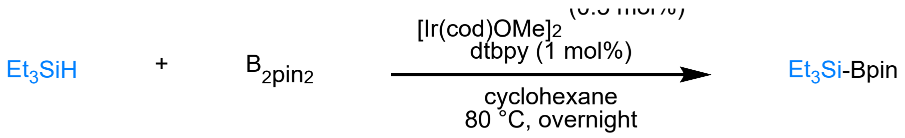

In air, $[Ir(cod)OMe]_2$ (13.1 mg, 0.02 mmol, 0.5 mol%), dtbpy (10.8 mg, 0.04 mmol, 1 mol%) and B_2pin_2 (1.02 g, 4.0 mmol, 1.0 equiv) were weighed into a 25 mL Schlenk tube equipped with a magnetic stirring bar. The tube was evacuated and filled with argon (three cycles). Then cyclohexane (1 mL) and Et_3SiH (4 equiv, 16.0 mmol) were added to the tube subsequently under argon atmosphere. The resulting dark brown reaction mixture was stirred vigorously at 80 ºC (oil bath) overnight. Upon completion of the reaction, the solvent was evaporated under reduced pressure and the residue was purified by flash column chromatography (silica gel, PE, R_f = 0.2) to afford the pure desired silylborane in 25% yield (233.6 mg) as a colorless oil. ^{1}H NMR (500 MHz, Chloroform-d): δ 1.23 (s, 12H), 0.96 (t, J = 7.9 Hz, 9H), 0.59 (q, J = 7.9 Hz, 6H). ^{1}H NMR are in agreement with previously reported literature values.[6]

3. Characterization of substrates

tert-butyl (1,3-diphenylprop-2-yn-1-yl) carbonate (1a)

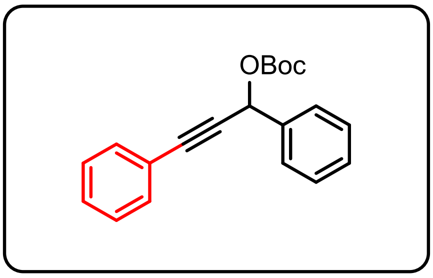

Following the **general procedure A** on 10 mmol scale, yellow solid, m.p. 76-78 °C, yield: 70% (2165 mg, 2 steps), column chromatography (silica gel, PE: EA = 100:1, v/v).

^{1}H NMR (500 MHz, Chloroform-*d*) δ 7.62 (dt, J = 8.4, 2.1 Hz, 2H), 7.49 – 7.46 (m, 2H), 7.40 (dddd, J = 10.8, 6.9, 4.6, 2.4 Hz, 3H), 7.34 – 7.29 (m, 3H), 6.49 (s, 1H), 1.51 (s, 9H).

^{13}C NMR (126 MHz, Chloroform-*d*) δ 152.6, 137.0, 131.9, 129.0, 128.8, 128.7, 128.3, 127.8, 122.2, 87.6, 85.4, 83.0, 69.1, 27.8.

HRMS (ESI) m/z: $[M+H]^+$ Calcd. for $C_{20}H_{21}O_3^+$ 309.1485; Found: 309.1485.

tert-butyl (1-phenyl-3-(p-tolyl)prop-2-yn-1-yl) carbonate (1b)

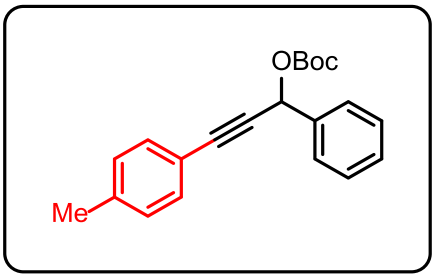

Following the **general procedure A** on 3 mmol scale, yellow oil, yield: 71% (682.1 mg, 2 steps), column chromatography (silica gel, PE: EA = 100:1, v/v).

^{1}H NMR (500 MHz, Chloroform-*d*) δ 7.65 – 7.61 (m, 2H), 7.43 – 7.35 (m, 5H), 7.14 – 7.11 (m, 2H), 6.50 (s, 1H), 2.35 (s, 3H), 1.51 (s, 9H).

^{13}C NMR (126 MHz, Chloroform-*d*) δ 152.6, 139.0, 137.1, 131.8, 129.1, 129.0, 128.7, 127.9, 119.1, 87.9, 84.7, 83.0, 69.2, 27.8, 21.6.

HRMS (ESI) m/z: $[M+H]^+$ Calcd. for $C_{21}H_{23}O_3^+$ 323.1642; Found: 323.1637.

tert-butyl (3-(4-isopropylphenyl)-1-phenylprop-2-yn-1-yl) carbonate (1c)

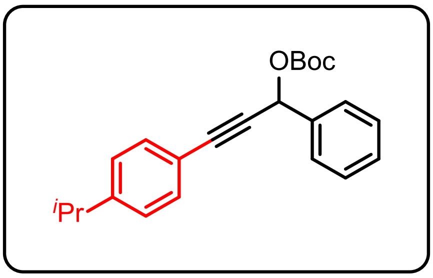

Following the **general procedure A** on 3 mmol scale, yellow oil, yield: 63% (657.7 mg, 2 steps), column chromatography (silica gel, PE: EA = 100:1, v/v).

^{1}H NMR (500 MHz, Chloroform-*d*) δ 7.65 – 7.61 (m, 2H), 7.43 – 7.35 (m, 5H), 7.20 – 7.16 (m, 2H), 6.50 (s, 1H), 2.90 (p, J = 7.0 Hz, 1H), 1.51 (s, 9H), 1.24 (d, J = 6.9 Hz, 6H).

^{13}C NMR (126 MHz, Chloroform-*d*) δ 152.6, 149.9, 137.1, 131.9, 129.0, 128.7, 127.9, 126.4, 119.5, 87.9, 84.6, 83.0, 69.2, 34.1, 27.8, 23.8.

HRMS (ESI) m/z: $[M+H]^+$ Calcd. for $C_{23}H_{27}O_3^+$ 351.1955; Found: 351.1955.

tert-butyl (3-(4-butylphenyl)-1-phenylprop-2-yn-1-yl) carbonate (1d)

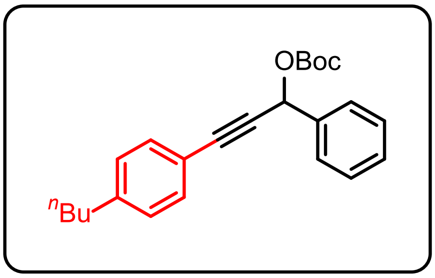

Following the **general procedure A** on 3 mmol scale, yellow oil, yield: 77% (840.9 mg, 2 steps), column chromatography (silica gel, PE: EA = 100:1, v/v).

^{1}H NMR (500 MHz, Chloroform-*d*) δ 7.66 – 7.62 (m, 2H), 7.43 – 7.36 (m, 5H), 7.15 – 7.12 (m, 2H), 6.51 (s, 1H), 2.63 – 2.59 (m, 2H), 1.65 – 1.57 (m, 2H), 1.52 (s, 9H), 1.35 (h, J = 7.4 Hz, 2H), 0.93 (t, J = 7.3 Hz, 3H).

^{13}C NMR (126 MHz, Chloroform-*d*) δ 152.6, 144.0, 137.1, 131.8, 129.0, 128.7, 128.4, 127.9, 119.3, 87.9, 84.7, 83.0, 69.2, 35.6, 33.4, 27.8, 22.3, 14.0.

HRMS (ESI) m/z: $[M+H]^+$ Calcd. for $C_{24}H_{29}O_3^+$ 365.2111; Found: 365.2109.

3-([1,1'-biphenyl]-4-yl)-1-phenylprop-2-yn-1-yl tert-butyl carbonate (1e)

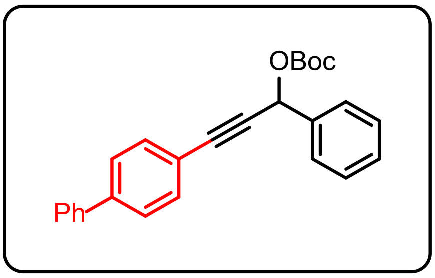

Following the **general procedure A** on 3 mmol scale, white solid, m.p. 96-98 ºC, yield: 82% (949.9 mg, 2 steps), column chromatography (silica gel, PE: EA = 80:1, v/v).

^{1}H NMR (500 MHz, Chloroform-*d*) δ 7.67 – 7.64 (m, 2H), 7.61 – 7.58 (m, 2H), 7.56 (s, 4H), 7.48 – 7.35 (m, 6H), 6.53 (s, 1H), 1.53 (s, 9H).

^{13}C NMR (126 MHz, Chloroform-*d*) δ 152.6, 141.6, 140.3, 137.0, 132.4, 129.1, 128.9, 128.7, 127.9, 127.8, 127.1, 127.0, 121.1, 87.6, 86.0, 83.1, 69.2, 27.8.

HRMS (ESI) m/z: $[M+H]^+$ Calcd. for $C_{26}H_{25}O_3^+$ 385.1798; Found: 385.1798.

tert-butyl (3-(4-fluorophenyl)-1-phenylprop-2-yn-1-yl) carbonate (1f)

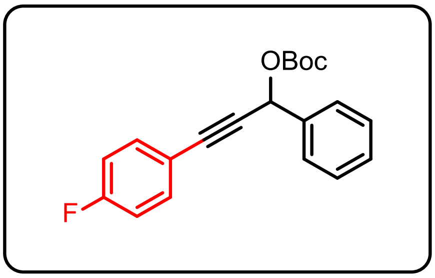

Following the **general procedure A** on 3 mmol scale, yellow oil, yield: 71% (697.6 mg, 2 steps), column chromatography (silica gel, PE: EA = 100:1, v/v).

^{1}H NMR (500 MHz, Chloroform-*d*) δ 7.63 – 7.58 (m, 2H), 7.48 – 7.43 (m, 2H), 7.43 – 7.37 (m, 3H), 7.03 – 6.98 (m, 2H), 6.47 (s, 1H), 1.50 (s, 9H).

^{13}C NMR (126 MHz, Chloroform-*d*) δ 162.8 (d, J = 250.1 Hz), 152.6, 136.9, 133.9 (d, J = 8.6 Hz), 129.1, 128.7, 127.8, 118.3 (d, J = 3.6 Hz), 115.6 (d, J = 22.2 Hz), 86.6, 85.1, 83.1, 69.0, 27.8.

^{19}F NMR (471 MHz, Chloroform-*d*) δ -110.1.

HRMS (ESI) m/z: $[M+H]^+$ Calcd. for $C_{20}H_{20}FO_3^+$ 327.1391; Found: 327.1391.

tert-butyl (3-(4-chlorophenyl)-1-phenylprop-2-yn-1-yl) carbonate (1g)

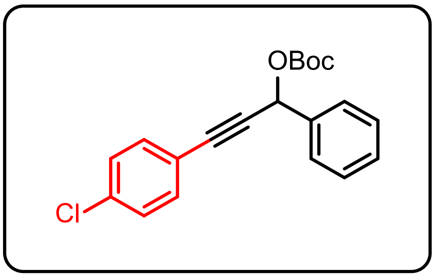

Following the **general procedure A** on 3 mmol scale, white solid, m.p. 84-86 °C, yield: 88% (903.7 mg, 2 steps), column chromatography (silica gel, PE: EA = 100:1, v/v).

^{1}H NMR (500 MHz, Chloroform-*d*) δ 7.62 – 7.59 (m, 2H), 7.43 – 7.37 (m, 5H), 7.31 – 7.28 (m, 2H), 6.48 (s, 1H), 1.51 (s, 9H).

^{13}C NMR (126 MHz, Chloroform-*d*) δ 152.5, 136.8, 134.9, 133.1, 129.2, 128.8, 128.7, 127.8, 120.7, 86.5, 86.4, 83.2, 69.0, 27.8.

HRMS (ESI) m/z: $[M+H]^+$ Calcd. for $C_{20}H_{20}ClO_3^+$ 343.1095; Found: 343.1091.

3-(4-bromophenyl)-1-phenylprop-2-yn-1-yl tert-butyl carbonate (1h)

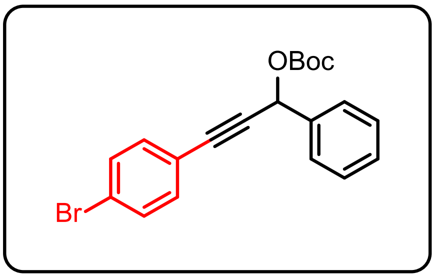

Following the **general procedure A** on 3 mmol scale, white solid, m.p. 95-97 °C, yield: 77% (895.9 mg, 2 steps), column chromatography (silica gel, PE: EA = 100:1, v/v).

^{1}H NMR (500 MHz, Chloroform-*d*) δ 7.63 – 7.60 (m, 2H), 7.47 – 7.37 (m, 5H), 7.35 – 7.32 (m, 2H), 6.49 (s, 1H), 1.51 (s, 9H).

^{13}C NMR (126 MHz, Chloroform-*d*) δ 152.6, 136.7, 133.3, 131.6, 129.2, 128.8, 127.8, 123.2, 121.1, 86.6, 86.5, 83.2, 69.0, 27.8.

HRMS (ESI) m/z: $[M+H]^+$ Calcd. for $C_{20}H_{20}BrO_3^+$ 387.0590; Found: 387.0586.

tert-butyl (3-(2-fluorophenyl)-1-phenylprop-2-yn-1-yl) carbonate (1i)

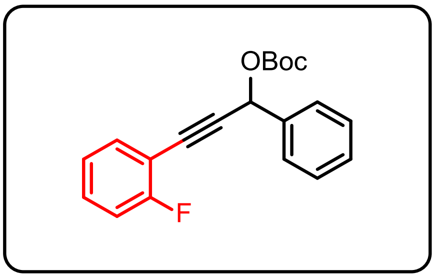

Following the **general procedure A** on 3 mmol scale, yellow oil, yield: 98% (958.2 mg, 2 steps), column chromatography (silica gel, PE: EA = 100:1, v/v).

^{1}H NMR (500 MHz, Chloroform-*d*) δ 7.65 – 7.62 (m, 2H), 7.46 (td, J = 7.3, 1.8 Hz, 1H), 7.43 – 7.37 (m, 3H), 7.32 (dddd, J = 8.3, 7.3, 5.3, 1.8 Hz, 1H), 7.11 – 7.05 (m, 2H), 6.51 (s, 1H), 1.51 (s, 9H).

^{13}C NMR (126 MHz, Chloroform-*d*) δ 163.0 (d, J = 252.6 Hz), 152.5, 136.6, 133.7, 130.6 (d, J = 7.9 Hz), 129.1, 128.7, 128.0, 123.9 (d, J = 3.7 Hz), 115.5 (d, J = 20.8 Hz), 110.8 (d, J = 15.6 Hz), 90.5, 83.1, 81.1, 69.0, 27.8.

^{19}F NMR (471 MHz, Chloroform-*d*) δ -109.3.

HRMS (ESI) m/z: $[M+H]^+$ Calcd. for $C_{20}H_{20}FO_3^+$ 327.1391; Found: 327.1391.

tert-butyl (3-(2-chlorophenyl)-1-phenylprop-2-yn-1-yl) carbonate (1j)

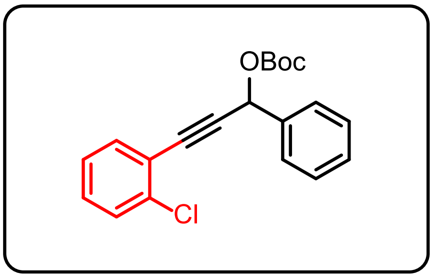

Following the **general procedure A** on 3 mmol scale, yellow oil, yield: 98% (1005.6 mg, 2 steps), column chromatography (silica gel, PE: EA = 100:1, v/v).

^{1}H NMR (500 MHz, Chloroform-*d*) δ 7.68 – 7.65 (m, 2H), 7.51 (dd, J = 7.6, 1.8 Hz, 1H), 7.44 – 7.38 (m, 4H), 7.28 – 7.25 (m, 1H), 7.21 (td, J = 7.6, 1.3 Hz, 1H), 6.53 (s, 1H), 1.51 (s, 9H).

^{13}C NMR (126 MHz, Chloroform-*d*) δ 152.6, 136.5, 136.4, 133.6, 129.9, 129.3, 129.2, 128.7, 128.1, 126.4, 122.2, 90.4, 84.4, 83.1, 69.1, 27.8.

HRMS (ESI) m/z: $[M+H]^+$ Calcd. for $C_{20}H_{20}ClO_3^+$ 343.1095; Found: 343.1095.

3-(2-bromophenyl)-1-phenylprop-2-yn-1-yl tert-butyl carbonate (1k)

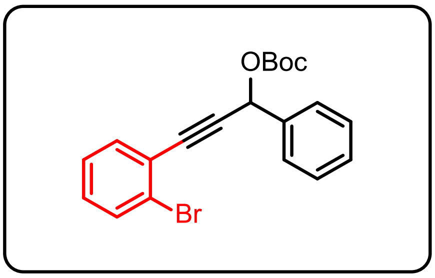

Following the **general procedure A** on 3 mmol scale, yellow oil, yield: 99% (1146.1 mg, 2 steps), column chromatography (silica gel, PE: EA = 100:1, v/v).

^{1}H NMR (500 MHz, Chloroform-*d*) δ 7.69 – 7.66 (m, 2H), 7.58 (dd, J = 8.1, 1.2 Hz, 1H), 7.50 (dd, J = 7.6, 1.8 Hz, 1H), 7.44 – 7.37 (m, 3H), 7.28 – 7.24 (m, 1H), 7.18 (td, J = 7.7, 1.7 Hz, 1H), 6.52 (s, 1H), 1.51 (s, 9H).

^{13}C NMR (126 MHz, Chloroform-*d*) δ 152.6, 136.5, 133.7, 132.5, 130.0, 129.1, 128.7, 128.1, 127.0, 125.8, 124.4, 89.8, 86.1, 83.1, 69.1, 27.8.

HRMS (ESI) m/z: $[M+H]^+$ Calcd. for $C_{20}H_{20}BrO_3^+$ 387.0590; Found: 387.0590.

tert-butyl (3-(3-fluorophenyl)-1-phenylprop-2-yn-1-yl) carbonate (1l)

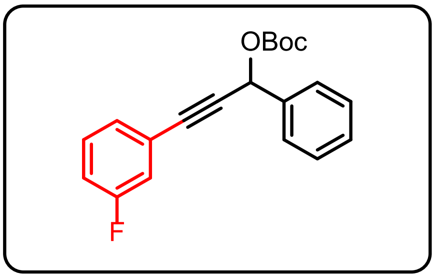

Following the **general procedure A** on 5 mmol scale, colorless oil, yield: 91% (1476.2 mg, 2 steps), column chromatography (silica gel, PE: EA = 100:1, v/v).

^{1}H NMR (500 MHz, Chloroform-*d*) δ 7.63 – 7.59 (m, 2H), 7.44 – 7.38 (m, 3H), 7.29 – 7.24 (m, 2H), 7.19 – 7.15 (m, 1H), 7.07 – 7.02 (m, 1H), 6.48 (s, 1H), 1.51 (s, 9H).

^{13}C NMR (126 MHz, Chloroform-*d*) δ 162.3 (d, J = 246.8 Hz), 152.5, 136.7, 129.9 (d, J = 8.6 Hz), 129.2, 128.8, 127.82, 127.78, 124.0 (d, J = 9.5 Hz), 118.7 (d, J = 23.1 Hz), 116.2 (d, J = 21.2 Hz), 86.34, 86.28 (d, J = 3.4 Hz), 83.2, 68.9, 27.8.

^{19}F NMR (471 MHz, Chloroform-*d*) δ -112.8.

HRMS (ESI) m/z: $[M+H]^+$ Calcd. for $C_{20}H_{20}FO_3^+$ 327.1391; Found: 327.1388.

tert-butyl (3-(3-chlorophenyl)-1-phenylprop-2-yn-1-yl) carbonate (1m)

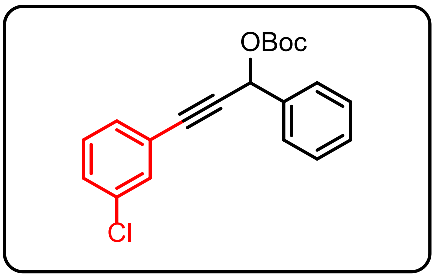

Following the **general procedure A** on 3 mmol scale, yellow solid, m.p. 84-86 °C, yield: 99% (1018.6 mg, 2 steps), column chromatography (silica gel, PE: EA = 100:1, v/v).

^{1}H NMR (500 MHz, Chloroform-*d*) δ 7.63 – 7.59 (m, 2H), 7.47 (t, J = 1.8 Hz, 1H), 7.44 – 7.38 (m, 3H), 7.36 (dt, J = 7.6, 1.4 Hz, 1H), 7.31 (ddd, J = 8.1, 2.1, 1.2 Hz, 1H), 7.24 (t, J = 7.8 Hz, 1H), 6.49 (s, 1H), 1.52 (s, 9H).

^{13}C NMR (126 MHz, Chloroform-*d*) δ 152.5, 136.7, 134.2, 131.8, 130.0, 129.6, 129.2, 129.1, 128.8, 127.8, 123.9, 86.7, 86.1, 83.2, 68.9, 27.8.

HRMS (ESI) m/z: $[M+H]^+$ Calcd. for $C_{20}H_{20}ClO_3^+$ 343.1095; Found: 343.1095.

3-(3-bromophenyl)-1-phenylprop-2-yn-1-yl tert-butyl carbonate (1n)

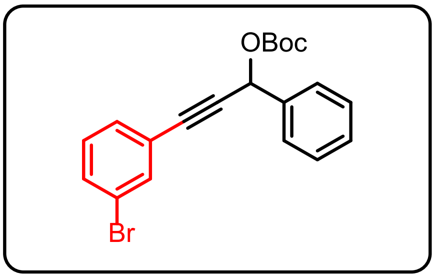

Following the **general procedure A** on 3 mmol scale, colorless oil, yield: 89% (1030.8 mg, 2 steps), column chromatography (silica gel, PE: EA = 100:1, v/v).

^{1}H NMR (500 MHz, Chloroform-*d*) δ 7.63 – 7.58 (m, 3H), 7.48 – 7.45 (m, 1H), 7.44 – 7.38 (m, 4H), 7.18 (t, J = 7.9 Hz, 1H), 6.47 (s, 1H), 1.51 (s, 9H).

^{13}C NMR (126 MHz, Chloroform-*d*) δ 152.5, 136.6, 134.6, 132.0, 130.5, 129.8, 129.2, 128.8, 127.8, 124.1, 122.1, 86.8, 86.0, 83.2, 68.9, 27.8.

HRMS (ESI) m/z: $[M+H]^+$ Calcd. for $C_{20}H_{20}BrO_3^+$ 387.0590; Found: 387.0591.

ethyl 4-(3-((tert-butoxycarbonyl)oxy)-3-phenylprop-1-yn-1-yl)benzoate (1o)

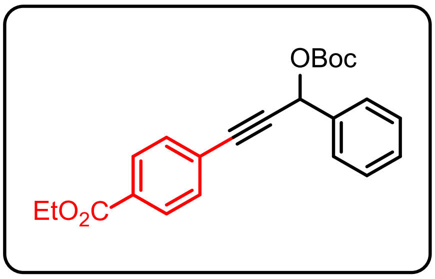

Following the **general procedure A** on 3 mmol scale, white solid, m.p. 94-96 ºC, yield: 80% (915.8 mg, 2 steps), column chromatography (silica gel, PE: EA = 50:1, v/v).

^{1}H NMR (500 MHz, Chloroform-*d*) δ 8.01 – 7.97 (m, 2H), 7.63 – 7.58 (m, 2H), 7.54 – 7.51 (m, 2H), 7.43 – 7.36 (m, 3H), 6.49 (s, 1H), 4.37 (q, J = 7.1 Hz, 2H), 1.51 (s, 9H), 1.39 (t, J = 7.1 Hz, 3H).

^{13}C NMR (126 MHz, Chloroform-*d*) δ 166.0, 152.5, 136.6, 131.8, 130.4, 129.4, 129.2, 128.8, 127.8, 126.7, 88.2, 86.8, 83.2, 68.9, 61.2, 27.8, 14.3.

HRMS (ESI) m/z: $[M+H]^+$ Calcd. for $C_{23}H_{25}O_5^+$ 381.1697; Found: 381.1698.

methyl 4-(3-((tert-butoxycarbonyl)oxy)-3-phenylprop-1-yn-1-yl)benzoate (1p)

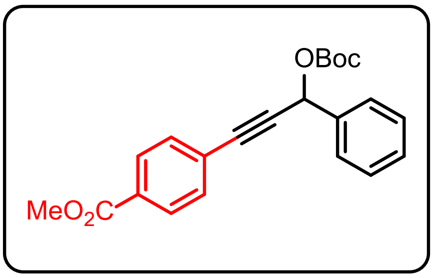

Following the **general procedure A** on 3 mmol scale, white solid, m.p. 130-132 ºC, yield: 75% (818.6 mg, 2 steps), column chromatography (silica gel, PE: EA = 50:1, v/v).

^{1}H NMR (500 MHz, Chloroform-*d*) δ 8.01 – 7.96 (m, 2H), 7.63 – 7.58 (m, 2H), 7.55 – 7.51 (m, 2H), 7.43 – 7.35 (m, 3H), 6.50 (s, 1H), 3.90 (s, 3H), 1.50 (s, 9H).

^{13}C NMR (126 MHz, Chloroform-*d*) δ 166.4, 152.5, 136.6, 131.8, 130.1, 129.4, 129.2, 128.8, 127.8, 126.8, 88.3, 86.7, 83.2, 68.9, 52.3, 27.8.

HRMS (ESI) m/z: $[M+H]^+$ Calcd. for $C_{22}H_{23}O_5^+$ 367.1540; Found: 367.1542.

3-(4-acetylphenyl)-1-phenylprop-2-yn-1-yl tert-butyl carbonate (1q)

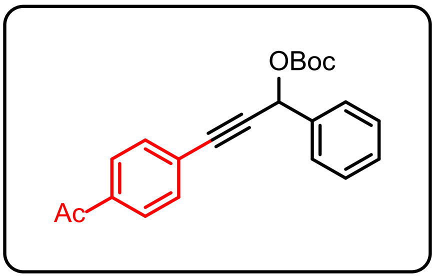

Following the **general procedure A** on 3 mmol scale, yellow oil, yield: 24% (249.8 mg, 2 steps), column chromatography (silica gel, PE: EA = 50:1, v/v).

^{1}H NMR (500 MHz, Chloroform-*d*) δ 7.90 (d, J = 8.2 Hz, 2H), 7.60 (dd, J = 8.0, 1.6 Hz, 2H), 7.55 (d, J = 8.4 Hz, 2H), 7.40 (qd, J = 7.8, 7.0, 3.8 Hz, 3H), 6.49 (s, 1H), 2.59 (s, 3H), 1.50 (s, 9H).

^{13}C NMR (126 MHz, Chloroform-*d*) δ 197.3, 152.5, 136.7, 136.6, 132.0, 129.2, 128.8, 128.2, 127.8, 127.0, 88.6, 86.7, 83.3, 68.9, 27.8, 26.7.

HRMS (ESI) m/z: $[M+H]^+$ Calcd. for $C_{22}H_{23}O_4^+$ 351.1591; Found: 351.1590.

tert-butyl (3-(4-cyanophenyl)-1-phenylprop-2-yn-1-yl) carbonate (1r)

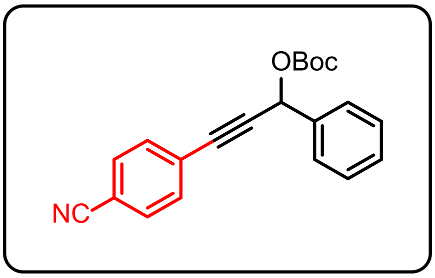

Following the **general procedure A** on 3 mmol scale, yellow oil, yield: 49% (489.6 mg, 2 steps), column chromatography (silica gel, PE: EA = 50:1, v/v).

^{1}H NMR (500 MHz, Chloroform-*d*) δ 7.62 – 7.57 (m, 4H), 7.56 – 7.53 (m, 2H), 7.44 – 7.38 (m, 3H), 6.48 (s, 1H), 1.50 (s, 9H).

^{13}C NMR (126 MHz, Chloroform-*d*) δ 152.4, 136.3, 132.4, 132.0, 129.3, 128.9, 127.8, 127.0, 118.3, 112.2, 89.8, 85.7, 83.4, 68.7, 27.8.

HRMS (ESI) m/z: $[M+H]^+$ Calcd. for $C_{21}H_{20}NO_3^+$ 334.1438; Found: 334.1437.

tert-butyl (3-(3-cyanophenyl)-1-phenylprop-2-yn-1-yl) carbonate (1s)

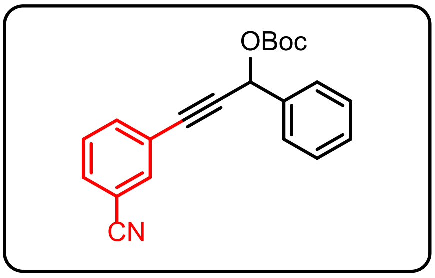

Following the **general procedure A** on 3 mmol scale, yellow solid, m.p. 116-118 °C, yield: 55% (545.5 mg, 2 steps), column chromatography (silica gel, PE: EA = 50:1, v/v).

^{1}H NMR (500 MHz, Chloroform-*d*) δ 7.74 (t, J = 1.7 Hz, 1H), 7.67 (dt, J = 7.9, 1.4 Hz, 1H), 7.60 (ddt, J = 9.4, 7.8, 1.5 Hz, 3H), 7.45 – 7.37 (m, 4H), 6.46 (s, 1H), 1.51 (s, 9H).

^{13}C NMR (126 MHz, Chloroform-*d*) δ 152.5, 136.4, 135.9, 135.2, 132.0, 129.30, 129.29, 128.8, 127.7, 123.8, 117.9, 112.9, 88.0, 85.0, 83.4, 68.7, 27.8.

HRMS (ESI) m/z: $[M+H]^+$ Calcd. for $C_{21}H_{20}NO_3^+$ 334.1438; Found: 334.1438.

tert-butyl (1-phenyl-3-(4-(trifluoromethyl)phenyl)prop-2-yn-1-yl) carbonate (1t)

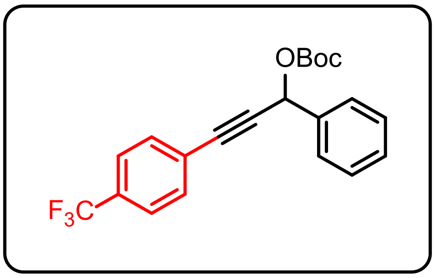

Following the **general procedure A** on 3 mmol scale, white solid, m.p. 85-87 °C, yield: 79% (892.2 mg, 2 steps), column chromatography (silica gel, PE: EA = 100:1, v/v).

^{1}H NMR (500 MHz, Chloroform-*d*) δ 7.63 – 7.60 (m, 2H), 7.58 (s, 4H), 7.44 – 7.39 (m, 3H), 6.50 (s, 1H), 1.51 (s, 9H).

^{13}C NMR (126 MHz, Chloroform-*d*) δ 152.5, 136.5, 132.1, 130.5 (q, J = 32.7 Hz), 129.2, 128.80, 127.8, 126.0, 125.2 (q, J = 3.7 Hz), 123.8 (q, J = 272.3 Hz), 87.9, 86.1, 83.3, 68.8, 27.8.

^{19}F NMR (471 MHz, Chloroform-*d*) δ -62.9.

HRMS (ESI) m/z: $[M+H]^+$ Calcd. for $C_{21}H_{20}F_3O_3^+$ 377.1359; Found: 393.1360.

methyl 2-(3-((tert-butoxycarbonyl)oxy)-3-phenylprop-1-yn-1-yl)benzoate (1u)

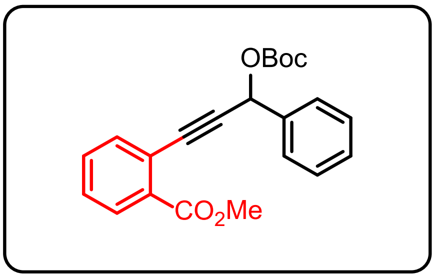

Following the **general procedure A** on 3 mmol scale, yellow oil, yield: 34% (375 mg, 2 steps), column chromatography (silica gel, PE: EA = 50:1, v/v).

^{1}H NMR (500 MHz, Chloroform-*d*) δ 7.94 (dd, J = 7.9, 1.4 Hz, 1H), 7.68 – 7.63 (m, 2H), 7.58 (dd, J = 7.7, 1.4 Hz, 1H), 7.46 (td, J = 7.6, 1.5 Hz, 1H), 7.43 – 7.35 (m, 4H), 6.53 (s, 1H), 3.82 (s, 3H), 1.51 (s, 9H).

^{13}C NMR (126 MHz, Chloroform-*d*) δ 166.7, 152.6, 136.9, 134.3, 132.3, 131.6, 130.5, 129.1, 128.7, 128.5, 128.0, 122.5, 90.1, 86.2, 83.0, 69.2, 52.1, 27.8.

HRMS (ESI) m/z: $[M+H]^+$ Calcd. for $C_{22}H_{23}O_5^+$ 367.1540; Found: 367.1539.

ethyl 2-(3-((tert-butoxycarbonyl)oxy)-3-phenylprop-1-yn-1-yl)benzoate (1v)

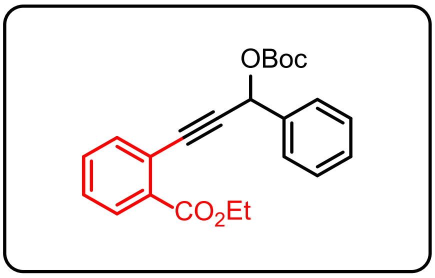

Following the **general procedure A** on 3 mmol scale, yellow oil, yield: 51% (579 mg, 2 steps), column chromatography (silica gel, PE: EA = 50:1, v/v).

^{1}H NMR (500 MHz, Chloroform-*d*) δ 7.94 (dd, J = 7.9, 1.5 Hz, 1H), 7.68 – 7.63 (m, 2H), 7.58 (dd, J = 7.7, 1.3 Hz, 1H), 7.45 (td, J = 7.6, 1.4 Hz, 1H), 7.42 – 7.34 (m, 4H), 6.54 (s, 1H), 4.33 (qd, J = 7.1, 4.4 Hz, 2H), 1.50 (s, 9H), 1.31 (t, J = 7.1 Hz, 3H).

^{13}C NMR (126 MHz, Chloroform-*d*) δ 166.2, 152.6, 136.8, 134.4, 132.6, 131.5, 130.4, 129.0, 128.7, 128.5, 128.0, 122.4, 90.0, 86.3, 83.0, 69.2, 61.3, 27.8, 14.2.

HRMS (ESI) m/z: $[M+H]^+$ Calcd. for $C_{23}H_{25}O_5^+$ 381.1697; Found: 381.1697.

tert-butyl (3-(4-(methylthio)phenyl)-1-phenylprop-2-yn-1-yl) carbonate (1w)

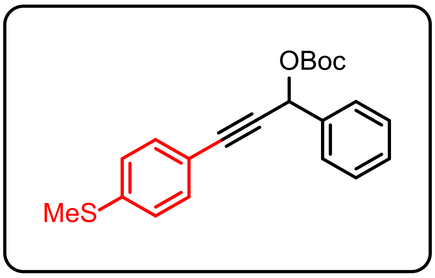

Following the **general procedure A** on 3 mmol scale, yellow oil, yield: 51% (542 mg, 2 steps), column chromatography (silica gel, PE: EA = 80:1, v/v).

^{1}H NMR (500 MHz, Chloroform-*d*) δ 7.63 – 7.59 (m, 2H), 7.43 – 7.36 (m, 5H), 7.18 – 7.15 (m, 2H), 6.48 (s, 1H), 2.48 (s, 3H), 1.50 (s, 9H).

^{13}C NMR (126 MHz, Chloroform-*d*) δ 152.6, 140.1, 137.0, 132.2, 129.1, 128.7, 127.8, 125.7, 118.4, 87.4, 85.4, 83.1, 69.2, 27.8, 15.3.

HRMS (ESI) m/z: $[M+H]^+$ Calcd. for $C_{21}H_{23}O_3S^+$ 355.1362; Found: 355.1362.

tert-butyl (1-phenyl-3-(4-(trifluoromethoxy)phenyl)prop-2-yn-1-yl) carbonate (1x)

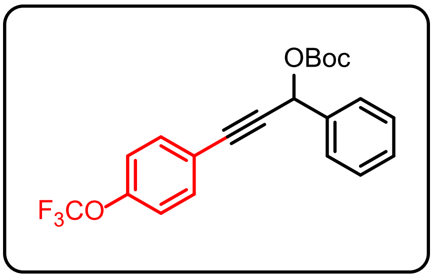

Following the **general procedure A** on 3 mmol scale, yellow oil, yield: 99% (983.3 mg, 2 steps), column chromatography (silica gel, PE: EA = 100:1, v/v).

^{1}H NMR (500 MHz, Chloroform-*d*) δ 7.63 – 7.59 (m, 2H), 7.52 – 7.48 (m, 2H), 7.44 – 7.36 (m, 3H), 7.17 (d, J = 8.1 Hz, 2H), 6.49 (s, 1H), 1.51 (s, 9H).

^{13}C NMR (126 MHz, Chloroform-*d*) δ 152.6, 149.3 (d, J = 1.7 Hz), 136.7, 133.5, 129.2, 128.8, 127.8, 120.9, 120.8, 120.4 (q, J = 257.8 Hz), 86.3, 86.1, 83.2, 68.9, 27.8.

^{19}F NMR (471 MHz, Chloroform-*d*) δ -57.8.

HRMS (ESI) m/z: $[M+H]^+$ Calcd. for $C_{21}H_{20}F_3O_4^+$ 393.1308; Found: 393.1308.

tert-butyl (3-(3-chloro-4-methylphenyl)-1-phenylprop-2-yn-1-yl) carbonate (1y)

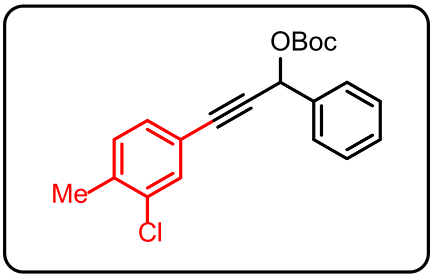

Following the **general procedure A** on 3 mmol scale, yellow oil, yield: 96% (1030.6 mg, 2 steps), column chromatography (silica gel, PE: EA = 100:1, v/v).

^{1}H NMR (500 MHz, Chloroform-*d*) δ 7.62 – 7.59 (m, 2H), 7.46 (d, J = 1.7 Hz, 1H), 7.43 – 7.37 (m, 3H), 7.26 (dd, J = 7.8, 1.7 Hz, 1H), 7.16 (d, J = 7.8 Hz, 1H), 6.47 (s, 1H), 2.37 (s, 3H), 1.51 (s, 9H).

^{13}C NMR (126 MHz, Chloroform-*d*) δ 152.6, 137.2, 136.8, 134.2, 132.2, 130.8, 130.1, 129.1, 128.7, 127.8, 121.1, 86.3, 85.8, 83.1, 69.0, 27.8, 20.1.

HRMS (ESI) m/z: $[M+H]^+$ Calcd. for $C_{21}H_{22}ClO_3^+$ 357.1252; Found: 357.1252.

tert-butyl (3-(4-fluoro-3-methylphenyl)-1-phenylprop-2-yn-1-yl) carbonate (1z)

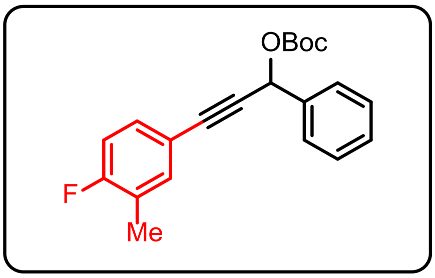

Following the **general procedure A** on 3 mmol scale, yellow oil, yield: 86% (881.8 mg, 2 steps), column chromatography (silica gel, PE: EA = 100:1, v/v).

^{1}H NMR (500 MHz, Chloroform-*d*) δ 7.63 – 7.60 (m, 2H), 7.43 – 7.37 (m, 3H), 7.33 – 7.31 (m, 1H), 7.30 – 7.26 (m, 1H), 6.96 – 6.92 (m, 1H), 6.47 (s, 1H), 2.24 (d, J = 2.0 Hz, 3H), 1.51 (s, 9H).

^{13}C NMR (126 MHz, Chloroform-*d*) δ 161.5 (d, J = 248.8 Hz), 152.6, 137.0, 135.2 (d, J = 5.5 Hz), 131.2 (d, J = 8.4 Hz), 129.1, 128.7, 127.8, 125.2 (d, J = 18.1 Hz), 117.9 (d, J = 3.7 Hz), 115.2 (d, J = 23.0 Hz), 86.9, 84.7, 83.0, 69.1, 27.8, 14.4 (d, J = 3.5 Hz).

^{19}F NMR (471 MHz, Chloroform-*d*) δ -114.6.

HRMS (ESI) m/z: $[M+H]^+$ Calcd. for $C_{21}H_{22}FO_3^+$ 341.1547; Found: 341.1544.

tert-butyl (3-(5-chloro-2-methoxyphenyl)-1-phenylprop-2-yn-1-yl) carbonate (1aa)

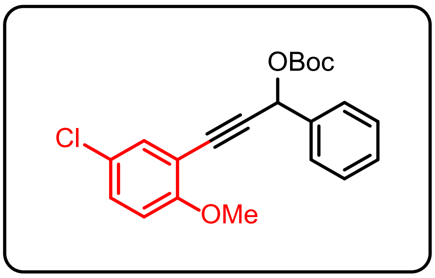

Following the **general procedure A** on 3 mmol scale, yellow oil, yield: 62% (695.4 mg, 2 steps), column chromatography (silica gel, PE: EA = 80:1, v/v).

^{1}H NMR (500 MHz, Chloroform-*d*) δ 7.66 – 7.63 (m, 2H), 7.43 – 7.36 (m, 4H), 7.24 (dd, J = 8.9, 2.7 Hz, 1H), 6.78 (d, J = 8.9 Hz, 1H), 6.52 (s, 1H), 3.84 (s, 3H), 1.50 (s, 9H).

^{13}C NMR (126 MHz, Chloroform-*d*) δ 159.1, 152.6, 136.8, 133.2, 130.0, 129.1, 128.7, 128.0, 125.1, 113.0, 111.9, 90.4, 83.0, 82.8, 69.1, 56.1, 27.8.

HRMS (ESI) m/z: $[M+H]^+$ Calcd. for $C_{21}H_{22}ClO_4^+$ 373.1201; Found: 373.1201.

tert-butyl (3-(3,5-dichlorophenyl)-1-phenylprop-2-yn-1-yl) carbonate (1ab)

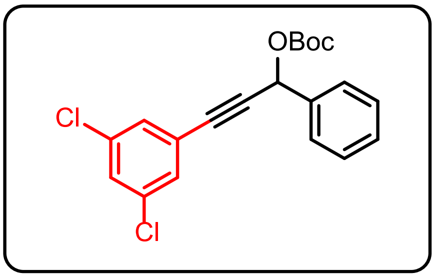

Following the **general procedure A** on 3 mmol scale, yellow oil, yield: 85% (953.6 mg, 2 steps), column chromatography (silica gel, PE: EA = 100:1, v/v).

^{1}H NMR (500 MHz, Chloroform-*d*) δ 7.60 – 7.56 (m, 2H), 7.44 – 7.38 (m, 3H), 7.34 (d, J = 1.9 Hz, 2H), 7.33 (d, J = 1.9 Hz, 1H), 6.46 (s, 1H), 1.51 (s, 9H).

^{13}C NMR (126 MHz, Chloroform-*d*) δ 152.5, 136.4, 134.9, 130.1, 129.3, 129.2, 128.8, 127.8, 124.9, 87.9, 84.8, 83.3, 68.7, 27.8.

HRMS (ESI) m/z: $[M+H]^+$ Calcd. for $C_{20}H_{19}Cl_2O_3^+$ 377.0706; Found: 377.0708.

tert-butyl (3-(naphthalen-1-yl)-1-phenylprop-2-yn-1-yl) carbonate (1ac)

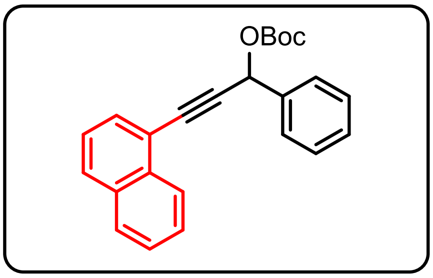

Following the **general procedure A** on 3 mmol scale, yellow oil, yield: 86% (920.4 mg, 2 steps), column chromatography (silica gel, PE: EA = 80:1, v/v).

^{1}H NMR (500 MHz, Chloroform-*d*) δ 8.34 (dd, J = 8.2, 1.3 Hz, 1H), 7.85 (dd, J = 8.3, 1.2 Hz, 2H), 7.75 – 7.72 (m, 3H), 7.55 (dddd, J = 22.0, 8.1, 6.9, 1.4 Hz, 2H), 7.48 – 7.39 (m, 4H), 6.65 (s, 1H), 1.55 (s, 9H).

^{13}C NMR (126 MHz, Chloroform-*d*) δ 152.7, 136.9, 133.4, 133.1, 130.9, 129.3, 129.2, 128.8, 128.3, 128.0, 127.0, 126.5, 126.2, 125.1, 119.8, 90.3, 85.9, 83.1, 69.4, 27.9.

HRMS (ESI) m/z: $[M+H]^+$ Calcd. for $C_{24}H_{23}O_3^+$ 359.1642; Found: 359.1642.

tert-butyl (1-phenyl-3-(thiophen-2-yl)prop-2-yn-1-yl) carbonate (1ad)

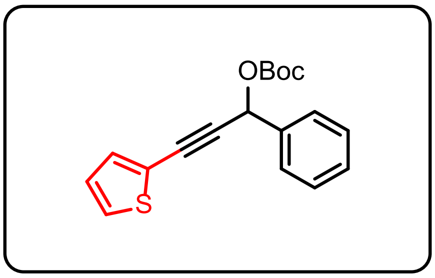

Following the **general procedure A** on 3 mmol scale, brown oil, yield: 22% (204.4 mg, 2 steps), column chromatography (silica gel, PE: EA = 80:1, v/v).

^{1}H NMR (500 MHz, Chloroform-*d*) δ 7.62 – 7.58 (m, 2H), 7.43 – 7.37 (m, 3H), 7.28 – 7.25 (m, 2H), 6.97 (dd, J = 5.1, 3.6 Hz, 1H), 6.50 (s, 1H), 1.51 (s, 9H).

^{13}C NMR (126 MHz, Chloroform-*d*) δ 152.5, 136.7, 133.0, 129.1, 128.8, 127.9, 127.8, 127.0, 122.0, 89.2, 83.1, 81.0, 69.1, 27.8.

HRMS (ESI) m/z: $[M+H]^+$ Calcd. for $C_{18}H_{19}O_3S^+$ 315.1049; Found: 315.1048.

tert-butyl (1-phenylhept-2-yn-1-yl) carbonate (1ae)

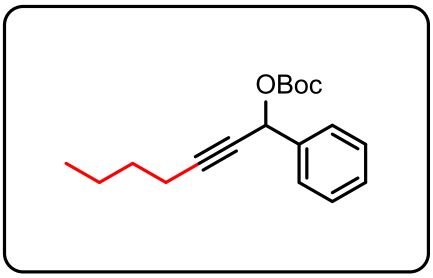

Following the **general procedure B** on 3 mmol scale, yellow oil, yield: 79% (683.5 mg, 2 steps), column chromatography (silica gel, PE: EA = 100:1, v/v).

^{1}H NMR (500 MHz, Chloroform-*d*) δ 7.56 – 7.52 (m, 2H), 7.39 – 7.33 (m, 3H), 6.24 (t, J = 2.1 Hz, 1H), 2.27 (td, J = 7.1, 2.1 Hz, 2H), 1.52 – 1.50 (m, 2H), 1.48 (s, 9H), 1.44 – 1.39 (m, 2H), 0.90 (t, J = 7.3 Hz, 3H).

^{13}C NMR (126 MHz, Chloroform-*d*) δ 152.7, 137.5, 128.8, 128.5, 127.7, 89.0, 82.7, 76.5, 69.1, 30.4, 27.8, 21.9, 18.6, 13.6.

HRMS (ESI) m/z: $[M+H]^+$ Calcd. for $C_{18}H_{25}O_3^+$ 289.1798; Found: 289.1799.

tert-butyl (1-phenylnon-2-yn-1-yl) carbonate (1af)

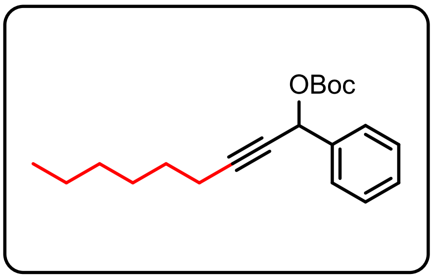

Following the **general procedure B** on 3 mmol scale, yellow oil, yield: 69% (653.8 mg, 2 steps), column chromatography (silica gel, PE: EA = 100:1, v/v).

^{1}H NMR (500 MHz, Chloroform-*d*) δ 7.56 – 7.52 (m, 2H), 7.39 – 7.33 (m, 3H), 6.24 (t, J = 2.1 Hz, 1H), 2.26 (td, J = 7.1, 2.1 Hz, 2H), 1.55 – 1.51 (m, 2H), 1.48 (s, 9H), 1.41 – 1.35 (m, 2H), 1.33 – 1.26 (m, 4H), 0.88 (t, J = 7.0 Hz, 3H).

^{13}C NMR (126 MHz, Chloroform-*d*) δ 152.7, 137.5, 128.8, 128.5, 127.7, 89.0, 82.7, 76.6, 69.1, 31.3, 28.5, 28.3, 27.8, 22.5, 18.9, 14.0.

HRMS (ESI) m/z: $[M+H]^+$ Calcd. for $C_{20}H_{29}O_3^+$ 317.2111; Found: 317.2111.

tert-butyl (1-(4-fluorophenyl)-3-phenylprop-2-yn-1-yl) carbonate (1ag)

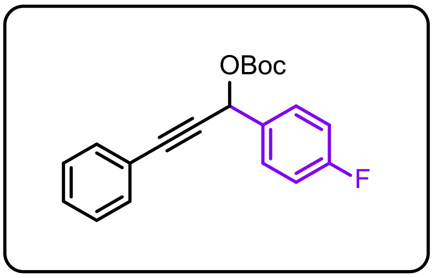

Following the **general procedure B** on 3 mmol scale, yellow oil, yield: 76% (742.1 mg, 2 steps), column chromatography (silica gel, PE: EA = 100:1, v/v).

^{1}H NMR (500 MHz, Chloroform-*d*) δ 7.64 – 7.59 (m, 2H), 7.50 – 7.46 (m, 2H), 7.35 – 7.29 (m, 3H), 7.12 – 7.06 (m, 2H), 6.47 (s, 1H), 1.51 (s, 9H).

^{19}F NMR (471 MHz, Chloroform-*d*) δ -112.4.

^{13}C NMR (126 MHz, Chloroform-*d*) δ 163.1 (d, J = 248.0 Hz), 152.5, 133.0 (d, J = 3.2 Hz), 131.9, 129.9 (d, J = 8.3 Hz), 128.9, 128.3, 122.0, 115.6 (d, J = 21.8 Hz), 87.8, 85.1, 83.2, 68.4, 27.8.

HRMS (ESI) m/z: $[M+H]^+$ Calcd. for $C_{20}H_{20}FO_3^+$ 327.1391; Found: 327.1391.

tert-butyl (1-(4-chlorophenyl)-3-phenylprop-2-yn-1-yl) carbonate (1ah)

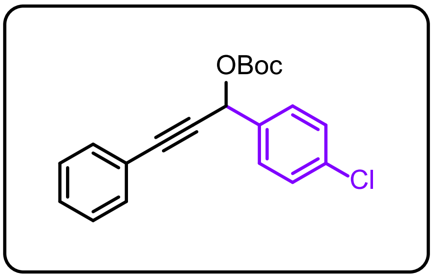

Following the **general procedure B** on 3 mmol scale, yellow solid, m.p. 62-64 ºC, yield: 86% (887.1 mg, 2 steps), column chromatography (silica gel, PE: EA = 100:1, v/v).

^{1}H NMR (500 MHz, Chloroform-*d*) δ 7.58 – 7.55 (m, 2H), 7.49 – 7.46 (m, 2H), 7.39 – 7.36 (m, 2H), 7.35 – 7.30 (m, 3H), 6.47 (s, 1H), 1.51 (s, 9H).

^{13}C NMR (126 MHz, Chloroform-*d*) δ 152.5, 135.6, 135.0, 131.9, 129.3, 129.0, 128.9, 128.4, 121.9, 88.0, 84.9, 83.3, 68.3, 27.8.

HRMS (ESI) m/z: $[M+H]^+$ Calcd. for $C_{20}H_{20}ClO_3^+$ 343.1095; Found: 343.1095.

1-(4-bromophenyl)-3-phenylprop-2-yn-1-yl tert-butyl carbonate (1ai)

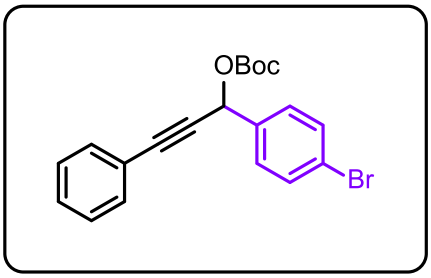

Following the **general procedure B** on 3 mmol scale, white solid, m.p. 86-88 °C, yield: 85% (987.2 mg, 2 steps), column chromatography (silica gel, PE: EA = 100:1, v/v).

^{1}H NMR (500 MHz, Chloroform-*d*) δ 7.55 – 7.52 (m, 2H), 7.51 – 7.49 (m, 2H), 7.48 – 7.46 (m, 2H), 7.35 – 7.29 (m, 3H), 6.45 (s, 1H), 1.51 (s, 9H).

^{13}C NMR (126 MHz, Chloroform-*d*) δ 152.4, 136.1, 131.90, 131.88, 129.5, 129.0, 128.4, 123.2, 121.9, 88.0, 84.8, 83.3, 68.4, 27.8.

HRMS (ESI) m/z: $[M+H]^+$ Calcd. for $C_{20}H_{20}BrO_3^+$ 387.0590; Found: 387.0589.

tert-butyl (1-(4-(tert-butyl)phenyl)-3-phenylprop-2-yn-1-yl) carbonate (1aj)

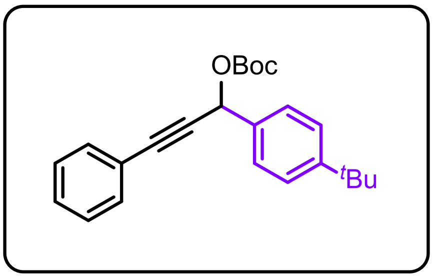

Following the **general procedure B** on 3 mmol scale, white solid, m.p. 112-114 °C, yield: 48% (524.6 mg, 2 steps), column chromatography (silica gel, PE: EA = 100:1, v/v).

^{1}H NMR (500 MHz, Chloroform-*d*) δ 7.58 – 7.55 (m, 2H), 7.50 – 7.47 (m, 2H), 7.45 – 7.42 (m, 2H), 7.34 – 7.29 (m, 3H), 6.49 (s, 1H), 1.51 (s, 9H), 1.33 (s, 9H).

^{13}C NMR (126 MHz, Chloroform-*d*) δ 152.7, 152.2, 133.9, 131.9, 128.7, 128.3, 127.7, 125.7, 122.3, 87.4, 85.6, 82.9, 69.0, 34.7, 31.3, 27.8.

HRMS (ESI) m/z: $[M+H]^+$ Calcd. for $C_{24}H_{29}O_3$+ 365.2111; Found: 365.2111.

tert-butyl (3-phenyl-1-(4-(trifluoromethoxy)phenyl)prop-2-yn-1-yl) carbonate (1ak)

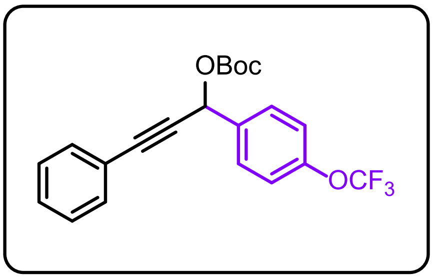

Following the **general procedure B** on 3 mmol scale, white solid, m.p. 67-69 °C, yield: 77% (898.9 mg, 2 steps), column chromatography (silica gel, PE: EA = 100:1, v/v).

^{1}H NMR (500 MHz, Chloroform-*d*) δ 7.68 – 7.65 (m, 2H), 7.49 – 7.46 (m, 2H), 7.35 – 7.30 (m, 3H), 7.27 – 7.24 (m, 2H), 6.49 (s, 1H), 1.51 (s, 9H).

^{19}F NMR (471 MHz, Chloroform-*d*) δ -57.8.

^{13}C NMR (126 MHz, Chloroform-*d*) δ 152.5, 149.6 (d, J = 1.9 Hz), 135.7, 131.9, 129.5, 129.0, 128.3, 121.9, 121.1, 120.4 (q, J = 257.5 Hz), 88.1, 84.8, 83.4, 68.2, 27.8.

HRMS (ESI) m/z: $[M+H]^+$ Calcd. for $C_{21}H_{20}F_3O_4^+$ 393.1308; Found: 393.1305.

tert-butyl (3-phenyl-1-(4-(trifluoromethyl)phenyl)prop-2-yn-1-yl) carbonate (1al)

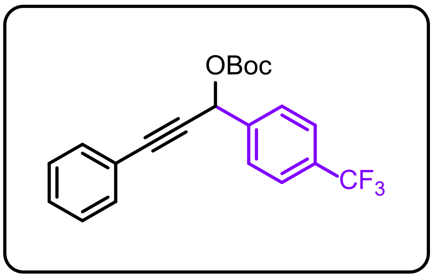

Following the **general procedure B** on 3 mmol scale, white solid, m.p. 101-103 °C, yield: 50% (567.3 mg, 2 steps), column chromatography (silica gel, PE: EA = 100:1, v/v).

^{1}H NMR (500 MHz, Chloroform-*d*) δ 7.74 (d, J = 8.2 Hz, 2H), 7.67 (d, J = 8.2 Hz, 2H), 7.49 – 7.46 (m, 2H), 7.36 – 7.30 (m, 3H), 6.53 (s, 1H), 1.51 (s, 9H).

^{19}F NMR (471 MHz, Chloroform-*d*) δ -62.7.

^{13}C NMR (126 MHz, Chloroform-*d*) δ 152.4, 140.9, 131.9, 131.1 (q, J = 32.5 Hz), 129.1, 128.4, 128.0, 125.7 (q, J = 3.7 Hz), 123.9 (q, J = 272.3 Hz), 121.8, 88.3, 84.5, 83.5, 68.2, 27.8.

HRMS (ESI) m/z: $[M+H]^+$ Calcd. for $C_{21}H_{20}F_3O_3^+$ 377.1359; Found: 377.1360.

1-([1,1'-biphenyl]-4-yl)-3-phenylprop-2-yn-1-yl tert-butyl carbonate (1am)

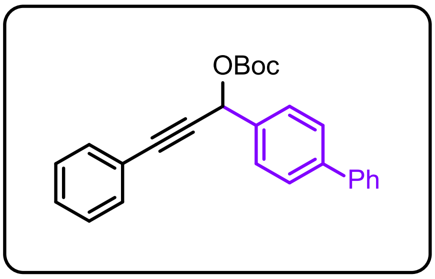

Following the **general procedure B** on 3 mmol scale, yellow solid, m.p. 126-128 °C, yield: 52% (594.4 mg, 2 steps), column chromatography (silica gel, PE: EA = 80:1, v/v).

^{1}H NMR (500 MHz, Chloroform-*d*) δ 7.74 – 7.70 (m, 2H), 7.65 (d, J = 8.3 Hz, 2H), 7.62 (dd, J = 7.9, 1.4 Hz, 2H), 7.54 – 7.50 (m, 2H), 7.47 (t, J = 7.7 Hz, 2H), 7.40 – 7.31 (m, 4H), 6.57 (s, 1H), 1.54 (s, 9H).

^{13}C NMR (126 MHz, Chloroform-*d*) δ 152.6, 142.1, 140.6, 136.0, 131.9, 128.87, 128.85, 128.3, 127.6, 127.5, 127.2, 122.2 87.8, 85.4, 83.1, 68.9, 27.8.

HRMS (ESI) m/z: $[M+H]^+$ Calcd. for $C_{26}H_{25}O_3^+$ 385.1798; Found: 385.1798.

tert-butyl (1-(naphthalen-1-yl)-3-phenylprop-2-yn-1-yl) carbonate (1an)

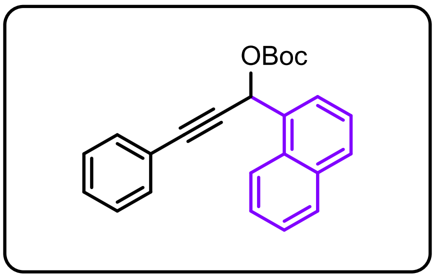

Following the **general procedure B** on 3 mmol scale, yellow oil, yield: 52% (556.6 mg, 2 steps), column chromatography (silica gel, PE: EA = 80:1, v/v).

^{1}H NMR (500 MHz, Chloroform-*d*) δ 8.36 (d, J = 8.5 Hz, 1H), 7.93 – 7.88 (m, 3H), 7.61 (ddd, J = 8.5, 6.8, 1.5 Hz, 1H), 7.56 – 7.49 (m, 2H), 7.49 – 7.46 (m, 2H), 7.34 – 7.28 (m, 3H), 7.16 (s, 1H), 1.53 (s, 9H).

^{13}C NMR (126 MHz, Chloroform-*d*) δ 152.7, 134.1, 132.4, 131.9, 130.7, 130.0, 128.84, 128.80, 128.3, 126.7, 126.6, 126.0, 125.3, 123.9, 122.3, 88.1, 85.5, 83.1, 67.5, 27.8.

HRMS (ESI) m/z: $[M+H]^+$ Calcd. for $C_{24}H_{23}O_3^+$ 359.1642; Found: 359.1642.

tert-butyl (1,5-diphenylpent-1-yn-3-yl) carbonate (1ao)

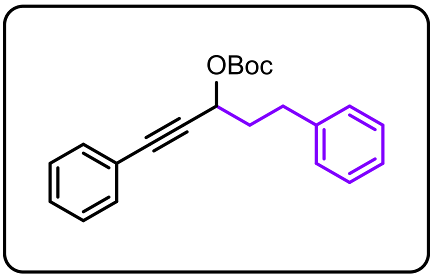

Following the **general procedure B** on 3 mmol scale, yellow oil, yield: 72% (727.2 mg, 2 steps), column chromatography (silica gel, PE: EA = 80:1, v/v).

^{1}H NMR (500 MHz, Chloroform-*d*) δ 7.48 – 7.44 (m, 2H), 7.35 – 7.29 (m, 5H), 7.25 – 7.19 (m, 3H), 5.42 (t, J = 6.6 Hz, 1H), 2.88 (t, J = 8.0 Hz, 2H), 2.29 – 2.17 (m, 2H), 1.53 (s, 9H).

^{13}C NMR (126 MHz, Chloroform-*d*) δ 152.7, 140.8, 131.9, 128.7, 128.6, 128.5, 128.3, 126.2, 122.3, 86.2, 86.0, 82.8, 67.0, 36.6, 31.4, 27.8.

HRMS (ESI) m/z: $[M+H]^+$ Calcd. for $C_{22}H_{25}O_3^+$ 337.1798; Found: 337.1791.

tert-butyl (4-methyl-1-phenylpent-1-yn-3-yl) carbonate (1ap)

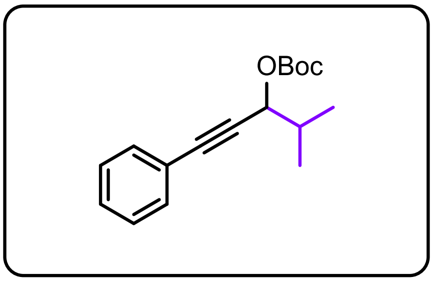

Following the **general procedure B** on 3 mmol scale, colorless oil, yield: 51% (419.5 mg, 2 steps), column chromatography (silica gel, PE: EA = 100:1, v/v).

^{1}H NMR (500 MHz, Chloroform-*d*) δ 7.46 – 7.42 (m, 2H), 7.32 – 7.27 (m, 3H), 5.27 (d, J = 5.6 Hz, 1H), 2.13 (pd, J = 6.8, 5.6 Hz, 1H), 1.51 (s, 9H), 1.11 (d, J = 6.7 Hz, 3H), 1.07 (d, J = 6.8 Hz, 3H).

^{13}C NMR (126 MHz, Chloroform-*d*) δ 153.0, 131.9, 128.5, 128.2, 122.5, 86.4, 85.0, 82.5, 72.6, 32.8, 27.8, 18.4, 17.6.

HRMS (ESI) m/z: $[M+H]^+$ Calcd. for $C_{17}H_{23}O_3^+$ 275.1642; Found: 275.1641.

4. Optimization studies

Supplementary Table 1. The effects of ligand[a]

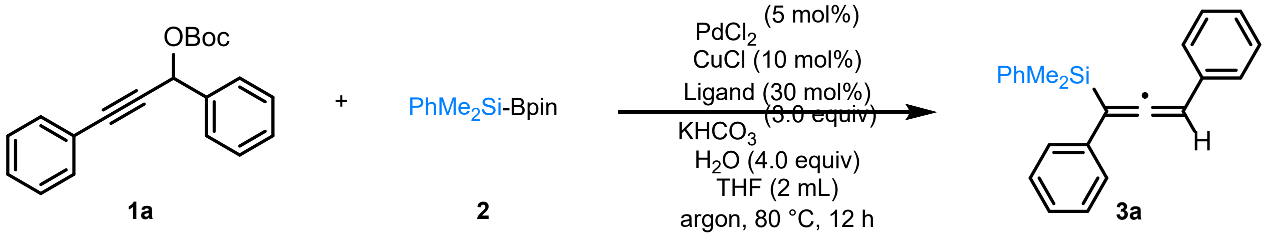

Entry	Ligand	Yield[b] (%)
1	X-Phos	trace
2	S-Phos	trace
3	RuPhos	trace
4	P(p-tol)3	43
5	TDMPP	0
**6**	TFPP	**80**

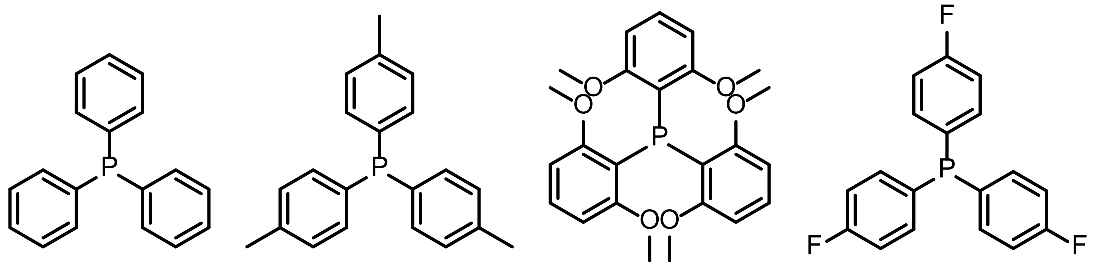

PPh$_3$ P(p-tol)3 TDMPP TFPP

[a] Reaction condition: **1a** (0.2 mmol, 1.0 equiv), **2** (0.4 mmol, 2.0 equiv), $PdCl_2$ (5 mol%), CuCl (10 mol%), ligand (30 mol%) and $KHCO_3$ (3.0 equiv) in THF (2 mL) at 80 ºC for 12 h under argon, 25 mL Schlenk tube. [b] Isolated yield.

Supplementary Table 2. The effects of Pd catalyst[a]

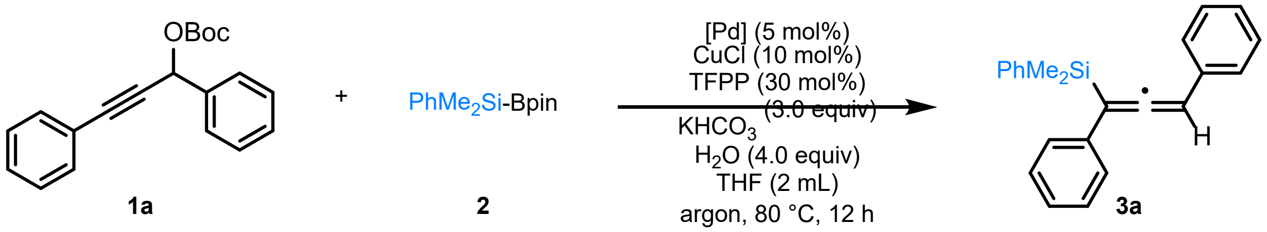

Entry	[Pd]	Yield[b] (%)
**1**	**PdI_2**	**85**
2	$[Pd(allyl)Cl]_2$	10
3	$Pd(dppf)Cl_2$	trace
4	$Pd(dppf)Cl_2$•CH_2Cl_2	0
5	$Pd(PCy_3)_2Cl_2$	58

[a] Reaction condition: **1a** (0.2 mmol, 1.0 equiv), **2** (0.4 mmol, 2.0 equiv), [Pd] (5 mol%), CuCl (10 mol%), **TFPP** (30 mol%), $KHCO_3$ (3.0 equiv) and H_2O (4.0 equiv) in THF (2 mL) at 80 ºC for 12 h under argon, 25 mL Schlenk tube. [b] Isolated yield.

Supplementary Table 3. The effects of Cu salt[a]

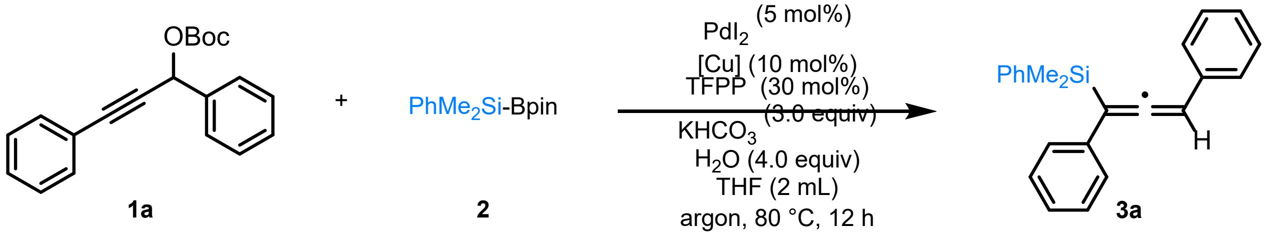

Entry	[Cu]	Yield[b] (%)
1	IMesCuCl	82
2	IPrCuCl	0
3	CuI	72
4	$Cu(OTf)_2$	trace

[a] Reaction condition: **1a** (0.2 mmol, 1.0 equiv), **2** (0.4 mmol, 2.0 equiv), PdI_2 (5 mol%), [Cu] (10 mol%), **TFPP** (30 mol%), $KHCO_3$ (3.0 equiv) and H_2O (4.0 equiv) in THF (2 mL) at 80 °C for 12 h under argon, 25 mL Schlenk tube. [b] Isolated yield.

Supplementary Table 4. The effects of base[a]

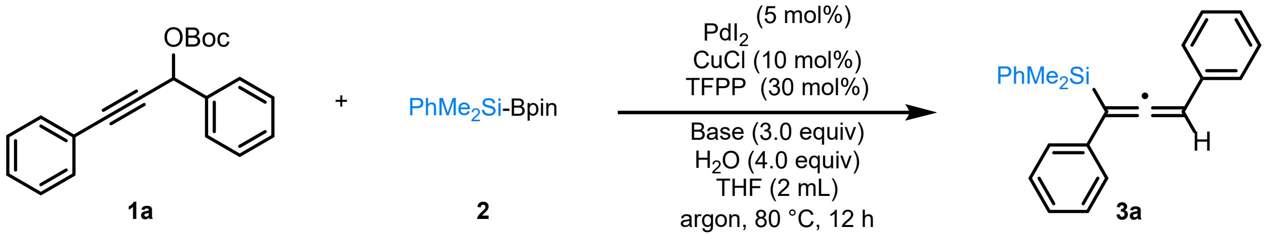

Entry	Base	Yield[b] (%)
**1**	**$NaHCO_3$**	**92**
2	Cs_2CO_3	0
3	KO^{t}Bu	0
4	Et_3N	68

[a] Reaction condition: **1a** (0.2 mmol, 1.0 equiv), **2** (0.4 mmol, 2.0 equiv), PdI_2 (5 mol%), CuCl (10 mol%), **TFPP** (30 mol%), base (3.0 equiv) and H_2O (4.0 equiv) in THF (2 mL) at 80 ºC for 12 h under argon, 25 mL Schlenk tube. [b] Isolated yield.

Supplementary Table 5. The effects of equivalent of base[a]

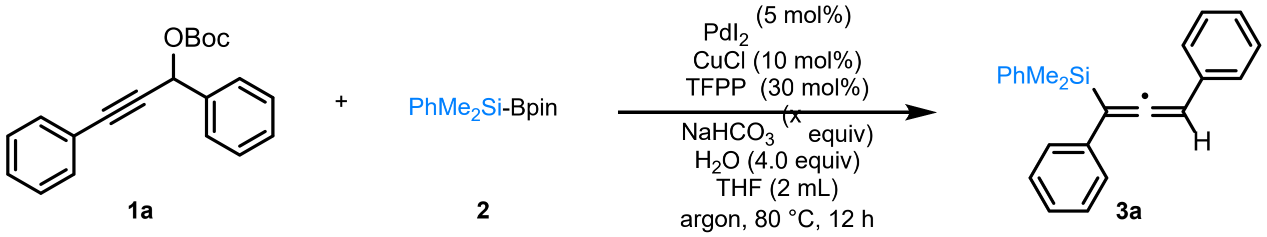

Entry	x (equiv)	Yield[b] (%)
**1**	**2.0**	**92**
2	3.0	80
3	4.0	91

[a] Reaction condition: **1a** (0.2 mmol, 1.0 equiv), **2** (0.4 mmol, 2.0 equiv), PdI_2 (5 mol%), CuCl (10 mol%), **TFPP** (30 mol%), $NaHCO_3$ (x equiv) and H_2O (4.0 equiv) in THF (2 mL) at 80 ºC for 12 h under argon, 25 mL Schlenk tube. [b] Isolated yield.

Supplementary Table 6. The effects of H_2O[a]

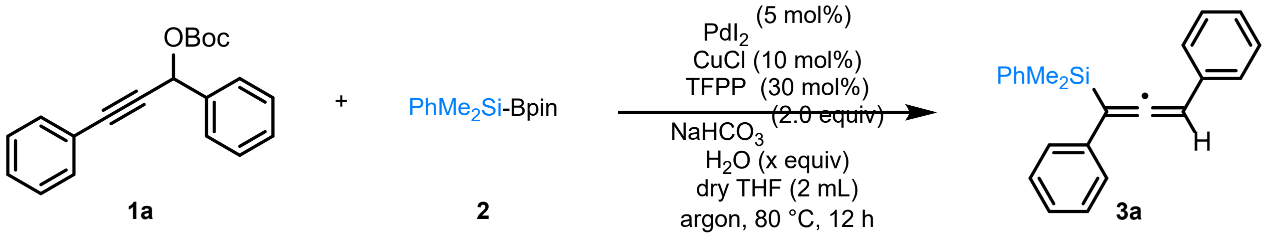

Entry	x (equiv)	Yield[b] (%)
1	0	67
2	3.0	89
**3**	**4.0**	**94**
4	5.0	87

[a] Reaction condition: **1a** (0.2 mmol, 1.0 equiv), **2** (0.4 mmol, 2.0 equiv), PdI_2 (5 mol%), CuCl (10 mol%), **TFPP** (30 mol%), $NaHCO_3$ (2.0 equiv) and H_2O (x equiv) in dry THF (2 mL) at 80 ºC for 12 h under argon, 25 mL Schlenk tube. [b] Isolated yield.

Supplementary Table 7. The effects of temperature[a]

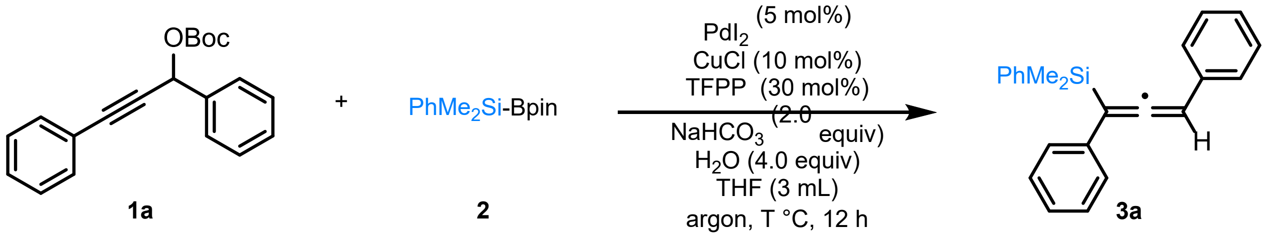

Entry	T (°C)	Yield[b] (%)
**1**	**70**	**98**
2	90	88
3	100	83

[a] Reaction condition: **1a** (0.2 mmol, 1.0 equiv), **2** (0.4 mmol, 2.0 equiv), PdI_2 (5 mol%), CuCl (10 mol%),TFPP (30 mol%), $NaHCO_3$ (2.0 equiv) and H_2O (4.0 equiv) in THF (3 mL) at T ºC for 12 h under argon, 25 mL Schlenk tube. [b] Isolated yield.

Supplementary Table 8. The effects of leaving group[a]

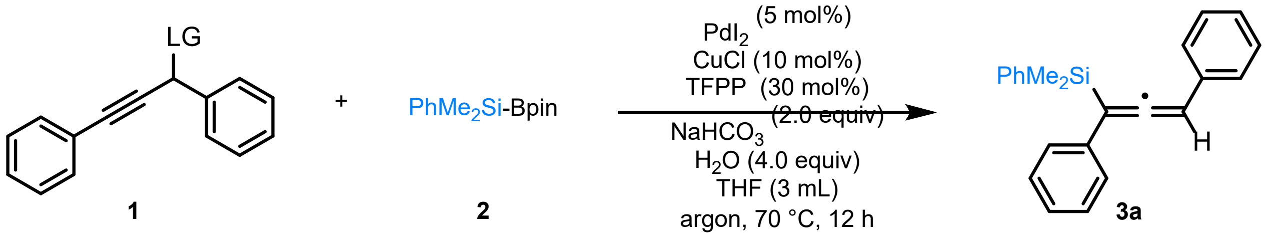

Entry	LG	Yield[b] (%)
**1**	**OBoc**	**98**
2	OAc	65
3	OBz	69
4	OPiv	39

[a] Reaction condition: **1** (0.2 mmol, 1.0 equiv), **2** (0.4 mmol, 2.0 equiv), PdI_2 (5 mol%), CuCl (10 mol%), **TFPP** (30 mol%), $NaHCO_3$ (2.0 equiv) and H_2O (4.0 equiv) in THF (3 mL) at 70 ºC for 12 h under argon, 25 mL Schlenk tube. [b] Isolated yield.

5.1. General procedure C for the synthesis of products 3

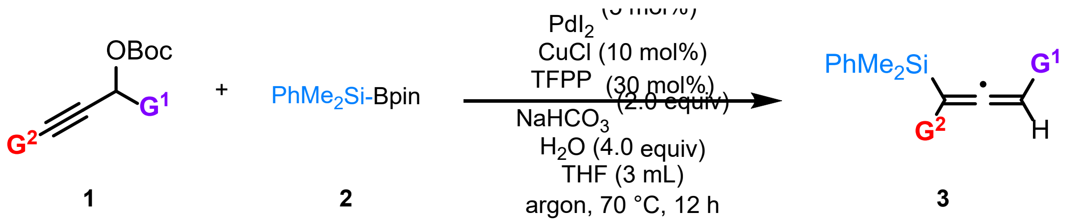

In air, PdI_2 (3.6 mg, 0.01 mmol, 5 mol%), CuCl (2.0 mg, 0.02 mmol, 10 mol%), **L4** (19.0 mg, 0.06 mmol, 30 mol%), $NaHCO_3$ (33.6 mg, 0.4 mmol, 2.0 equiv) were weighed into a 25 mL Schlenk tube equipped with a magnetic stirring bar. The tube was evacuated and filled with argon (three cycles). Then THF (2 mL), 0.2 mmol **1** (1.0 equiv) in 1 mL THF, 0.4 mmol **2** (2.0 equiv), H_2O (15 μL, 0.8 mmol, 4.0 equiv) were added to the tube subsequently under argon atmosphere. The resulting reaction mixture was stirred vigorously at 70 ºC for 12 h. Upon completion of the reaction, the solvent was evaporated under reduced pressure and the residue was purified by flash column chromatography (silica gel, PE or PE: EA=100:1, v/v) to afford the pure desired products **3**.

5.2. Large scale experiment of synthesis of products 3a

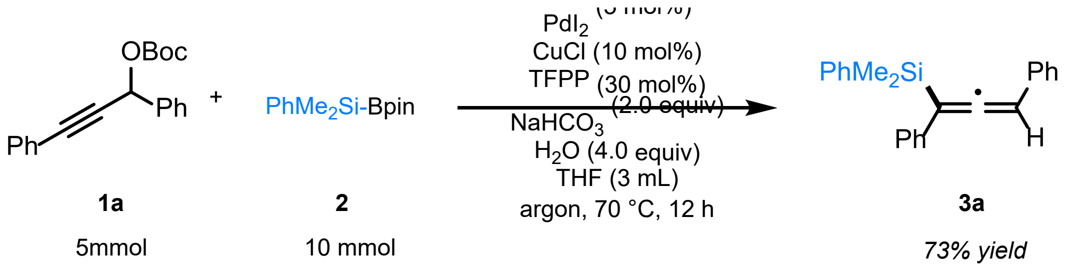

6. General procedure D for the synthesis of products 4

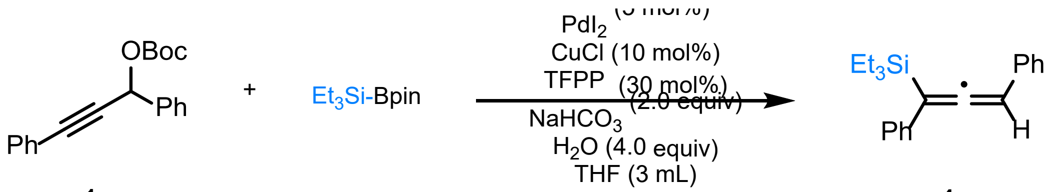

In air, PdI_2 (3.6 mg, 0.01 mmol, 5 mol%), CuCl (2.0 mg, 0.02 mmol, 10 mol%), **L4** (19.0 mg, 0.06 mmol, 30 mol%), $NaHCO_3$ (33.6 mg, 0.4 mmol, 2.0 equiv) were weighed into a 25 mL Schlenk tube equipped with a magnetic stirring bar. The tube was evacuated and filled with argon (three cycles). Then THF (2 mL), 0.2 mmol **1a** (1.0 equiv) in 1 mL THF, 0.4 mmol Et_3Si-Bpin (2.0 equiv), H_2O (15 μL, 0.8 mmol, 4.0 equiv) were added to the tube subsequently under argon atmosphere. The resulting reaction mixture was stirred vigorously at 70 ºC for 12 h. Upon completion of the reaction, the solvent was evaporated under reduced pressure and the residue was purified by flash column chromatography (silica gel, PE) to afford the pure desired products **4** in 54% yield (32.9 mg) as a yellow oil.

7. Characterization of products

(1,3-diphenylpropa-1,2-dien-1-yl)dimethyl(phenyl)silane (3a)

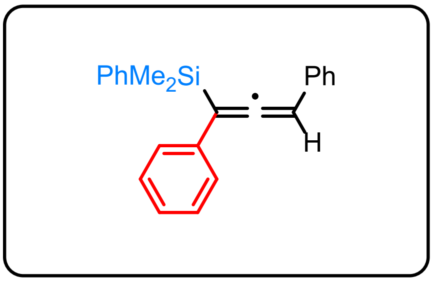

Following the **general procedure C** on 0.2 mmol scale, yellow oil, yield: 98% (63.9 mg), column chromatography (silica gel, PE: EA = 100:0, v/v).

^{1}H NMR (500 MHz, Chloroform-*d*) δ 7.74 – 7.69 (m, 2H), 7.45 – 7.34 (m, 9H), 7.30 – 7.24 (m, 3H), 7.23 – 7.19 (m, 1H), 6.38 (s, 1H), 0.59 (d, J = 10.5 Hz, 6H).

^{13}C NMR (126 MHz, Chloroform-*d*) δ 211.0, 138.1, 136.2, 134.6, 134.0, 129.4, 128.8, 128.6, 128.13, 128.07, 126.74, 126.65, 126.4, 103.2, 92.0, -1.6.

HRMS (ESI) m/z: $[M+H]^+$ Calcd. for $C_{23}H_{23}Si^+$ 327.1564; Found: 327.1564.

dimethyl(phenyl)(3-phenyl-1-(p-tolyl)propa-1,2-dien-1-yl)silane (3b)

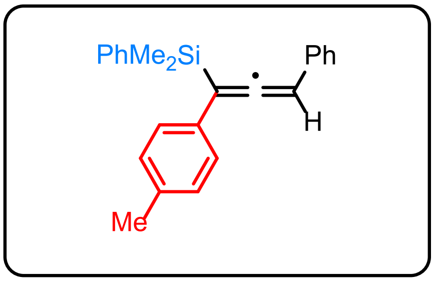

Following the **general procedure C** on 0.2 mmol scale, yellow oil, yield: 80% (54.4 mg), column chromatography (silica gel, PE: EA = 100:0, v/v).

^{1}H NMR (500 MHz, Chloroform-*d*) δ 7.71 – 7.64 (m, 2H), 7.40 (dd, J = 5.4, 1.8 Hz, 3H), 7.37 – 7.31 (m, 4H), 7.21 (tt, J = 6.5, 1.7 Hz, 3H), 7.05 (d, J = 7.8 Hz, 2H), 6.33 (s, 1H), 2.31 (s, 3H), 0.53 (d, J = 10.6 Hz, 6H).

^{13}C NMR (126 MHz, Chloroform-*d*) δ 210.7, 138.2 136.5, 134.7, 134.0, 132.9, 129.31, 129.28, 128.8, 127.97, 128.00, 126.5, 126.3, 102.8, 92.0, 21.1, -1.56, -1.59.

HRMS (ESI) m/z: $[M+H]^+$ Calcd. for $C_{24}H_{25}Si^+$ 341.1720; Found: 341.1720.

(1-(4-isopropylphenyl)-3-phenylpropa-1,2-dien-1-yl)dimethyl(phenyl)silane (3c)

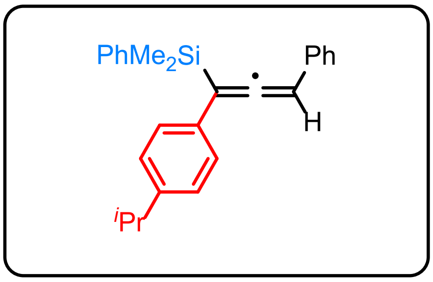

Following the **general procedure C** on 0.2 mmol scale, yellow oil, yield: 53% (38.9 mg), column chromatography (silica gel, PE: EA = 100:0, v/v).

^{1}H NMR (500 MHz, Chloroform-*d*) δ 7.71 – 7.66 (m, 2H), 7.42 – 7.38 (m, 3H), 7.37 – 7.31 (m, 4H), 7.26 – 7.23 (m, 2H), 7.21 (ddt, J = 8.7, 6.4, 1.8 Hz, 1H), 7.12 – 7.09 (m, 2H), 6.34 (s, 1H), 2.86 (p, J = 6.9 Hz, 1H), 1.23 (d, J = 6.9 Hz, 6H), 0.54 (d, J = 13.4 Hz, 6H).

^{13}C NMR (126 MHz, Chloroform-*d*) δ 210.9, 147.4, 138.3, 134.7, 134.0, 133.2, 129.3, 128.8, 128.0, 126.7, 126.5, 126.3, 102.8, 92.1, 33.8, 24.0, -1.5.

HRMS (ESI) m/z: $[M+H]^+$ Calcd. for $C_{26}H_{29}Si^+$ 369.2033; Found: 369.2031.

(1-(4-butylphenyl)-3-phenylpropa-1,2-dien-1-yl)dimethyl(phenyl)silane (3d)

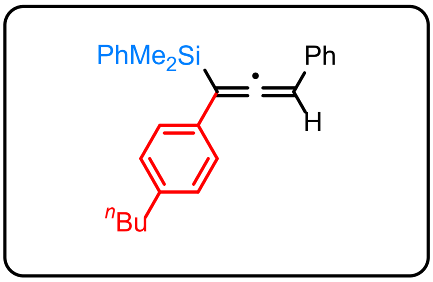

Following the **general procedure C** on 0.2 mmol scale, yellow oil, yield: 62% (47.6 mg), column chromatography (silica gel, PE: EA = 100:0, v/v).

^{1}H NMR (500 MHz, Chloroform-*d*) δ 7.70 – 7.66 (m, 2H), 7.42 – 7.38 (m, 3H), 7.38 – 7.34 (m, 3H), 7.34 – 7.32 (m, 1H), 7.24 – 7.19 (m, 3H), 7.08 – 7.04 (m, 2H), 6.33 (s, 1H), 2.59 – 2.54 (m, 2H), 1.60 – 1.54 (m, 2H), 1.35 (dq, J = 14.7, 7.4 Hz, 2H), 0.93 (t, J = 7.3 Hz, 3H), 0.53 (d, J = 12.2 Hz, 6H).

^{13}C NMR (126 MHz, Chloroform-*d*) δ 210.8, 141.5, 134.7, 134.0, 133.1, 129.3, 128.8, 128.6, 128.01, 127.95, 126.5, 126.3, 102.8, 92.0, 35.3, 33.6, 22.4, 14.0, -1.5.

HRMS (ESI) m/z: $[M+H]^+$ Calcd. for $C_{27}H_{31}Si^+$ 383.2190; Found: 383.2191.

(1-([1,1'-biphenyl]-4-yl)-3-phenylpropa-1,2-dien-1-yl)dimethyl(phenyl)silane (3e)

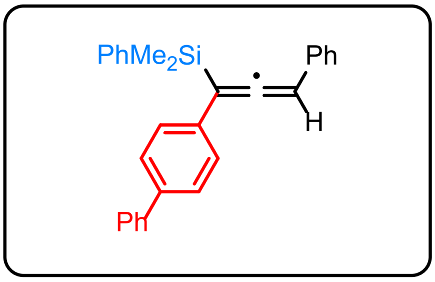

Following the **general procedure C** on 0.2 mmol scale, yellow oil, yield: 97% (77.8 mg), column chromatography (silica gel, PE: EA = 100:0, v/v).

^{1}H NMR (500 MHz, Chloroform-*d*) δ 7.81 – 7.75 (m, 2H), 7.65 – 7.61 (m, 2H), 7.57 – 7.53 (m, 2H), 7.51 – 7.37 (m, 12H), 7.32 – 7.27 (m, 1H), 6.45 (s, 1H), 0.64 (d, J = 11.5 Hz, 6H).

^{13}C NMR (126 MHz, Chloroform-*d*) δ 211.3, 140.8, 139.6, 138.1, 135.1, 134.6, 134.1, 129.5, 128.92, 128.86, 128.6, 128.2, 127.4, 127.3, 127.0, 126.8, 126.5, 102.9, 92.3, -1.45, -1.48.

HRMS (ESI) m/z: $[M+H]^+$ Calcd. for $C_{29}H_{27}Si^+$ 403.1877; Found: 403.1877.

(1-(4-fluorophenyl)-3-phenylpropa-1,2-dien-1-yl)dimethyl(phenyl)silane (3f)

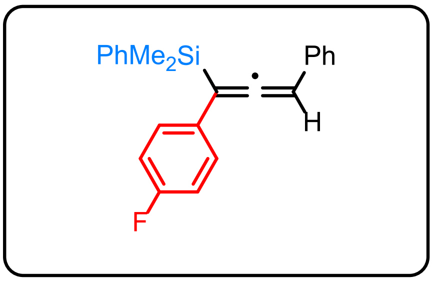

Following the **general procedure C** on 0.2 mmol scale, yellow oil, yield: 98% (67.5 mg), column chromatography (silica gel, PE: EA = 100:0, v/v).

^{1}H NMR (500 MHz, Chloroform-*d*) δ 7.69 – 7.63 (m, 2H), 7.43 – 7.38 (m, 3H), 7.37 – 7.33 (m, 4H), 7.27 – 7.21 (m, 3H), 6.96 – 6.89 (m, 2H), 6.35 (s, 1H), 0.53 (d, J = 10.0 Hz, 6H).

^{19}F NMR (471 MHz, Chloroform-*d*) δ -115.8.

^{13}C NMR (126 MHz, Chloroform-*d*) δ 210.7 (d, J = 1.8 Hz), 161.8 (d, J = 245.9 Hz), 137.8, 134.4, 134.0, 132.0 (d, J = 3.5 Hz), 129.53, 129.48 (d, J = 2.7 Hz), 128.9, 128.1, 126.7, 126.3, 115.4 (d, J = 21.6 Hz), 102.3, 92.2, -1.70, -1.73.

HRMS (ESI) m/z: $[M+H]^+$ Calcd. for $C_{23}H_{22}FSi^+$ 345.1469; Found: 345.1469.

(1-(4-chlorophenyl)-3-phenylpropa-1,2-dien-1-yl)dimethyl(phenyl)silane (3g)

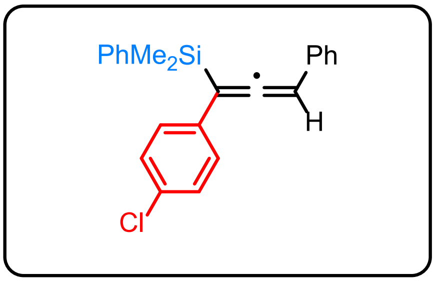

Following the **general procedure C** on 0.2 mmol scale, yellow oil, yield: 89% (63.9 mg), column chromatography (silica gel, PE: EA = 100:0, v/v).

^{1}H NMR (500 MHz, Chloroform-*d*) δ 7.68 – 7.61 (m, 2H), 7.43 – 7.38 (m, 3H), 7.34 (d, J = 5.1 Hz, 4H), 7.25 – 7.17 (m, 5H), 6.35 (s, 1H), 0.53 (d, J = 9.6 Hz, 6H).

^{13}C NMR (126 MHz, Chloroform-*d*) δ 211.0, 137.6, 134.6, 134.2, 133.9, 132.5, 129.5, 129.3, 128.9, 128.7, 128.1, 126.8, 126.4, 102.4, 92.3, -1.7, -1.8.

HRMS (ESI) m/z: $[M+H]^+$ Calcd. for $C_{23}H_{22}ClSi^+$ 361.1174; Found: 361.1173.

(1-(4-bromophenyl)-3-phenylpropa-1,2-dien-1-yl)dimethyl(phenyl)silane (3h)

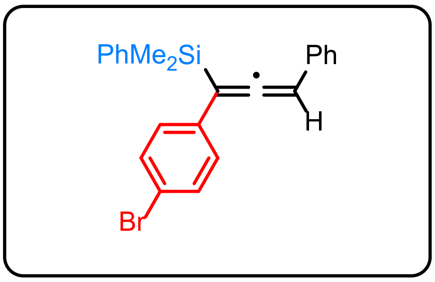

Following the **general procedure C** on 0.2 mmol scale, yellow oil, yield: 84% (67.7 mg), column chromatography (silica gel, PE: EA = 100:0, v/v).

^{1}H NMR (500 MHz, Chloroform-*d*) δ 7.68 – 7.61 (m, 2H), 7.41 (td, J = 5.1, 2.4 Hz, 3H), 7.34 (dq, J = 5.0, 3.0, 2.1 Hz, 6H), 7.23 (ddd, J = 5.5, 4.3, 2.7 Hz, 1H), 7.15 (dd, J = 8.4, 1.3 Hz, 2H), 6.34 (s, 1H), 0.53 (d, J = 9.5 Hz, 6H).

^{13}C NMR (126 MHz, Chloroform-*d*) δ 211.0, 137.6, 135.2, 134.1, 133.9, 131.6, 129.6, 129.5, 128.9, 128.1, 126.8, 126.4, 120.6, 102.4, 92.4, -1.7, -1.8.

HRMS (ESI) m/z: $[M+H]^+$ Calcd. For $C_{23}H_{22}BrSi^+$ 405.0669; Found: 405.0669.

(1-(2-fluorophenyl)-3-phenylpropa-1,2-dien-1-yl)dimethyl(phenyl)silane (3i)

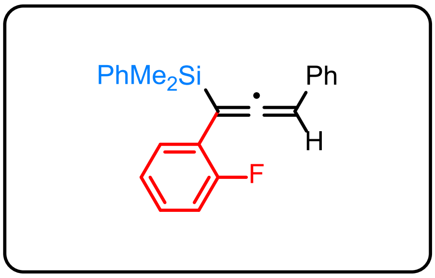

Following the **general procedure C** on 0.2 mmol scale, yellow oil, yield: 93% (63.9 mg), column chromatography (silica gel, PE: EA = 100:0, v/v).

^{1}H NMR (500 MHz, Chloroform-*d*) δ 7.60 (dd, J = 7.3, 1.9 Hz, 2H), 7.39 – 7.30 (m, 7H), 7.23 – 7.13 (m, 3H), 7.03 – 6.98 (m, 2H), 6.21 (s, 1H), 0.51 (s, 6H).

^{19}F NMR (471 MHz, Chloroform-*d*) δ -112.1.

^{13}C NMR (126 MHz, Chloroform-*d*) δ 210.8, 159.6 (d, J = 246.8 Hz), 137.7, 134.3, 133.9, 130.4 (d, J = 3.5 Hz), 129.3, 128.7, 128.2 (d, J = 8.1 Hz), 127.8, 126.6, 126.5, 124.5 (d, J = 15.3 Hz), 124.0 (d, J = 3.6 Hz), 115.7 (d, J = 22.3 Hz), 98.0, 90.7, -2.0 (d, J = 2.2 Hz), -2.2 (d, J = 2.2 Hz).

HRMS (ESI) m/z: $[M+H]^+$ Calcd. for $C_{23}H_{22}FSi^+$ 345.1469; Found: 345.1469.

(1-(2-chlorophenyl)-3-phenylpropa-1,2-dien-1-yl)dimethyl(phenyl)silane (3j)

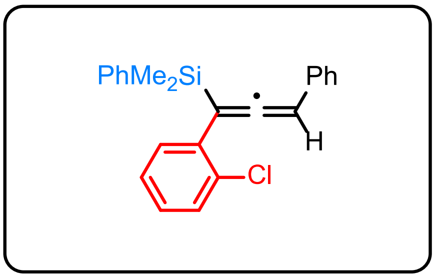

Following the **general procedure C** on 0.2 mmol scale, colorless oil, yield: 94% (67.5 mg), column chromatography (silica gel, PE: EA = 100:0, v/v).

^{1}H NMR (500 MHz, Chloroform-*d*) δ 7.61 – 7.55 (m, 2H), 7.41 – 7.29 (m, 8H), 7.21 – 7.17 (m, 1H), 7.14 – 7.10 (m, 2H), 7.05 – 7.01 (m, 1H), 6.14 (s, 1H), 0.50 (d, J = 2.4 Hz, 6H).

^{13}C NMR (126 MHz, Chloroform-*d*) δ 208.7, 137.5, 136.0, 134.3, 134.0, 132.7, 130.0, 129.7, 129.4, 128.6, 127.8, 127.6, 126.7, 126.6, 126.5, 101.5, 90.4, -2.3, -2.6.

HRMS (ESI) m/z: $[M+H]^+$ Calcd. for $C_{23}H_{22}ClSi^+$ 361.1174; Found: 361.1176.

(1-(2-bromophenyl)-3-phenylpropa-1,2-dien-1-yl)dimethyl(phenyl)silane (3k)

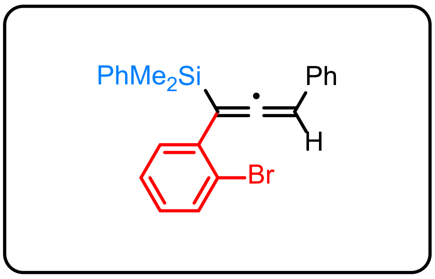

Following the **general procedure C** on 0.2 mmol scale, yellow oil, yield: 84% (67.7 mg), column chromatography (silica gel, PE: EA = 100:0, v/v).

^{1}H NMR (500 MHz, Chloroform-*d*) δ 7.57 (td, J = 7.6, 1.4 Hz, 3H), 7.40 – 7.34 (m, 5H), 7.33 – 7.29 (m, 2H), 7.21 – 7.15 (m, 2H), 7.07 – 7.00 (m, 2H), 6.14 (s, 1H), 0.51 (s, 6H).

^{13}C NMR (126 MHz, Chloroform-*d*) δ 208.4, 138.0, 137.4, 134.3, 134.0, 132.9, 129.9, 129.4, 128.6, 127.82, 127.79, 127.1, 126.8, 126.5, 123.1, 103.4, 90.6, -2.4, -2.6.

HRMS (ESI) m/z: $[M+H]^+$ Calcd. for $C_{23}H_{22}BrSi^+$ 405.0669; Found: 405.0667.

(1-(3-fluorophenyl)-3-phenylpropa-1,2-dien-1-yl)dimethyl(phenyl)silane (3l)

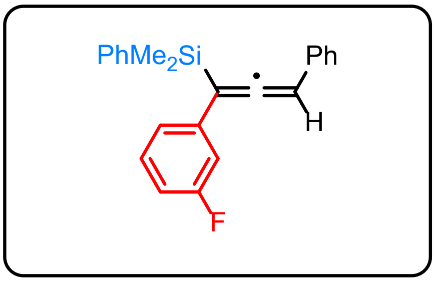

Following the **general procedure C** on 0.2 mmol scale, yellow oil, yield: 98% (67.5 mg), column chromatography (silica gel, PE: EA = 100:0, v/v).

^{1}H NMR (500 MHz, Chloroform-*d*) δ 7.67 – 7.63 (m, 2H), 7.42 – 7.37 (m, 3H), 7.37 – 7.32 (m, 4H), 7.25 – 7.21 (m, 1H), 7.17 (td, J = 8.0, 6.1 Hz, 1H), 7.04 (dt, J = 7.8, 1.3 Hz, 1H), 7.00 (dt, J = 10.3, 2.2 Hz, 1H), 6.86 (tdd, J = 8.4, 2.7, 0.9 Hz, 1H), 6.36 (s, 1H), 0.53 (d, J = 9.9 Hz, 6H).

^{19}F NMR (471 MHz, Chloroform-*d*) δ -113.1.

^{13}C NMR (126 MHz, Chloroform-*d*) δ 211.2, 162.9 (d, J = 245.6 Hz), 138.6 (d, J = 7.3 Hz), 137.6, 134.1, 133.9, 129.8 (d, J = 8.5 Hz), 129.5, 128.9, 128.1, 126.8, 126.4, 123.8 (d, J = 2.8 Hz), 114.8 (d, J = 21.9 Hz), 113.6 (d, J = 21.2 Hz), 102.7 (d, J = 2.3 Hz), 92.3, -1.7, -1.8.

HRMS (ESI) m/z: $[M+H]^+$ Calcd. for $C_{23}H_{22}FSi^+$ 345.1469; Found: 345.1470.

(1-(3-chlorophenyl)-3-phenylpropa-1,2-dien-1-yl)dimethyl(phenyl)silane (3m)

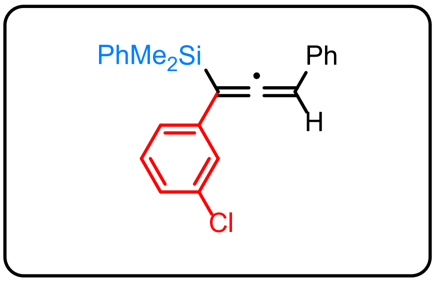

Following the **general procedure C** on 0.2 mmol scale, colorless oil, yield: 92% (66.1 mg), column chromatography (silica gel, PE: EA = 100:0, v/v).

^{1}H NMR (500 MHz, Chloroform-*d*) δ 7.67 – 7.62 (m, 2H), 7.43 – 7.38 (m, 3H), 7.37 – 7.33 (m, 4H), 7.30 (q, J = 1.4 Hz, 1H), 7.25 – 7.21 (m, 1H), 7.16 – 7.10 (m, 3H), 6.36 (s, 1H), 0.53 (d, J = 9.7 Hz, 6H).

^{13}C NMR (126 MHz, Chloroform-*d*) δ 211.1, 138.2, 137.5, 134.4, 134.0, 133.9, 129.6, 129.6, 128.9, 128.1, 128.0, 126.9, 126.8, 126.4, 126.1, 102.5, 92.4, -1.7, -1.8.

HRMS (ESI) m/z: $[M+H]^+$ Calcd. for $C_{23}H_{22}ClSi^+$ 361.1174; Found: 361.1175.

(1-(3-bromophenyl)-3-phenylpropa-1,2-dien-1-yl)dimethyl(phenyl)silane (3n)

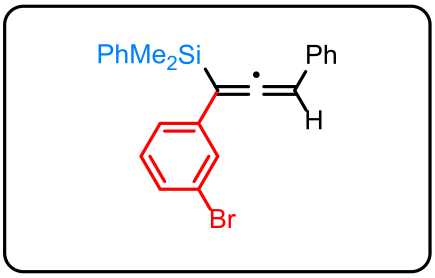

Following the **general procedure C** on 0.2 mmol scale, yellow oil, yield: 78% (62.9 mg), column chromatography (silica gel, PE: EA = 100:0, v/v).

^{1}H NMR (500 MHz, Chloroform-*d*) δ 7.67 – 7.62 (m, 2H), 7.46 (t, J = 1.8 Hz, 1H), 7.43 – 7.38 (m, 3H), 7.37 – 7.33 (m, 4H), 7.30 (ddd, J = 7.9, 2.0, 1.1 Hz, 1H), 7.25 – 7.22 (m, 1H), 7.15 (dt, J = 8.0, 1.3 Hz, 1H), 7.07 (t, J = 7.8 Hz, 1H), 6.36 (s, 1H), 0.53 (d, J = 9.9 Hz, 6H).

^{13}C NMR (126 MHz, Chloroform-*d*) δ 211.1, 138.5, 137.5, 134.02, 133.95, 130.9, 129.9, 129.7, 129.6, 128.9, 128.1, 126.9, 126.6, 126.4, 122.7, 102.4, 92.4, -1.7, -1.8.

HRMS (ESI) m/z: $[M+H]^+$ Calcd. for $C_{23}H_{22}BrSi^+$ 405.0669; Found: 405.0676.

ethyl 4-(1-(dimethyl(phenyl)silyl)-3-phenylpropa-1,2-dien-1-yl)benzoate (3o)

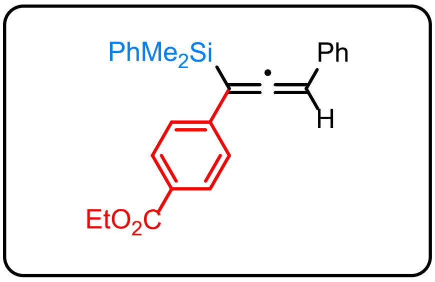

Following the **general procedure C** on 0.2 mmol scale, yellow oil, yield: 96% (76.8 mg), column chromatography (silica gel, PE: EA = 100:1, v/v).

^{1}H NMR (500 MHz, Chloroform-*d*) δ 7.94 (dd, J = 8.4, 1.7 Hz, 2H), 7.70 – 7.64 (m, 2H), 7.42 (qd, J = 4.0, 2.1 Hz, 3H), 7.37 (dd, J = 5.8, 2.2 Hz, 6H), 7.25 (ddd, J = 8.6, 5.2, 3.5 Hz, 1H), 6.41 (s, 1H), 4.37 (q, J = 7.1 Hz, 2H), 1.39 (t, J = 7.1 Hz, 3H), 0.57 (d, J = 8.5 Hz, 6H).

^{13}C NMR (126 MHz, Chloroform-*d*) δ 211.8, 166.5, 141.3, 137.5, 134.0, 129.8, 129.6, 128.9, 128.7, 128.2, 128.0, 126.9, 126.5, 103.1, 92.4, 60.9, 14.4, -1.67, -1.73.

HRMS (ESI) m/z: $[M+H]^+$ Calcd. for $C_{26}H_{27}O_2Si^+$ 399.1775; Found: 399.1773.

methyl 4-(1-(dimethyl(phenyl)silyl)-3-phenylpropa-1,2-dien-1-yl)benzoate (3p)

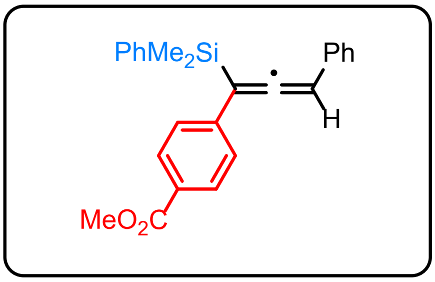

Following the **general procedure C** on 0.2 mmol scale, yellow oil, yield: 98% (75.3 mg), column chromatography (silica gel, PE: EA = 100:1, v/v).

^{1}H NMR (500 MHz, Chloroform-*d*) δ 7.94 – 7.90 (m, 2H), 7.68 – 7.64 (m, 2H), 7.43 – 7.39 (m, 3H), 7.38 – 7.34 (m, 6H), 7.26 – 7.22 (m, 1H), 6.40 (s, 1H), 3.89 (s, 3H), 0.55 (d, J = 8.7 Hz, 6H).

^{13}C NMR (126 MHz, Chloroform-*d*) δ 211.8, 167.0, 141.4, 137.5, 134.0, 133.9, 129.9, 129.6, 128.9, 128.3, 128.1, 128.0, 126.9, 126.4, 103.1, 92.4, 52.1, -1.68, -1.73.

HRMS (ESI) m/z: $[M+H]^+$ Calcd. for $C_{25}H_{25}O_2Si^+$ 385.1618; Found: 385.1618.

1-(4-(1-(dimethyl(phenyl)silyl)-3-phenylpropa-1,2-dien-1-yl)phenyl)ethan-1-one (3q)

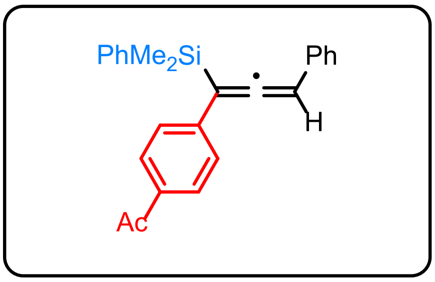

Following the **general procedure C** on 0.2 mmol scale, yellow oil, yield: 81% (58.3 mg), column chromatography (silica gel, PE: EA = 100:1, v/v).

^{1}H NMR (500 MHz, Chloroform-*d*) δ 7.84 – 7.81 (m, 2H), 7.67 – 7.64 (m, 2H), 7.43 – 7.39 (m, 3H), 7.38 (d, J = 2.0 Hz, 1H), 7.37 – 7.34 (m, 5H), 7.24 (dt, J = 8.7, 4.3 Hz, 1H), 6.40 (s, 1H), 2.55 (s, 3H), 0.55 (d, J = 8.9 Hz, 6H).

^{13}C NMR (126 MHz, Chloroform-*d*) δ 212.0, 197.6, 141.6, 137.5, 135.3, 133.94, 133.85, 129.6, 128.9, 128.7, 128.2, 128.1, 126.9, 126.4, 103.0, 92.4, 26.6, -1.7, -1.8.

HRMS (ESI) m/z: $[M+H]^+$ Calcd. for $C_{25}H_{25}OSi^+$ 369.1669; Found: 369.1669.

4-(1-(dimethyl(phenyl)silyl)-3-phenylpropa-1,2-dien-1-yl)benzonitrile (3r)

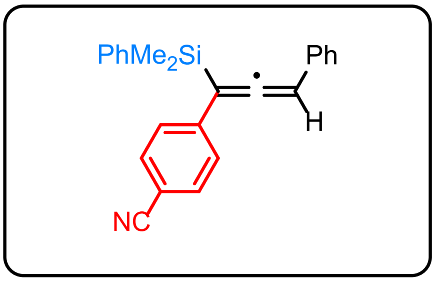

Following the **general procedure C** on 0.2 mmol scale, yellow oil, yield: 83% (58.3 mg), column chromatography (silica gel, PE: EA = 100:1, v/v).

^{1}H NMR (500 MHz, Chloroform-*d*) δ 7.64 – 7.60 (m, 2H), 7.50 – 7.47 (m, 2H), 7.43 – 7.38 (m, 3H), 7.36 – 7.31 (m, 6H), 7.26 – 7.22 (m, 1H), 6.41 (s, 1H), 0.53 (d, J = 9.2 Hz, 6H).

^{13}C NMR (126 MHz, Chloroform-*d*) δ 212.2, 141.6, 137.1, 133.9, 133.4, 132.3, 129.7, 129.0, 128.6, 128.2, 127.1, 126.5, 119.0, 110.1, 102.9, 92.8, -1.8, -1.9.

HRMS (ESI) m/z: $[M+H]^+$ Calcd. for $C_{24}H_{22}NSi^+$ 352.1516; Found: 352.1517.

3-(1-(dimethyl(phenyl)silyl)-3-phenylpropa-1,2-dien-1-yl)benzonitrile (3s)

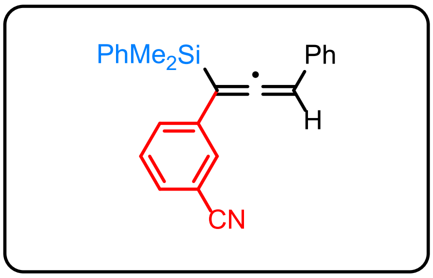

Following the **general procedure C** on 0.2 mmol scale, yellow oil, yield: 98% (68.5 mg), column chromatography (silica gel, PE: EA = 100:1, v/v).

^{1}H NMR (500 MHz, Chloroform-*d*) δ 7.67 – 7.62 (m, 2H), 7.56 (d, J = 1.9 Hz, 1H), 7.49 – 7.41 (m, 5H), 7.38 – 7.33 (m, 4H), 7.31 (t, J = 7.8 Hz, 1H), 7.28 – 7.24 (m, 1H), 6.42 (s, 1H), 0.56 (d, J = 9.4 Hz, 6H).

^{13}C NMR (126 MHz, Chloroform-*d*) δ 211.6, 138.0, 137.0, 133.9, 133.5, 132.3, 131.4, 130.1, 129.8, 129.3, 129.0, 128.3, 127.1, 126.5, 118.9, 112.7, 102.2, 92.8, -1.8, -1.9.

HRMS (ESI) m/z: $[M+H]^+$ Calcd. for $C_{24}H_{22}NSi^+$ 352.1516; Found: 352.1515.

dimethyl(phenyl)(3-phenyl-1-(4-(trifluoromethyl)phenyl)propa-1,2-dien-1-yl)silane (3t)

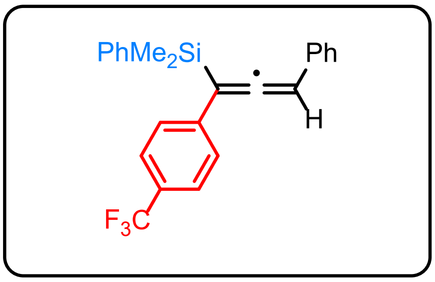

Following the **general procedure C** on 0.2 mmol scale, yellow oil, yield: 70% (55.2 mg), column chromatography (silica gel, PE: EA = 100:0, v/v).

^{1}H NMR (500 MHz, Chloroform-*d*) δ 7.67 – 7.62 (m, 2H), 7.47 (d, J = 8.2 Hz, 2H), 7.43 – 7.33 (m, 9H), 7.24 (tt, J = 6.2, 3.0 Hz, 1H), 6.39 (s, 1H), 0.53 (d, J = 9.8 Hz, 6H).

^{19}F NMR (471 MHz, Chloroform-*d*) δ -62.4.

^{13}C NMR (126 MHz, Chloroform-*d*) δ 211.7, 140.3, 137.4, 133.9, 133.8, 129.6, 128.9, 128.6 (q, J = 32.5 Hz), 128.19, 128.17, 127.0, 126.4, 125.4 (q, J = 3.8 Hz), 124.2 (q, J = 272.0 Hz), 102.7, 92.4, -1.78, -1.84.

HRMS (ESI) m/z: $[M+H]^+$ Calcd. for $C_{24}H_{22}F_3Si^+$ 395.1437; Found: 395.1436.

methyl 2-(1-(dimethyl(phenyl)silyl)-3-phenylpropa-1,2-dien-1-yl)benzoate (3u)

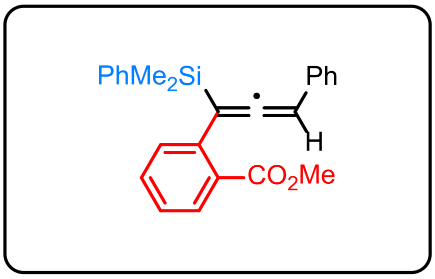

Following the **general procedure C** on 0.2 mmol scale, yellow oil, yield: 98% (75.3 mg), column chromatography (silica gel, PE: EA = 100:1, v/v).

^{1}H NMR (500 MHz, Chloroform-*d*) δ 7.83 (dd, J = 7.8, 1.4 Hz, 1H), 7.54 – 7.51 (m, 2H), 7.36 (dd, J = 7.4, 1.6 Hz, 2H), 7.35 – 7.31 (m, 4H), 7.29 (d, J = 7.3 Hz, 2H), 7.24 (dd, J = 7.6, 1.3 Hz, 1H), 7.18 – 7.14 (m, 1H), 7.08 (dd, J = 7.6, 1.3 Hz, 1H), 6.09 (s, 1H), 3.78 (s, 3H), 0.46 (d, J = 6.1 Hz, 6H).

^{13}C NMR (126 MHz, Chloroform-*d*) δ 206.7, 167.8, 138.6, 137.9, 134.9, 134.0, 131.8, 130.4, 130.1, 129.2, 128.6, 127.7, 126.6, 126.4, 126.4, 104.2, 90.3, 52.0, -2.5, -2.7.

HRMS (ESI) m/z: $[M+H]^+$ Calcd. for $C_{25}H_{25}O_2Si^+$ 385.1618; Found: 385.1618.

ethyl 2-(1-(dimethyl(phenyl)silyl)-3-phenylpropa-1,2-dien-1-yl)benzoate (3v)

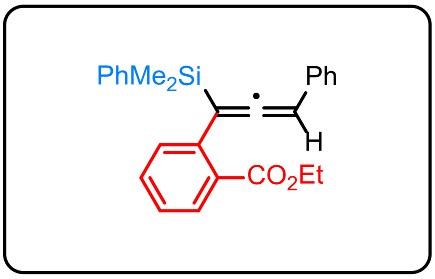

Following the **general procedure C** on 0.2 mmol scale, yellow oil, yield: 98% (78.1 mg), column chromatography (silica gel, PE: EA = 100:1, v/v).

^{1}H NMR (500 MHz, Chloroform-*d*) δ 7.85 (dd, J = 7.8, 1.5 Hz, 1H), 7.58 – 7.54 (m, 2H), 7.40 – 7.33 (m, 6H), 7.30 (dd, J = 8.6, 6.9 Hz, 2H), 7.27 – 7.24 (m, 1H), 7.20 – 7.16 (m, 1H), 7.08 (dd, J = 7.7, 1.4 Hz, 1H), 6.10 (s, 1H), 4.29 (q, J = 7.2 Hz, 2H), 1.36 (t, J = 7.2 Hz, 3H), 0.50 (d, J = 7.0 Hz, 6H).

^{13}C NMR (126 MHz, Chloroform-*d*) δ 206.8, 167.4, 138.5, 138.0, 135.0, 134.0, 131.6, 130.3, 130.0, 129.7, 129.2, 128.6, 127.8, 126.6, 126.4, 126.3, 104.1, 90.3, 60.9, 14.4, -2.5, -2.7.

HRMS (ESI) m/z: $[M+H]^+$ Calcd. for $C_{26}H_{27}O_2Si^+$ 399.1775; Found: 399.1773.

dimethyl(1-(4-(methylthio)phenyl)-3-phenylpropa-1,2-dien-1-yl)(phenyl)silane (3w)

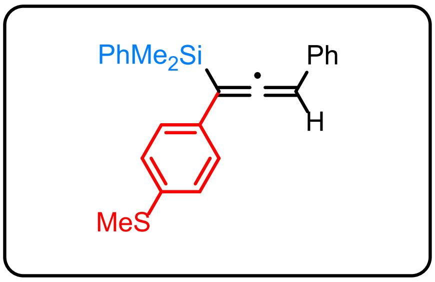

Following the **general procedure C** on 0.2 mmol scale, yellow oil, yield: 82% (61.1 mg), column chromatography (silica gel, PE: EA = 100:0, v/v).

^{1}H NMR (500 MHz, Chloroform-*d*) δ 7.66 – 7.62 (m, 2H), 7.40 – 7.36 (m, 3H), 7.32 (d, J = 4.7 Hz, 4H), 7.22 – 7.18 (m, 3H), 7.12 – 7.08 (m, 2H), 6.32 (s, 1H), 2.43 (s, 3H), 0.50 (d, J = 10.3 Hz, 6H).

^{13}C NMR (126 MHz, Chloroform-*d*) δ 210.9, 137.9, 136.7, 134.5, 134.0, 132.7, 129.4, 128.8, 128.5, 128.1, 126.8, 126.6, 126.3, 102.5, 92.2, 15.9, -1.6, -1.7.

HRMS (ESI) m/z: $[M+H]^+$ Calcd. For $C_{24}H_{25}SSi^+$ 373.1411; Found: 373.1411.

dimethyl(phenyl)(3-phenyl-1-(4-(trifluoromethoxy)phenyl)propa-1,2-dien-1-yl)silane (3x)

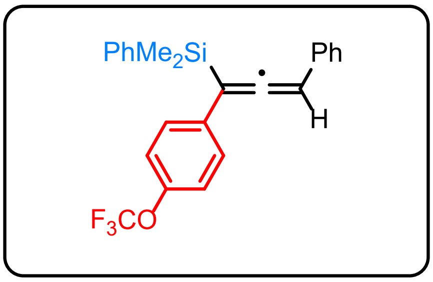

Following the **general procedure C** on 0.2 mmol scale, yellow oil, yield: 95% (65.9 mg), column chromatography (silica gel, PE: EA = 100:0, v/v).

^{1}H NMR (500 MHz, Chloroform-*d*) δ 7.69 (ddd, J = 6.1, 3.0, 1.4 Hz, 2H), 7.46 – 7.41 (m, 3H), 7.40 – 7.36 (m, 4H), 7.34 – 7.30 (m, 2H), 7.29 – 7.24 (m, 1H), 7.12 – 7.08 (m, 2H), 6.39 (s, 1H), 0.56 (d, J = 11.2 Hz, 6H).

^{19}F NMR (471 MHz, Chloroform-*d*) δ -57.8.

^{13}C NMR (126 MHz, Chloroform-*d*) δ 211.2, 148.0 (d, J = 2.2 Hz), 137.6, 135.0, 134.1, 134.0, 129.6, 129.3, 128.9, 128.2, 126.9, 126.4, 121.1, 120.5 (q, J = 257.1 Hz), 102.3, 92.4, -1.7, -1.8.

HRMS (ESI) m/z: $[M+H]^+$ Calcd. for $C_{24}H_{22}F_3OSi^+$ 411.1387; Found: 411.1387.

(1-(3-chloro-4-methylphenyl)-3-phenylpropa-1,2-dien-1-yl)dimethyl(phenyl)silane (3y)

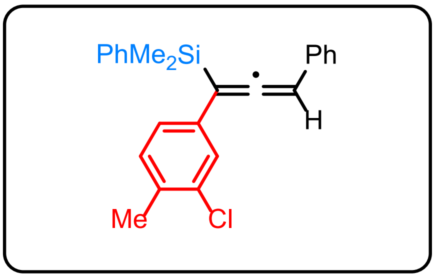

Following the **general procedure C** on 0.2 mmol scale, yellow oil, yield: 84% (62.8 mg), column chromatography (silica gel, PE: EA = 100:0, v/v).

^{1}H NMR (500 MHz, Chloroform-*d*) δ 7.70 – 7.65 (m, 2H), 7.44 – 7.39 (m, 3H), 7.36 (d, J = 4.3 Hz, 4H), 7.33 (d, J = 1.7 Hz, 1H), 7.26 – 7.22 (m, 1H), 7.10 – 7.04 (m, 2H), 6.36 (s, 1H), 2.33 (s, 3H), 0.55 (d, J = 9.4 Hz, 6H).

^{13}C NMR (126 MHz, Chloroform-*d*) δ 210.9, 137.7, 135.3, 134.5, 134.4, 134.3, 134.0, 130.9, 129.5, 128.9, 128.4, 128.1, 126.8, 126.4, 126.3, 102.2, 92.3, 19.7, -1.6, -1.7.

HRMS (ESI) m/z: $[M+H]^+$ Calcd. for $C_{24}H_{24}ClSi^+$ 375.1330; Found: 375.1331.

(1-(4-fluoro-3-methylphenyl)-3-phenylpropa-1,2-dien-1-yl)dimethyl(phenyl)silane (3z)

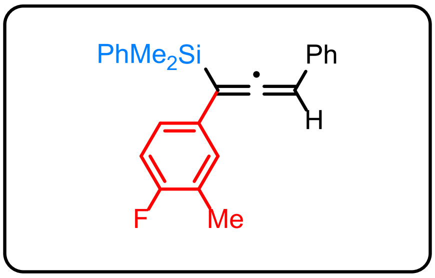

Following the **general procedure C** on 0.2 mmol scale, yellow oil, yield: 91% (65.5 mg), column chromatography (silica gel, PE: EA = 100:0, v/v).

^{1}H NMR (500 MHz, Chloroform-*d*) δ 7.69 – 7.64 (m, 2H), 7.41 (dt, J = 5.7, 3.3 Hz, 3H), 7.38 – 7.33 (m, 4H), 7.23 (tt, J = 5.1, 3.4 Hz, 1H), 7.12 (dd, J = 7.4, 2.4 Hz, 1H), 7.03 (ddd, J = 7.7, 4.9, 2.4 Hz, 1H), 6.86 (t, J = 8.9 Hz, 1H), 6.33 (s, 1H), 2.19 (d, J = 2.0 Hz, 3H), 0.53 (d, J = 10.2 Hz, 6H).

^{19}F NMR (471 MHz, Chloroform-*d*) δ -120.1.

^{13}C NMR (126 MHz, Chloroform-*d*) δ 210.5 (d, J = 1.8 Hz), 160.4 (d, J = 244.8 Hz), 137.9, 134.5, 134.0, 131.6 (d, J = 3.6 Hz), 131.0 (d, J = 5.0 Hz), 129.5, 128.8, 128.1, 126.8 (d, J = 8.0 Hz), 126.7, 126.4, 124.9 (d, J = 17.4 Hz), 115.0 (d, J = 22.6 Hz), 102.3, 92.0, 14.6 (d, J = 3.3 Hz), -1.6, -1.7.

HRMS (ESI) m/z: $[M+H]^+$ Calcd. for $C_{24}H_{24}FSi^+$ 359.1626; Found: 359.1623.

(1-(5-chloro-2-methoxyphenyl)-3-phenylpropa-1,2-dien-1-yl)dimethyl(phenyl)silane (3aa)

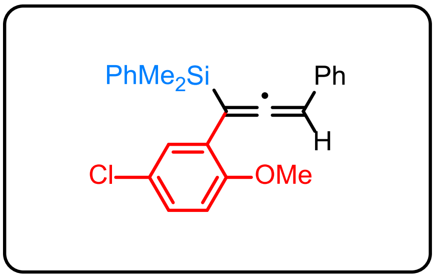

Following the **general procedure C** on 0.2 mmol scale, yellow oil, yield: 91% (71 mg), column chromatography (silica gel, PE: EA = 100:0, v/v).

^{1}H NMR (500 MHz, Chloroform-*d*) δ 7.59 – 7.54 (m, 2H), 7.38 – 7.31 (m, 7H), 7.21 (ddt, J = 8.5, 6.6, 1.7 Hz, 1H), 7.18 (t, J = 2.4 Hz, 1H), 7.13 (ddd, J = 8.7, 2.6, 1.3 Hz, 1H), 6.68 (d, J = 8.7 Hz, 1H), 6.21 (d, J = 1.8 Hz, 1H), 3.52 (s, 3H), 0.47 (dd, J = 4.5, 1.8 Hz, 6H).

^{13}C NMR (126 MHz, Chloroform-*d*) δ 210.4, 154.7, 138.5, 134.4, 133.6, 129.5, 128.9, 128.7, 127.8, 127.6, 127.5, 126.6, 126.5, 125.5, 111.7, 100.1, 90.9, 55.0, -1.6, -1.8.

HRMS (ESI) m/z: $[M+H]^+$ Calcd. for $C_{24}H_{24}ClOSi^+$ 391.1279; Found: 391.1280.

(1-(3,5-dichlorophenyl)-3-phenylpropa-1,2-dien-1-yl)dimethyl(phenyl)silane (3ab)

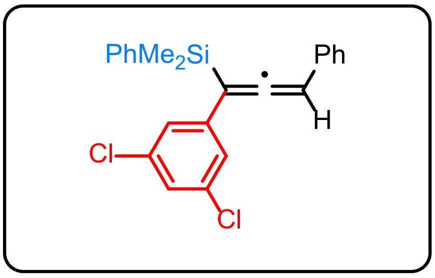

Following the **general procedure C** on 0.2 mmol scale, yellow oil, yield: 87% (68.5 mg), column chromatography (silica gel, PE: EA = 100:0, v/v).

^{1}H NMR (500 MHz, Chloroform-*d*) δ 7.66 – 7.61 (m, 2H), 7.44 – 7.39 (m, 3H), 7.38 – 7.31 (m, 4H), 7.25 (td, J = 6.9, 1.7 Hz, 1H), 7.16 (t, J = 1.9 Hz, 1H), 7.13 (d, J = 1.8 Hz, 2H), 6.38 (s, 1H), 0.54 (d, J = 9.4 Hz, 6H).

^{13}C NMR (126 MHz, Chloroform-*d*) δ 211.4, 139.7, 136.9, 134.8, 133.9, 133.6, 129.8, 128.9, 128.2, 127.1, 126.7, 126.5, 126.3, 102.1, 92.7, -1.8, -2.0.

HRMS (ESI) m/z: $[M+H]^+$ Calcd. for $C_{23}H_{21}Cl_2Si^+$ 395.0784; Found: 395.0784.

dimethyl(1-(naphthalen-1-yl)-3-phenylpropa-1,2-dien-1-yl)(phenyl)silane (3ac)

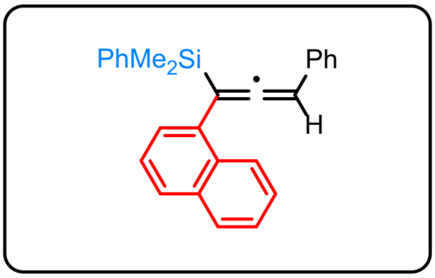

Following the **general procedure C** on 0.2 mmol scale, yellow oil, yield: 98% (73.7 mg), column chromatography (silica gel, PE: EA = 100:0, v/v).

^{1}H NMR (500 MHz, Chloroform-*d*) δ 8.13 (d, J = 8.2 Hz, 1H), 7.85 (dd, J = 7.7, 1.6 Hz, 1H), 7.74 (d, J = 8.2 Hz, 1H), 7.62 – 7.58 (m, 2H), 7.50 – 7.46 (m, 1H), 7.45 – 7.32 (m, 9H), 7.24 – 7.18 (m, 2H), 6.19 (s, 1H), 0.50 (d, J = 10.8 Hz, 6H).

^{13}C NMR (126 MHz, Chloroform-*d*) δ 208.6, 137.6, 134.8, 134.0, 134.0, 131.6, 129.4, 128.7, 128.3, 127.9, 126.8, 126.51, 126.45, 126.2, 125.8, 125.7, 125.6, 125.4, 101.6, 89.7, -2.46, -2.52.

HRMS (ESI) m/z: $[M+H]^+$ Calcd. for $C_{27}H_{25}Si^+$ 377.1720; Found: 377.1721.

dimethyl(phenyl)(3-phenyl-1-(thiophen-2-yl)propa-1,2-dien-1-yl)silane (3ad)

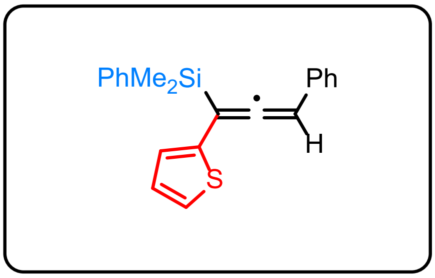

Following the **general procedure C** on 0.2 mmol scale, brown oil, yield: 63% (42 mg), column chromatography (silica gel, PE: EA = 100:0, v/v).

^{1}H NMR (500 MHz, Chloroform-*d*) δ 7.69 – 7.65 (m, 2H), 7.42 – 7.37 (m, 3H), 7.34 (d, J = 4.0 Hz, 4H), 7.24 – 7.20 (m, 1H), 7.12 (dd, J = 5.2, 1.1 Hz, 1H), 6.86 (dd, J = 5.2, 3.6 Hz, 1H), 6.75 (dt, J = 3.6, 0.9 Hz, 1H), 6.35 (s, 1H), 0.55 (d, J = 7.2 Hz, 6H).

^{13}C NMR (126 MHz, Chloroform-*d*) δ 210.5, 139.5, 137.3, 134.2, 134.1, 129.5, 128.8, 128.0, 127.4, 126.8, 126.7, 125.4, 124.6, 97.9, 92.8, -1.7, -1.9.

HRMS (ESI) m/z: $[M+H]^+$ Calcd. for $C_{21}H_{21}SSi^+$ 333.1128; Found: 333.1127.

dimethyl(phenyl)(1-phenylhepta-1,2-dien-3-yl)silane (3ae)

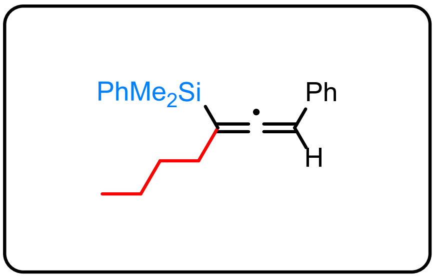

Following the **general procedure C** on 0.2 mmol scale, colorless oil, yield: 78% (47.7 mg), column chromatography (silica gel, PE: EA = 100:0, v/v).

^{1}H NMR (500 MHz, Chloroform-*d*) δ 7.65 – 7.60 (m, 2H), 7.41 (qt, J = 4.3, 2.4 Hz, 3H), 7.35 – 7.28 (m, 4H), 7.21 – 7.16 (m, 1H), 6.00 (t, J = 3.1 Hz, 1H), 2.16 – 2.04 (m, 2H), 1.55 – 1.42 (m, 2H), 1.34 (dt, J = 14.9, 7.4 Hz, 2H), 0.86 (t, J = 7.3 Hz, 3H), 0.46 (d, J = 9.9 Hz, 6H).

^{13}C NMR (126 MHz, Chloroform-*d*) δ 206.5, 137.9, 136.1, 133.9, 129.3, 128.6, 127.9, 126.0, 125.9, 100.1, 90.5, 31.4, 29.5, 22.5, 14.0, -2.77, -2.84.

HRMS (ESI) m/z: $[M+H]^+$ Calcd. for $C_{21}H_{27}Si^+$ 307.1877; Found: 307.1875.

dimethyl(phenyl)(1-phenylnona-1,2-dien-3-yl)silane (3af)

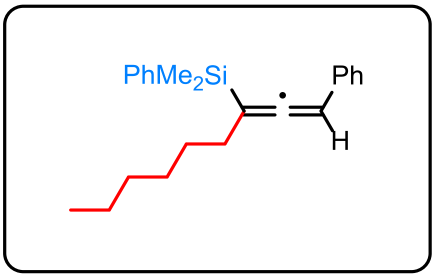

Following the **general procedure C** on 0.2 mmol scale, colorless oil, yield: 89% (59.4 mg), column chromatography (silica gel, PE: EA = 100:0, v/v).

^{1}H NMR (500 MHz, Chloroform-*d*) δ 7.65 – 7.61 (m, 2H), 7.42 (qd, J = 4.1, 1.1 Hz, 3H), 7.35 – 7.29 (m, 4H), 7.22 – 7.17 (m, 1H), 6.01 (t, J = 3.1 Hz, 1H), 2.11 (tttd, J = 12.0, 8.8, 6.1, 5.4, 3.1 Hz, 2H), 1.54 – 1.44 (m, 2H), 1.33 (dd, J = 14.4, 7.4 Hz, 2H), 1.30 – 1.26 (m, 2H), 1.26 – 1.22 (m, 2H), 0.88 (t, J = 7.0 Hz, 3H), 0.47 (d, J = 9.8 Hz, 6H).

^{13}C NMR (126 MHz, Chloroform-*d*) δ 206.5, 137.9, 136.1, 133.9, 129.3, 128.6, 127.9, 126.0, 125.9, 100.1, 90.5, 31.7, 29.8, 29.2, 29.1, 22.7, 14.1, -2.76, -2.84.

HRMS (ESI) m/z: $[M+H]^+$ Calcd. for $C_{23}H_{31}Si^+$ 335.2190; Found: 335.2188.

(3-(4-fluorophenyl)-1-phenylpropa-1,2-dien-1-yl)dimethyl(phenyl)silane (3ag)

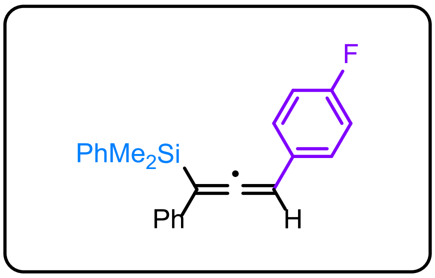

Following the **general procedure C** on 0.2 mmol scale, yellow oil, yield: 96% (65.9 mg), column chromatography (silica gel, PE: EA = 100:0, v/v).

^{1}H NMR (500 MHz, Chloroform-*d*) δ 7.69 – 7.63 (m, 2H), 7.42 – 7.37 (m, 3H), 7.32 – 7.28 (m, 4H), 7.27 – 7.23 (m, 2H), 7.21 – 7.17 (m, 1H), 7.06 – 7.01 (m, 2H), 6.30 (s, 1H), 0.54 (d, J = 11.4 Hz, 6H).

^{19}F NMR (471 MHz, Chloroform-*d*) δ -115.8.

^{13}C NMR (126 MHz, Chloroform-*d*) δ 210.7 (d, J = 2.3 Hz), 161.8 (d, J = 245.3 Hz), 137.9, 136.0, 134.0, 130.4 (d, J = 3.2 Hz), 129.4, 128.6, 128.1, 128.0, 127.6 (d, J = 7.8 Hz), 126.8, 115.7 (d, J = 21.8 Hz), 103.5, 91.0, -1.6, -1.7.

HRMS (ESI) m/z: $[M+H]^+$ Calcd. for $C_{23}H_{22}FSi^+$ 345.1469; Found: 345.1469.

(3-(4-chlorophenyl)-1-phenylpropa-1,2-dien-1-yl)dimethyl(phenyl)silane (3ah)

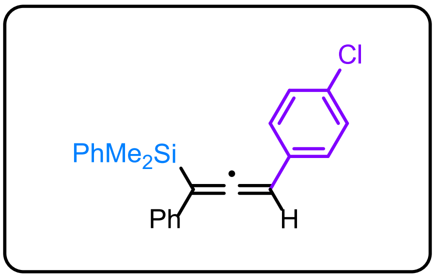

Following the **general procedure C** on 0.2 mmol scale, yellow oil, yield: 81% (58.5 mg), column chromatography (silica gel, PE: EA = 100:0, v/v).

^{1}H NMR (500 MHz, Chloroform-*d*) δ 7.69 – 7.64 (m, 2H), 7.43 – 7.38 (m, 3H), 7.33 – 7.24 (m, 8H), 7.20 (t, J = 7.1 Hz, 1H), 6.29 (s, 1H), 0.55 (d, J = 11.3 Hz, 6H).

^{13}C NMR (126 MHz, Chloroform-*d*) δ 210.7, 137.8, 135.8, 134.0, 133.2, 132.1, 129.5, 129.0, 128.6, 128.09, 128.08, 127.5, 126.9, 103.7, 91.1, -1.6, -1.7.

HRMS (ESI) m/z: $[M+H]^+$ Calcd. for $C_{23}H_{22}ClSi^+$ 361.1174; Found: 361.1174.

(3-(4-bromophenyl)-1-phenylpropa-1,2-dien-1-yl)dimethyl(phenyl)silane (3ai)

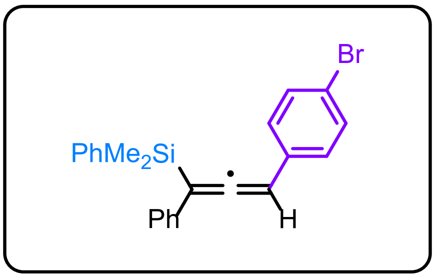

Following the **general procedure C** on 0.2 mmol scale, yellow oil, yield: 54% (43.6 mg), column chromatography (silica gel, PE: EA = 100:0, v/v).

^{1}H NMR (500 MHz, Chloroform-*d*) δ 7.67 – 7.62 (m, 2H), 7.46 – 7.42 (m, 2H), 7.40 (dtd, J = 7.0, 5.5, 5.0, 2.1 Hz, 3H), 7.30 – 7.27 (m, 2H), 7.26 – 7.23 (m, 2H), 7.22 – 7.17 (m, 3H), 6.26 (s, 1H), 0.53 (d, J = 11.2 Hz, 6H).

^{13}C NMR (126 MHz, Chloroform-*d*) δ 210.6, 137.8, 135.7, 133.9, 133.7, 131.9, 129.5, 128.6, 128.08, 128.06, 127.8, 126.9, 120.1, 103.7, 91.2, -1.66, -1.68.

HRMS (ESI) m/z: $[M+H]^+$ Calcd. for $C_{23}H_{22}BrSi^+$ 405.0669; Found: 405.0669.

(3-(4-(tert-butyl)phenyl)-1-phenylpropa-1,2-dien-1-yl)dimethyl(phenyl)silane (3aj)

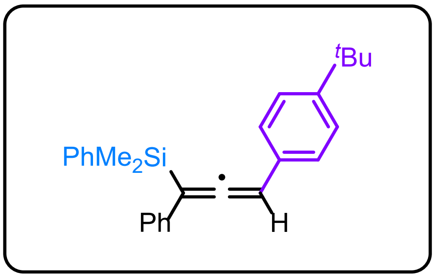

Following the **general procedure C** on 0.2 mmol scale, yellow oil, yield: 98% (74.9 mg), column chromatography (silica gel, PE: EA = 100:0, v/v).

^{1}H NMR (500 MHz, Chloroform-*d*) δ 7.73 – 7.69 (m, 2H), 7.44 – 7.39 (m, 5H), 7.35 – 7.31 (m, 4H), 7.27 – 7.23 (m, 2H), 7.20 – 7.16 (m, 1H), 6.37 (s, 1H), 1.38 (s, 9H), 0.56 (d, J = 9.3 Hz, 6H).

^{13}C NMR (126 MHz, Chloroform-*d*) δ 211.2, 149.7, 136.3, 134.1, 131.5, 129.4, 128.5, 128.12, 128.06, 126.7, 126.1, 125.8, 103.0, 91.8, 34.6, 31.4, -1.49, -1.53.

HRMS (ESI) m/z: $[M+H]^+$ Calcd. for $C_{27}H_{31}Si^+$ 383.2190; Found: 383.2189.

dimethyl(phenyl)(1-phenyl-3-(4-(trifluoromethoxy)phenyl)propa-1,2-dien-1-yl)silane (3ak)

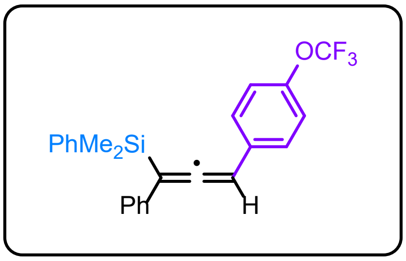

Following the **general procedure C** on 0.2 mmol scale, yellow oil, yield: 86% (70.8 mg), column chromatography (silica gel, PE: EA = 100:0, v/v).

^{1}H NMR (500 MHz, Chloroform-*d*) δ 7.71 – 7.67 (m, 2H), 7.45 – 7.40 (m, 3H), 7.39 – 7.35 (m, 2H), 7.34 – 7.31 (m, 2H), 7.30 – 7.26 (m, 2H), 7.22 (tt, J = 8.0, 1.3 Hz, 3H), 6.34 (s, 1H), 0.57 (d, J = 12.3 Hz, 6H).

^{19}F NMR (471 MHz, Chloroform-*d*) δ -57.8.

^{13}C NMR (126 MHz, Chloroform-*d*) δ 210.7, 147.8, 137.8, 135.8, 134.0, 133.6, 129.1, 128.7, 128.1, 127.4, 126.9, 121.5, 120.6 (q, J = 256.8 Hz), 103.7, 90.9, -1.6, -1.7.

HRMS (ESI) m/z: $[M+H]^+$ Calcd. for $C_{24}H_{22}F_3OSi^+$ 411.1387; Found: 411.1387.

dimethyl(phenyl)(1-phenyl-3-(4-(trifluoromethyl)phenyl)propa-1,2-dien-1-yl)silane (3al)

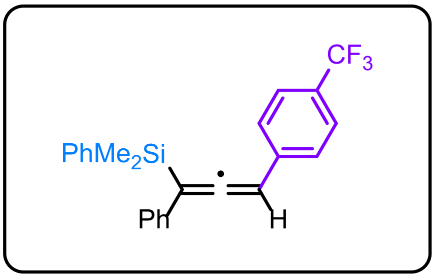

Following the **general procedure C** on 0.2 mmol scale, yellow oil, yield: 53% (42.1 mg), column chromatography (silica gel, PE: EA = 100:0, v/v).

^{1}H NMR (500 MHz, Chloroform-*d*) δ 7.68 – 7.64 (m, 2H), 7.58 (d, J = 8.1 Hz, 2H), 7.44 – 7.38 (m, 5H), 7.30 (dd, J = 8.4, 1.4 Hz, 2H), 7.28 – 7.24 (m, 2H), 7.22 – 7.18 (m, 1H), 6.34 (s, 1H), 0.55 (d, J = 12.3 Hz, 6H).

^{19}F NMR (471 MHz, Chloroform-*d*) δ -62.2.

^{13}C NMR (126 MHz, Chloroform-*d*) δ 210.8, 138.8, 137.6, 135.4, 133.9, 129.6, 128.7, 128.4 (q, J = 32.3 Hz), 128.11, 128.10, 127.0, 126.3, 125.7 (q, J = 3.8 Hz), 124.4 (q, J = 271.7 Hz), 103.8, 91.1, -1.7.

HRMS (ESI) m/z: $[M+H]^+$ Calcd. for $C_{24}H_{22}F_3Si^+$ 395.1437; Found: 395.1433.

(3-([1,1'-biphenyl]-4-yl)-1-phenylpropa-1,2-dien-1-yl)dimethyl(phenyl)silane (3am)

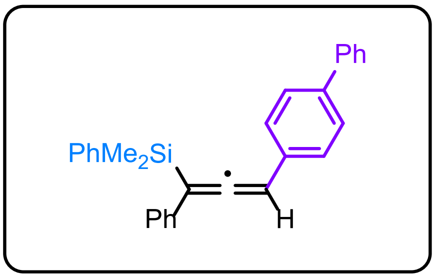

Following the **general procedure C** on 0.2 mmol scale, yellow oil, yield: 83% (66.6 mg), column chromatography (silica gel, PE: EA = 100:0, v/v).

^{1}H NMR (500 MHz, Chloroform-*d*) δ 7.74 – 7.71 (m, 2H), 7.68 – 7.65 (m, 2H), 7.64 – 7.61 (m, 2H), 7.52 – 7.42 (m, 7H), 7.42 – 7.38 (m, 1H), 7.38 – 7.35 (m, 2H), 7.31 – 7.26 (m, 2H), 7.24 – 7.20 (m, 1H), 6.42 (s, 1H), 0.60 (d, J = 10.6 Hz, 6H).

^{13}C NMR (126 MHz, Chloroform-*d*) δ 211.2, 140.9, 139.5, 138.1, 136.1, 134.0, 133.7, 129.4, 128.9, 128.6, 128.2, 128.1, 127.6, 127.3, 127.0, 126.8, 103.3, 91.7, -1.5.

HRMS (ESI) m/z: $[M+H]^+$ Calcd. for $C_{29}H_{27}Si^+$ 403.1877; Found: 403.1879.

dimethyl(3-(naphthalen-1-yl)-1-phenylpropa-1,2-dien-1-yl)(phenyl)silane (3an)

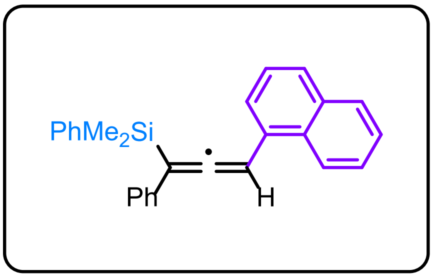

Following the **general procedure C** on 0.2 mmol scale, yellow oil, yield: 72% (53.8 mg), column chromatography (silica gel, PE: EA = 100:0, v/v).

^{1}H NMR (500 MHz, Chloroform-*d*) δ 8.33 – 8.29 (m, 1H), 7.93 – 7.89 (m, 1H), 7.78 (d, J = 8.2 Hz, 1H), 7.73 – 7.70 (m, 2H), 7.68 (dd, J = 7.1, 1.2 Hz, 1H), 7.57 – 7.53 (m, 2H), 7.51 – 7.47 (m, 1H), 7.42 (dddd, J = 7.1, 5.3, 3.6, 1.5 Hz, 5H), 7.31 – 7.27 (m, 2H), 7.24 – 7.20 (m, 1H), 7.07 (s, 1H), 0.60 (d, J = 5.0 Hz, 6H).

^{13}C NMR (126 MHz, Chloroform-*d*) δ 212.0, 138.1, 136.2, 134.2, 134.1, 130.8, 130.7, 129.4, 128.8, 128.7, 128.2, 128.1, 127.2, 126.7, 126.0, 125.83, 125.79, 124.7, 123.7, 101.9, 88.5, -1.5, -1.6.

HRMS (ESI) m/z: $[M+H]^+$ Calcd. for $C_{27}H_{25}Si^+$ 377.1720; Found: 377.1725.

(1,5-diphenylpenta-1,2-dien-1-yl)dimethyl(phenyl)silane (3ao)

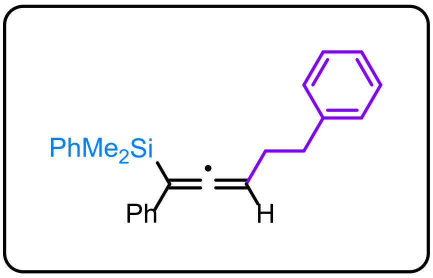

Following the **general procedure C** on 0.2 mmol scale, yellow oil, yield: 48% (33.8 mg), column chromatography (silica gel, PE: EA = 100:0, v/v).

^{1}H NMR (500 MHz, Chloroform-*d*) δ 7.68 – 7.61 (m, 2H), 7.40 (dd, J = 5.3, 1.8 Hz, 3H), 7.33 (t, J = 7.5 Hz, 2H), 7.27 – 7.14 (m, 8H), 5.30 (t, J = 6.7 Hz, 1H), 2.86 – 2.74 (m, 2H), 2.47 (tt, J = 8.1, 6.0 Hz, 2H), 0.50 (s, 6H).

^{13}C NMR (126 MHz, Chloroform-*d*) δ 209.6, 141.8, 138.6, 137.4, 134.0, 129.2, 128.6, 128.44, 128.38, 127.93, 127.86, 126.1, 126.0, 98.9, 87.3, 36.0, 30.3, -1.56, -1.60.

HRMS (ESI) m/z: $[M+H]^+$ Calcd. for $C_{25}H_{27}Si^+$ 355.1877; Found: 355.1876.

dimethyl(4-methyl-1-phenylpenta-1,2-dien-1-yl)(phenyl)silane (3ap)

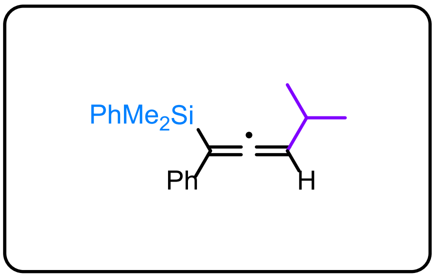

Following the **general procedure C** on 0.2 mmol scale, colorless oil, yield: 34% (19.7 mg), column chromatography (silica gel, PE: EA = 100:0, v/v).

^{1}H NMR (500 MHz, Chloroform-*d*) δ 7.63 – 7.60 (m, 2H), 7.39 – 7.36 (m, 3H), 7.26 (dd, J = 8.2, 1.3 Hz, 2H), 7.22 (dd, J = 8.5, 6.7 Hz, 2H), 7.16 – 7.11 (m, 1H), 5.29 (d, J = 5.8 Hz, 1H), 2.45 (dq, J = 13.4, 6.7 Hz, 1H), 1.10 (dd, J = 6.7, 2.0 Hz, 6H), 0.48 (d, J = 3.7 Hz, 6H).

^{13}C NMR (126 MHz, Chloroform-*d*) δ 208.4, 138.7, 137.7, 134.0, 129.1, 128.3, 127.9, 127.7, 126.0, 99.6, 95.5, 27.9, 23.0, 22.8, -1.5, -1.6.

HRMS (ESI) m/z: $[M+H]^+$ Calcd. for $C_{20}H_{25}Si^+$ 293.1720; Found: 293.1722.

(1,3-diphenylpropa-1,2-dien-1-yl)triethylsilane (4)

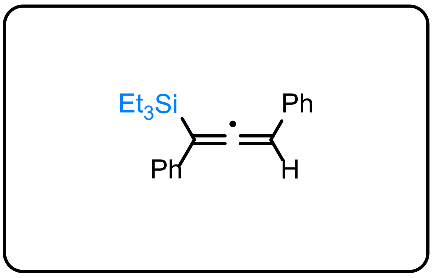

Following the **general procedure D** on 0.2 mmol scale, yellow oil, yield: 54% (32.9 mg), column chromatography (silica gel, PE: EA = 100:0, v/v).

^{1}H NMR (500 MHz, Chloroform-*d*) δ 7.40 – 7.30 (m, 8H), 7.25 – 7.18 (m, 2H), 6.23 (s, 1H), 1.02 (t, J = 7.8 Hz, 9H), 0.88 – 0.76 (m, 6H).

^{13}C NMR (126 MHz, Chloroform-*d*) δ 210.2, 137.2, 134.9, 128.7, 128.6, 127.7, 126.6, 126.4, 126.3, 101.8, 90.8, 7.5, 4.0.

HRMS (ESI) m/z: $[M+H]^+$ Calcd. for $C_{21}H_{27}Si^+$ 307.1877; Found: 307.1876.

8. NMR spectra

8.1 NMR spectra of substrates

^{1}H NMR spectrum ($CDCl_3$) of **1a**

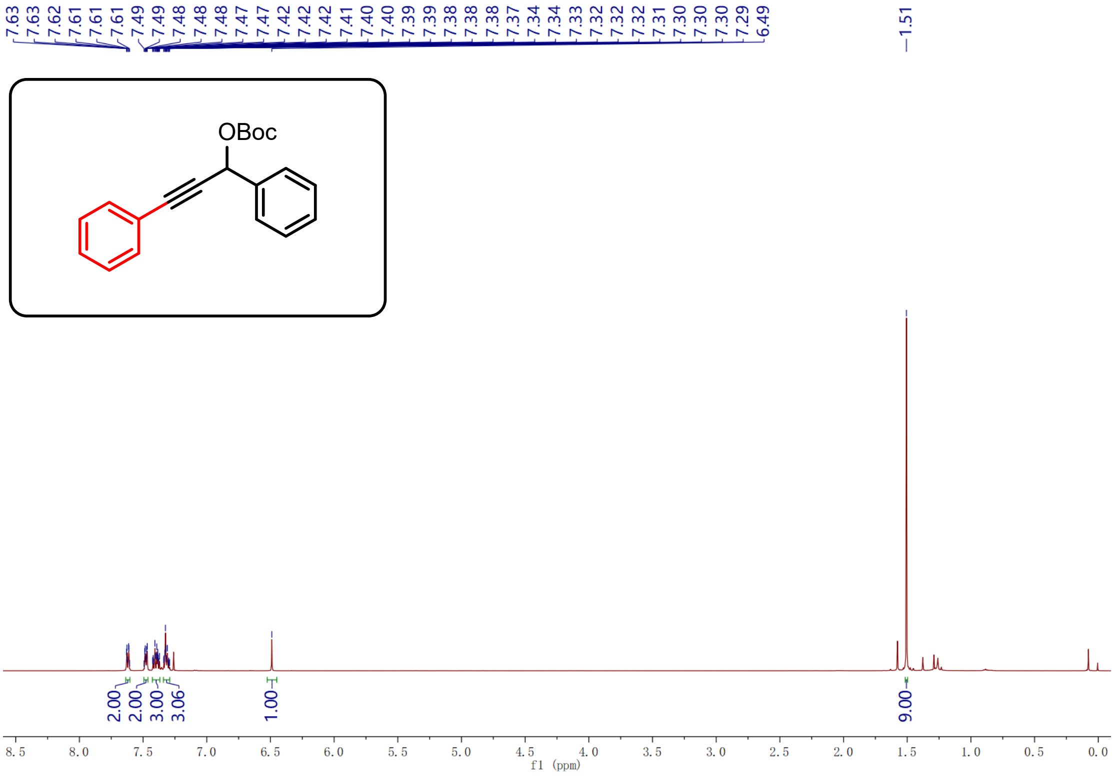

^{13}C NMR spectrum ($CDCl_3$) of **1a**

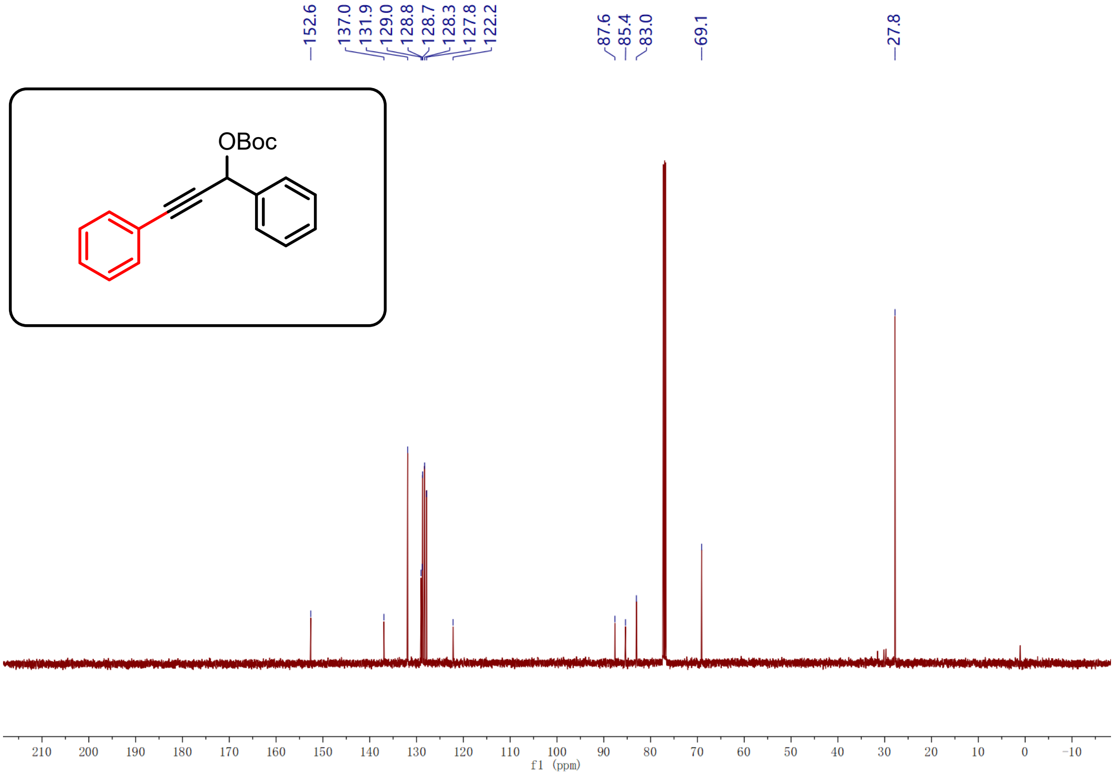

^{1}H NMR spectrum ($CDCl_3$) of **1b**

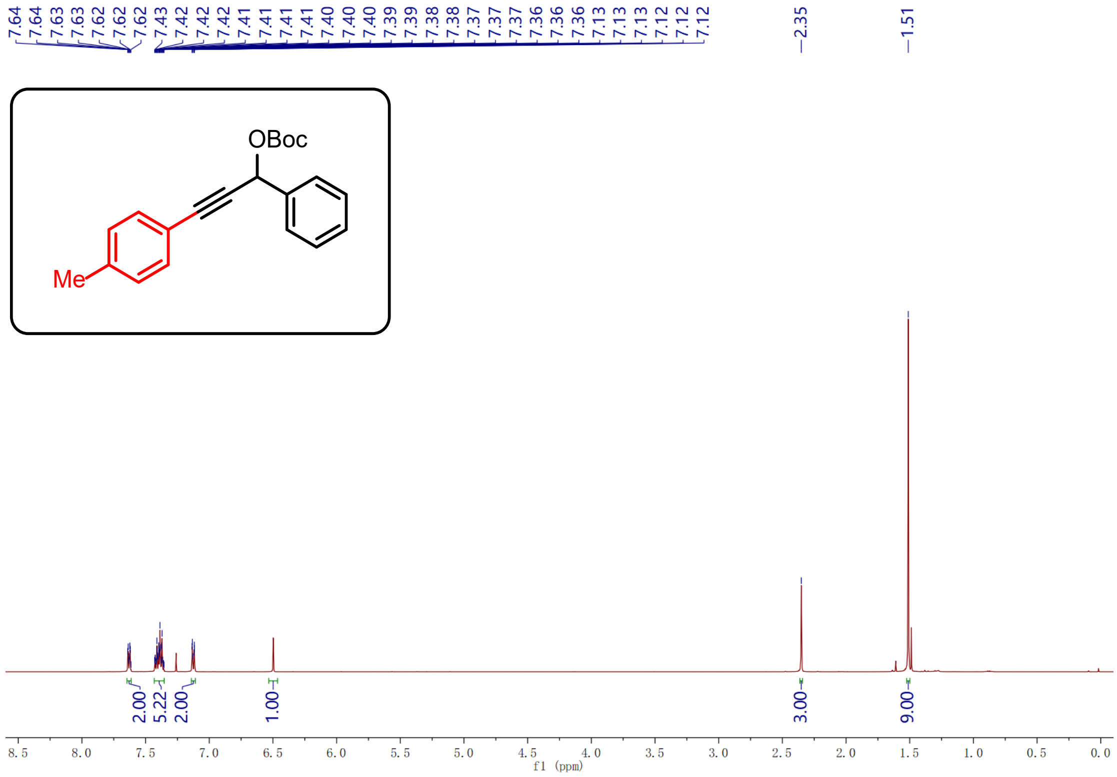

^{13}C NMR spectrum ($CDCl_3$) of **1b**

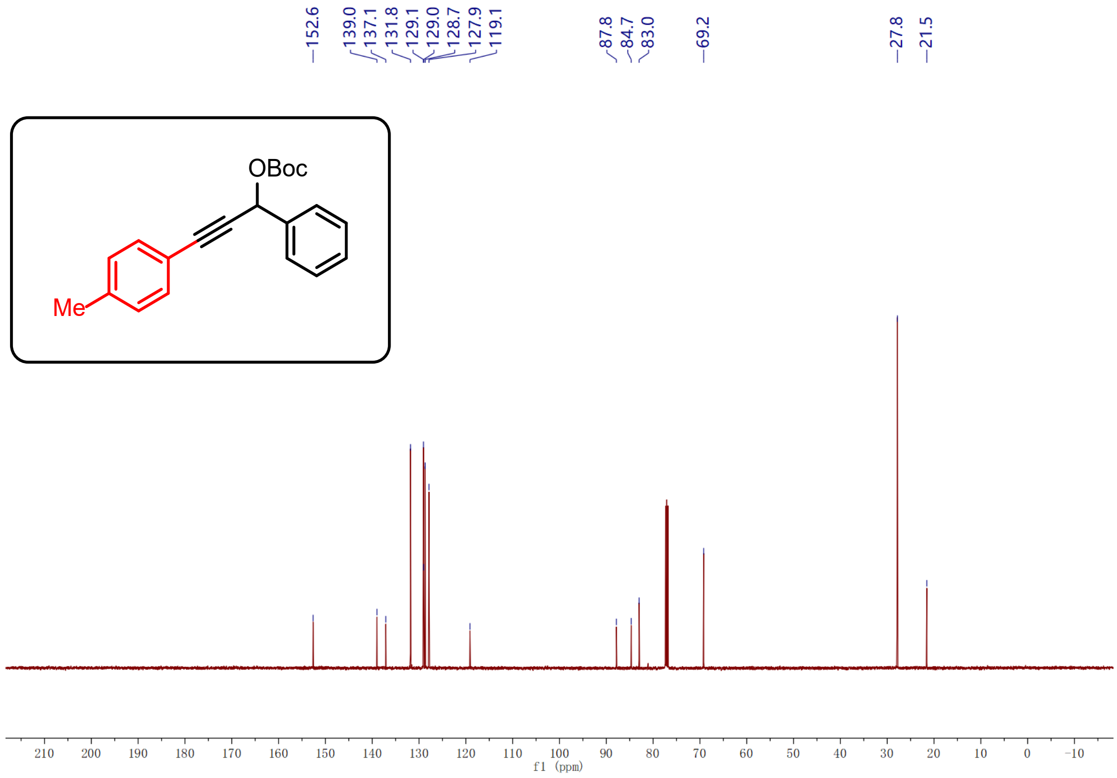

^{1}H NMR spectrum ($CDCl_3$) of **1c**

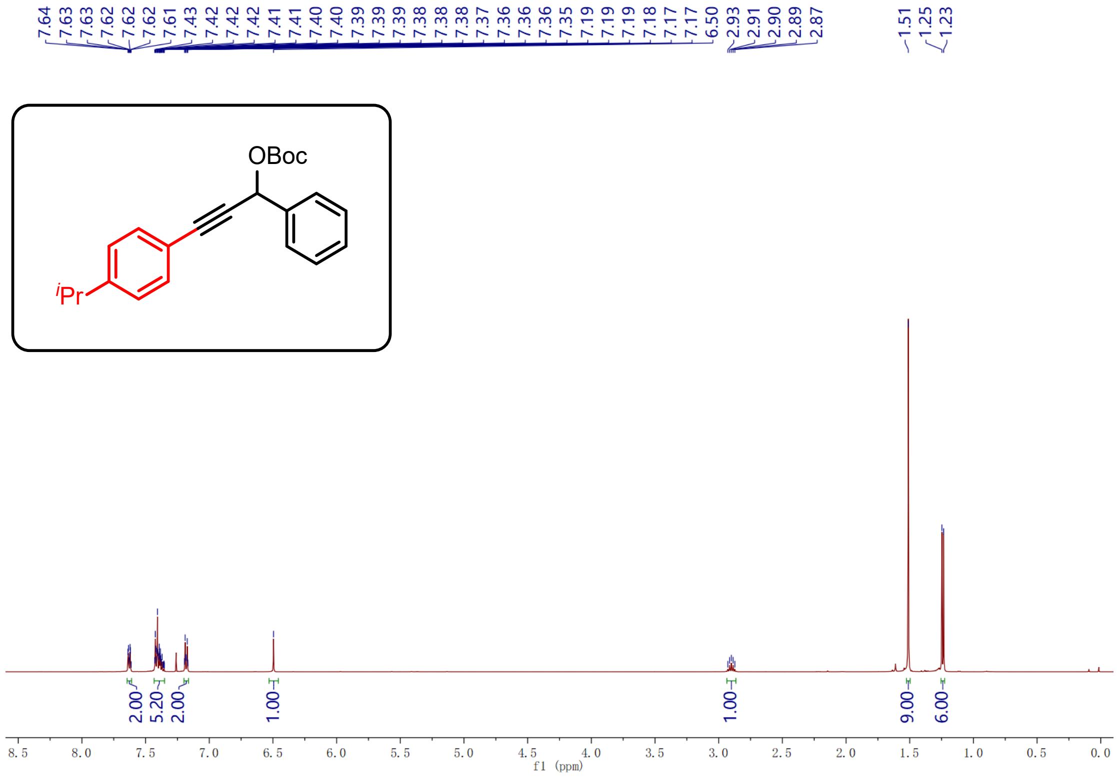

^{13}C NMR spectrum ($CDCl_3$) of **1c**

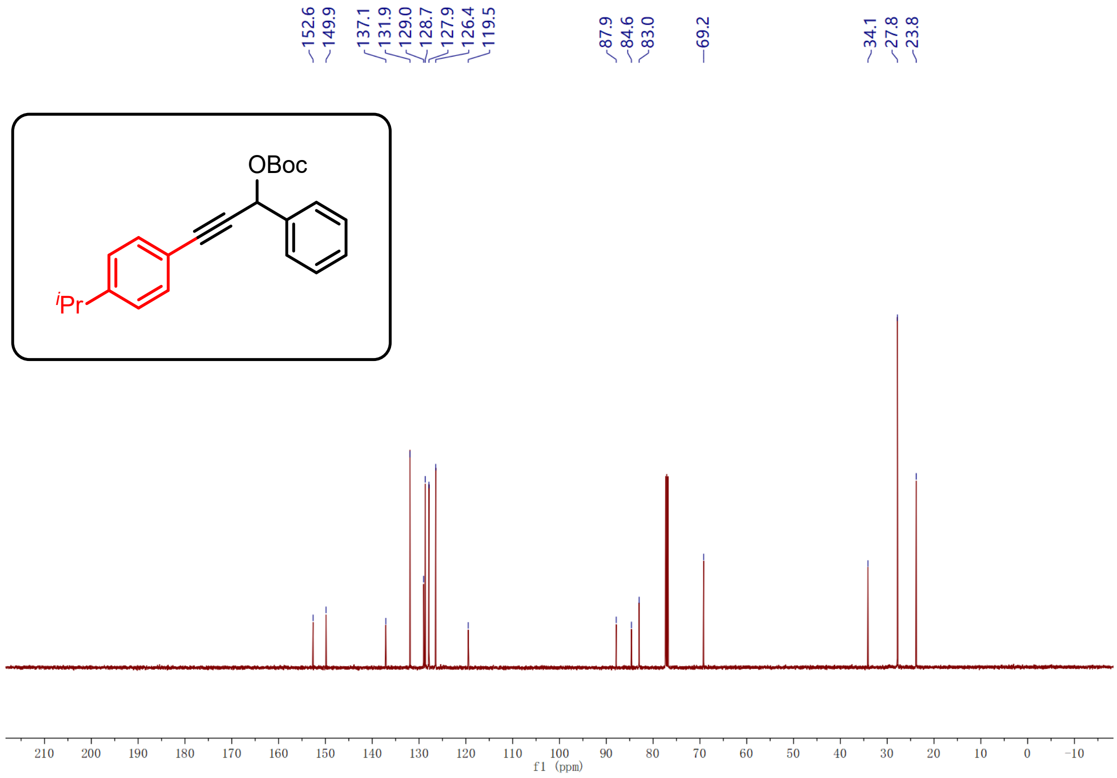

^{1}H NMR spectrum ($CDCl_3$) of **1d**

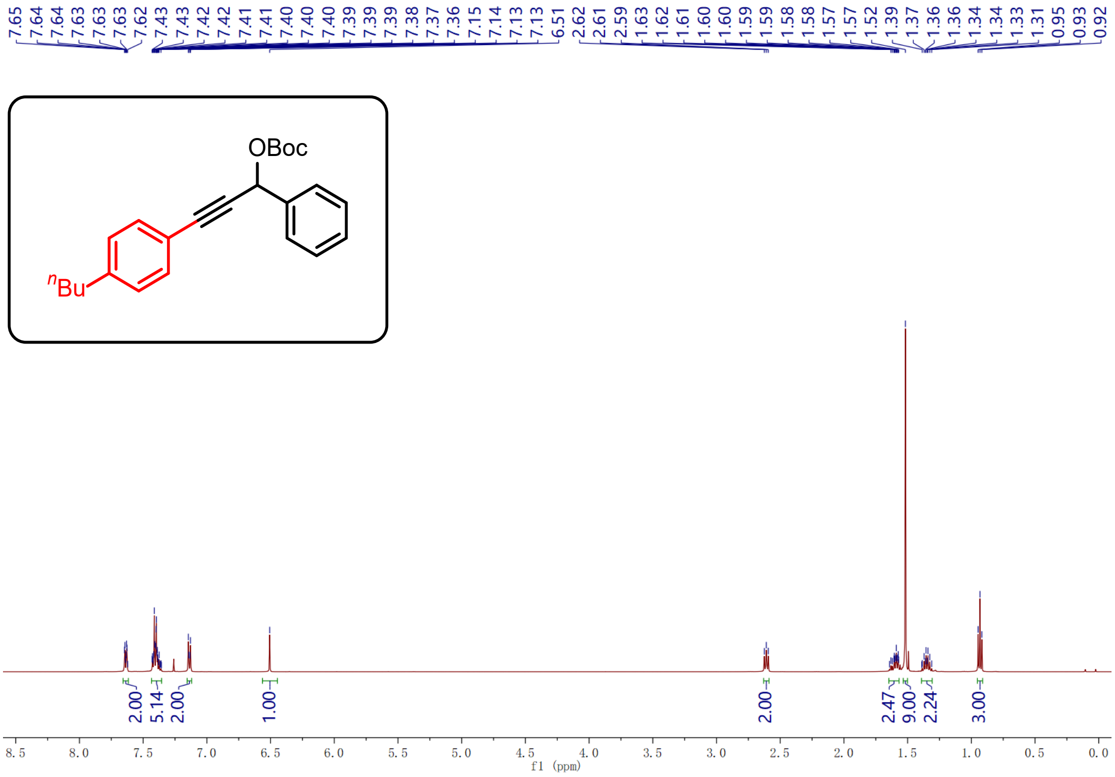

^{13}C NMR spectrum ($CDCl_3$) of **1d**

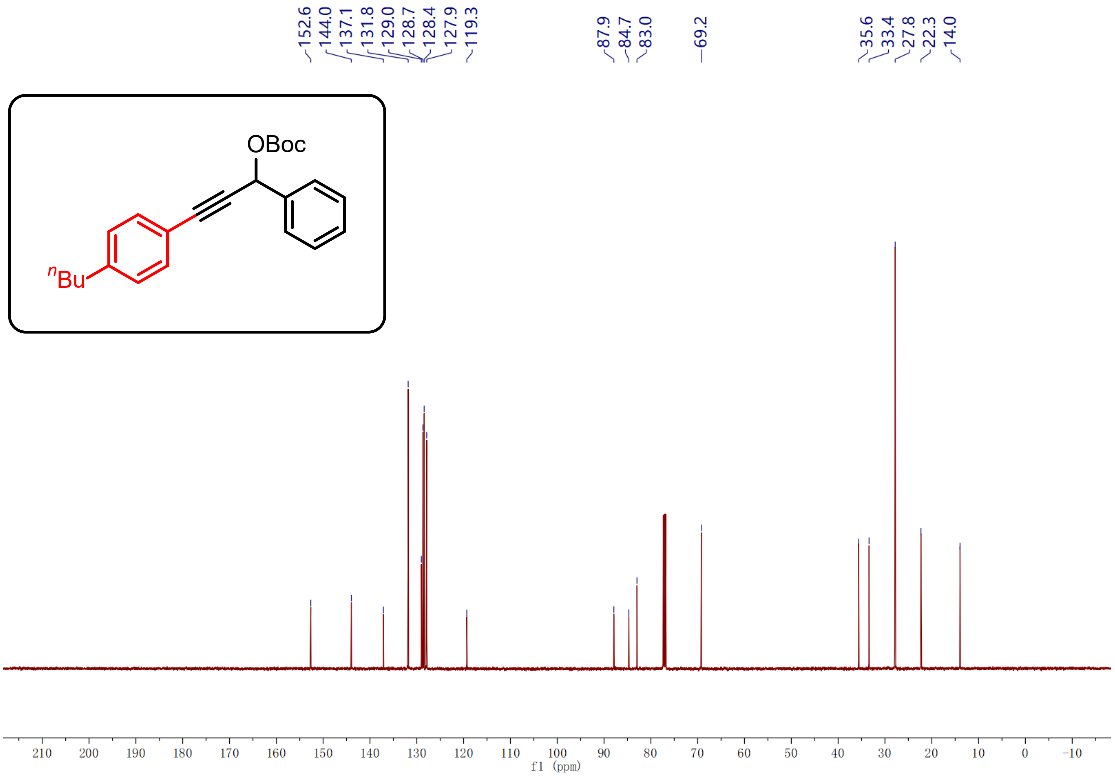

^{1}H NMR spectrum ($CDCl_3$) of **1e**

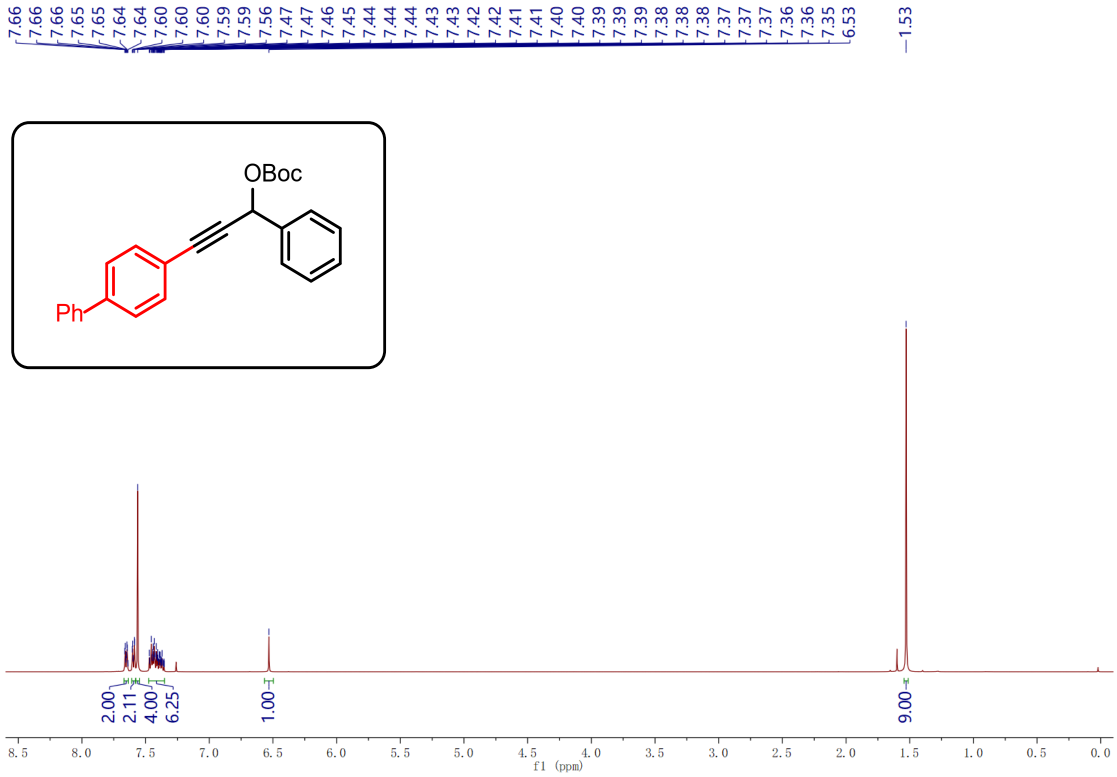

^{13}C NMR spectrum ($CDCl_3$) of **1e**

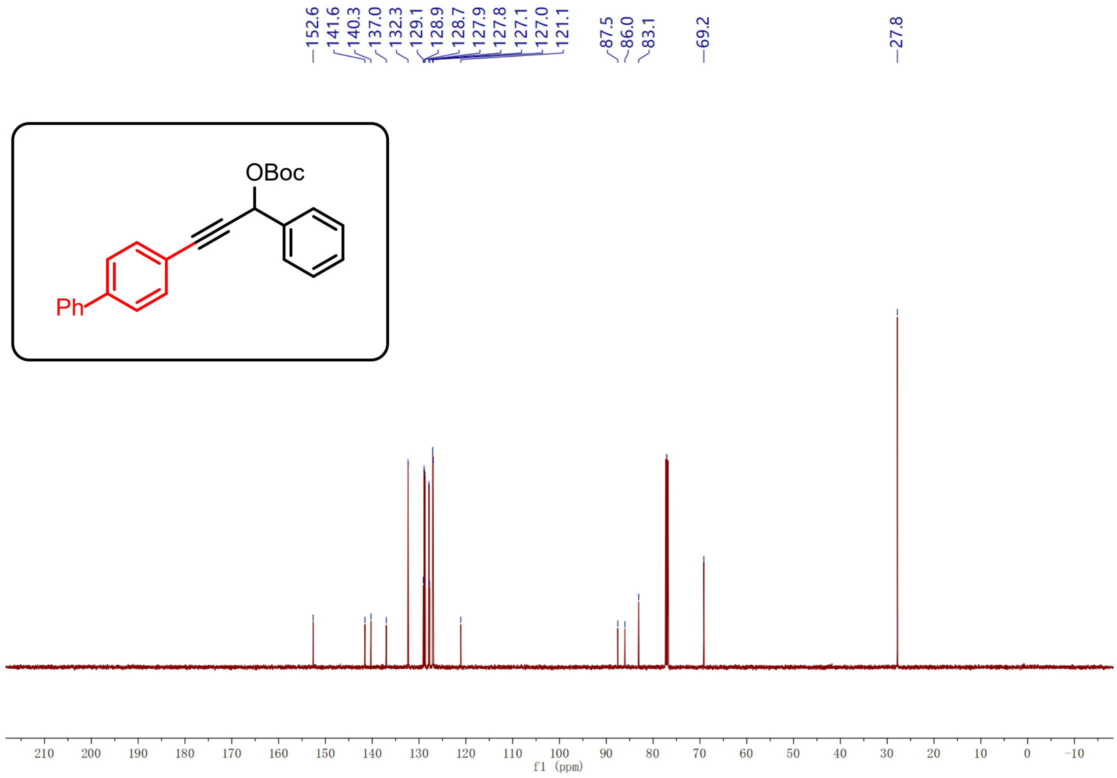

^{1}H NMR spectrum ($CDCl_3$) of **1f**

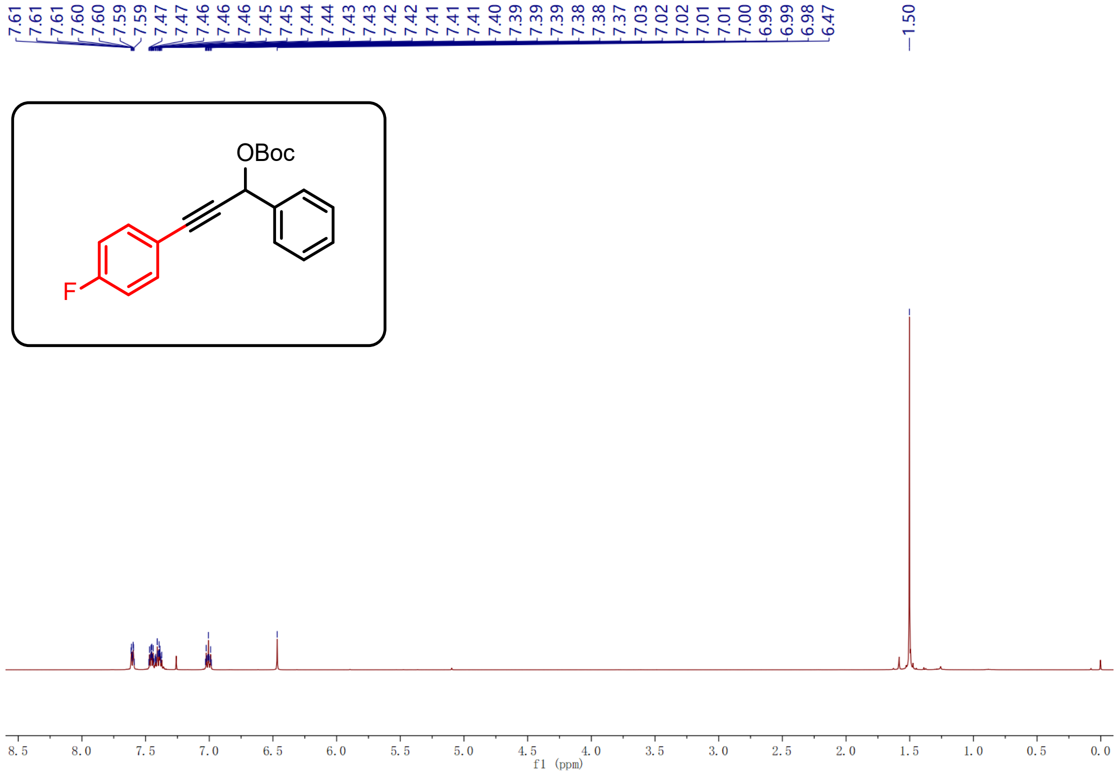

^{13}C NMR spectrum ($CDCl_3$) of **1f**

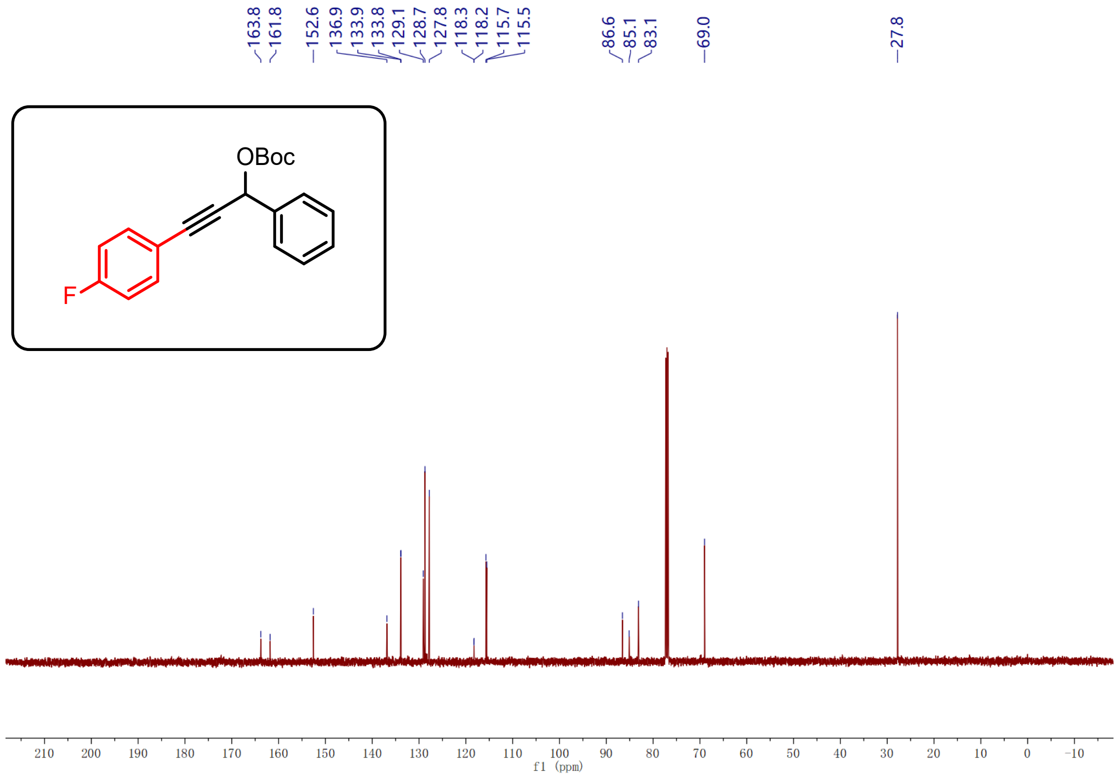

^{19}F NMR spectrum ($CDCl_3$) of **1f**

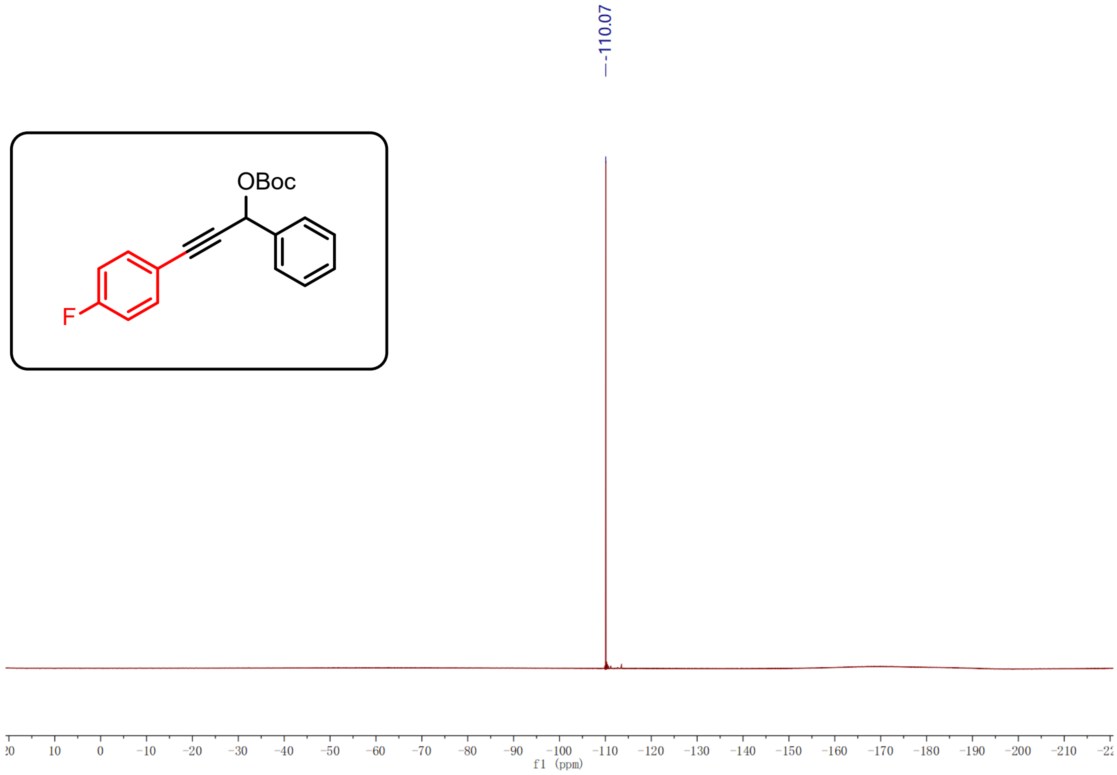

^{1}H NMR spectrum ($CDCl_3$) of **1g**

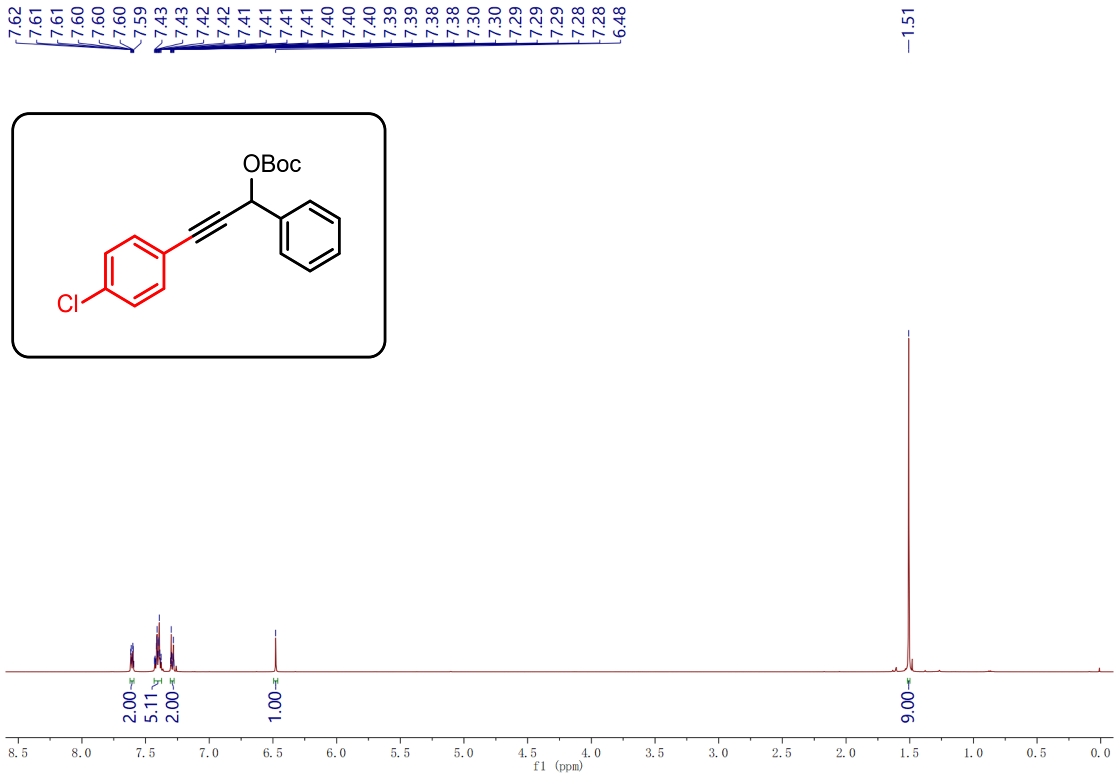

^{13}C NMR spectrum ($CDCl_3$) of **1g**

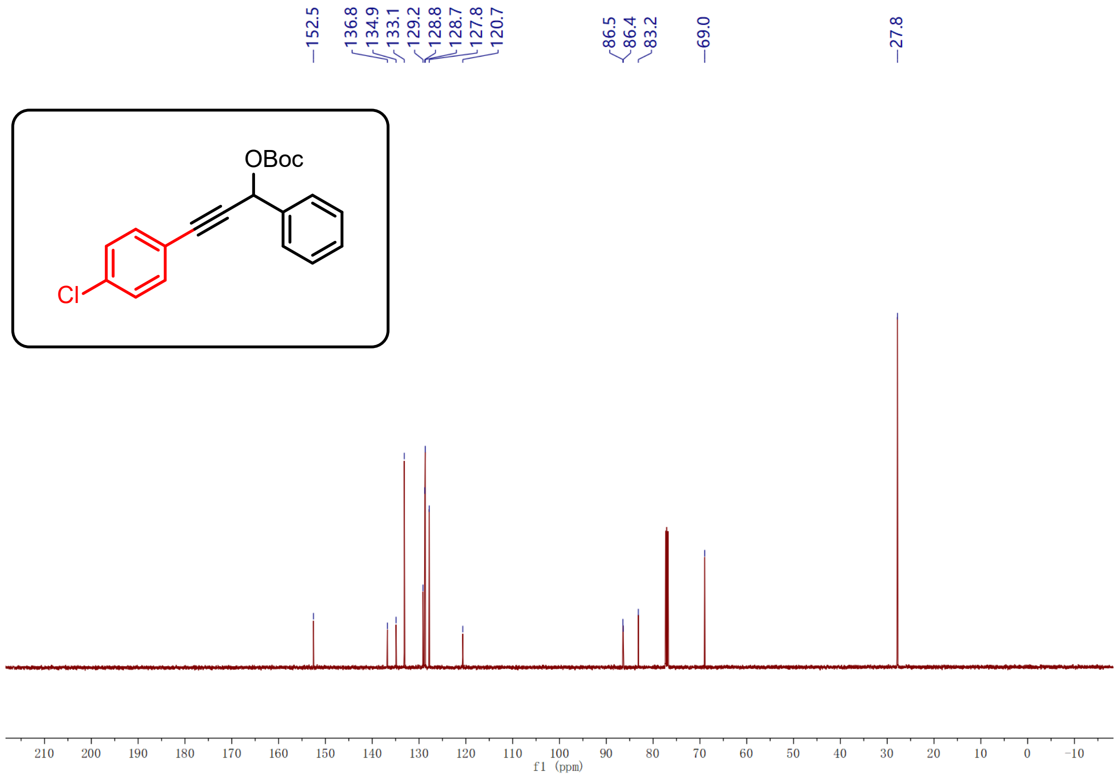

^{1}H NMR spectrum ($CDCl_3$) of **1h**

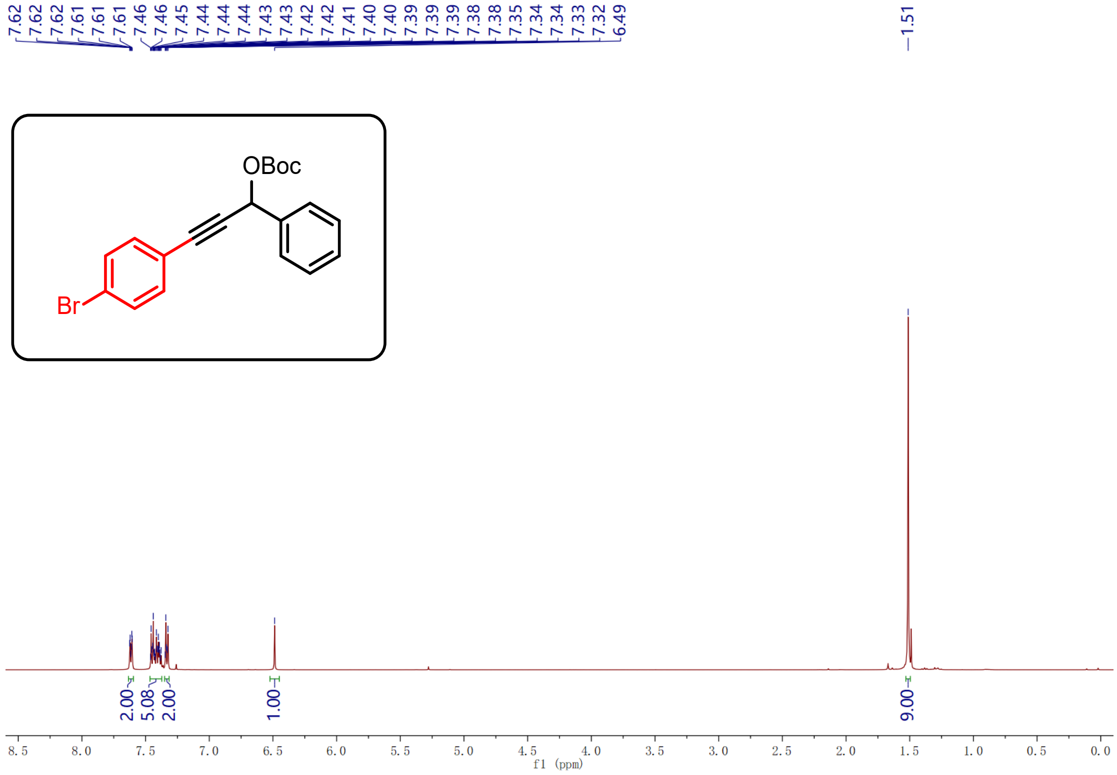

^{13}C NMR spectrum ($CDCl_3$) of **1h**

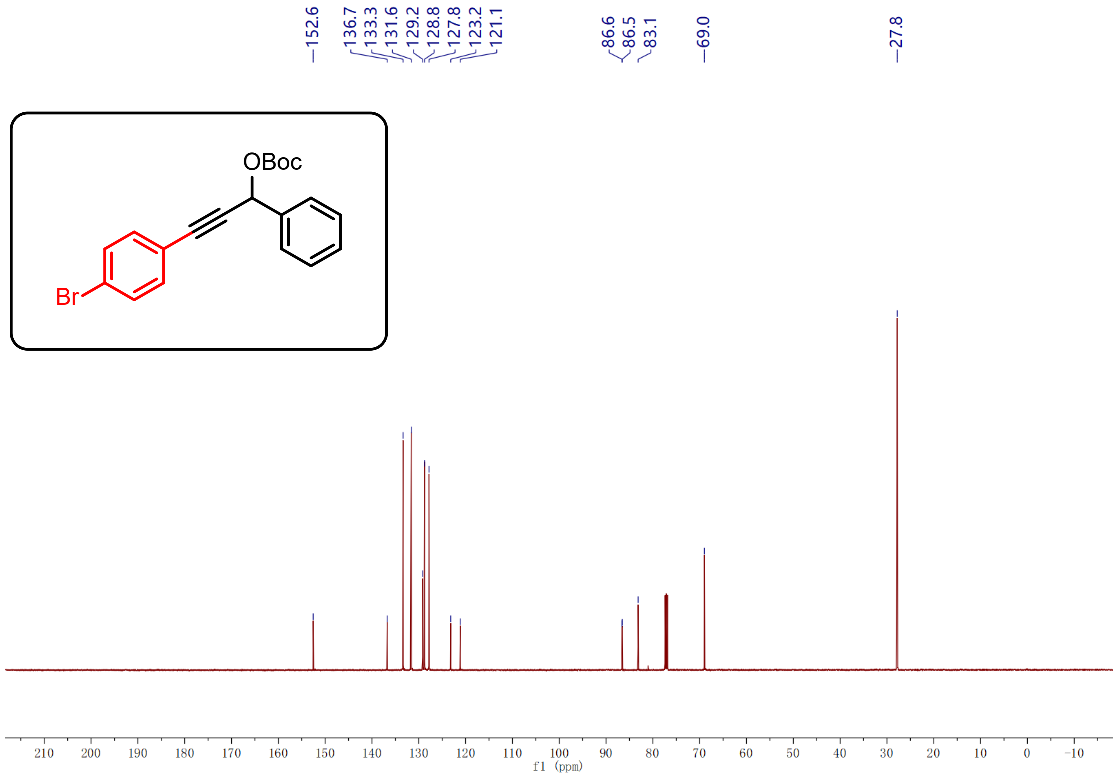

^{1}H NMR spectrum ($CDCl_3$) of **1i**

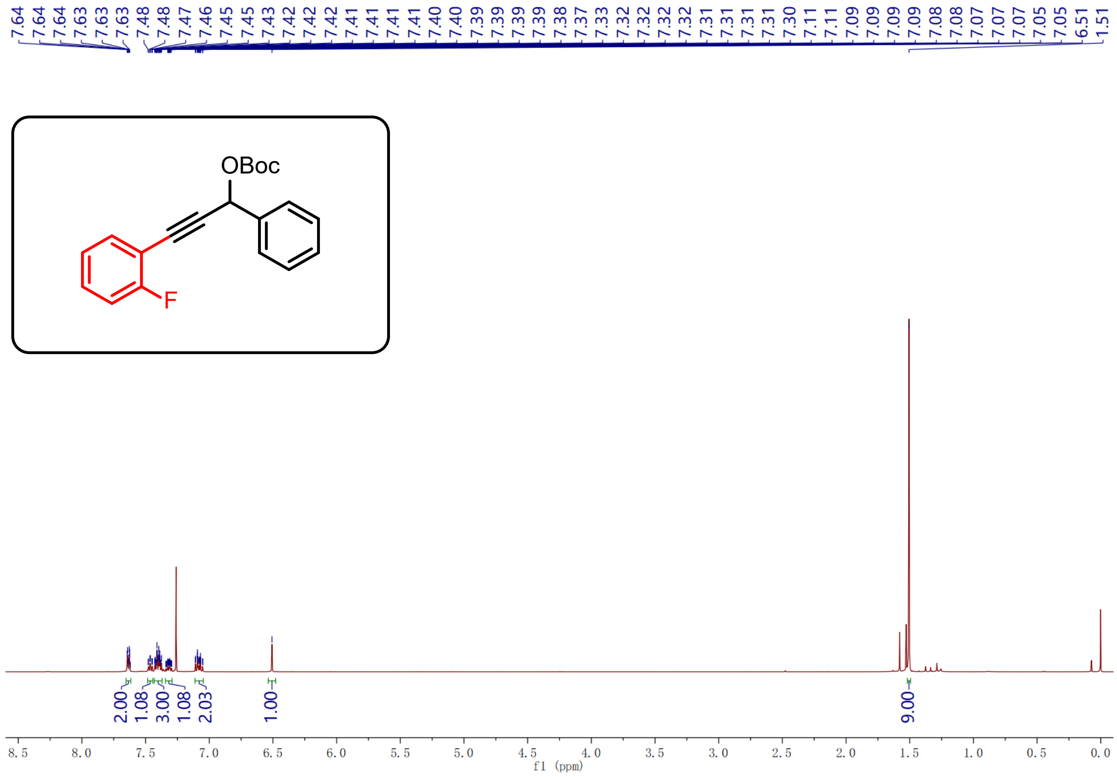

^{13}C NMR spectrum ($CDCl_3$) of **1i**

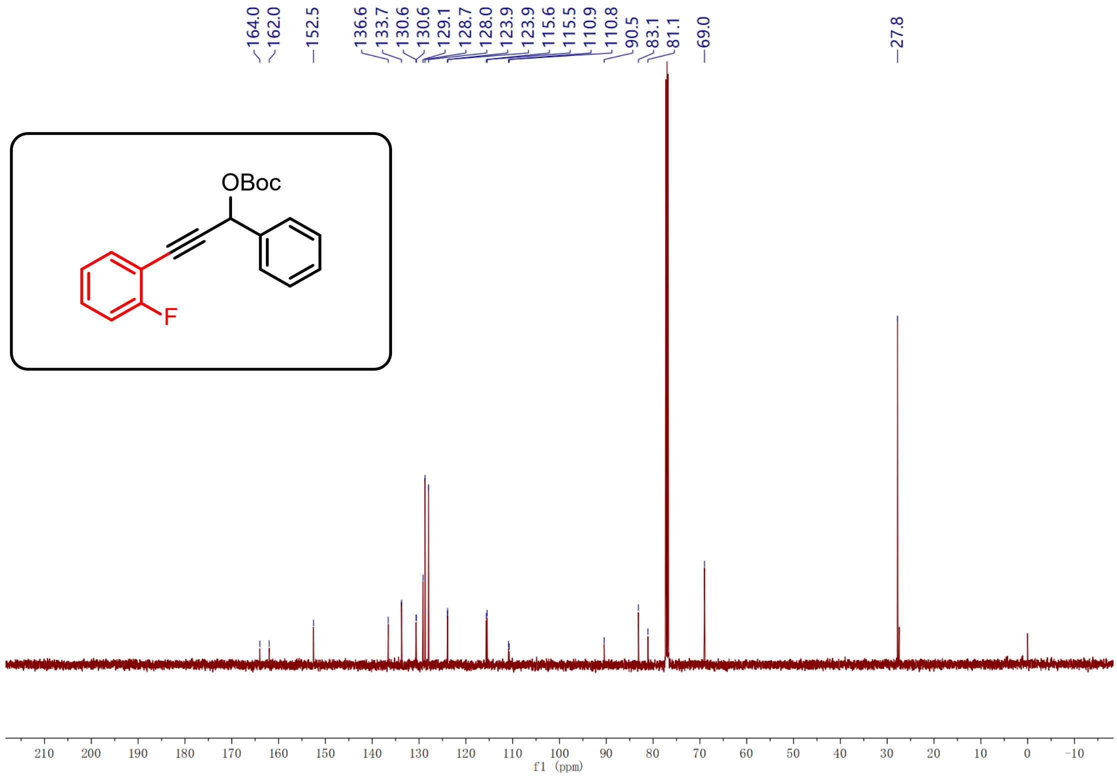

^{19}F NMR spectrum ($CDCl_3$) of **1i**

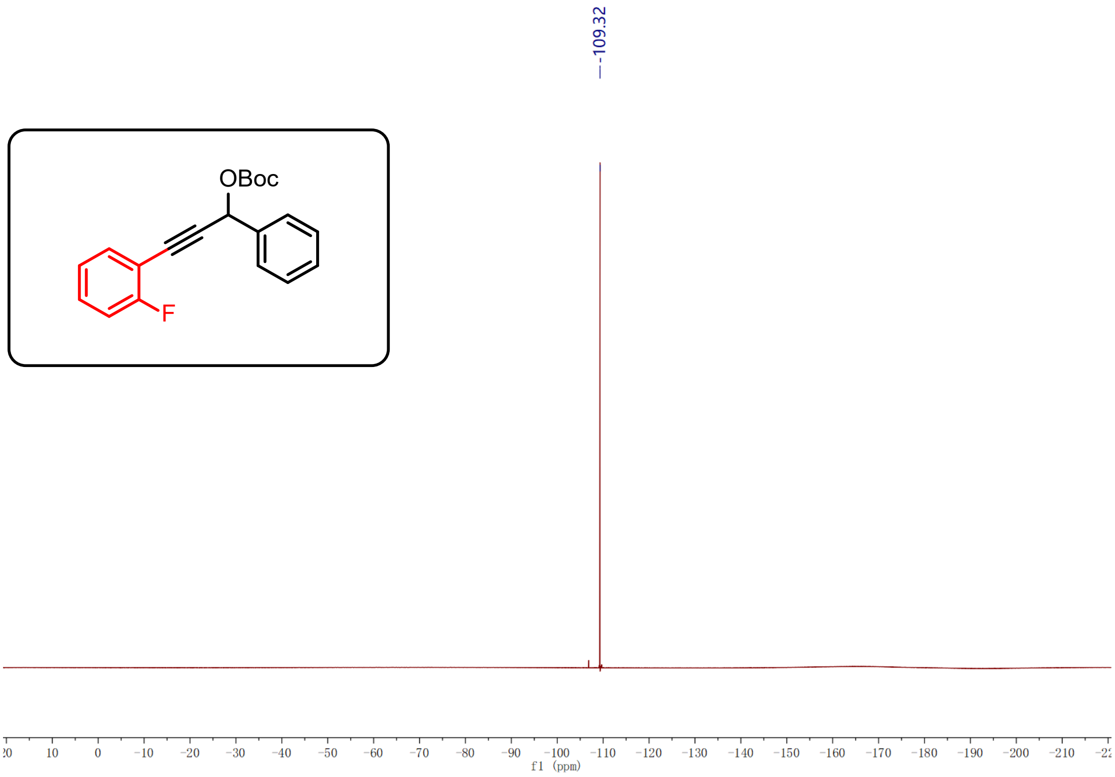

^{1}H NMR spectrum ($CDCl_3$) of **1j**

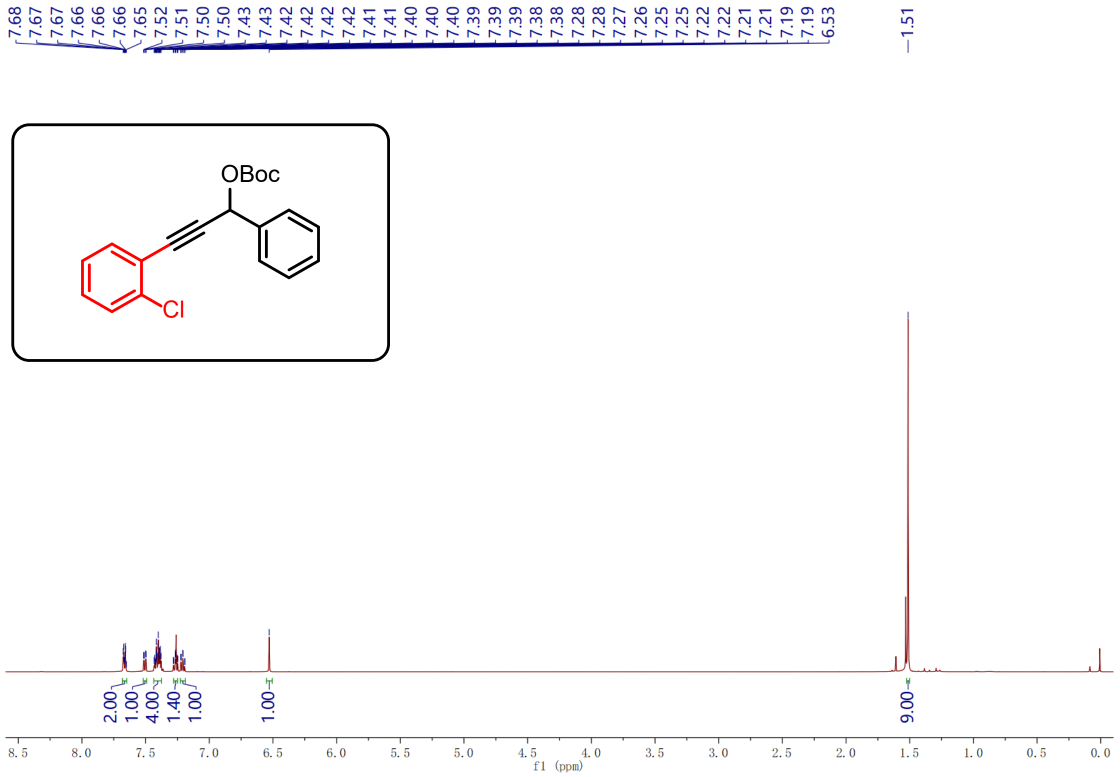

^{13}C NMR spectrum ($CDCl_3$) of **1j**

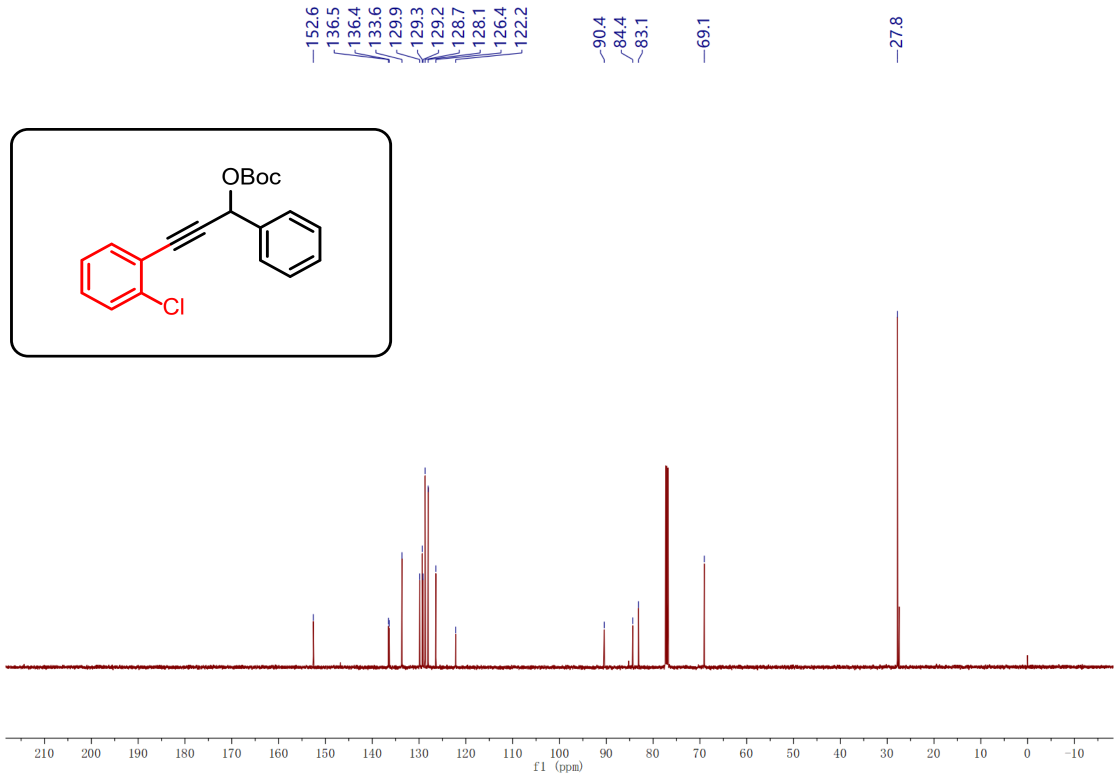

^{1}H NMR spectrum ($CDCl_3$) of **1k**

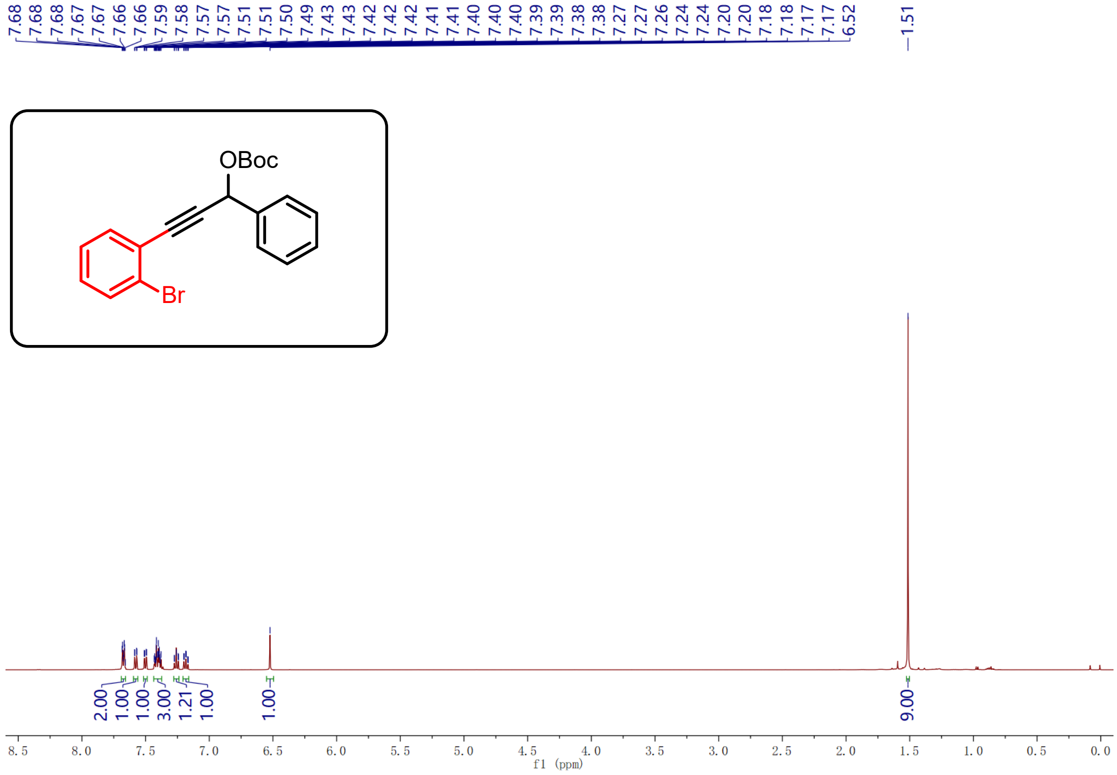

^{13}C NMR spectrum ($CDCl_3$) of **1k**

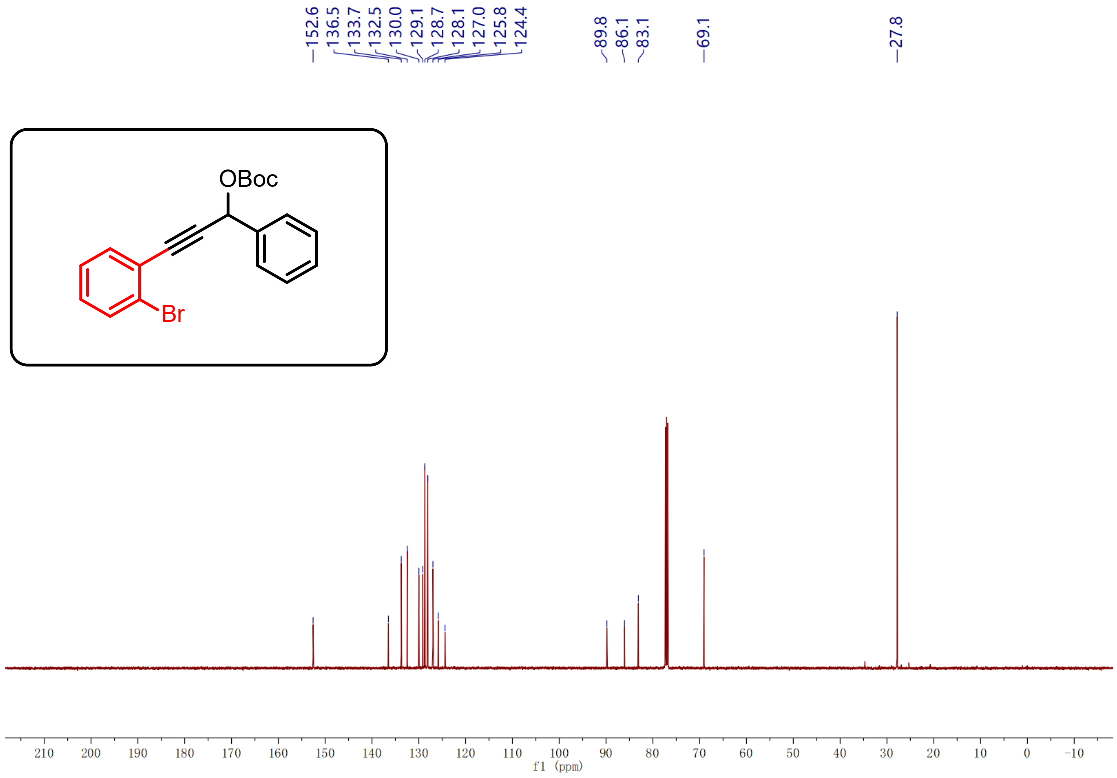

^{1}H NMR spectrum ($CDCl_3$) of **1l**

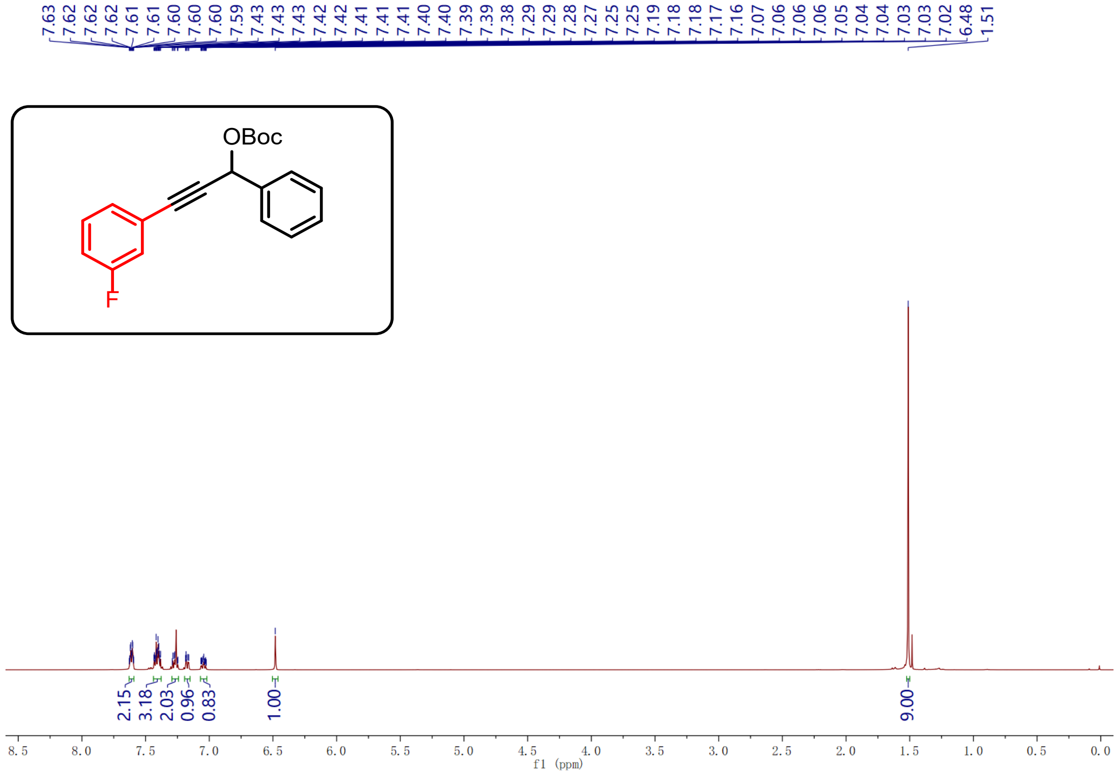

^{13}C NMR spectrum ($CDCl_3$) of **1l**

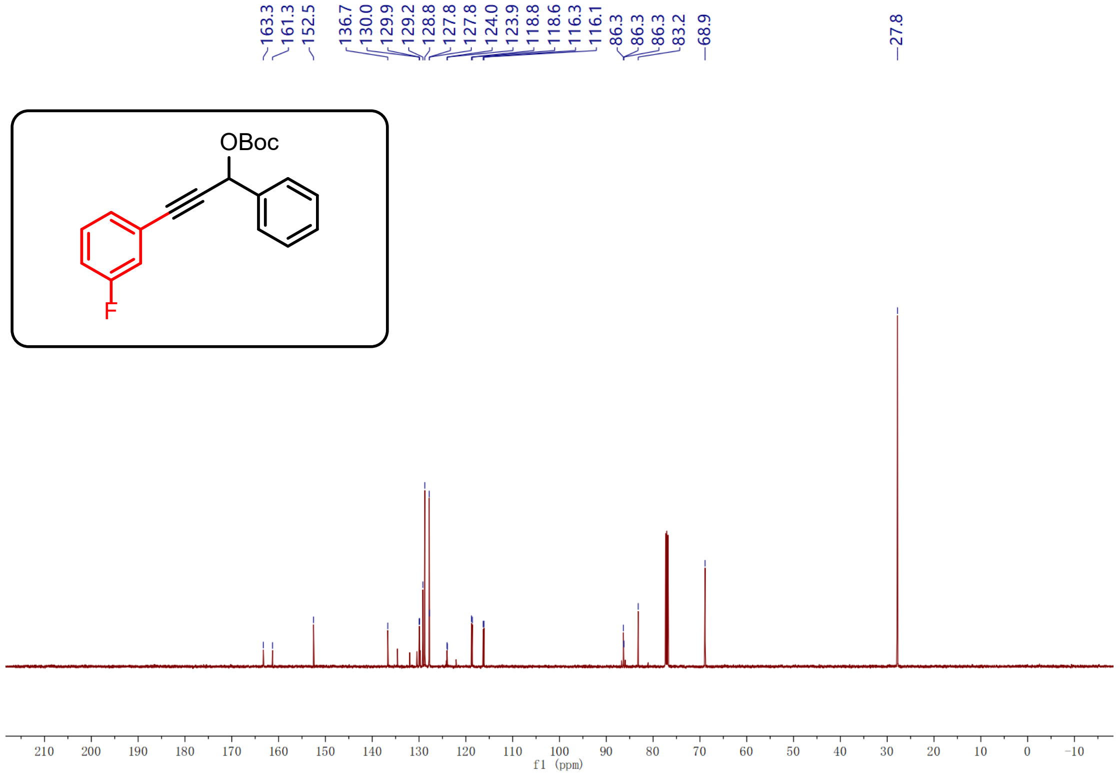

^{19}F NMR spectrum ($CDCl_3$) of **1l**

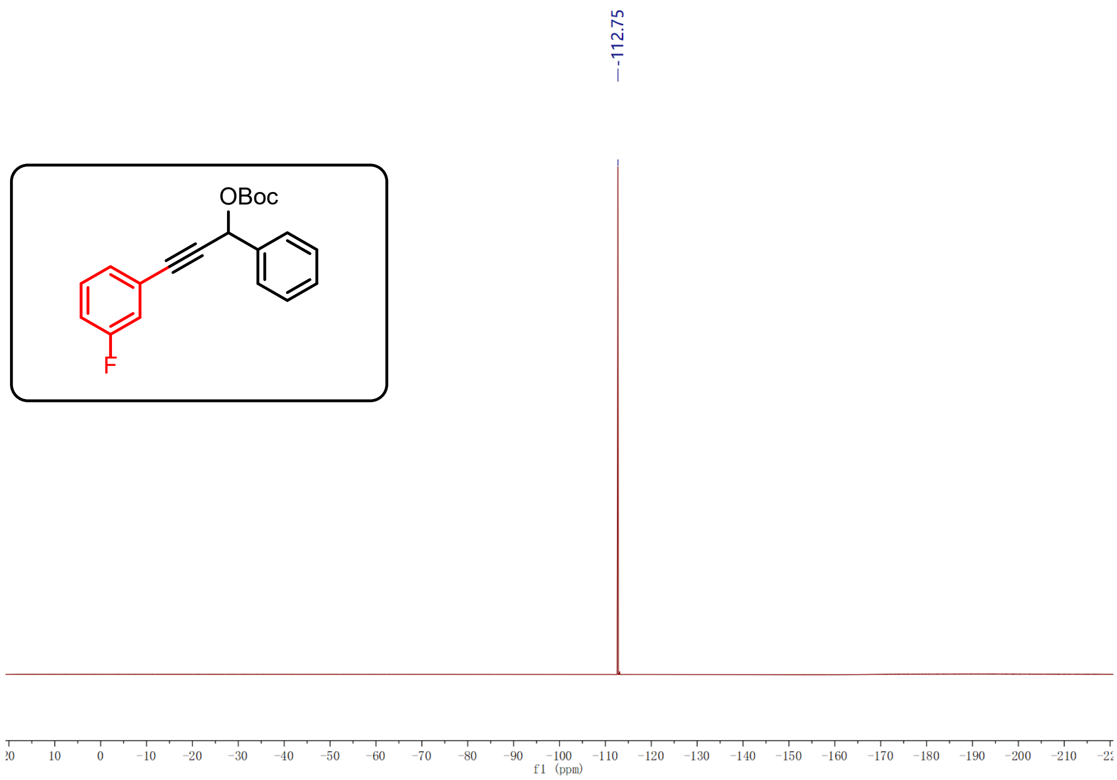

^{1}H NMR spectrum ($CDCl_3$) of **1m**

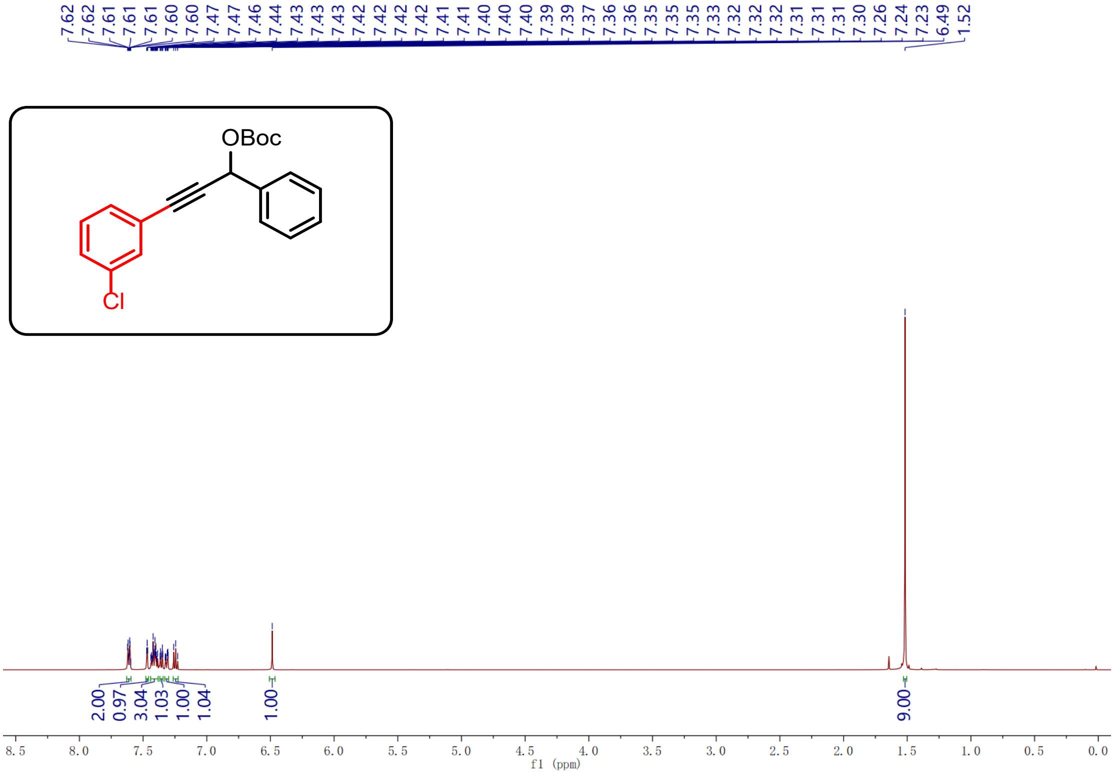

^{13}C NMR spectrum ($CDCl_3$) of **1m**

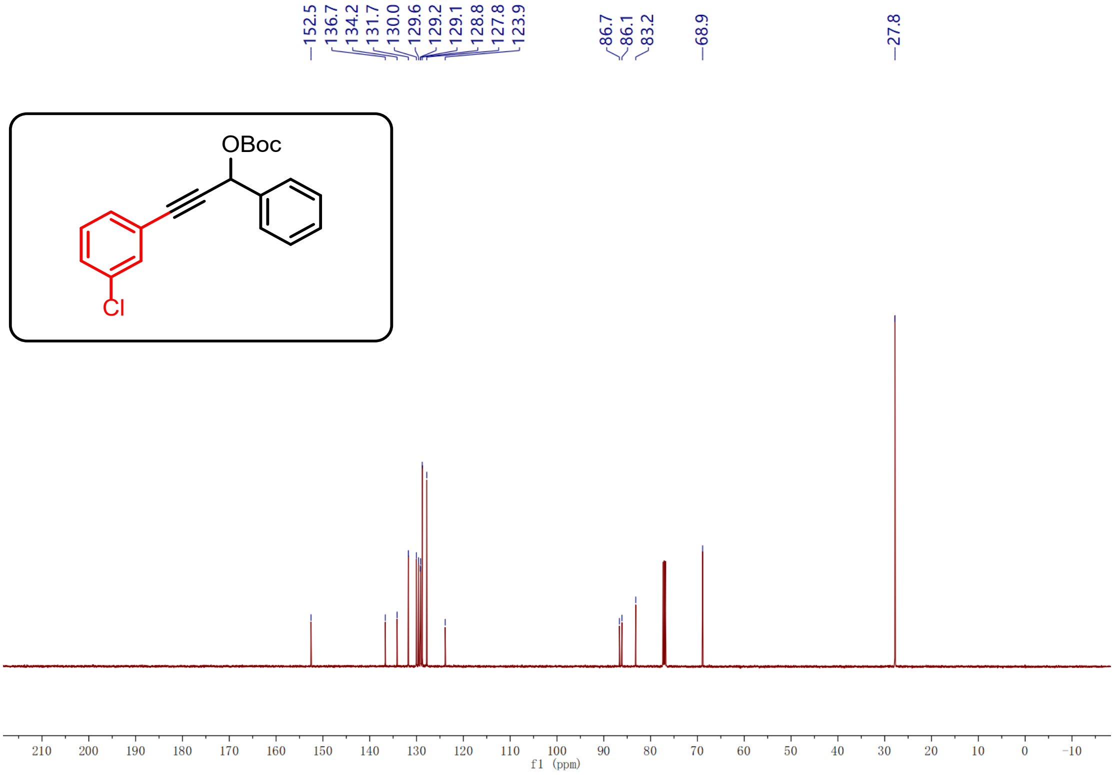

^{1}H NMR spectrum ($CDCl_3$) of **1n**

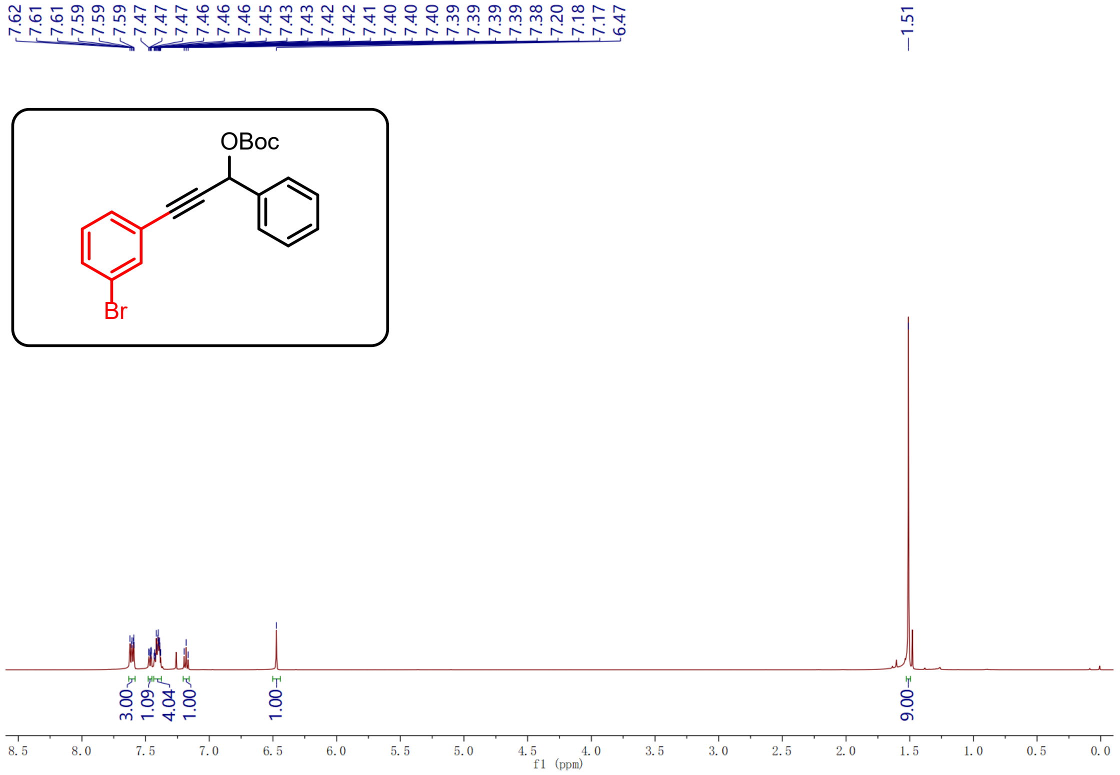

^{13}C NMR spectrum ($CDCl_3$) of **1n**

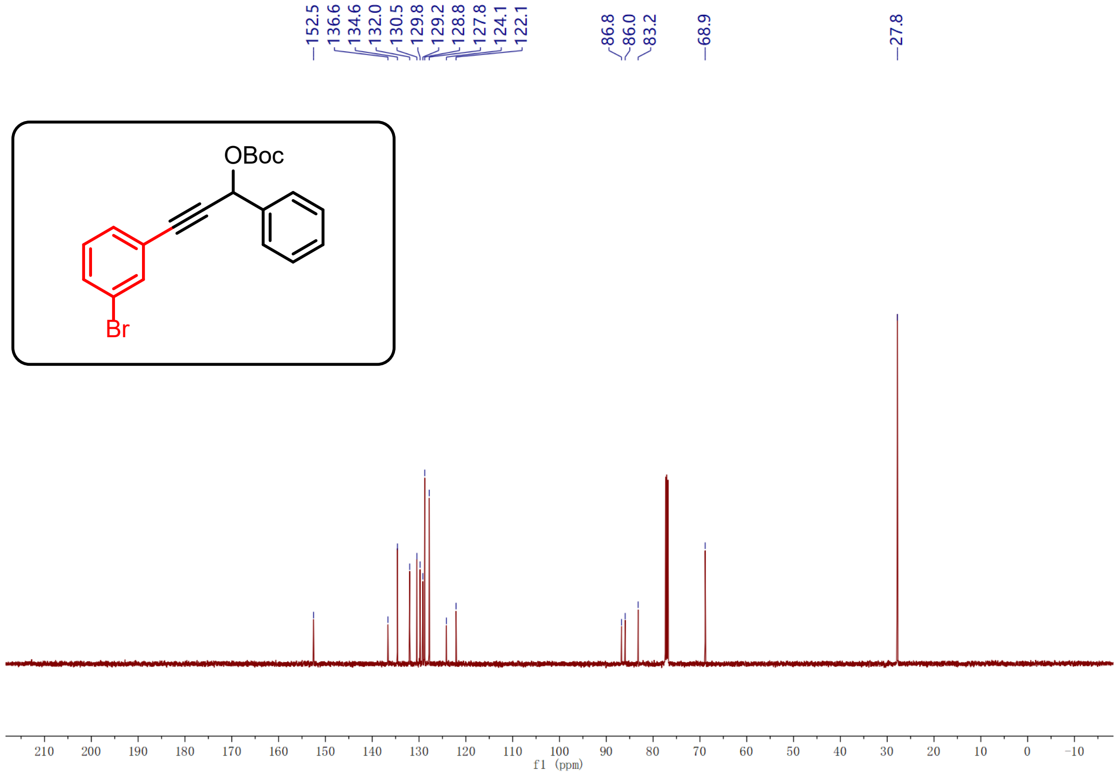

^{1}H NMR spectrum ($CDCl_3$) of **1o**

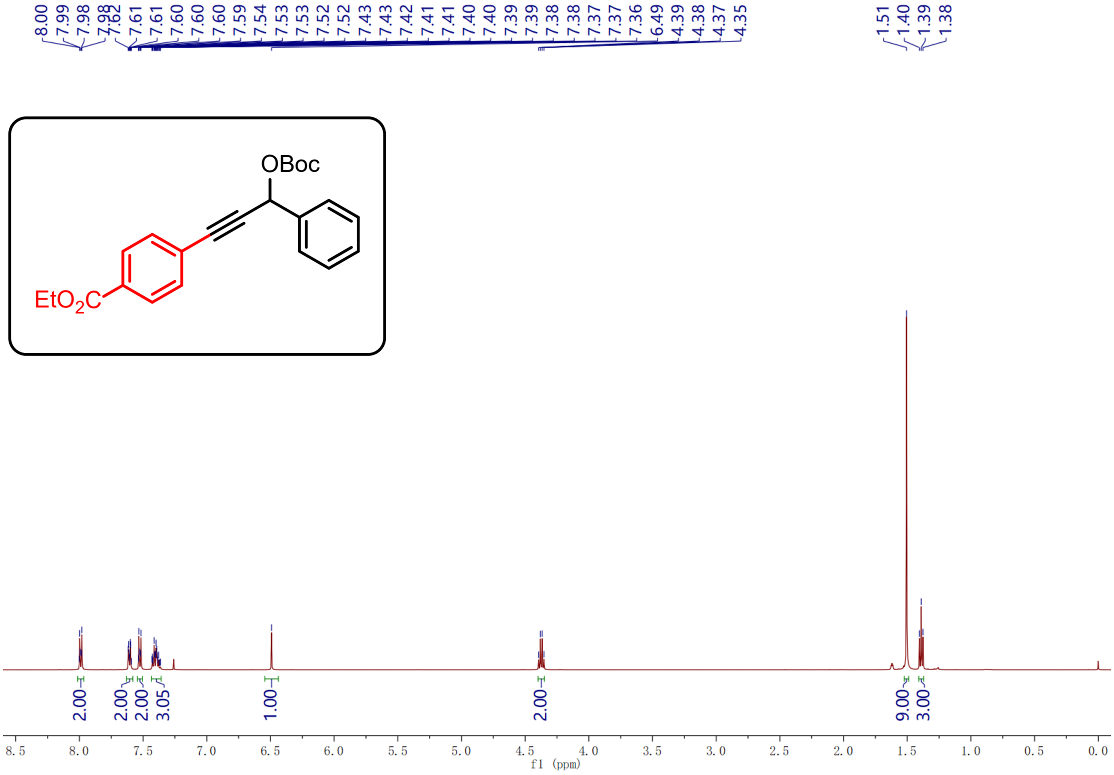

^{13}C NMR spectrum ($CDCl_3$) of **1o**

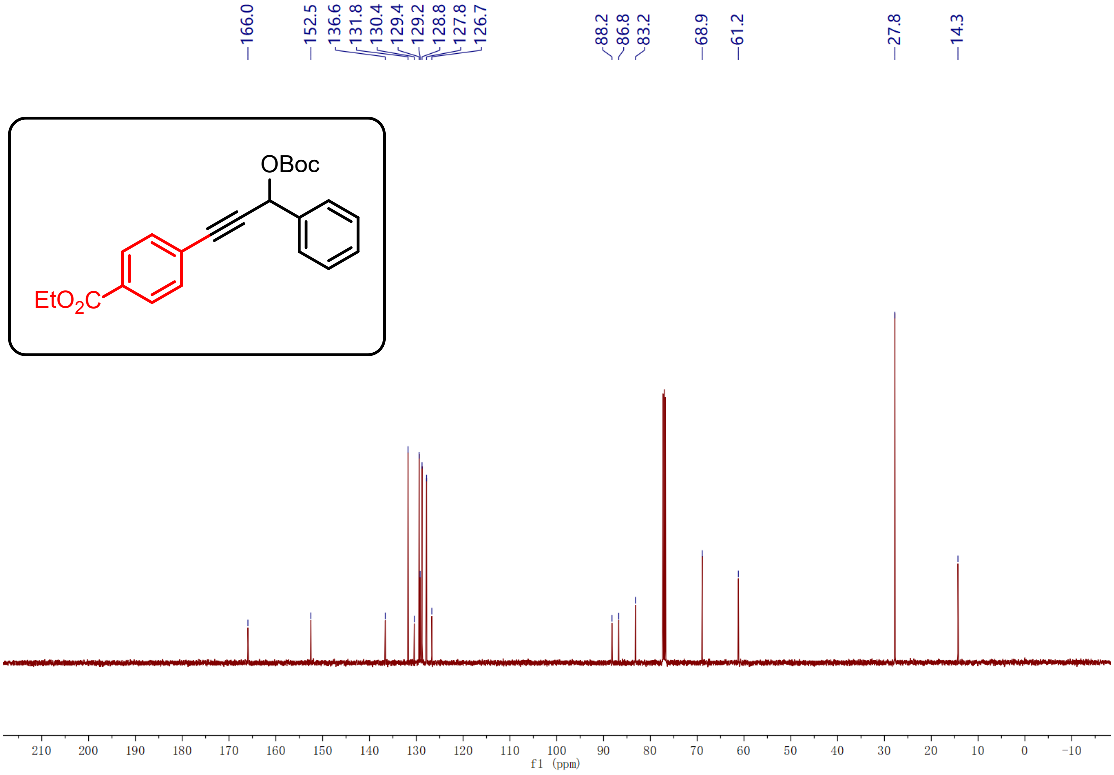

^{1}H NMR spectrum ($CDCl_3$) of **1p**

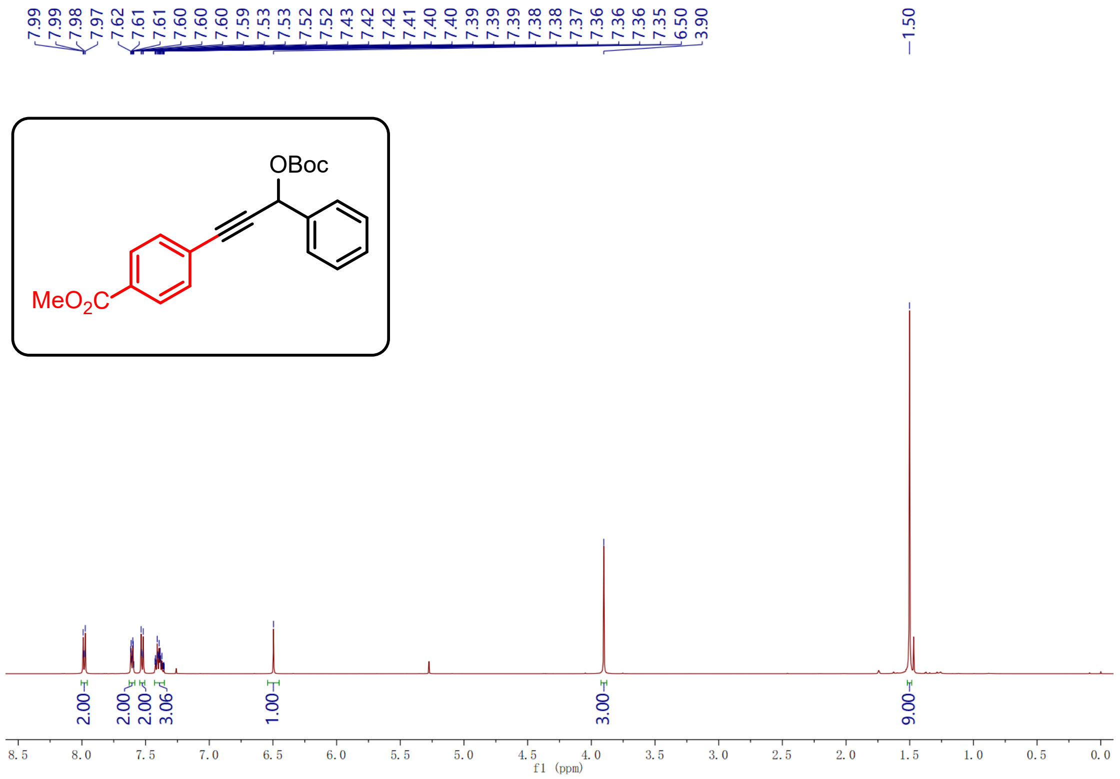

^{13}C NMR spectrum ($CDCl_3$) of **1p**

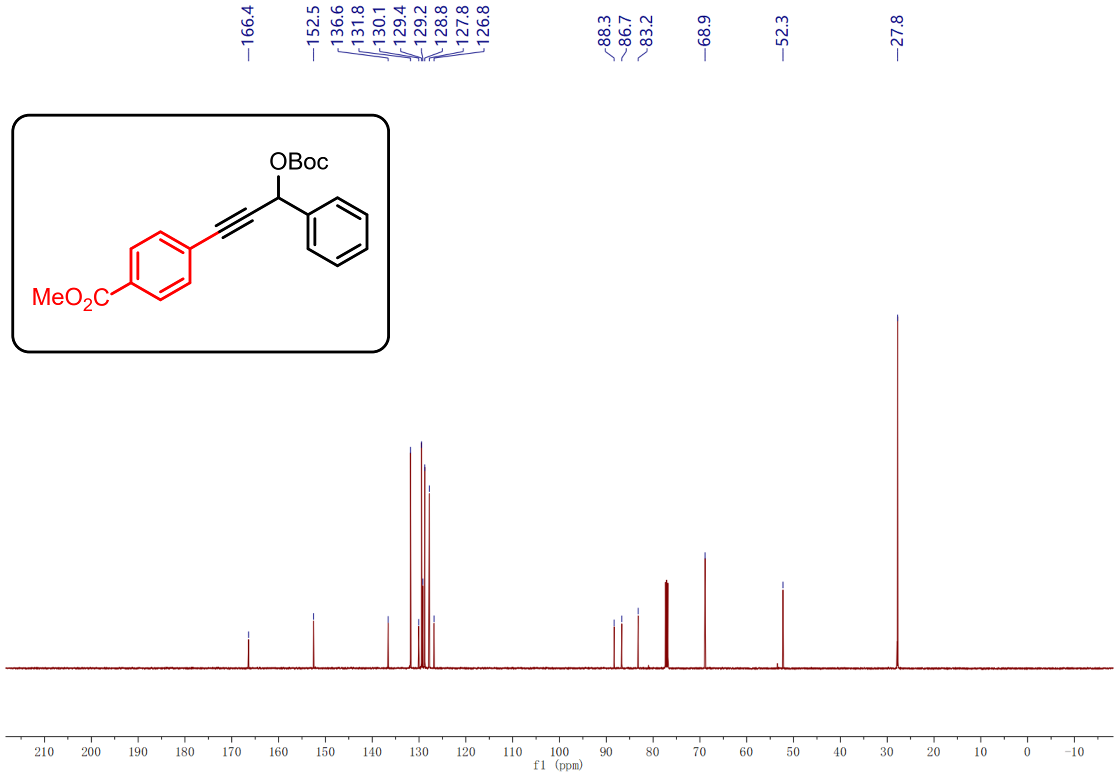

^{1}H NMR spectrum ($CDCl_3$) of **1q**

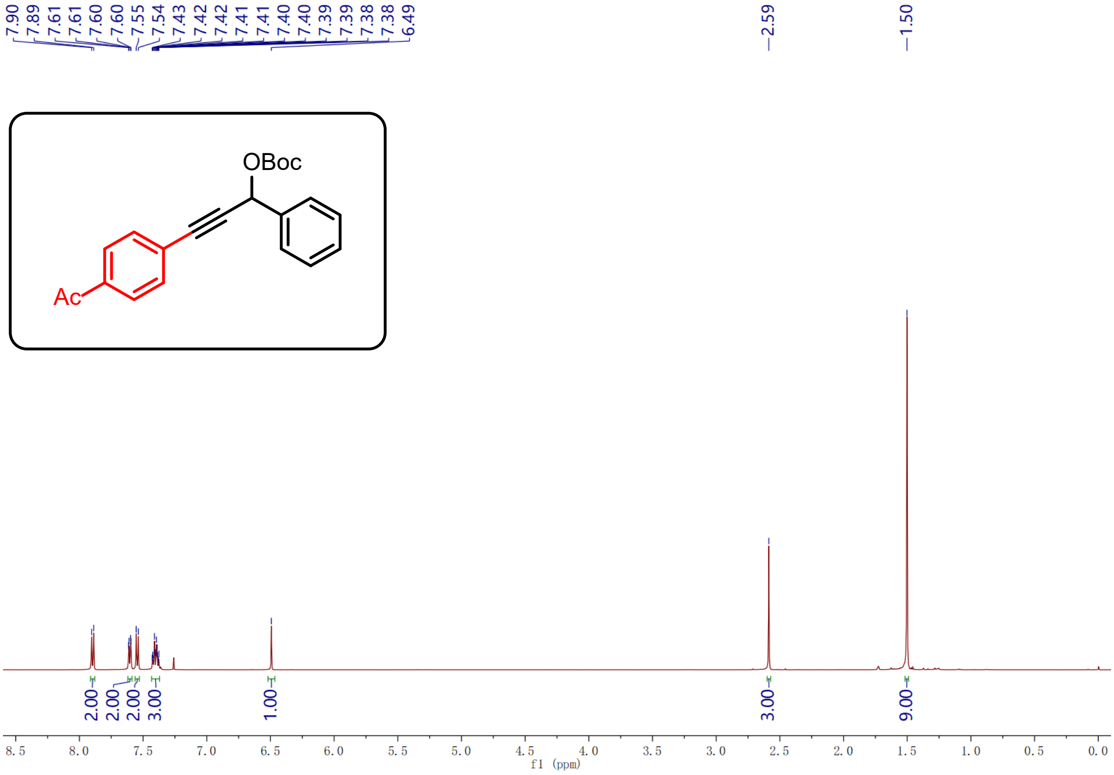

^{13}C NMR spectrum ($CDCl_3$) of **1q**

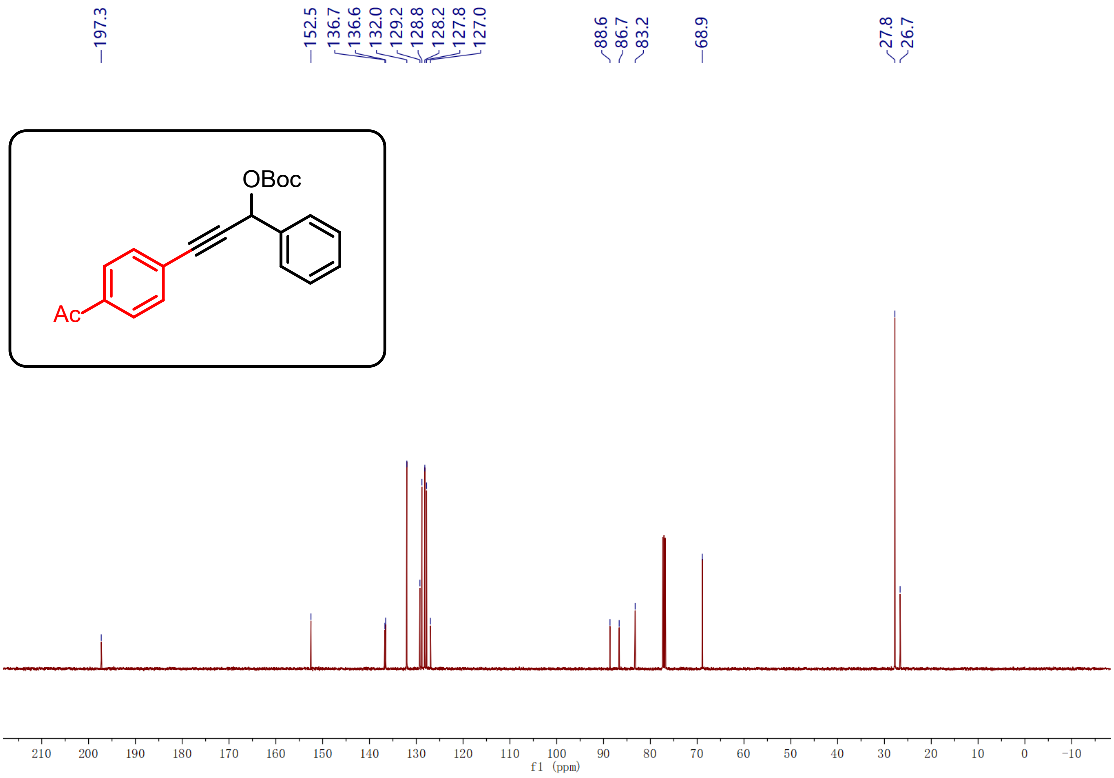

^{1}H NMR spectrum ($CDCl_3$) of **1r**

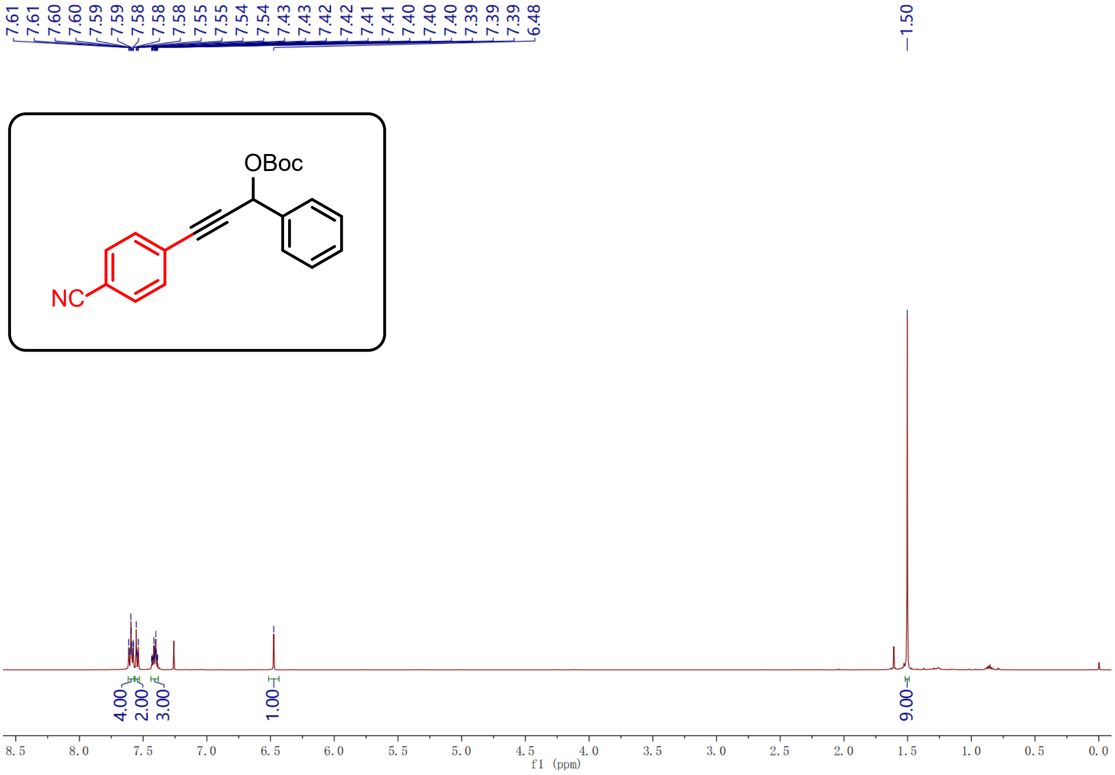

^{13}C NMR spectrum ($CDCl_3$) of **1r**

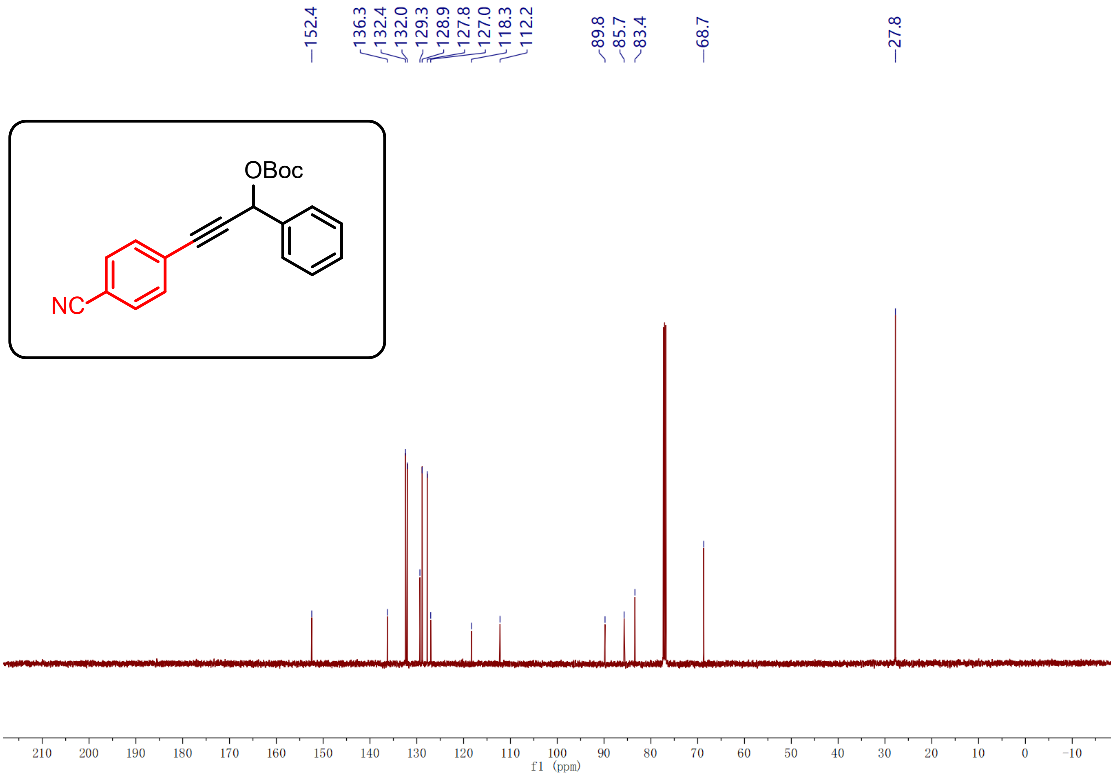

^{1}H NMR spectrum ($CDCl_3$) of **1s**

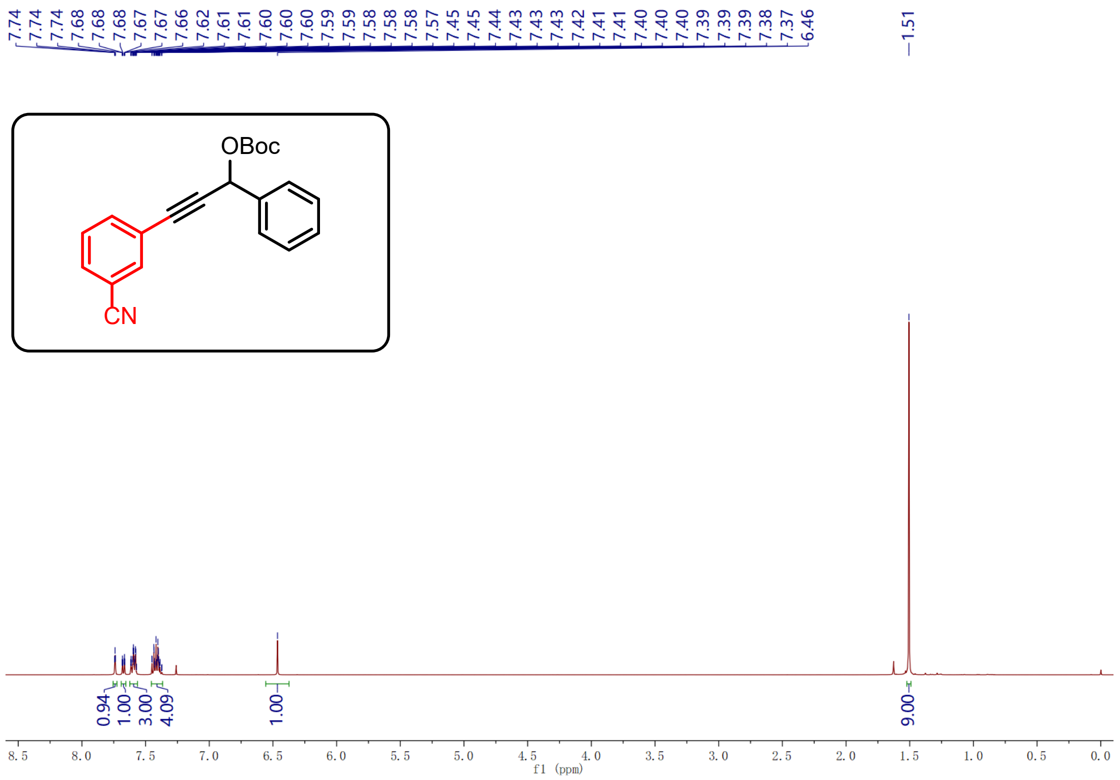

^{13}C NMR spectrum ($CDCl_3$) of **1s**

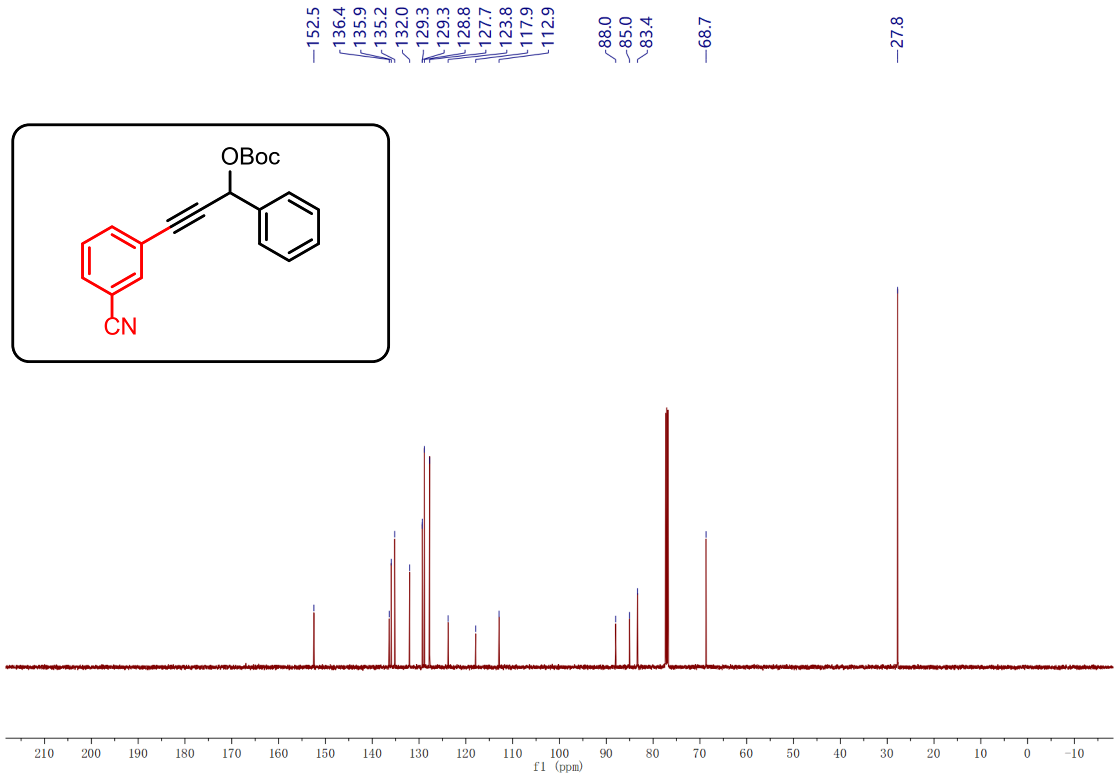

^{1}H NMR spectrum ($CDCl_3$) of **1t**

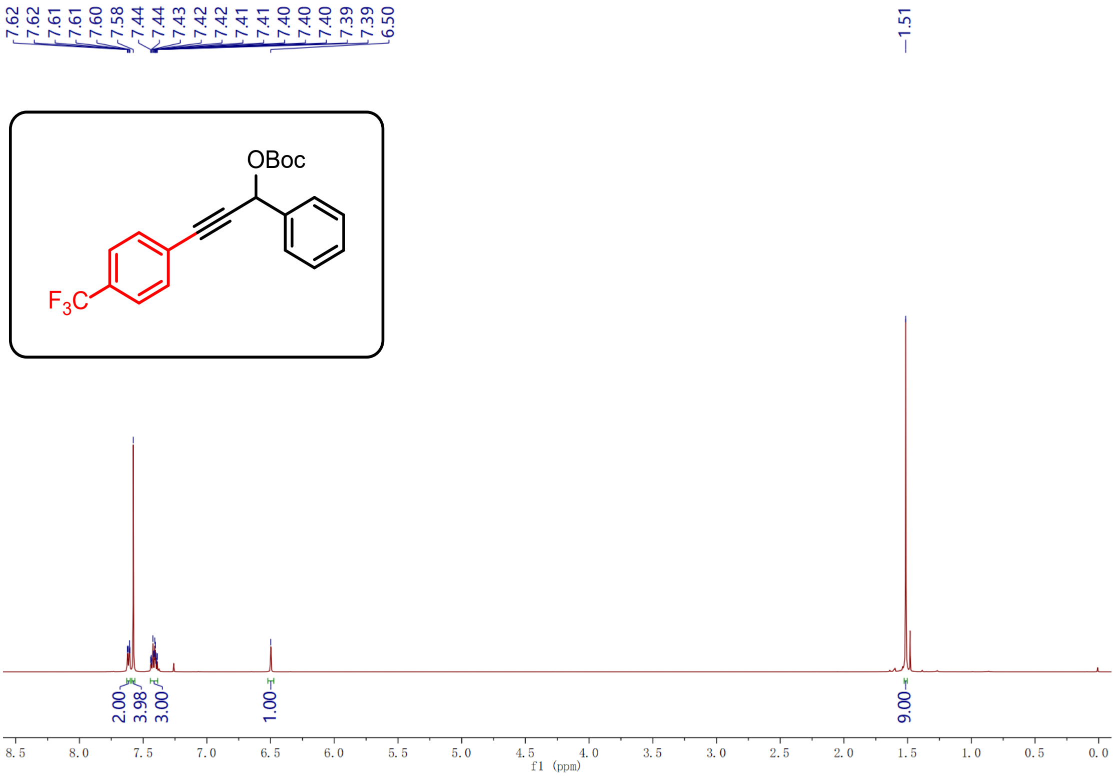

^{13}C NMR spectrum ($CDCl_3$) of **1t**

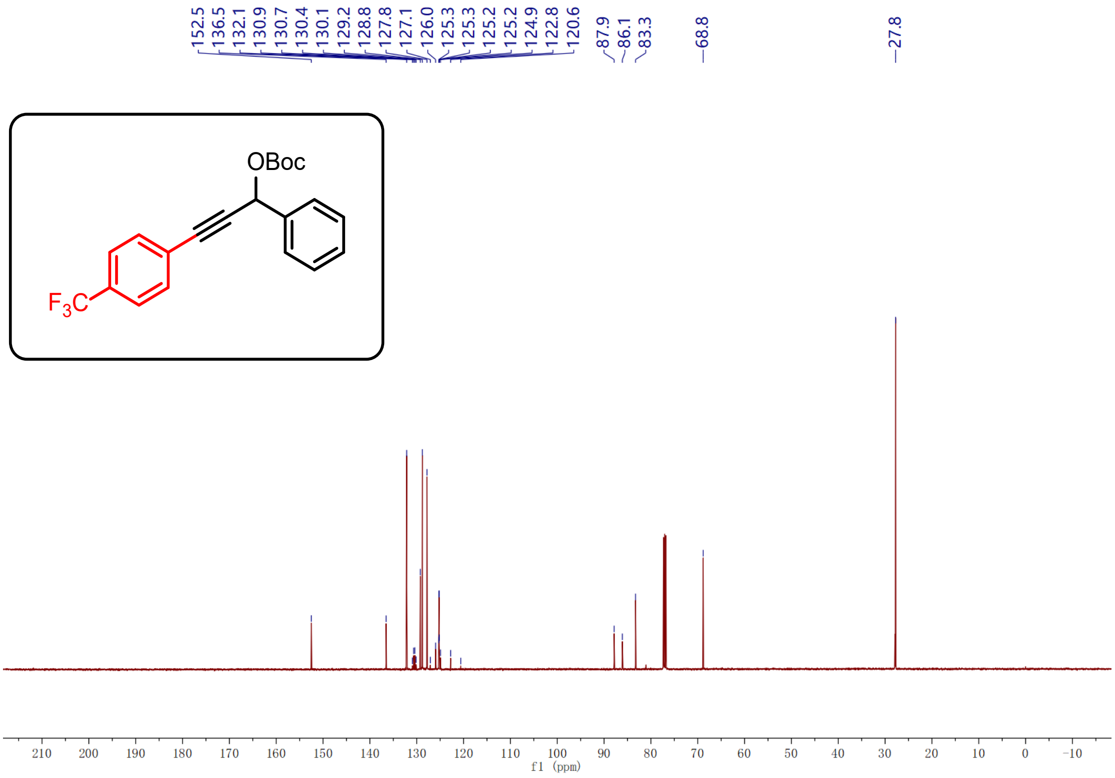

^{19}F NMR spectrum ($CDCl_3$) of **1t**

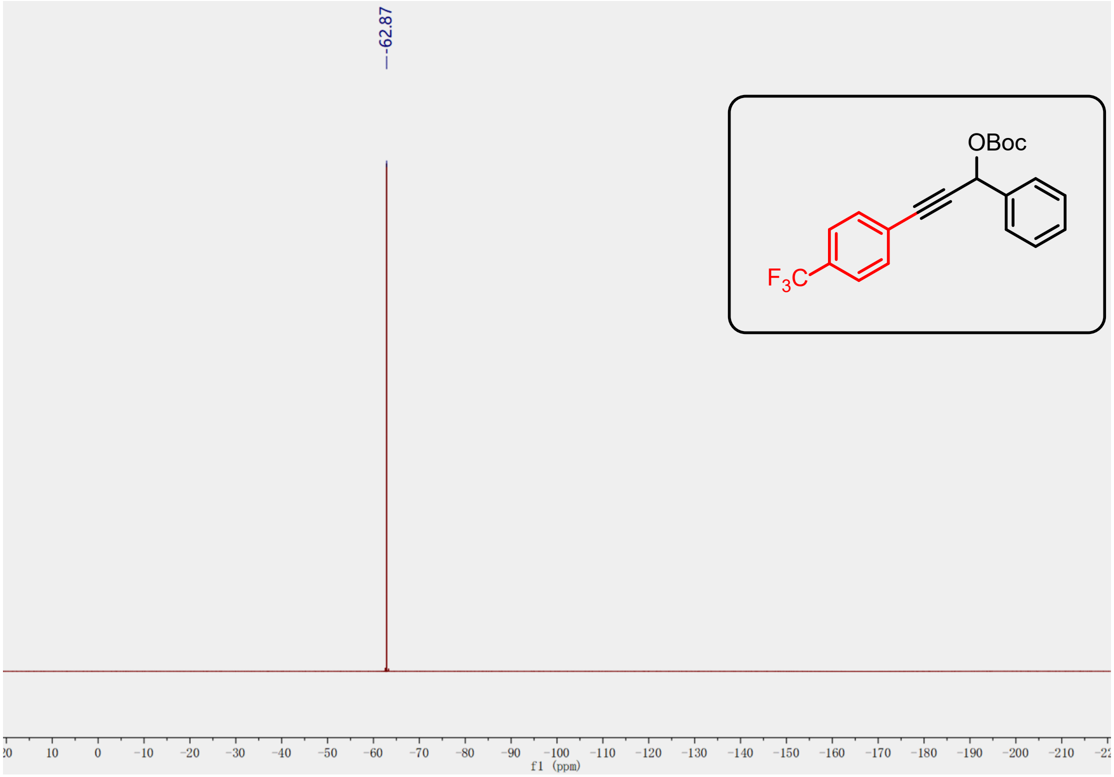

^{1}H NMR spectrum ($CDCl_3$) of **1u**

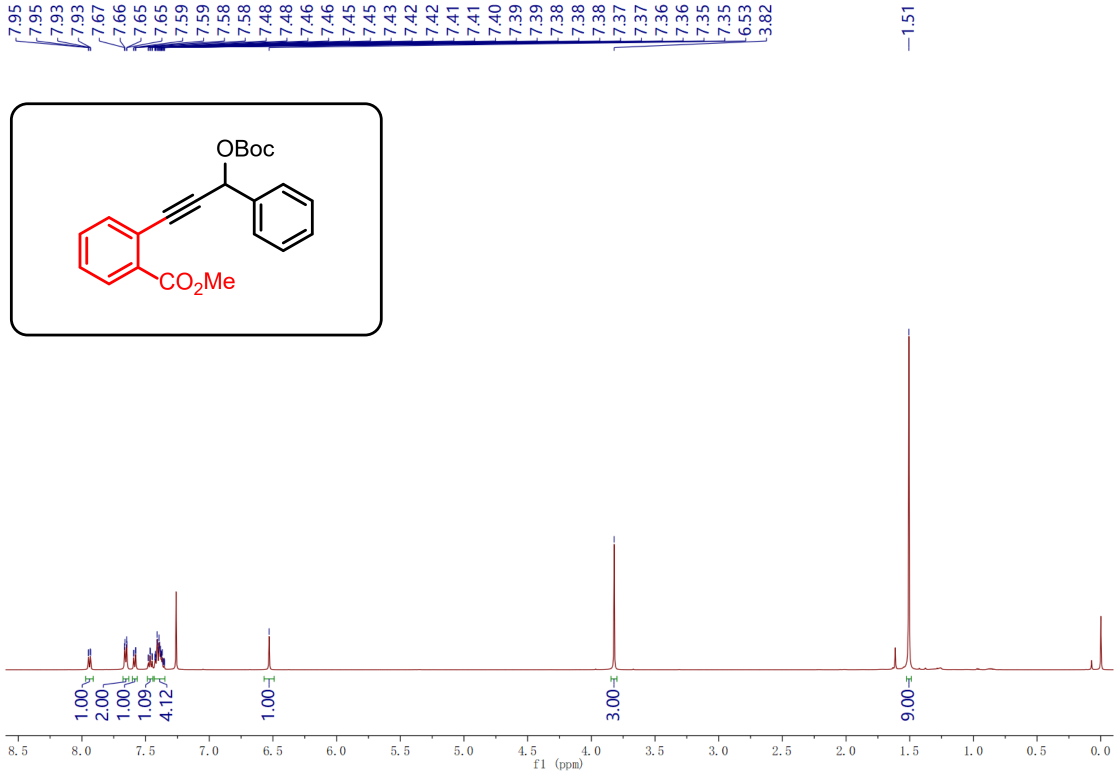

^{13}C NMR spectrum ($CDCl_3$) of **1u**

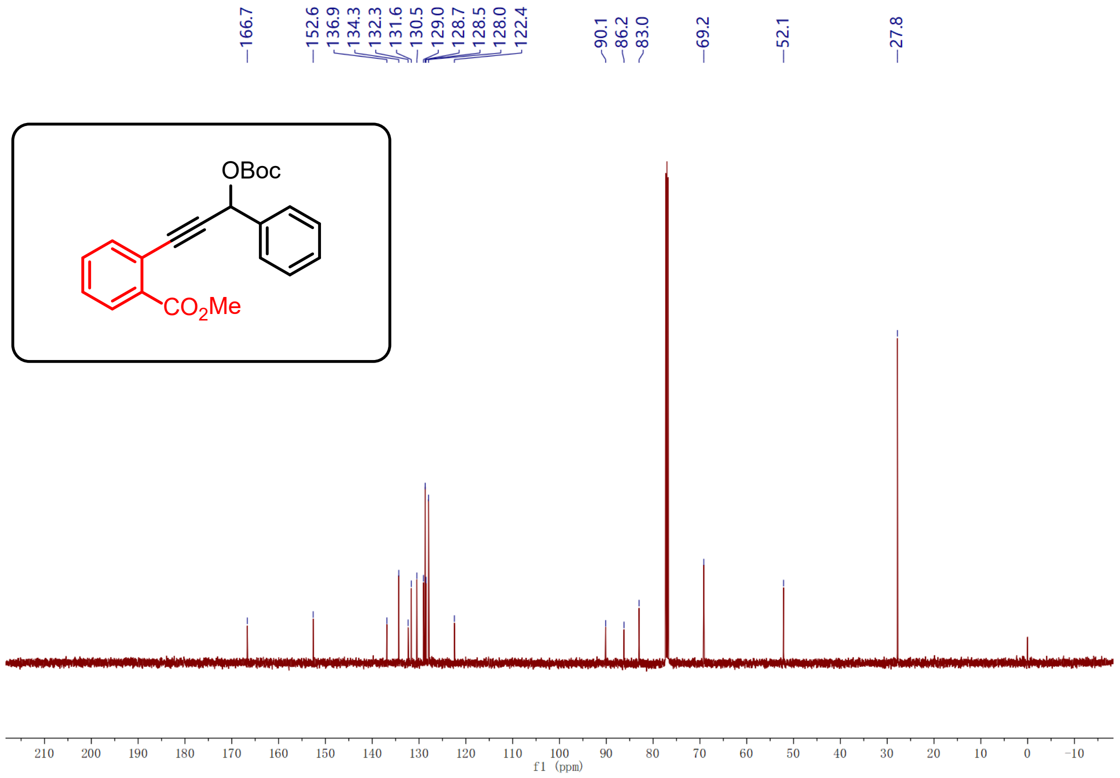

^{1}H NMR spectrum ($CDCl_3$) of **1v**

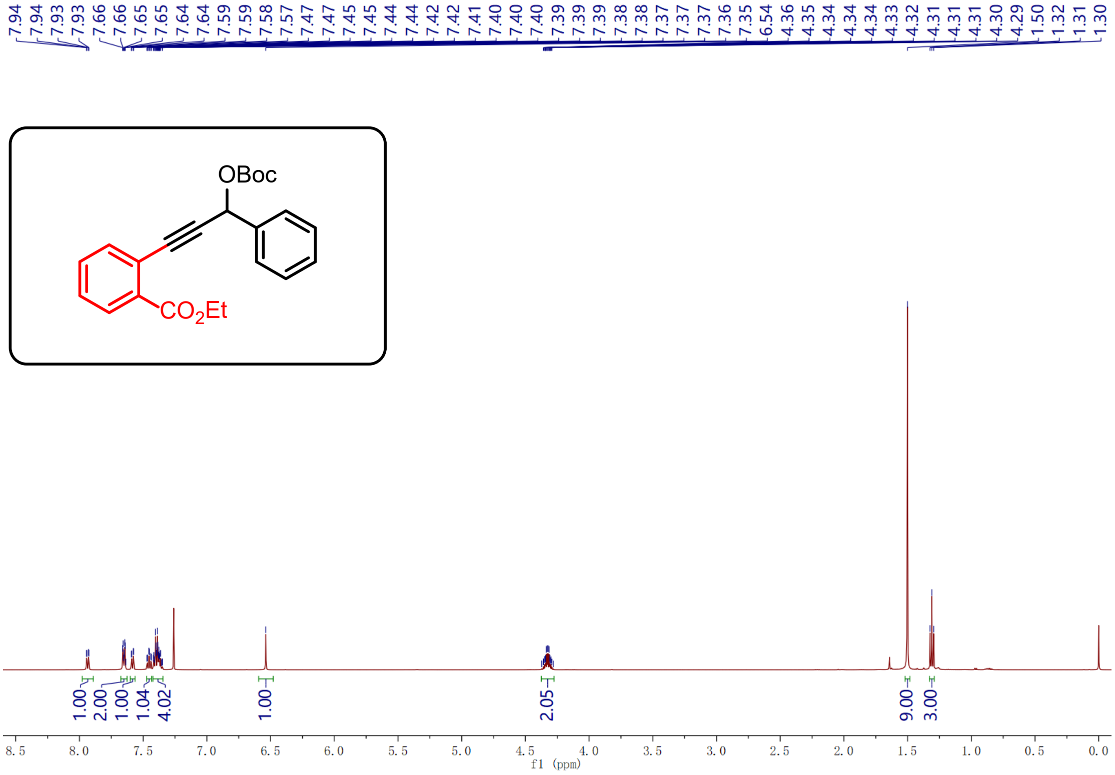

^{13}C NMR spectrum ($CDCl_3$) of **1v**

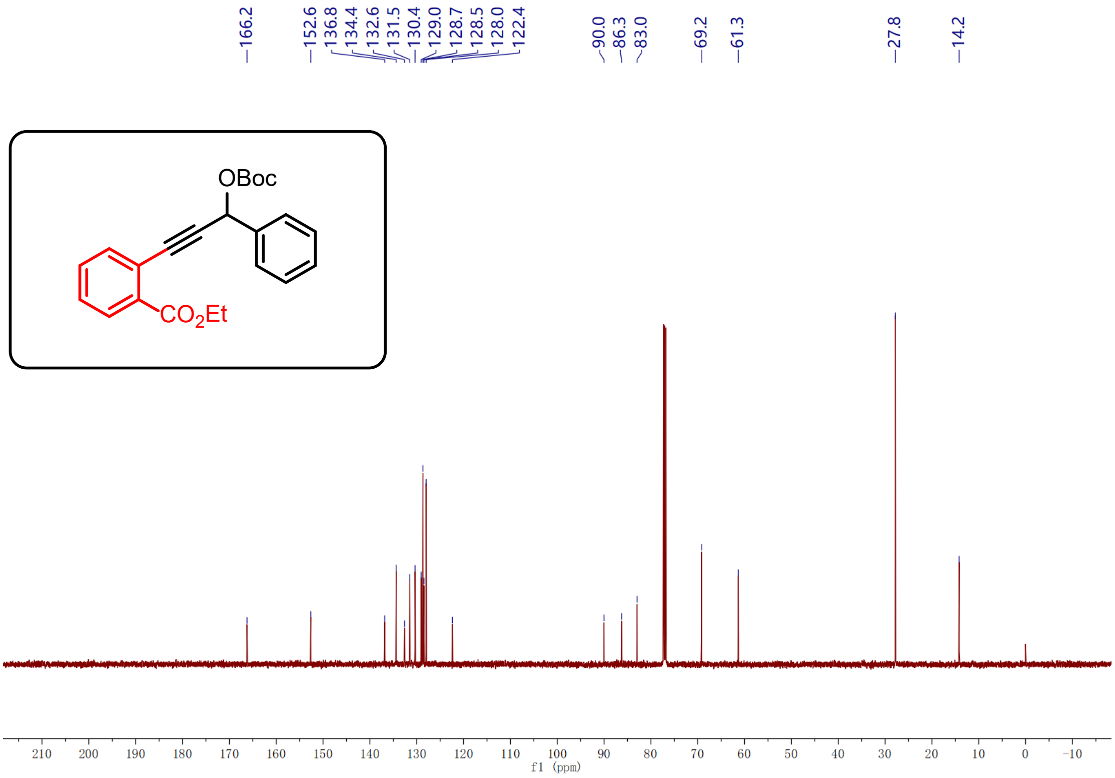

^{1}H NMR spectrum ($CDCl_3$) of **1w**

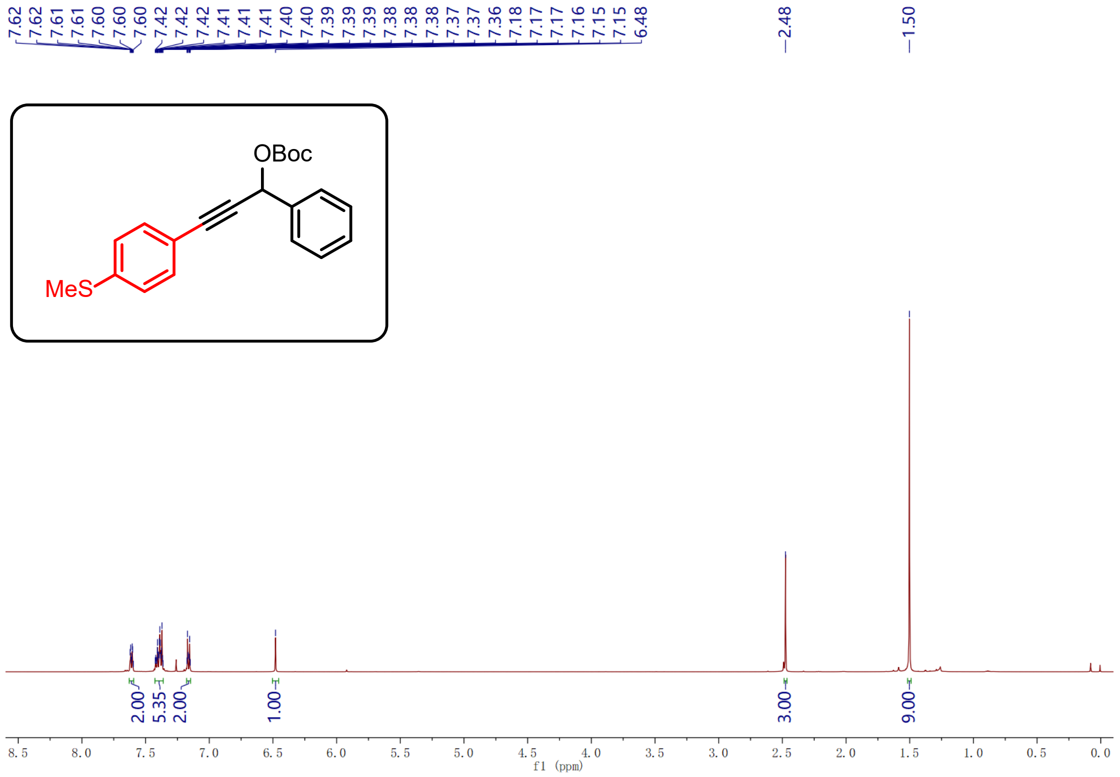

^{13}C NMR spectrum ($CDCl_3$) of **1w**

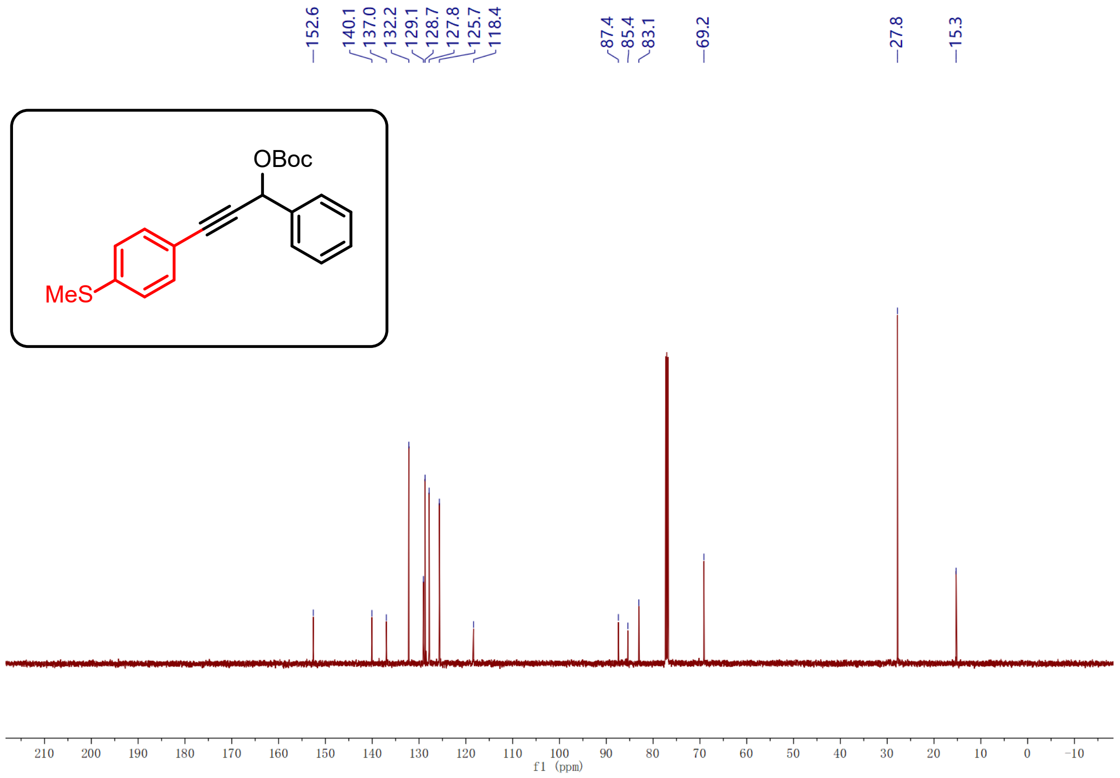

^{1}H NMR spectrum ($CDCl_3$) of **1x**

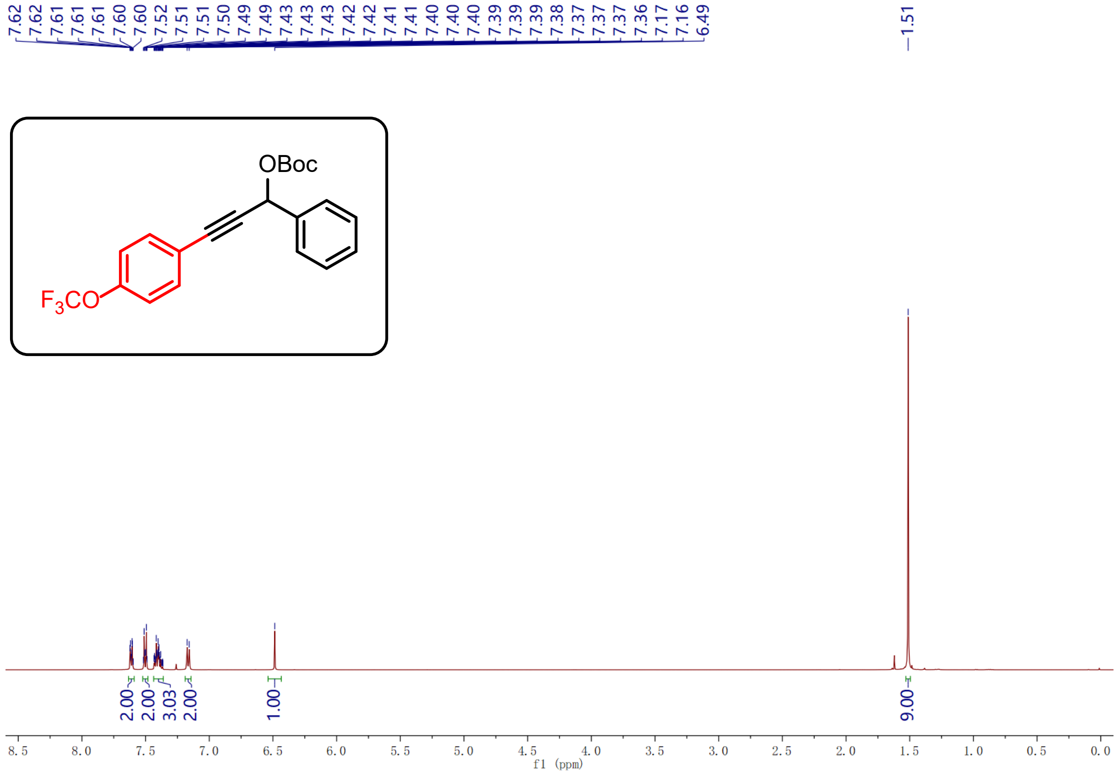

^{13}C NMR spectrum ($CDCl_3$) of **1x**

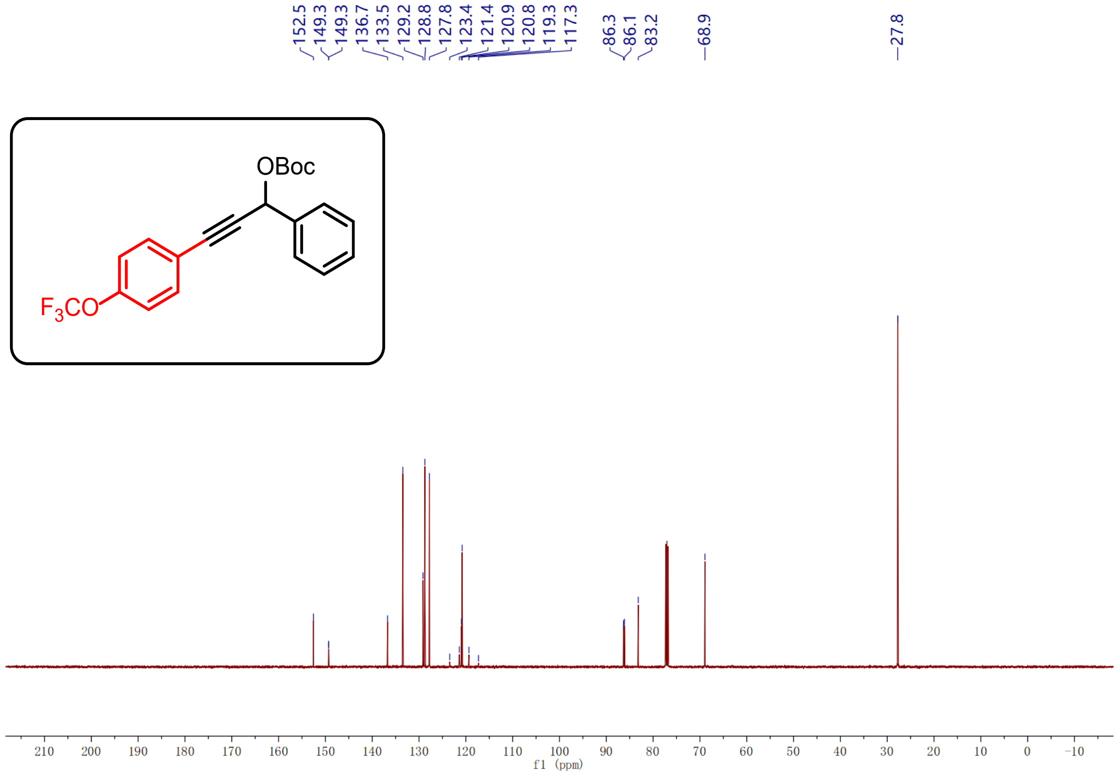

^{19}F NMR spectrum ($CDCl_3$) of **1x**

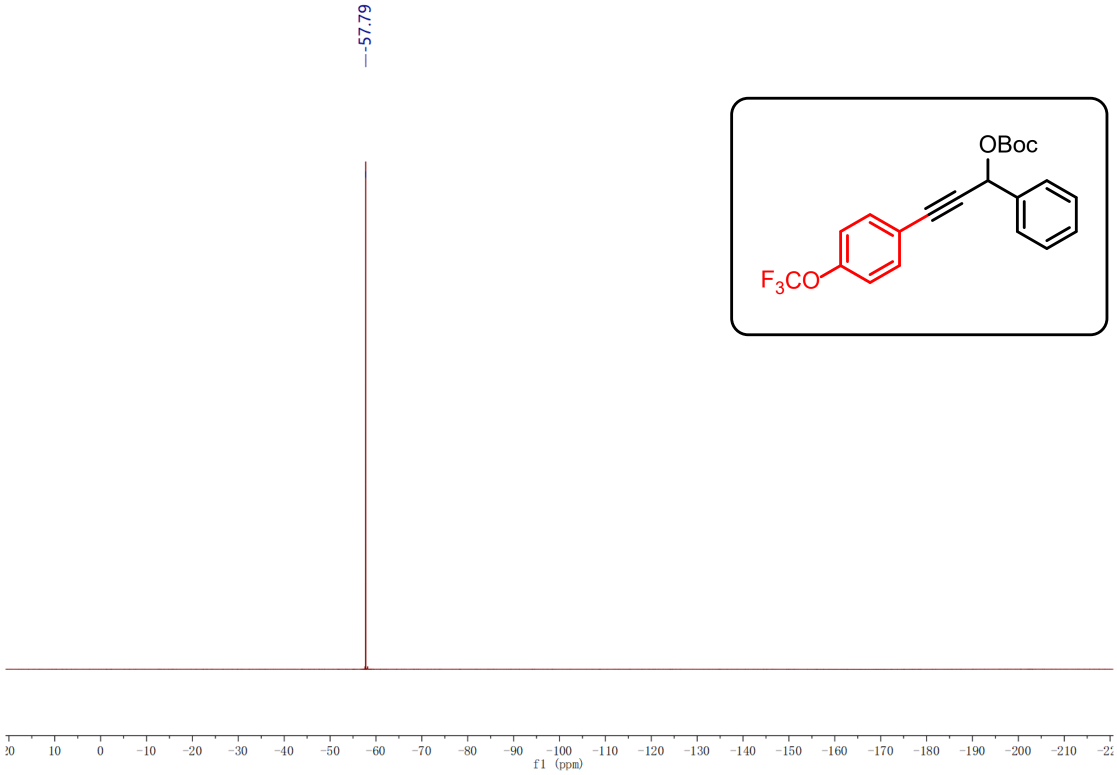

^{1}H NMR spectrum ($CDCl_3$) of **1y**

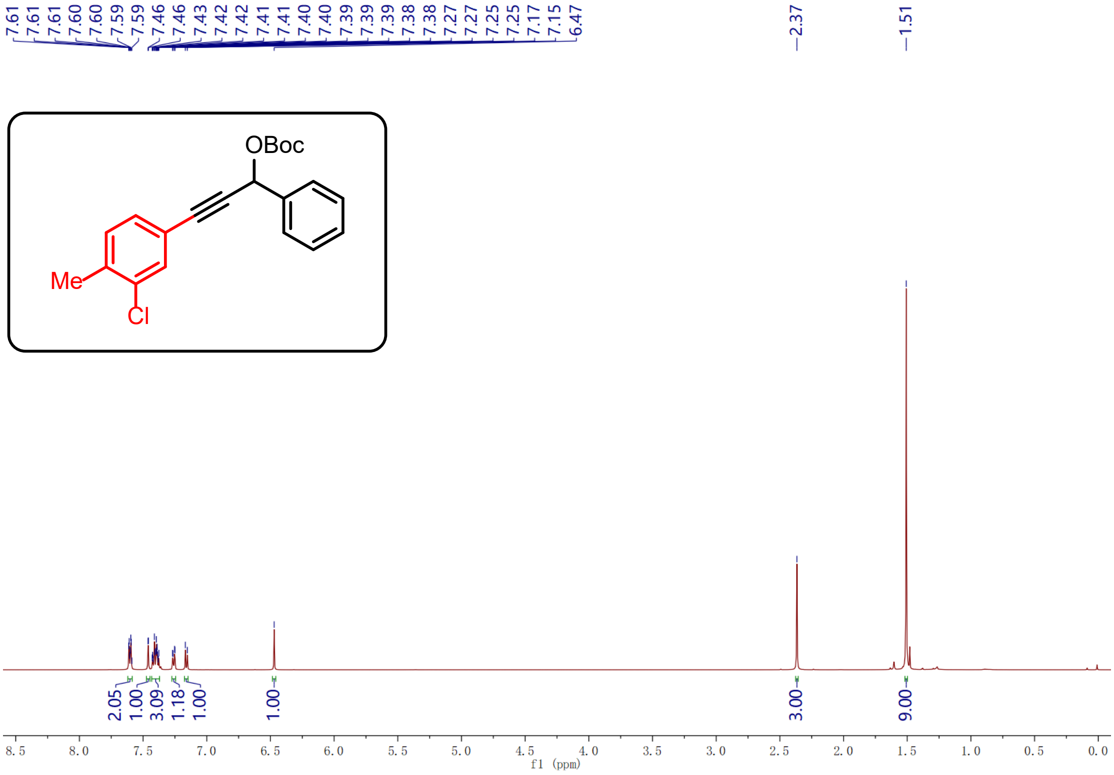

^{13}C NMR spectrum ($CDCl_3$) of **1y**

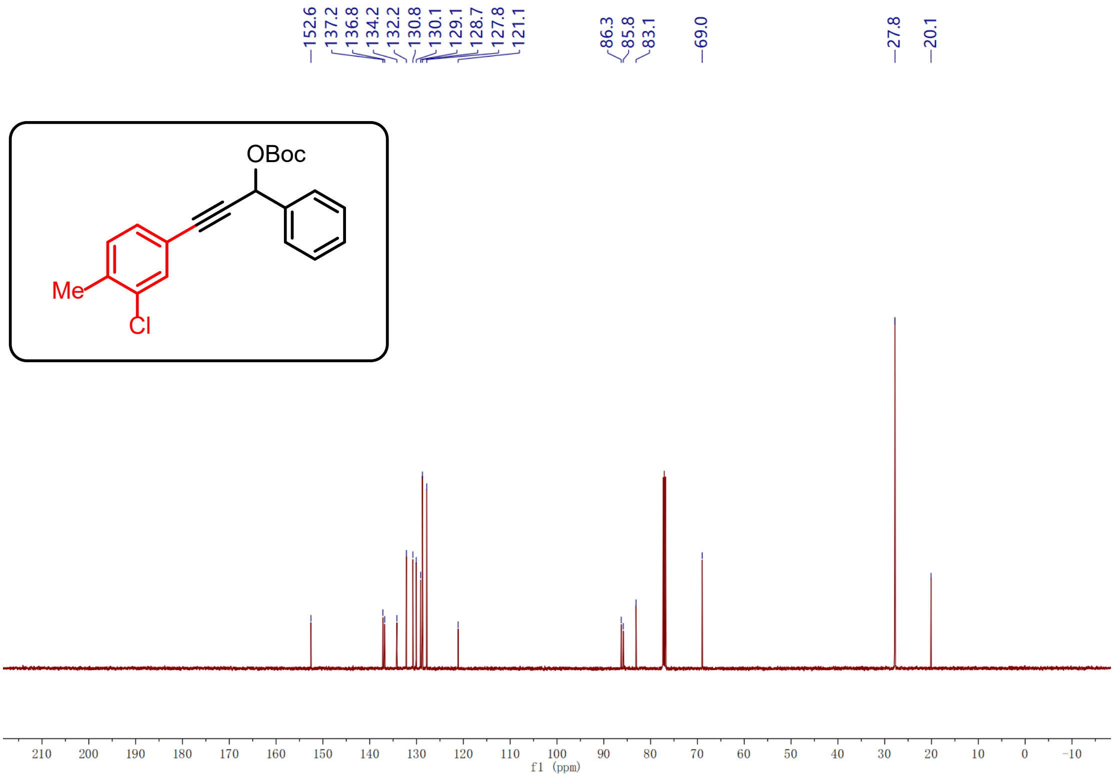

^{1}H NMR spectrum ($CDCl_3$) of **1z**

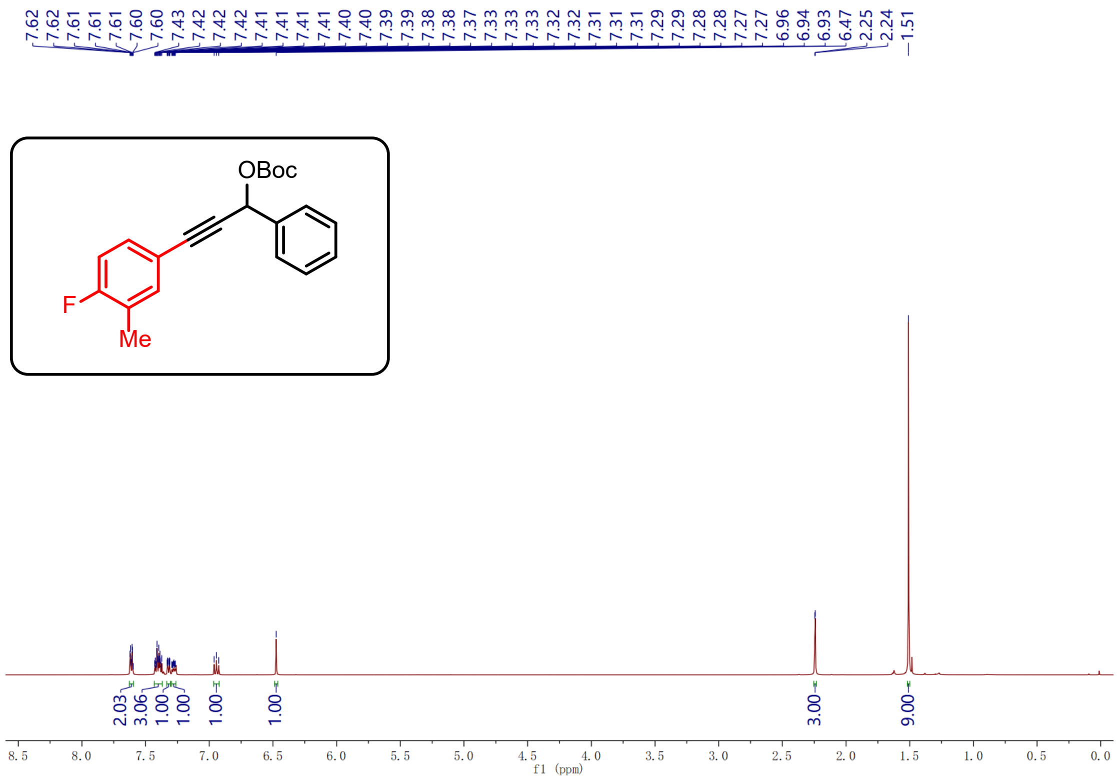

^{13}C NMR spectrum ($CDCl_3$) of **1z**

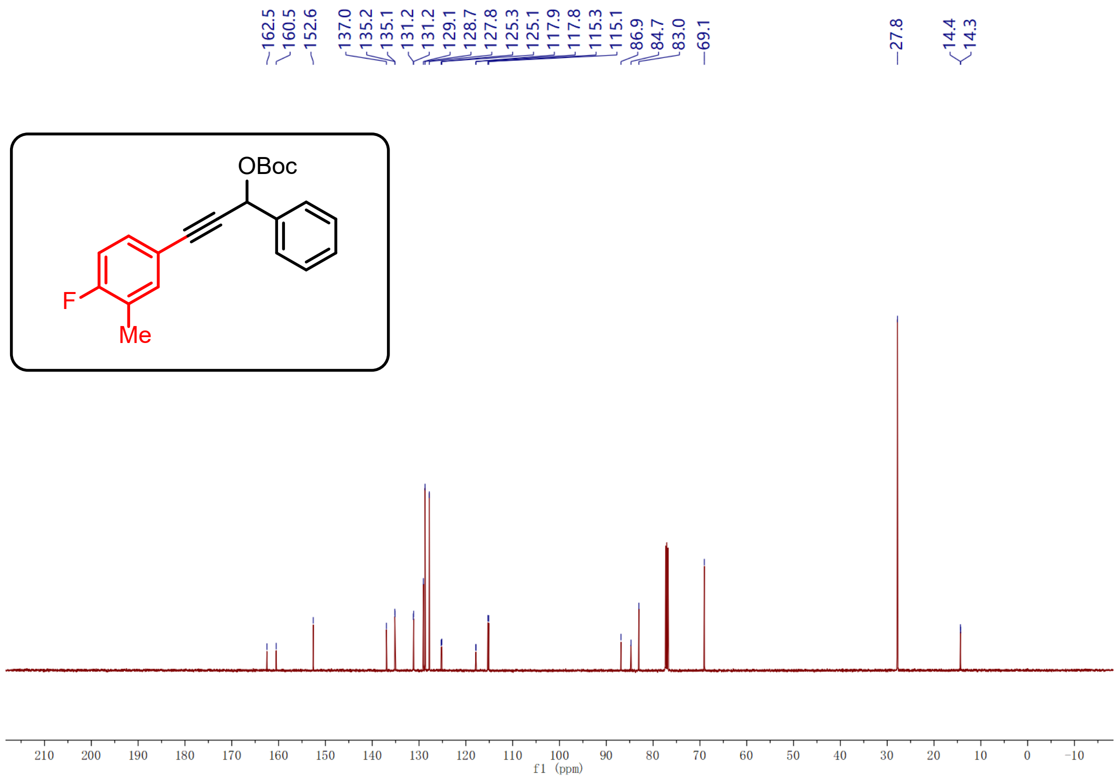

^{19}F NMR spectrum ($CDCl_3$) of **1z**

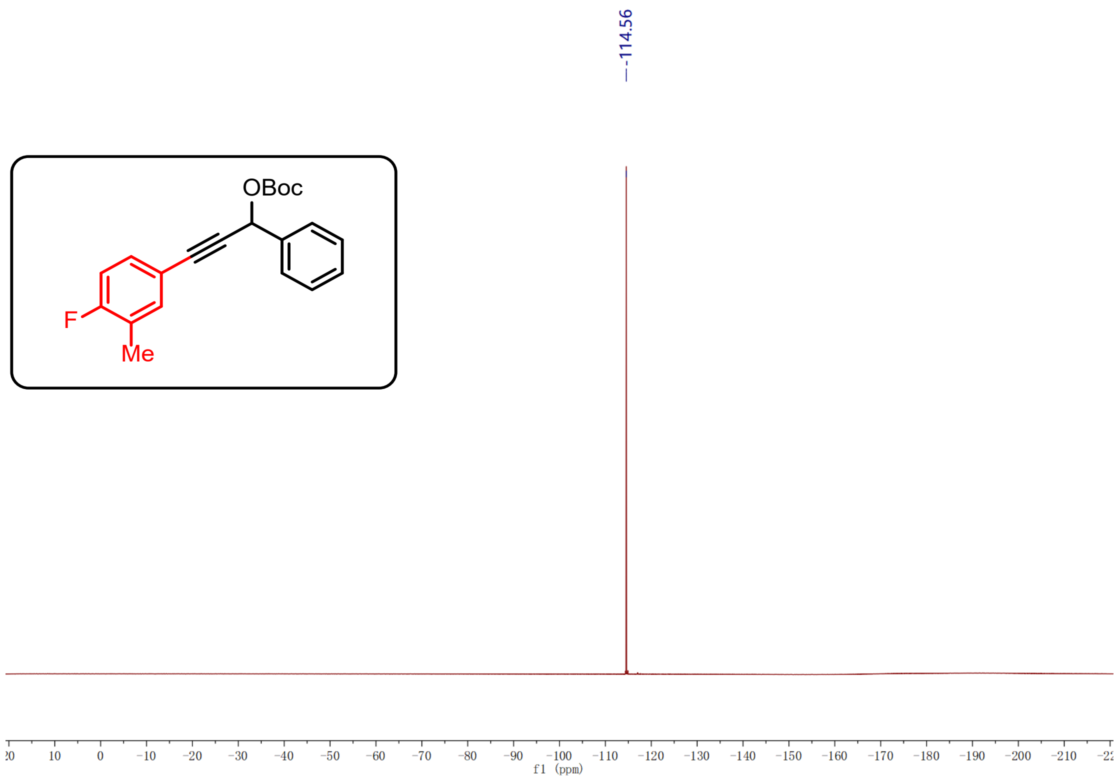

^{1}H NMR spectrum ($CDCl_3$) of **1aa**

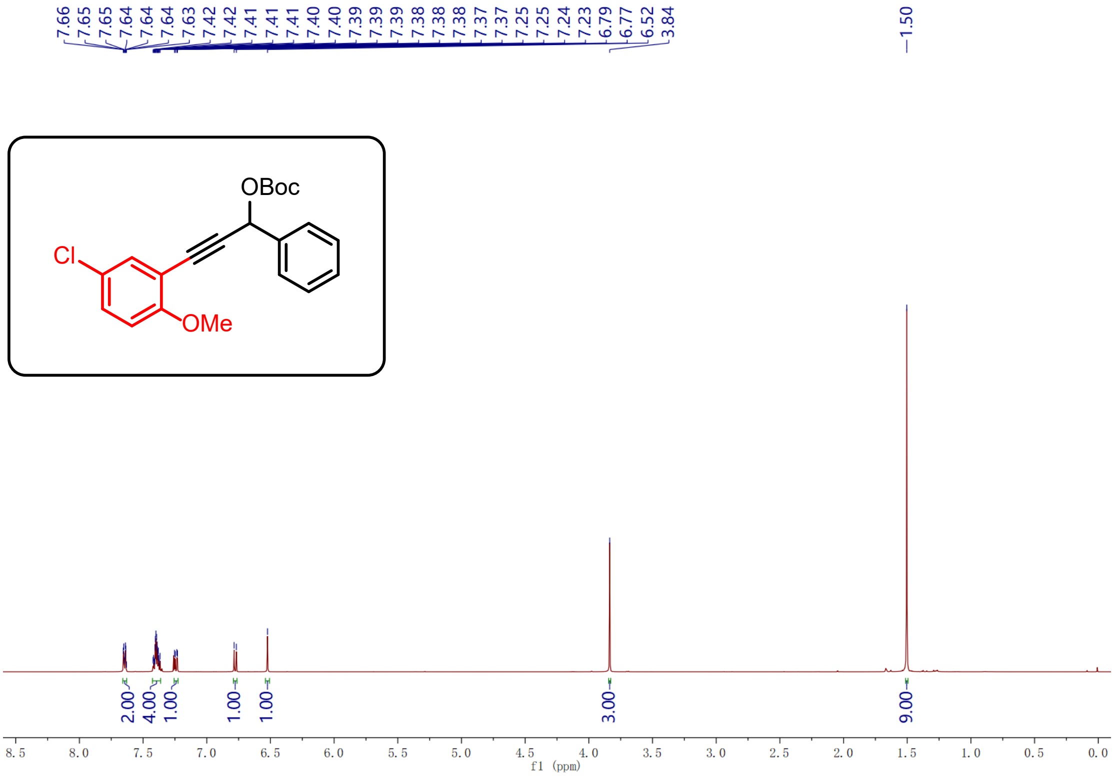

^{13}C NMR spectrum ($CDCl_3$) of **1aa**

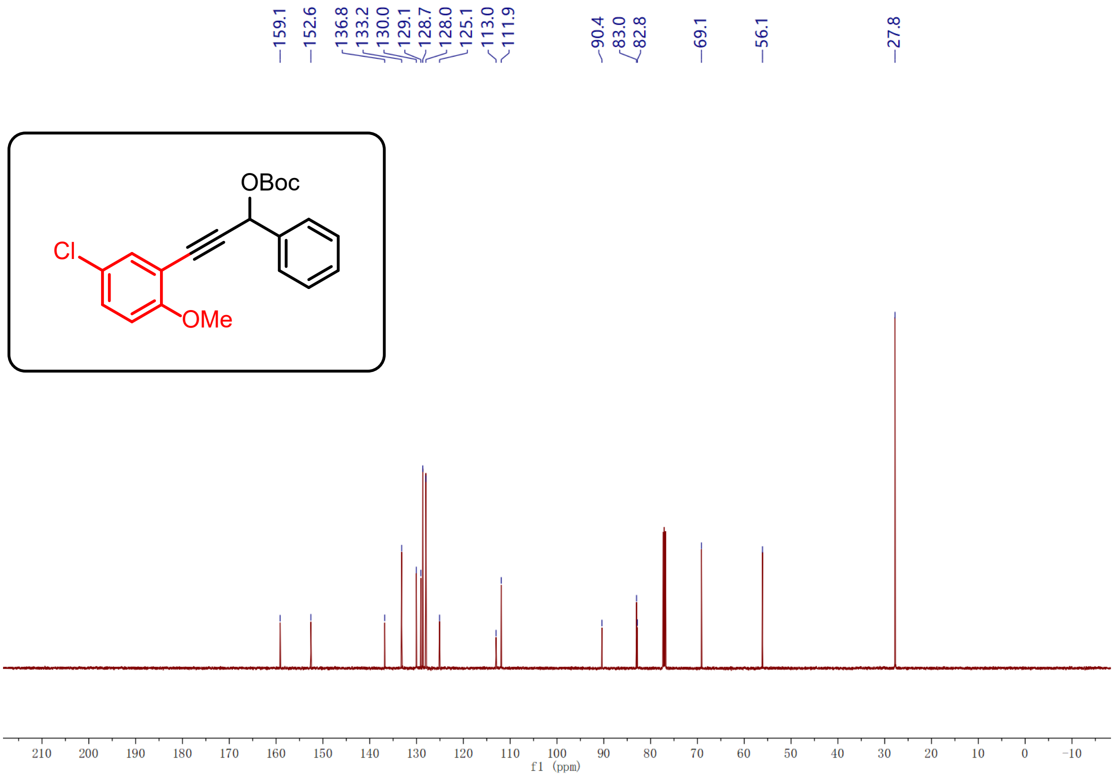

^{1}H NMR spectrum ($CDCl_3$) of **1ab**

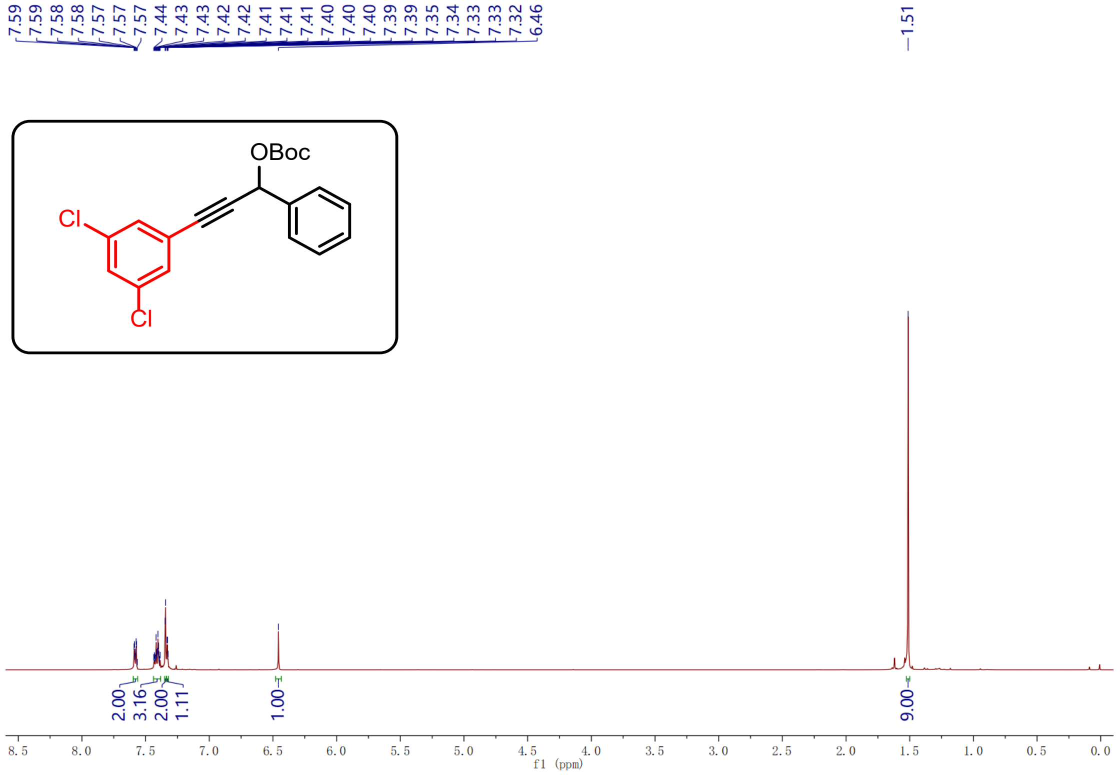

^{13}C NMR spectrum ($CDCl_3$) of **1ab**

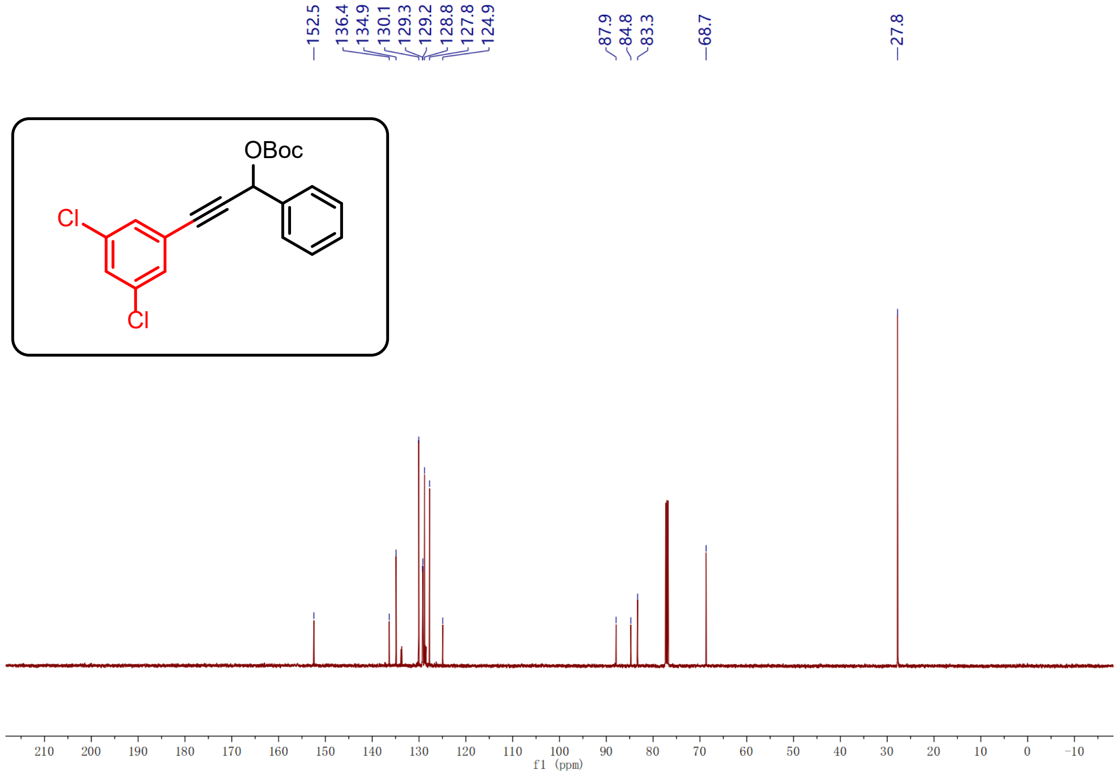

^{1}H NMR spectrum ($CDCl_3$) of **1ac**

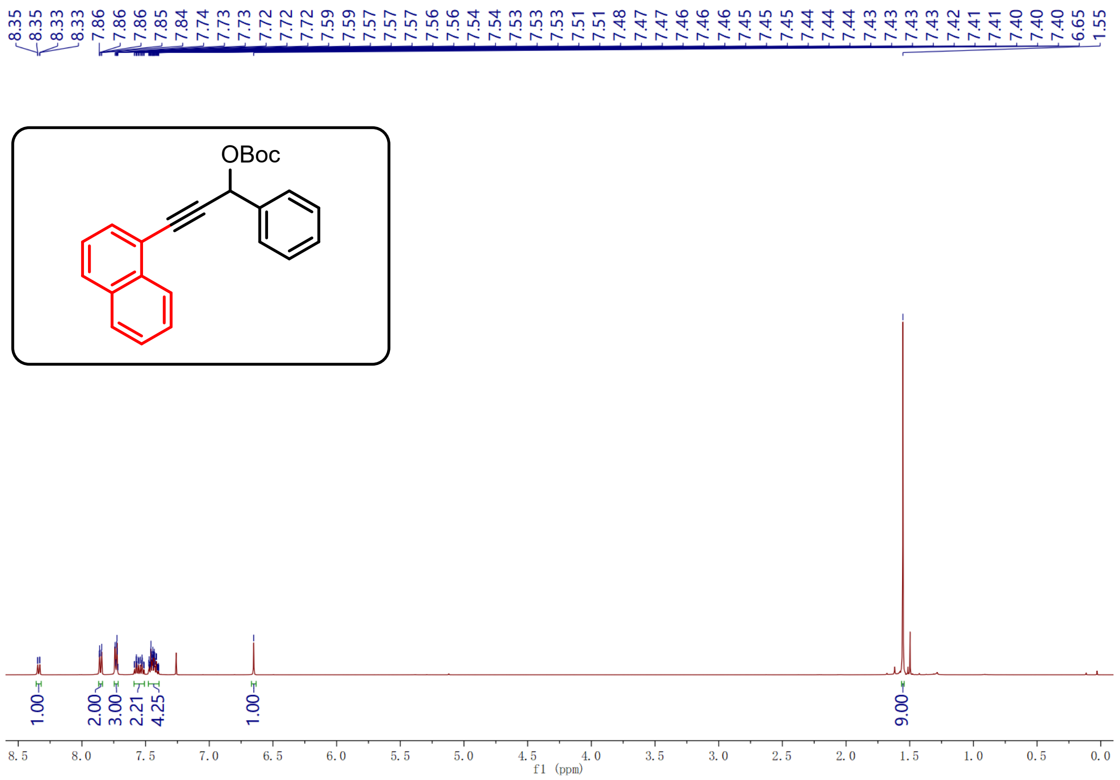

^{13}C NMR spectrum ($CDCl_3$) of **1ac**

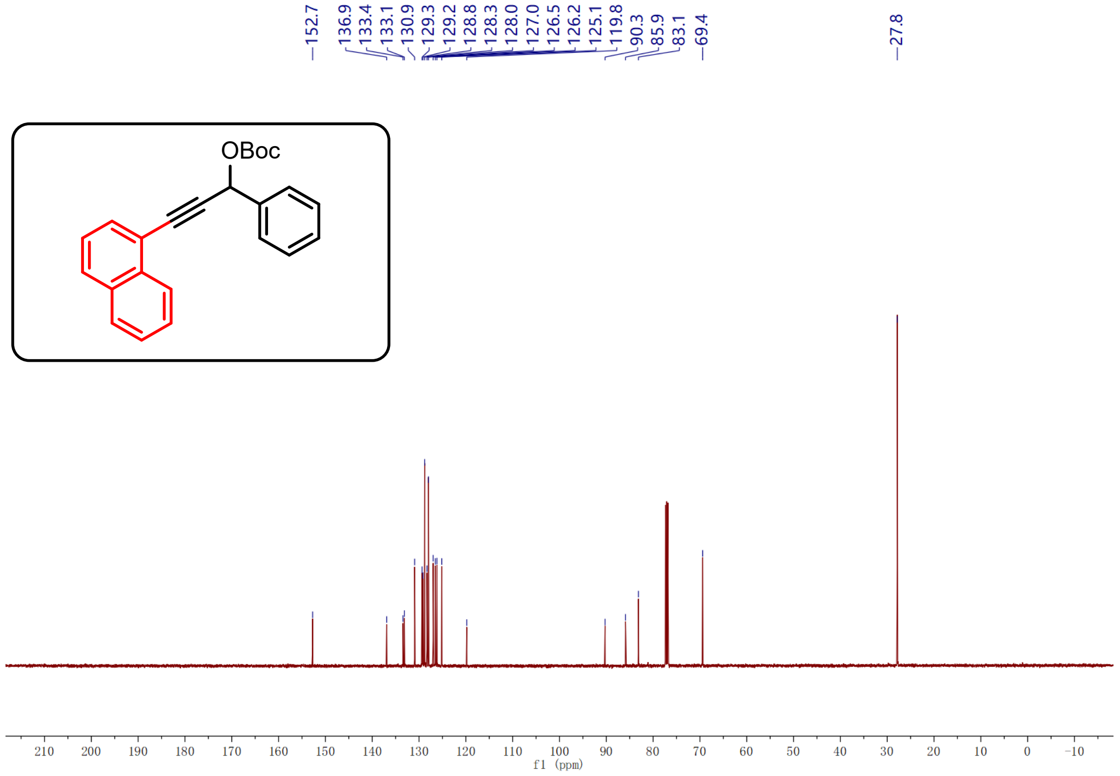

^{1}H NMR spectrum ($CDCl_3$) of **1ad**

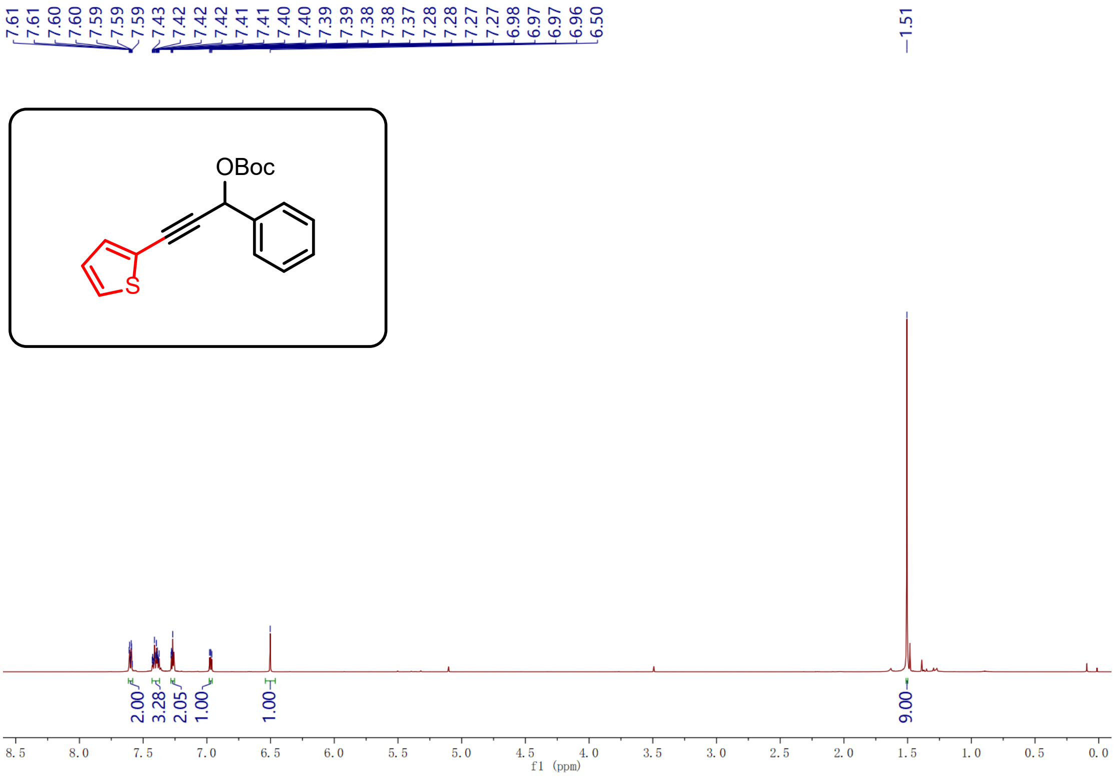

^{13}C NMR spectrum ($CDCl_3$) of **1ad**

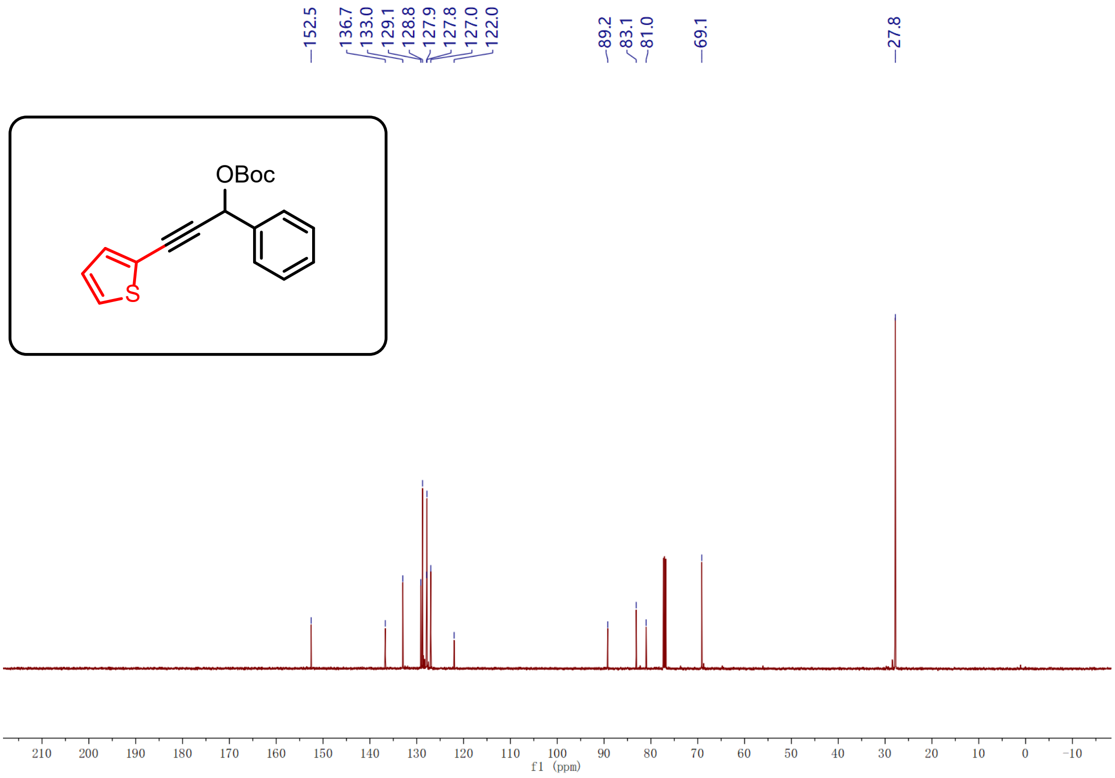

^{1}H NMR spectrum ($CDCl_3$) of **1ae**

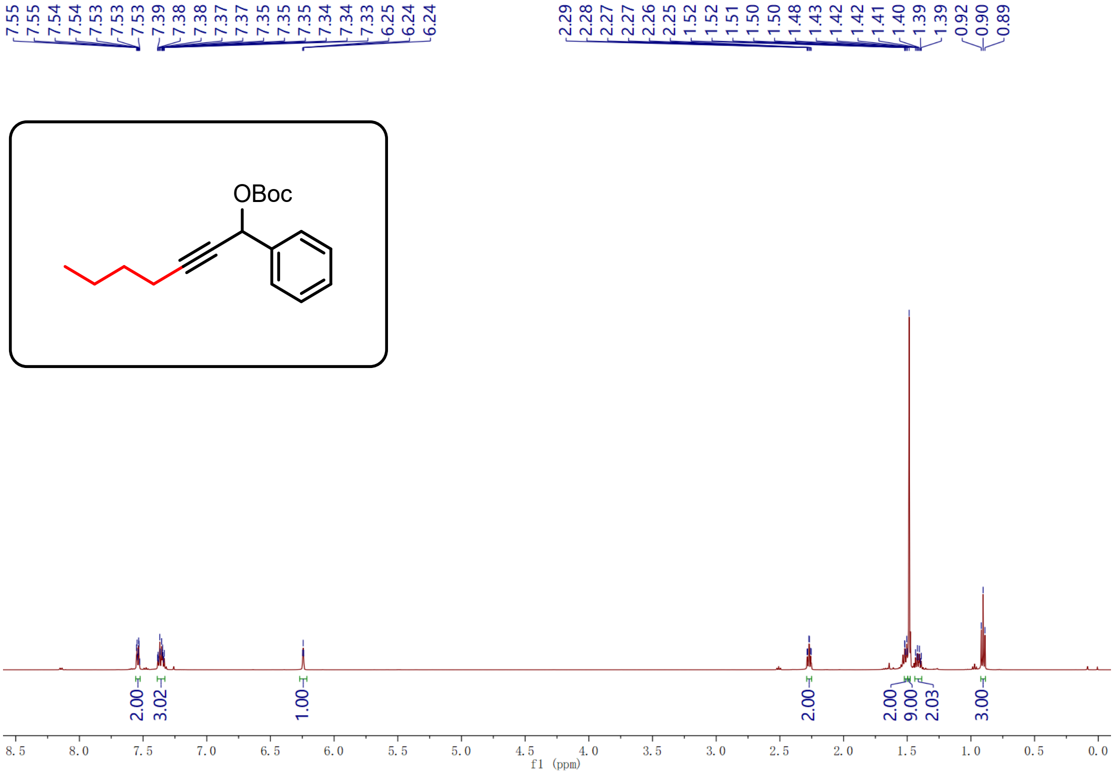

^{13}C NMR spectrum ($CDCl_3$) of **1ae**

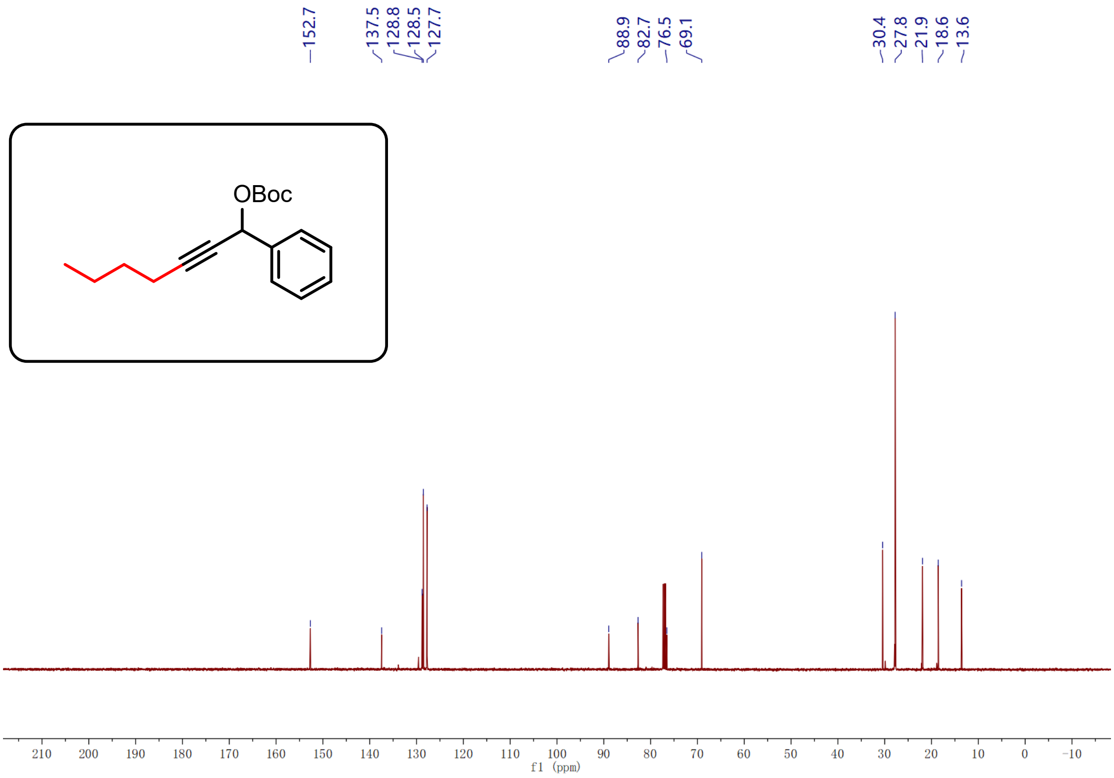

^{1}H NMR spectrum ($CDCl_3$) of **1af**

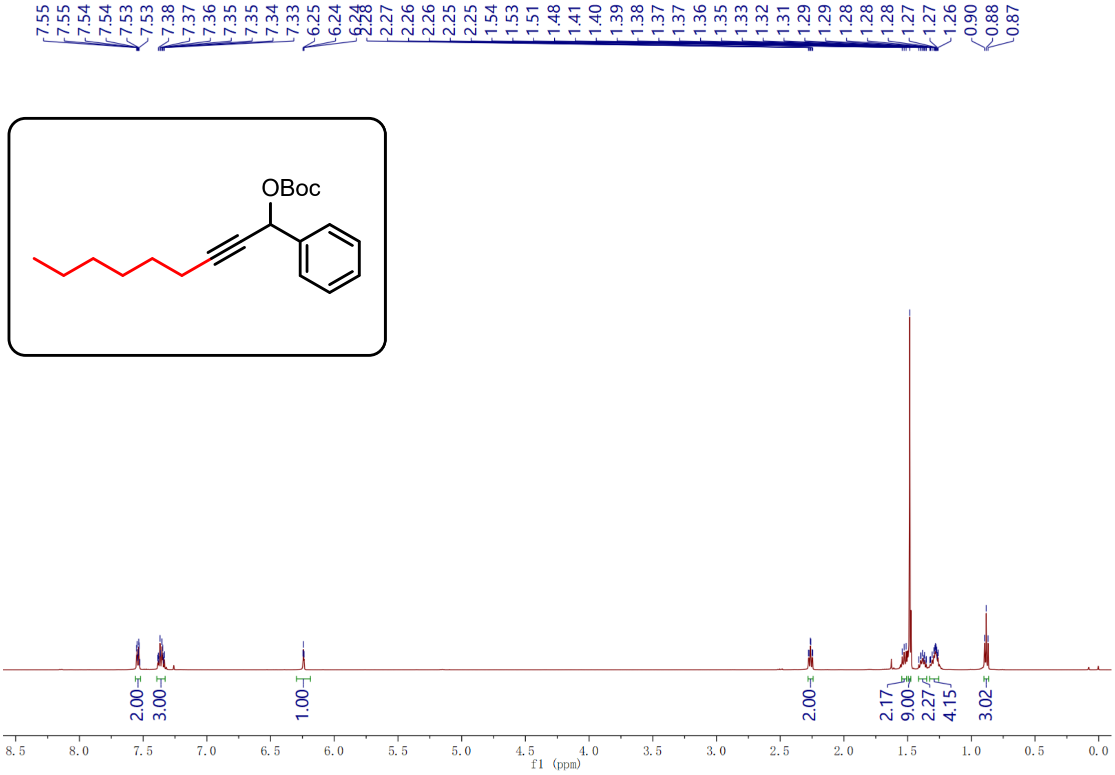

^{13}C NMR spectrum ($CDCl_3$) of **1af**

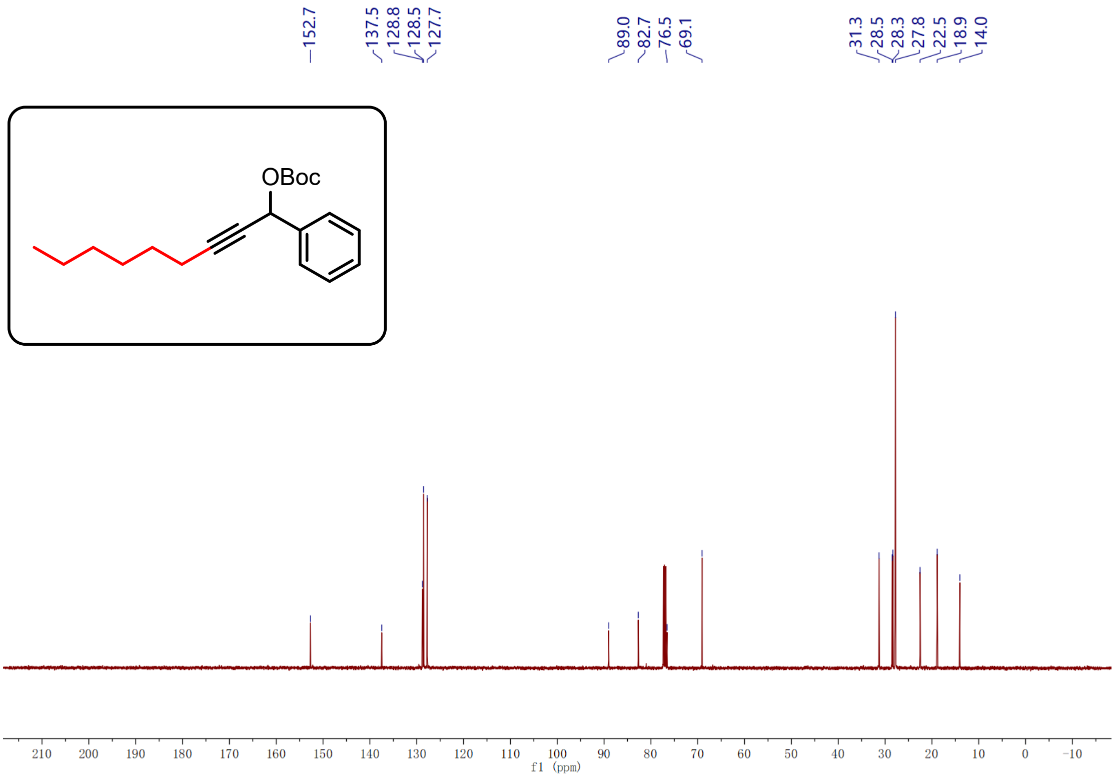

^{1}H NMR spectrum ($CDCl_3$) of **1ag**

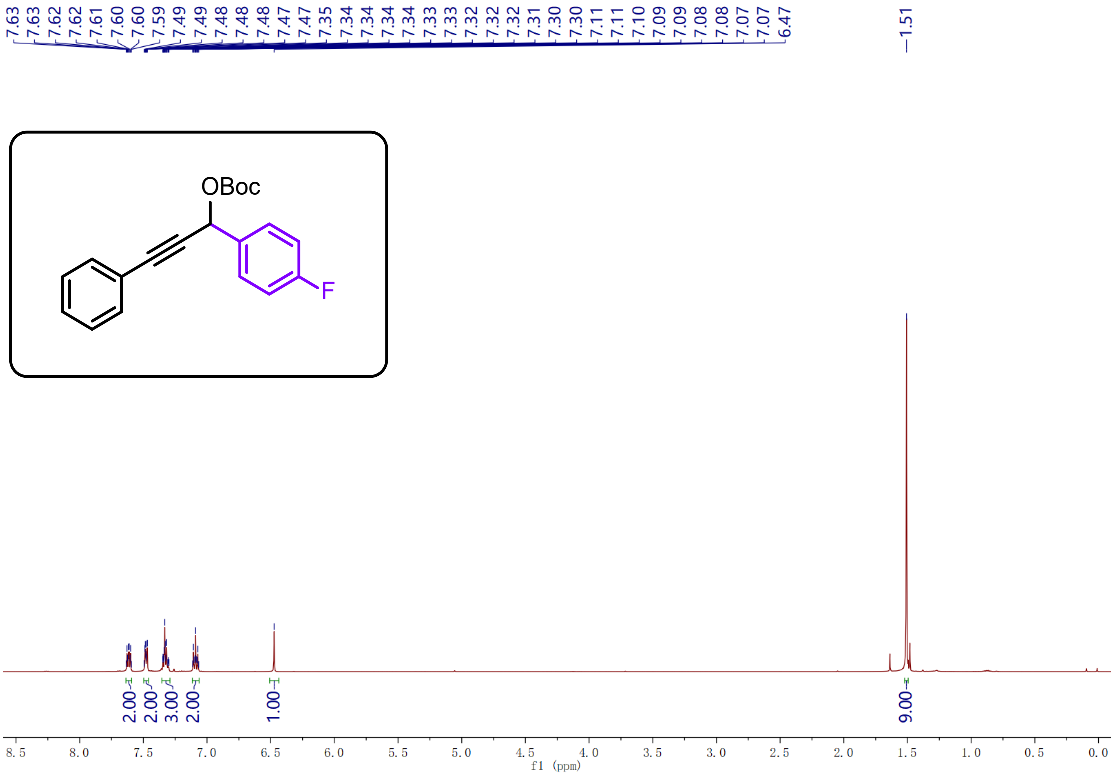

^{13}C NMR spectrum ($CDCl_3$) of **1ag**

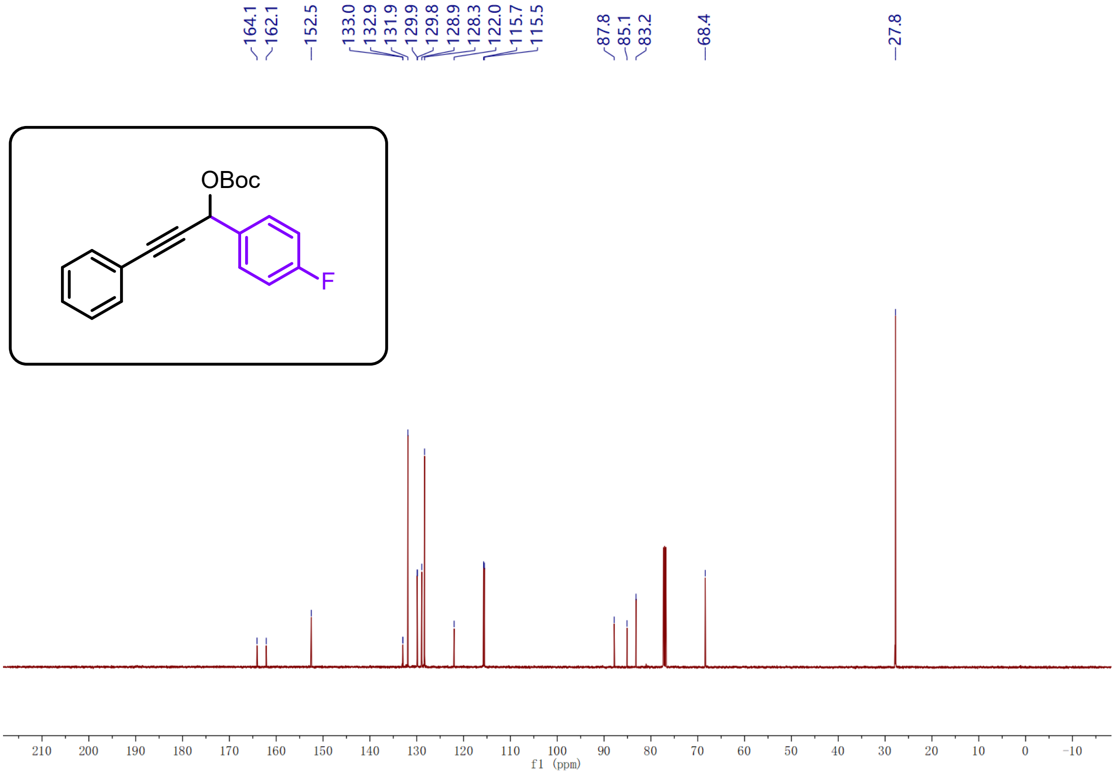

^{19}F NMR spectrum ($CDCl_3$) of **1ag**

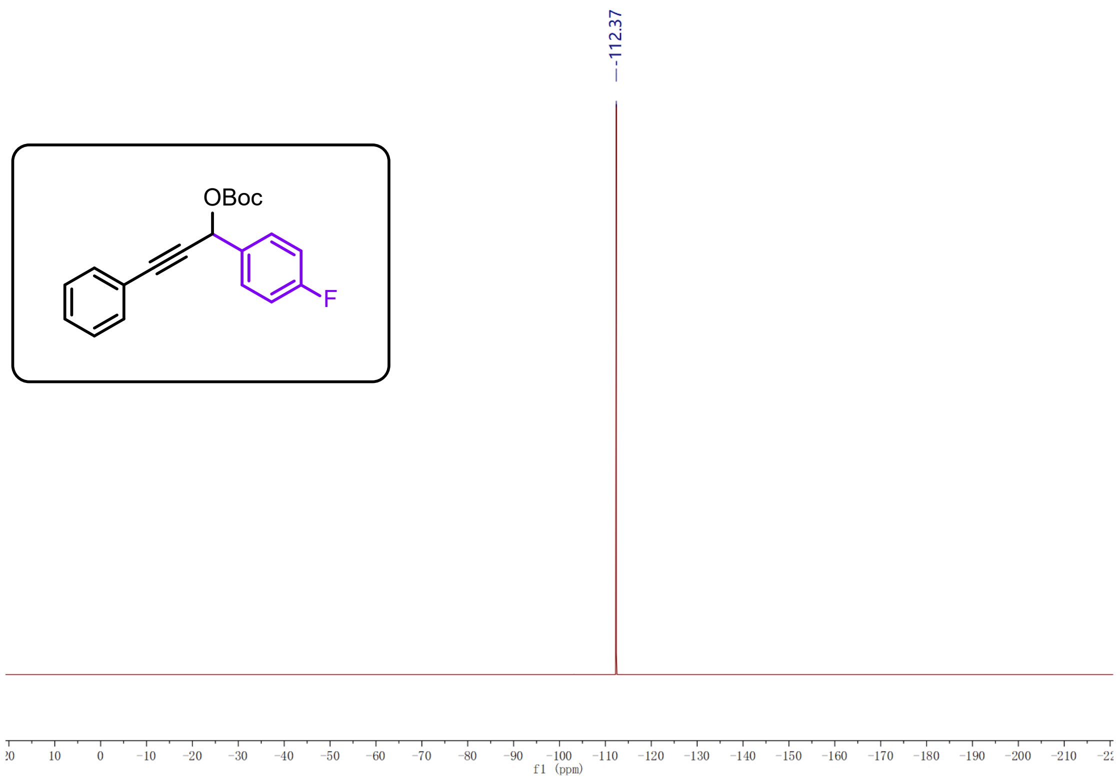

^{1}H NMR spectrum ($CDCl_3$) of **1ah**

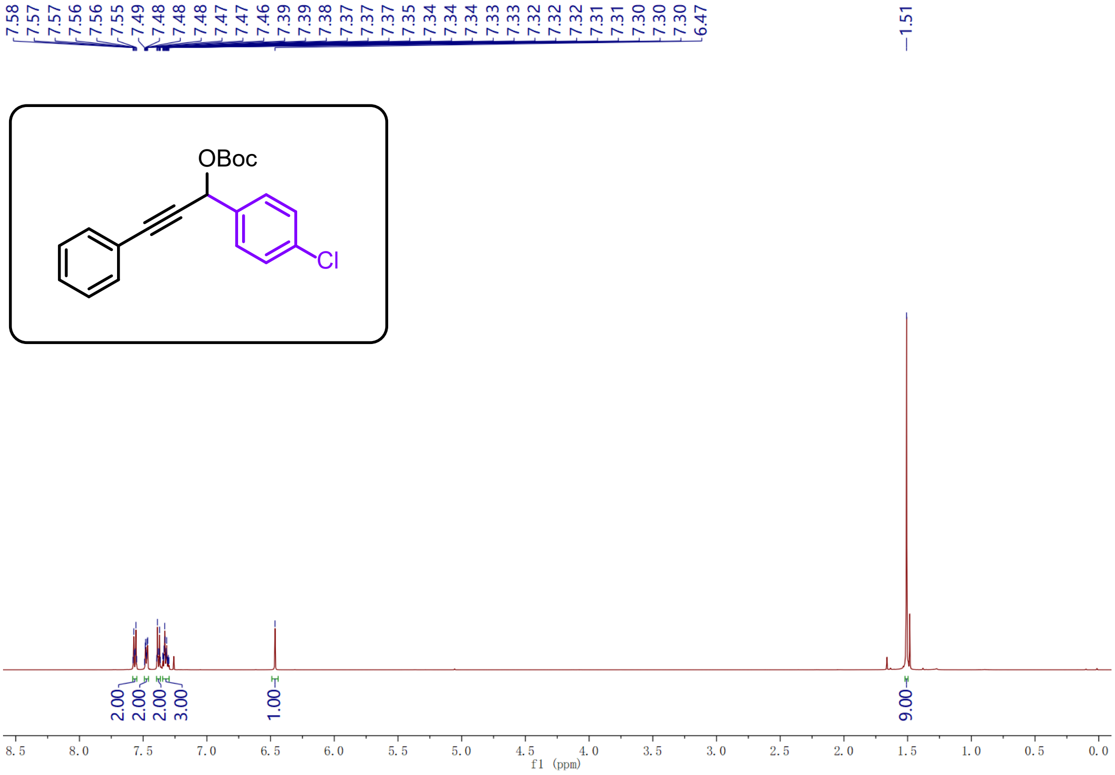

^{13}C NMR spectrum ($CDCl_3$) of **1ah**

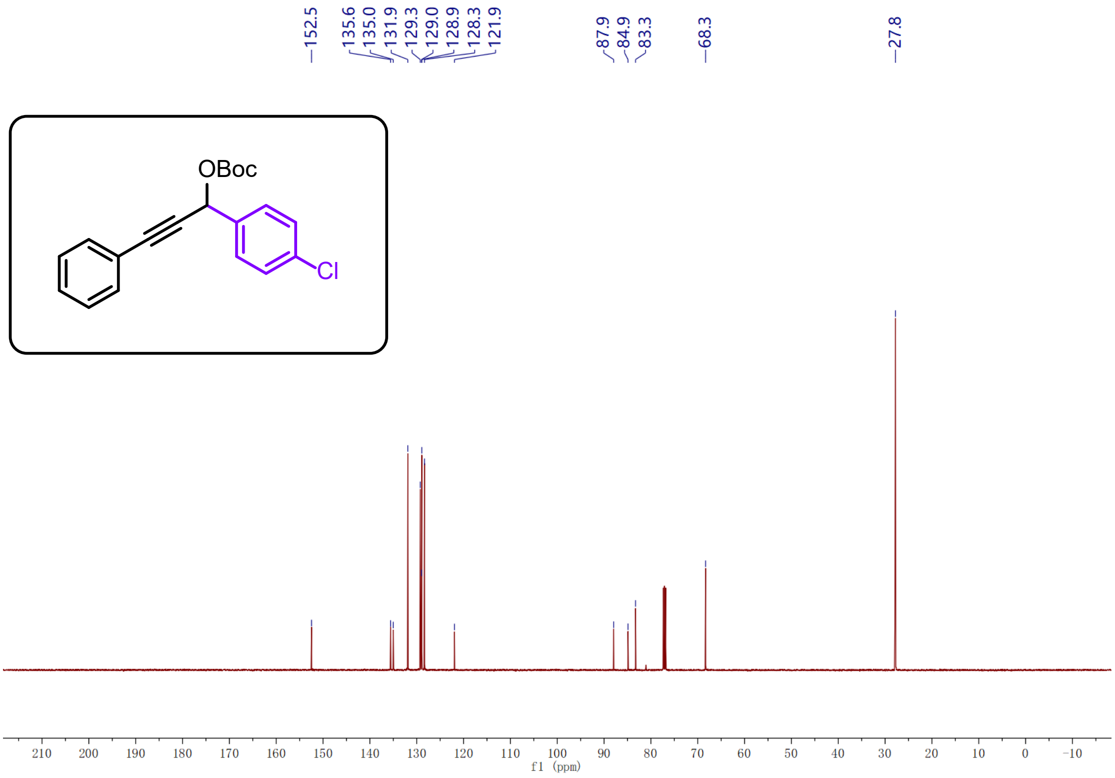

^{1}H NMR spectrum ($CDCl_3$) of **1ai**

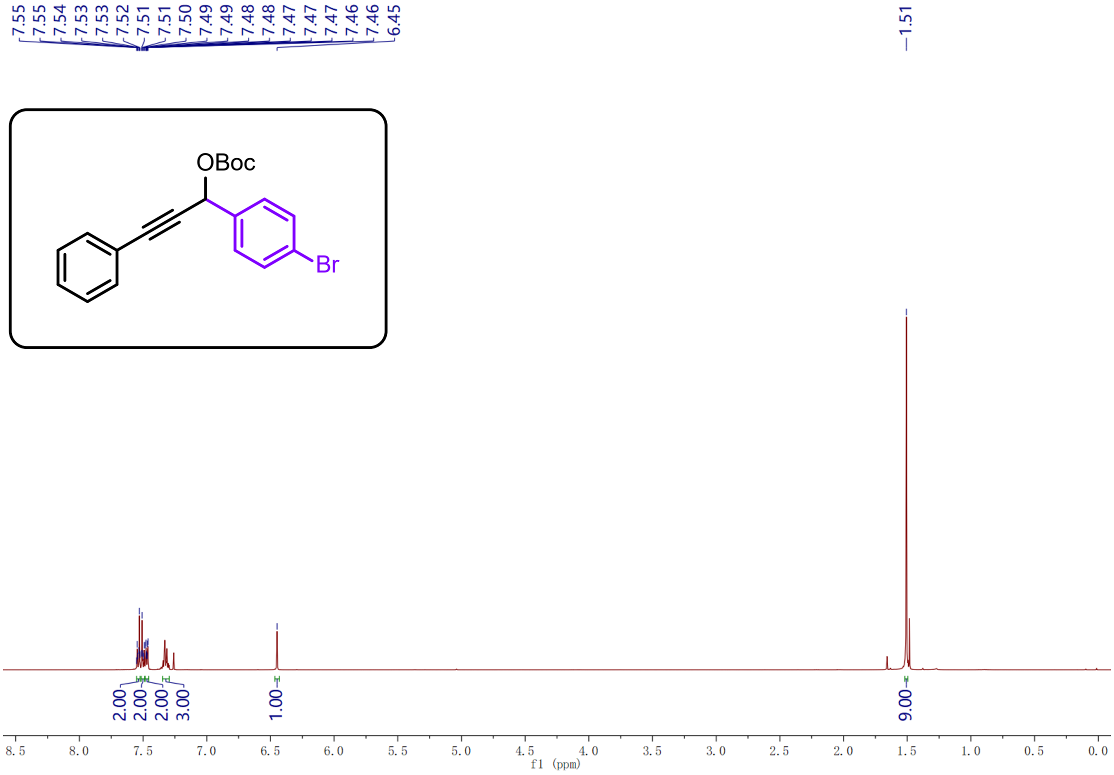

^{13}C NMR spectrum ($CDCl_3$) of **1ai**

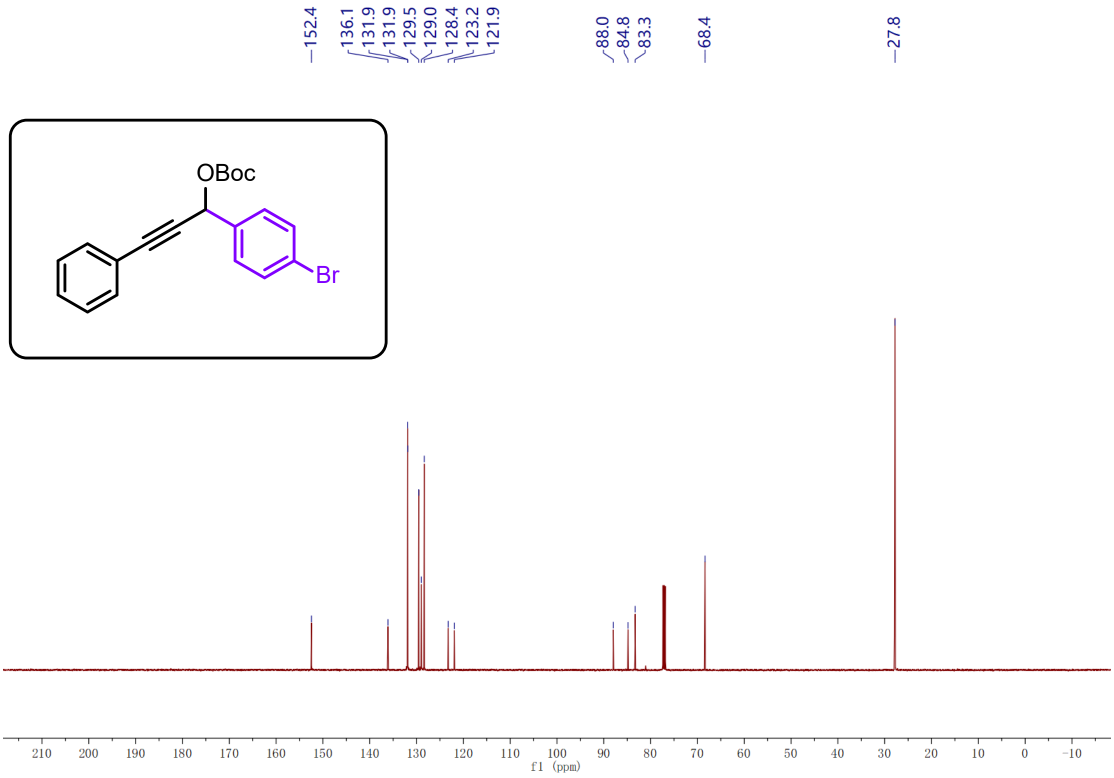

^{1}H NMR spectrum ($CDCl_3$) of **1aj**

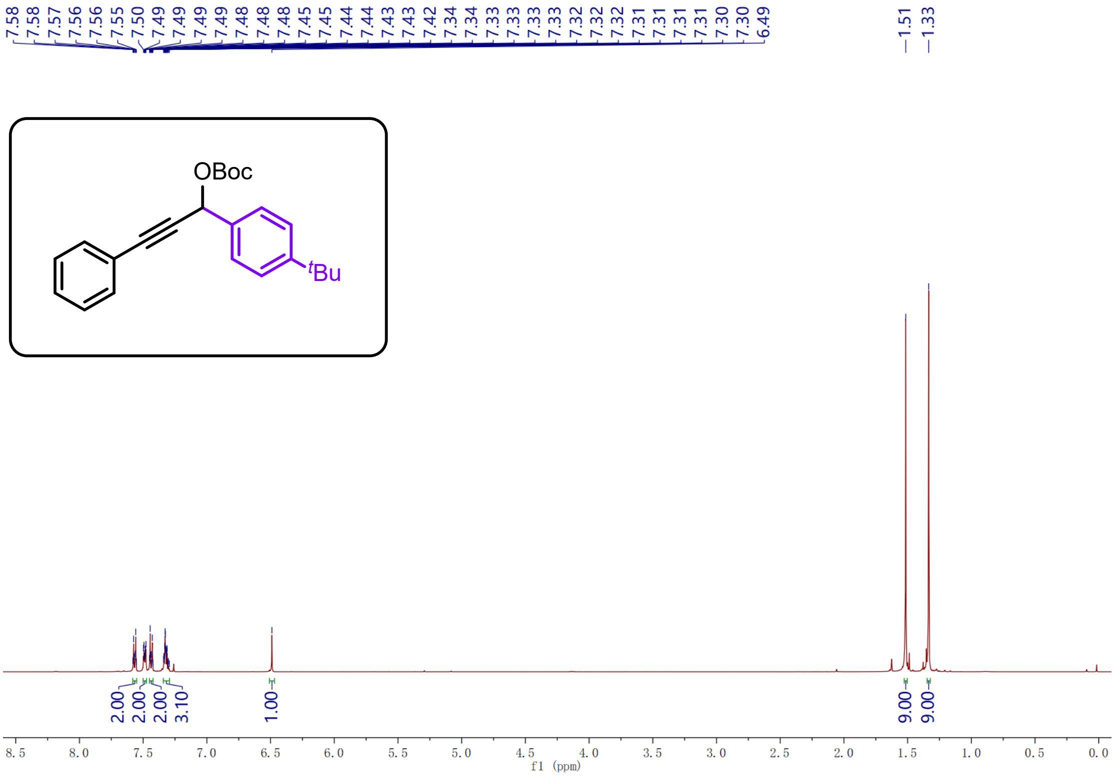

^{13}C NMR spectrum ($CDCl_3$) of **1aj**

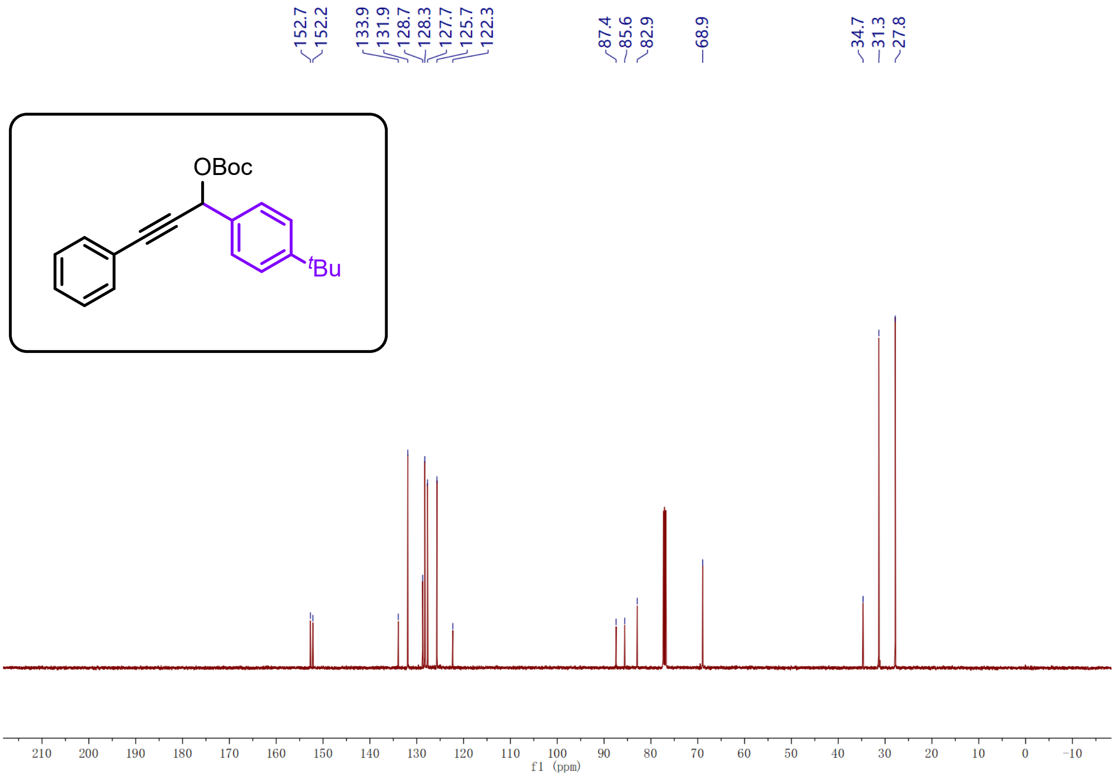

^{1}H NMR spectrum ($CDCl_3$) of **1ak**

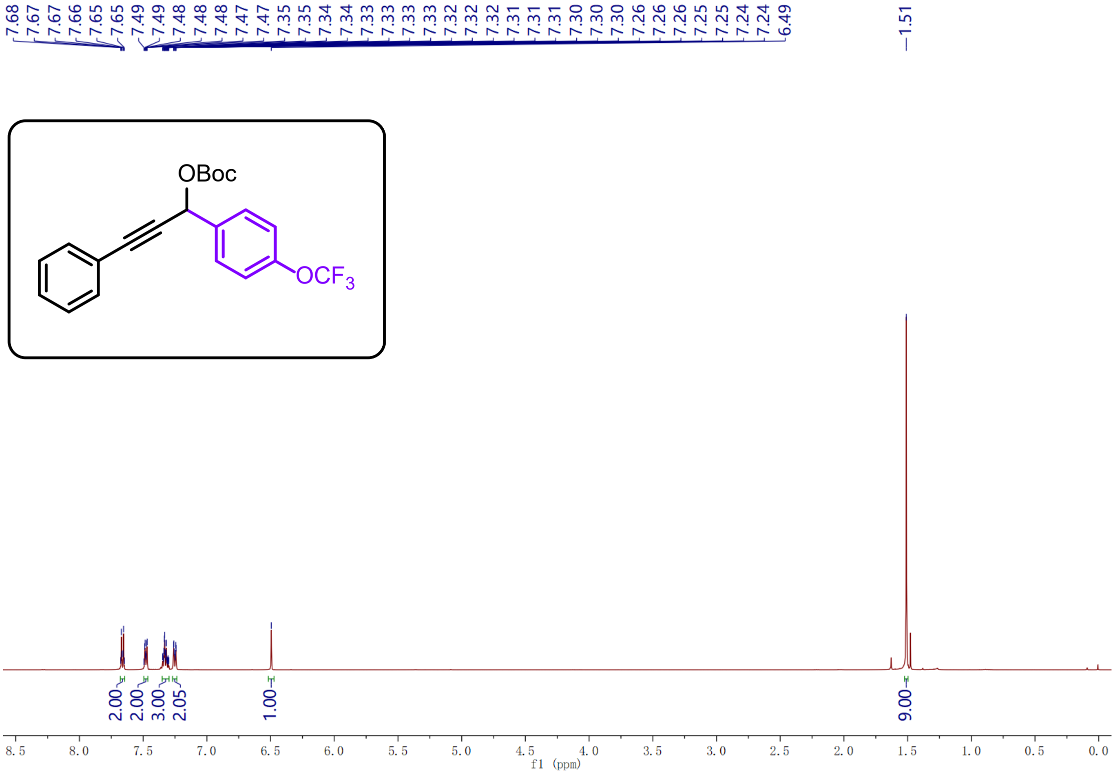

^{13}C NMR spectrum ($CDCl_3$) of **1ak**

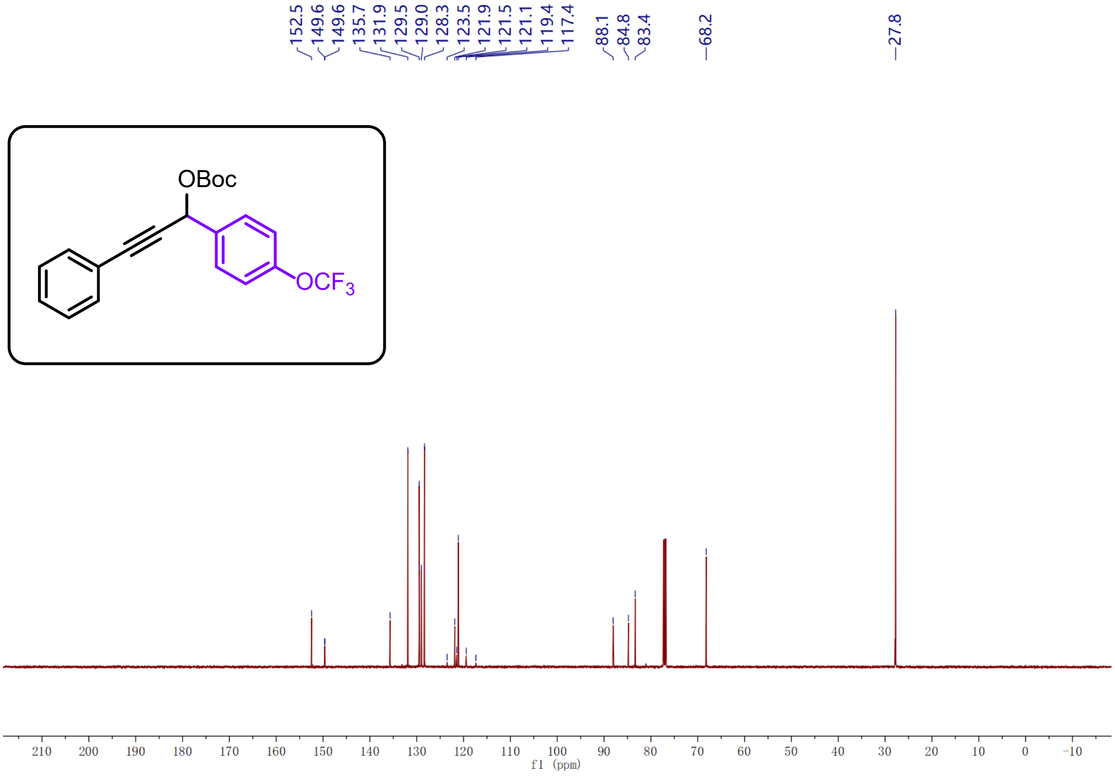

^{19}F NMR spectrum ($CDCl_3$) of **1ak**

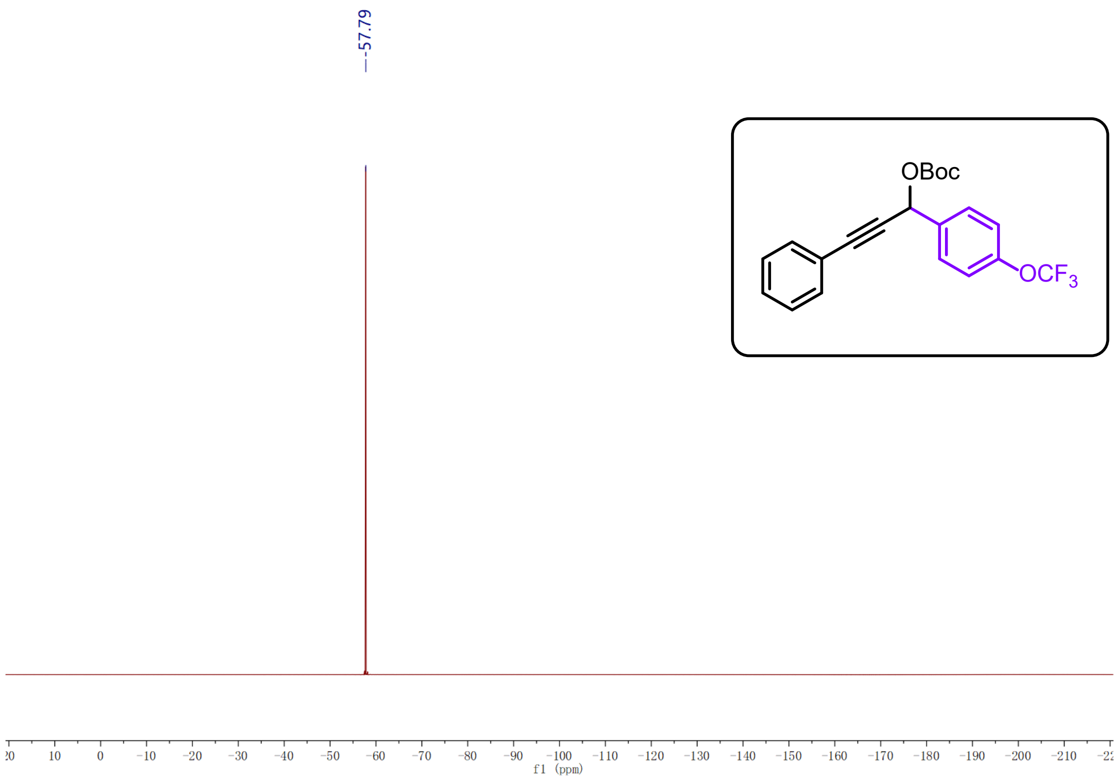

^{1}H NMR spectrum ($CDCl_3$) of **1al**

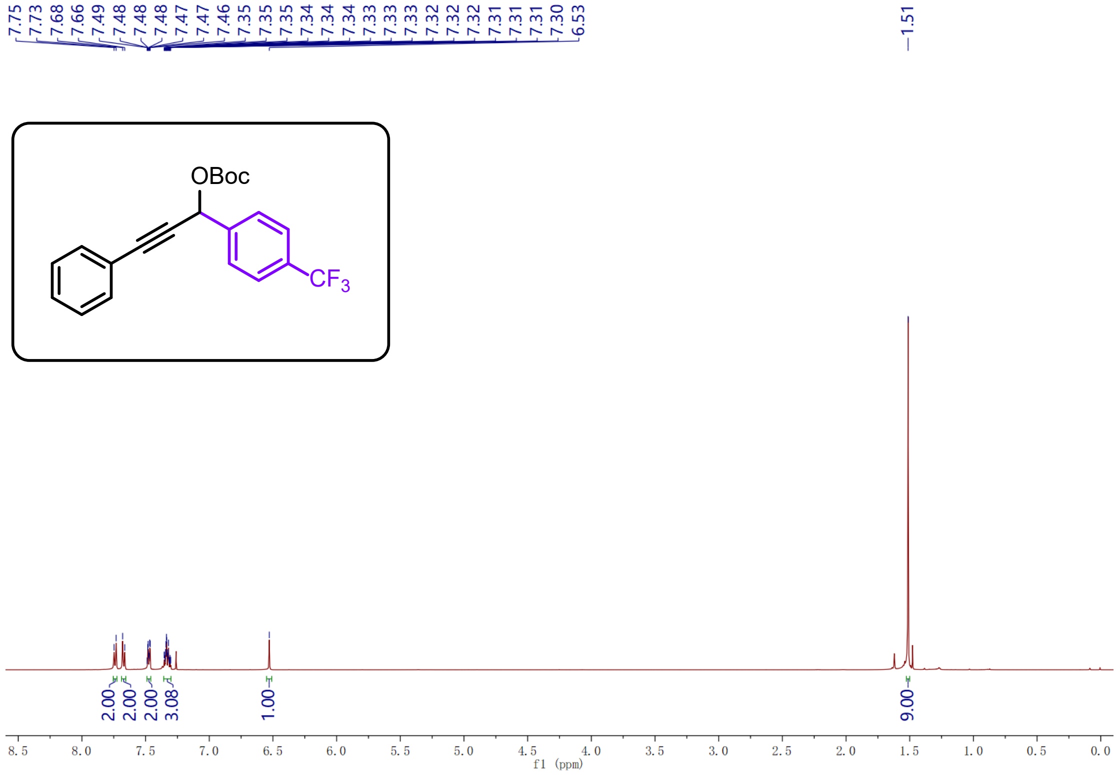

^{13}C NMR spectrum ($CDCl_3$) of **1al**

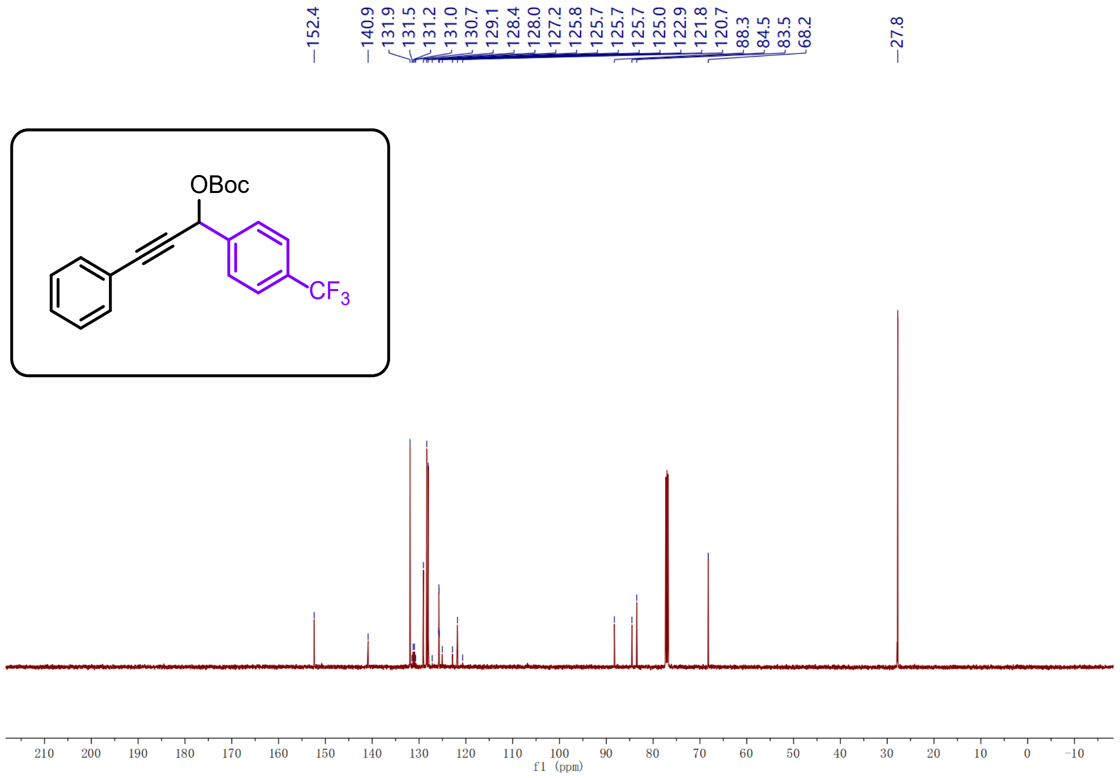

^{19}F NMR spectrum ($CDCl_3$) of **1al**

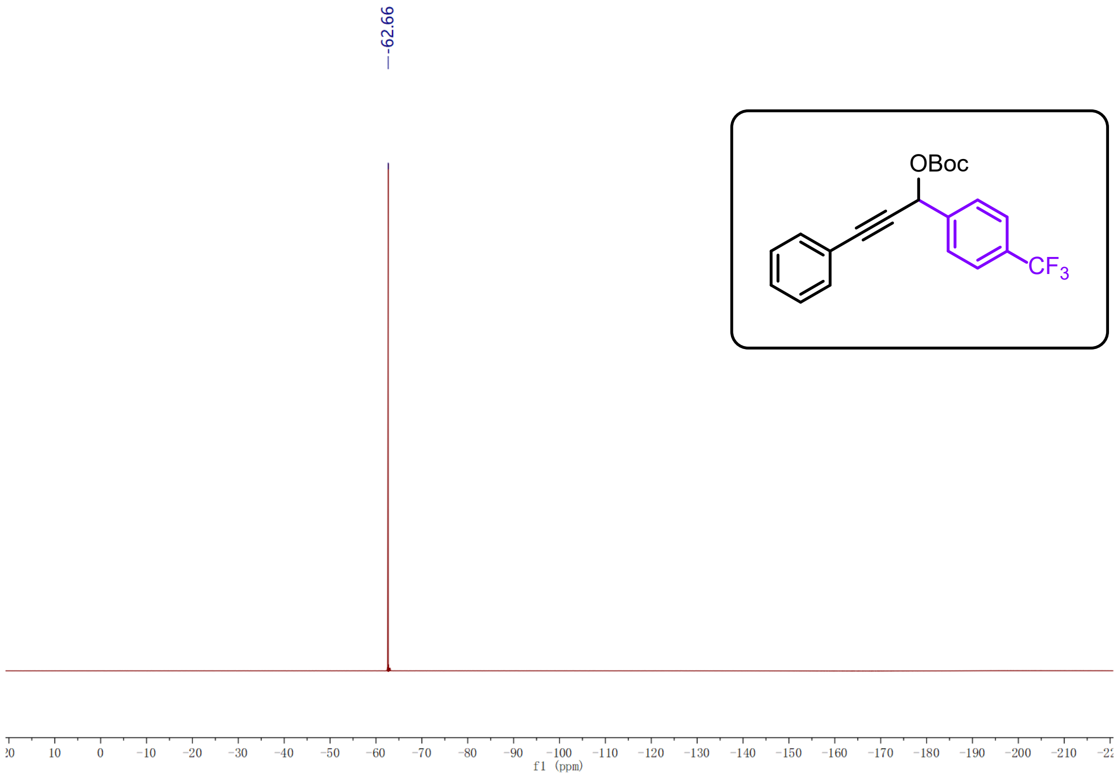

^{1}H NMR spectrum ($CDCl_3$) of **1am**

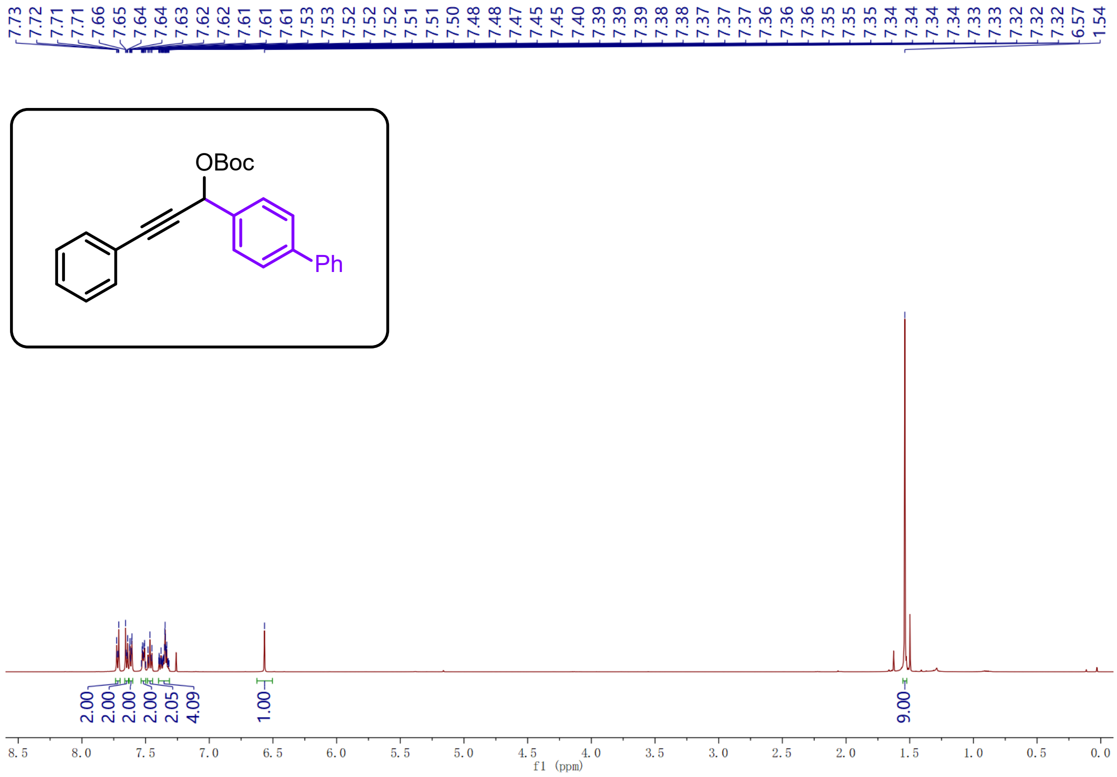

^{13}C NMR spectrum ($CDCl_3$) of **1am**

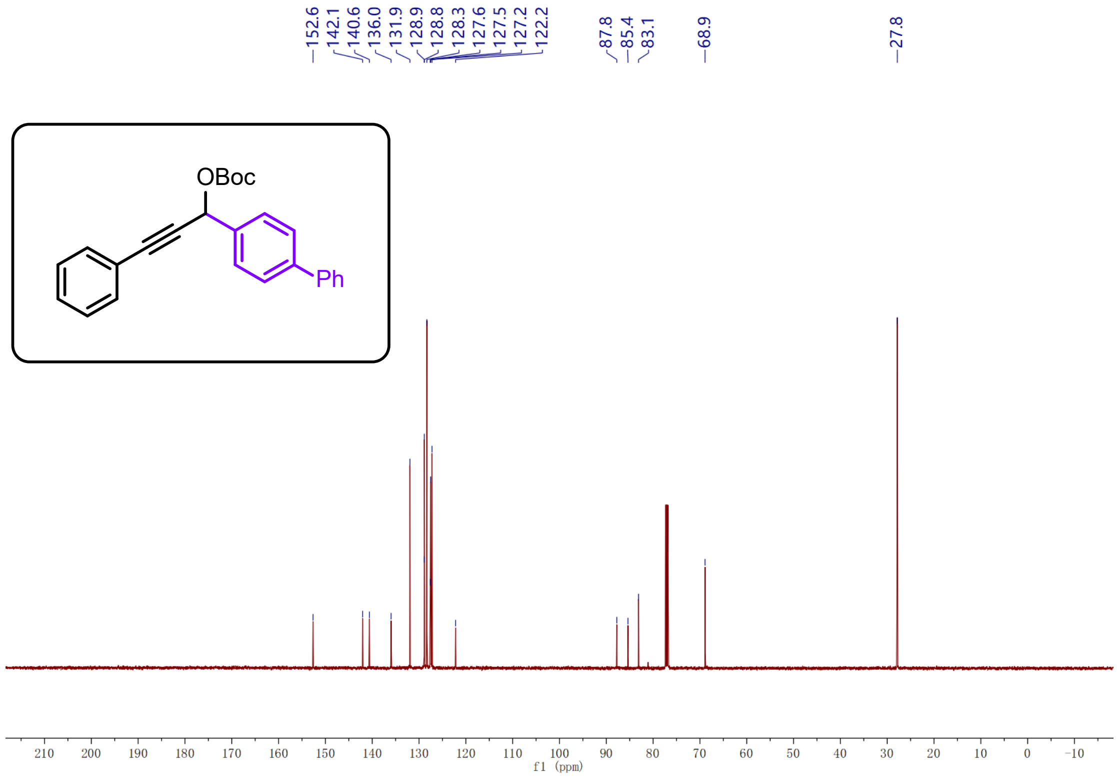

^{1}H NMR spectrum ($CDCl_3$) of **1an**

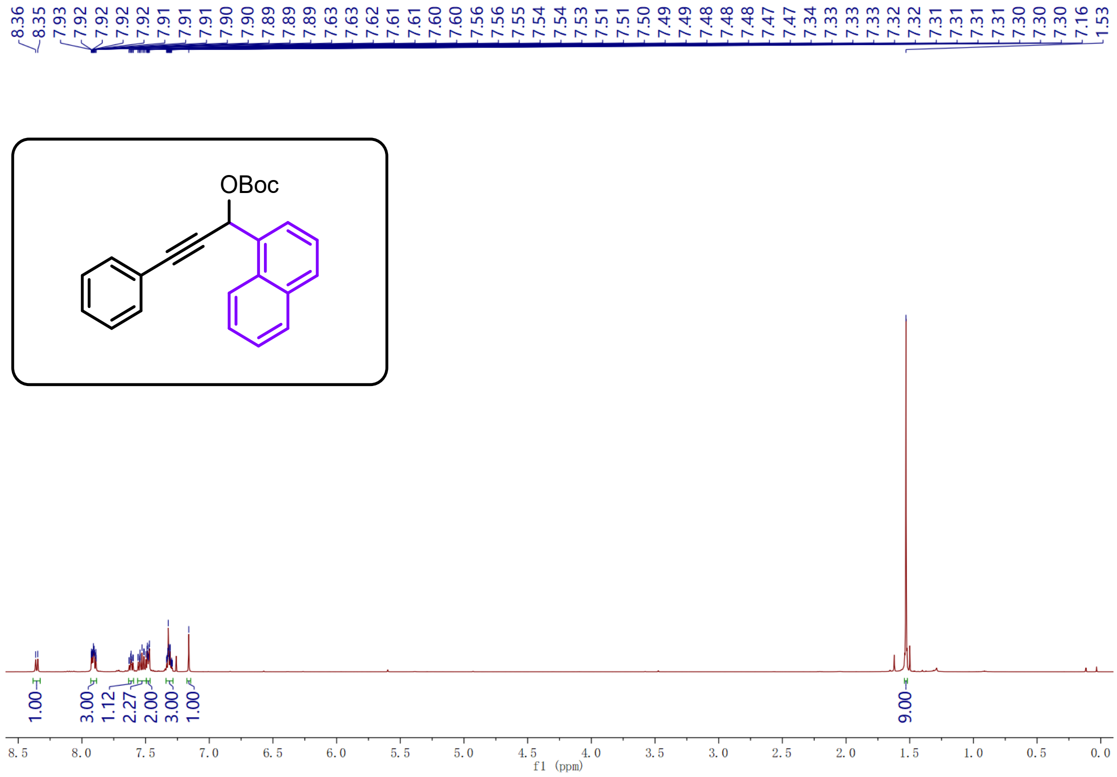

^{13}C NMR spectrum ($CDCl_3$) of **1an**

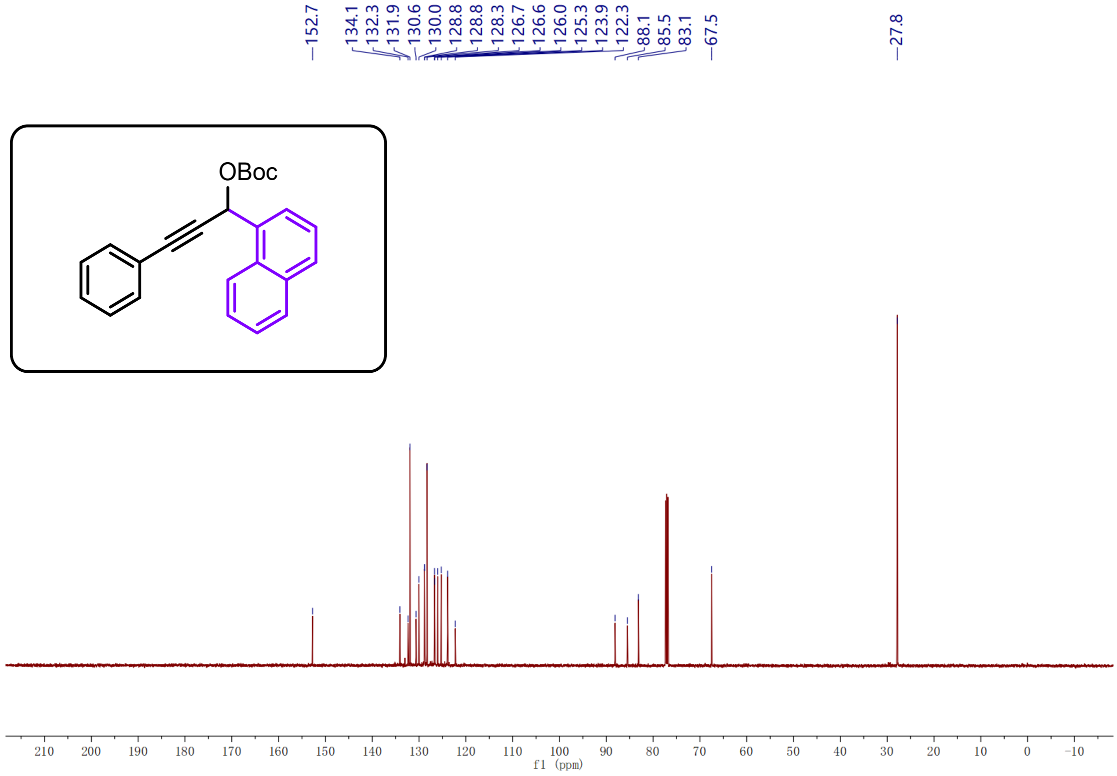

^{1}H NMR spectrum ($CDCl_3$) of **1ao**

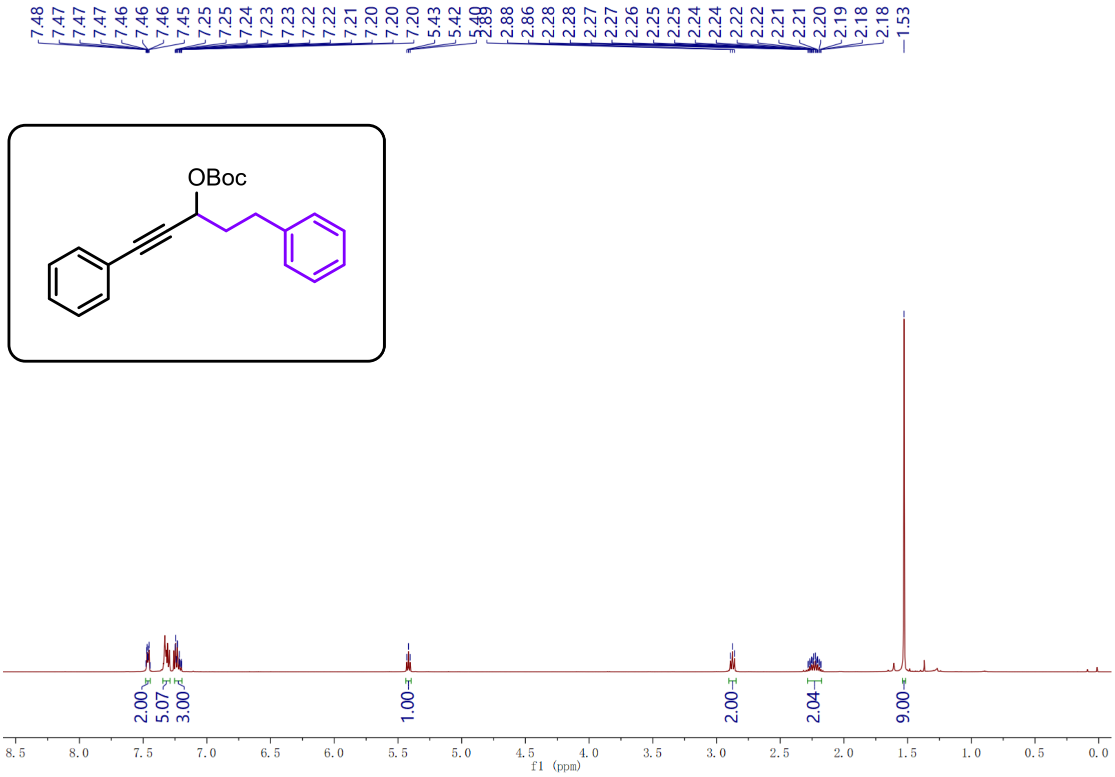

^{13}C NMR spectrum ($CDCl_3$) of **1ao**

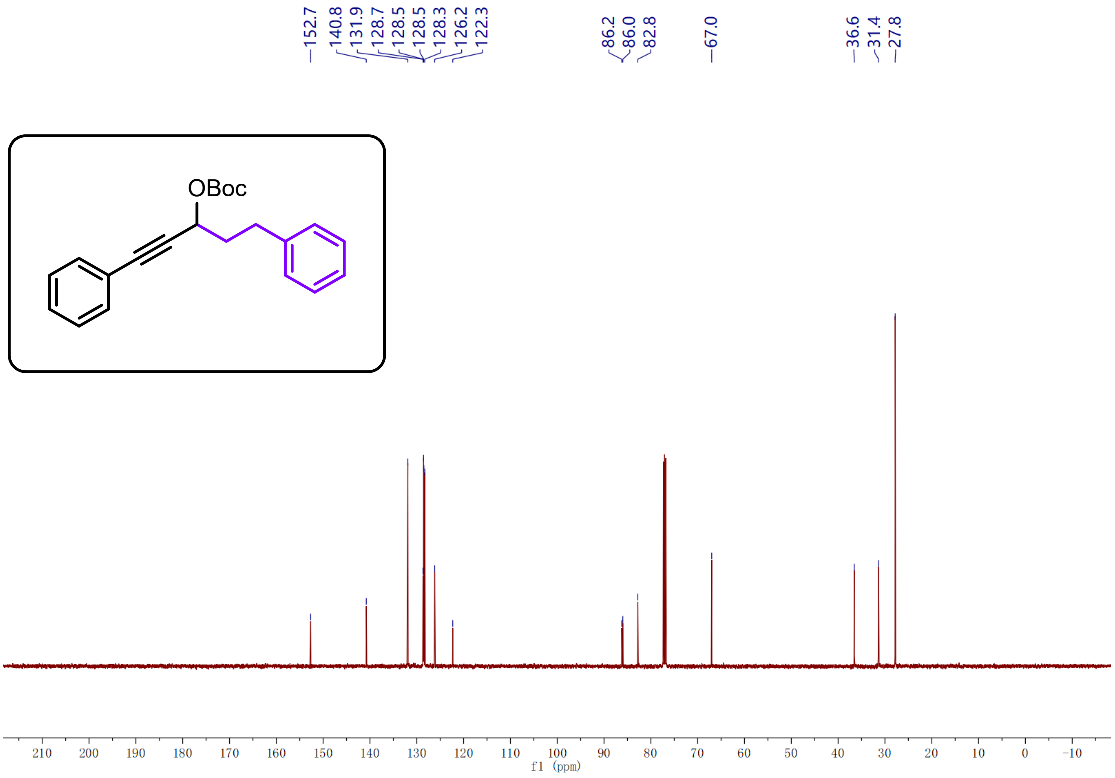

^{1}H NMR spectrum ($CDCl_3$) of **1ap**

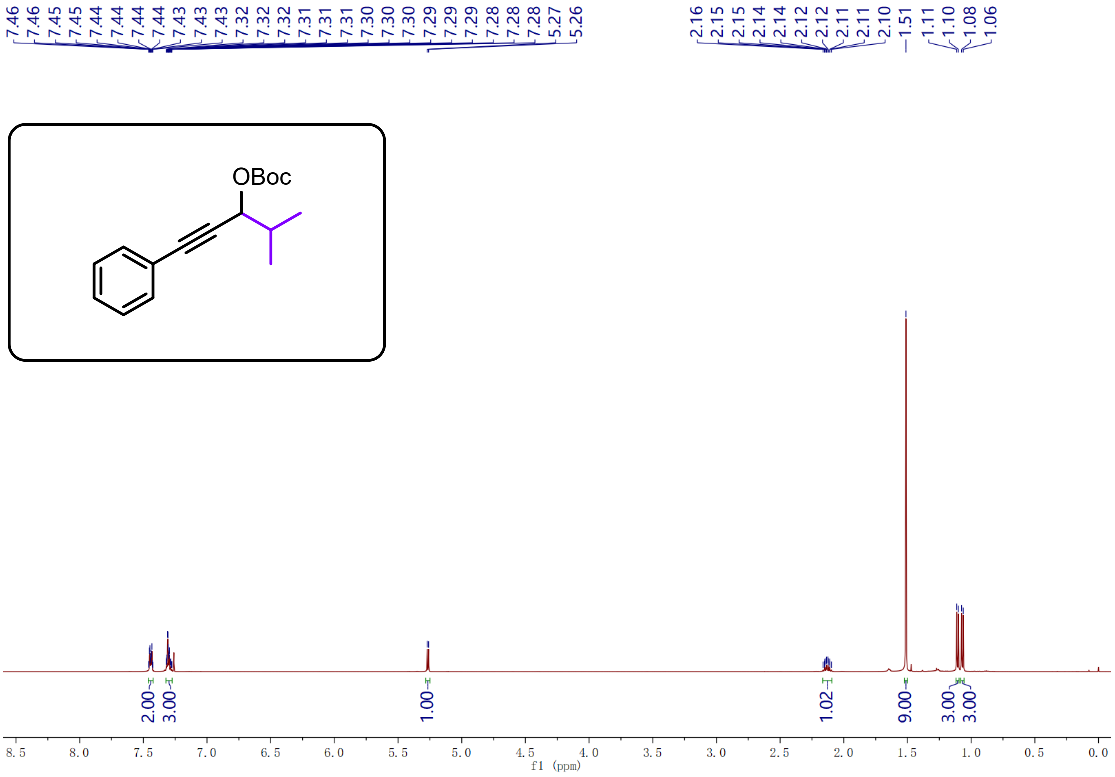

^{13}C NMR spectrum ($CDCl_3$) of **1ap**

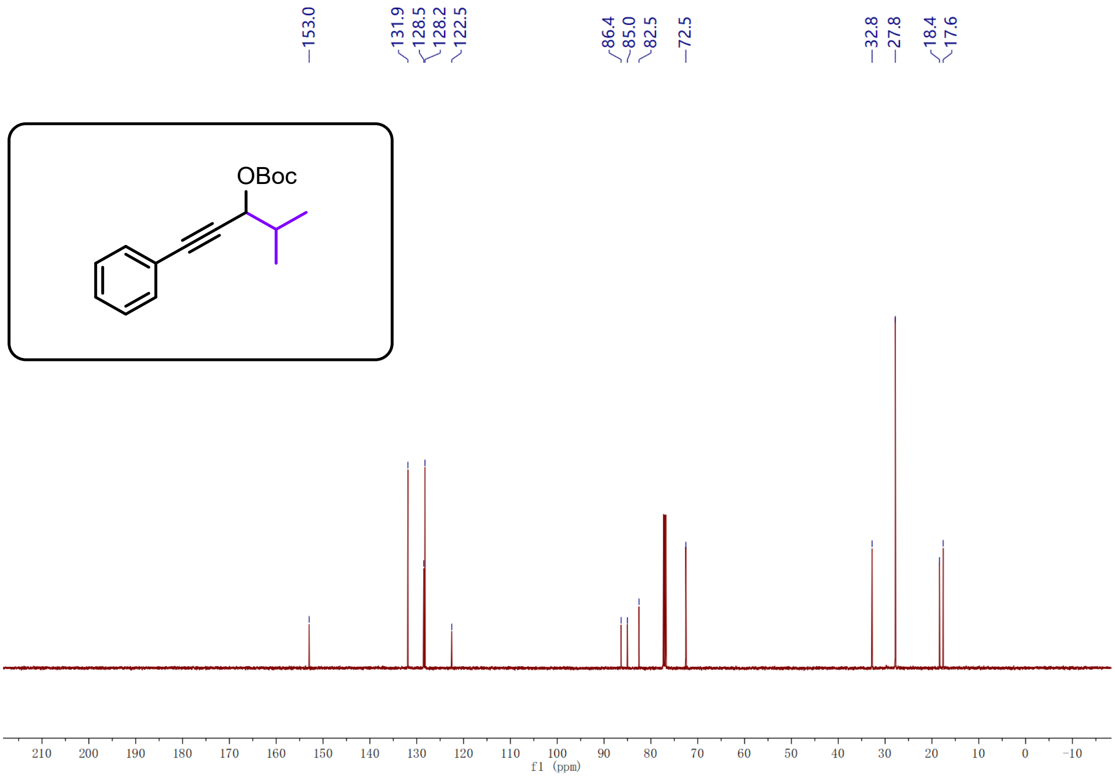

^{1}H NMR spectrum ($CDCl_3$) of **1,3-diphenylprop-2-yn-1-yl acetate**

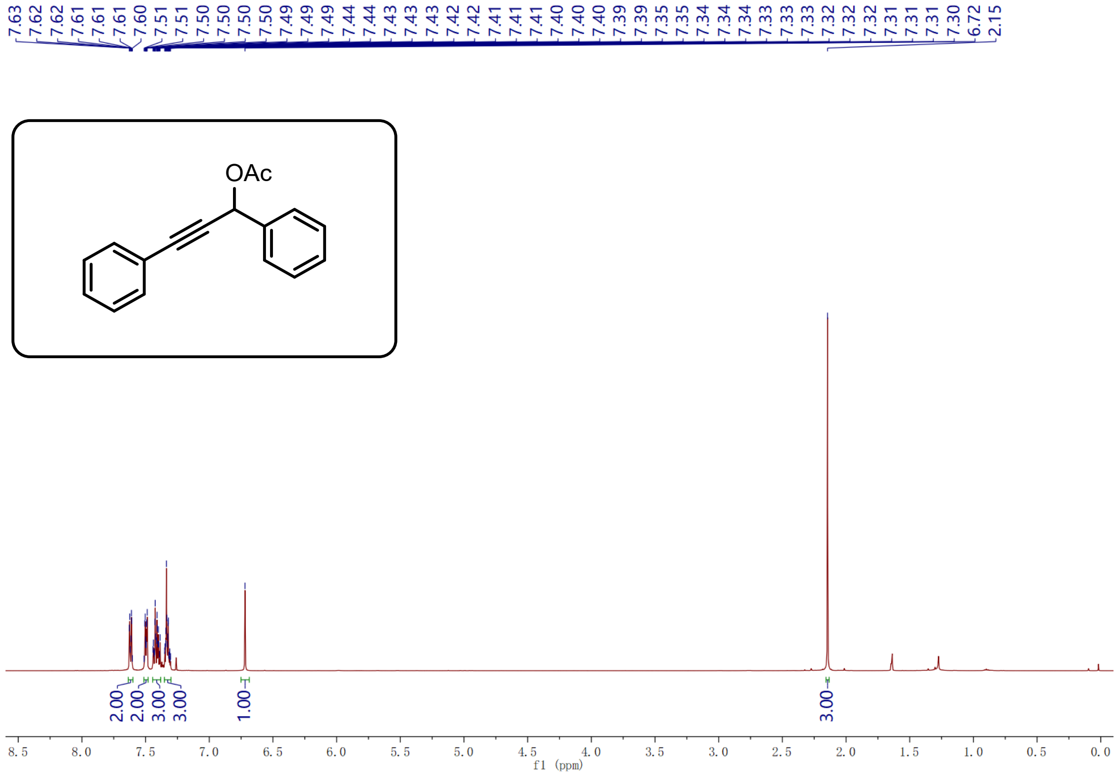

^{13}C NMR spectrum ($CDCl_3$) of **1,3-diphenylprop-2-yn-1-yl acetate**

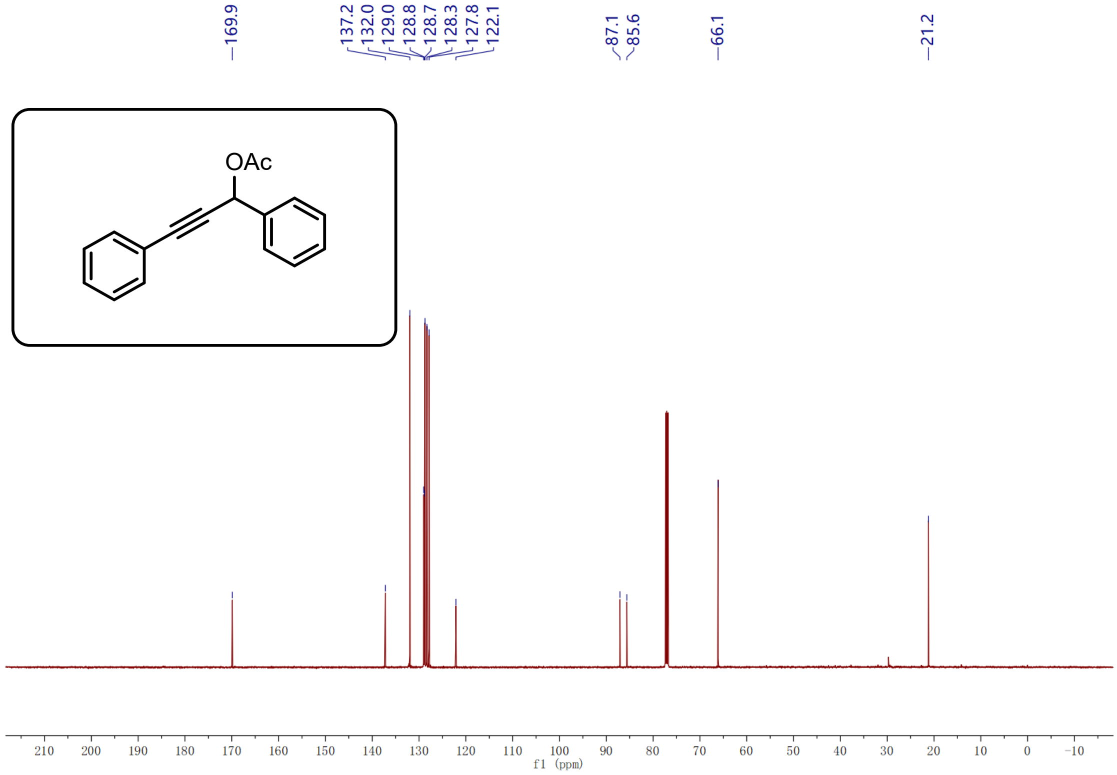

^{1}H NMR spectrum ($CDCl_3$) of **1,3-diphenylprop-2-yn-1-yl benzoate**

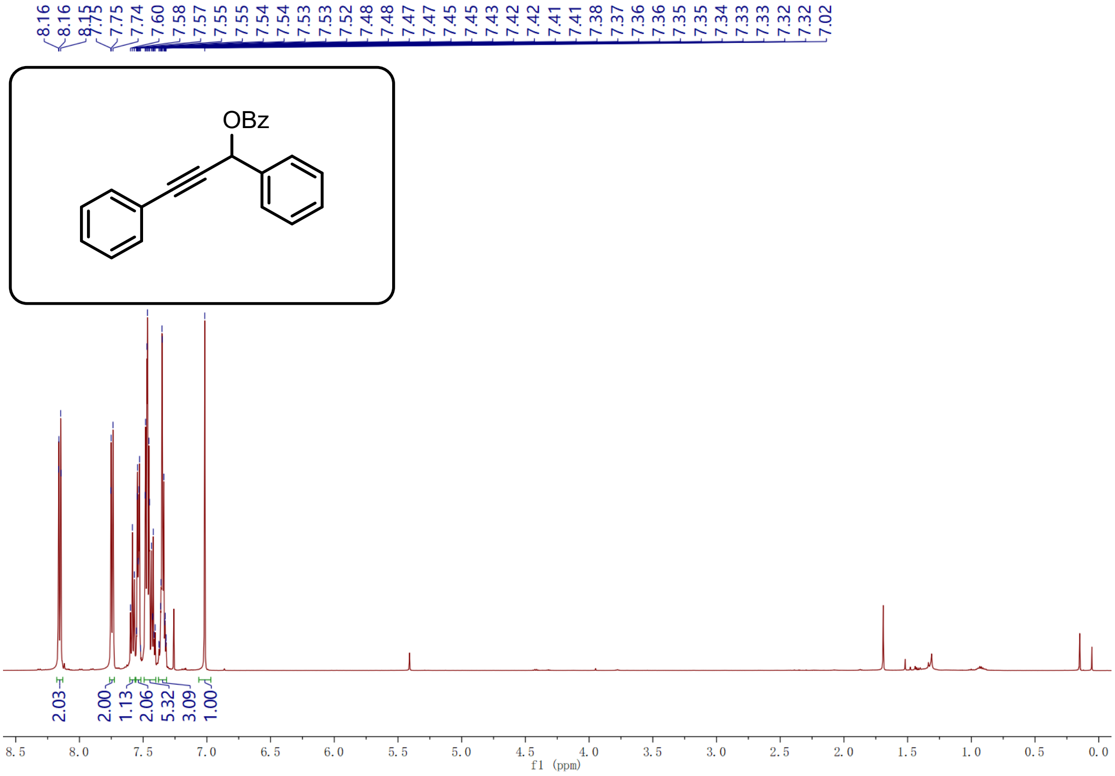

^{13}C NMR spectrum ($CDCl_3$) of **1,3-diphenylprop-2-yn-1-yl benzoate**

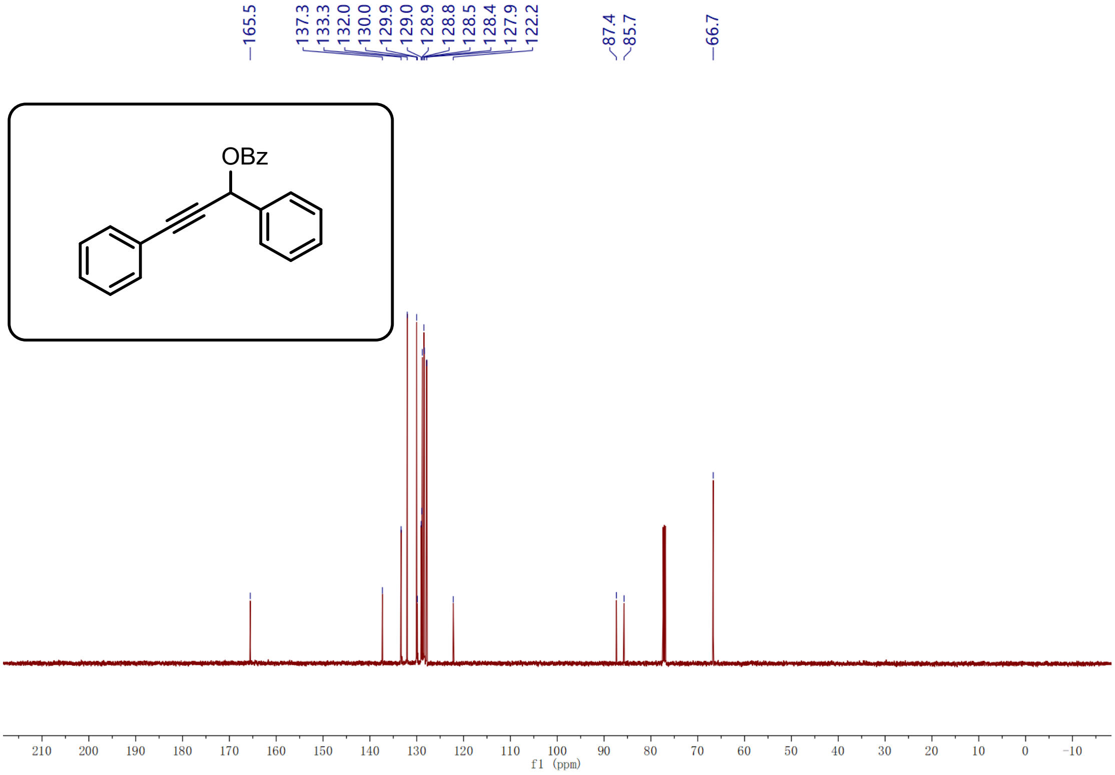

^{1}H NMR spectrum ($CDCl_3$) of **1,3-diphenylprop-2-yn-1-yl pivalate**

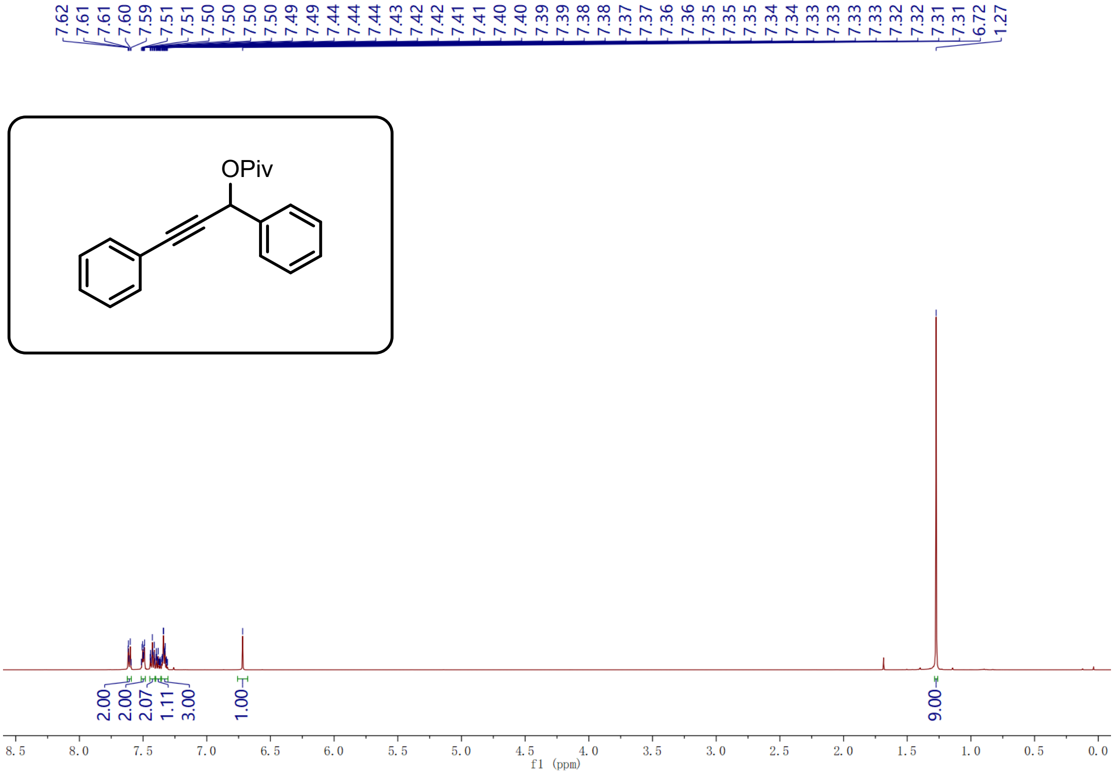

^{13}C NMR spectrum ($CDCl_3$) of **1,3-diphenylprop-2-yn-1-yl pivalate**

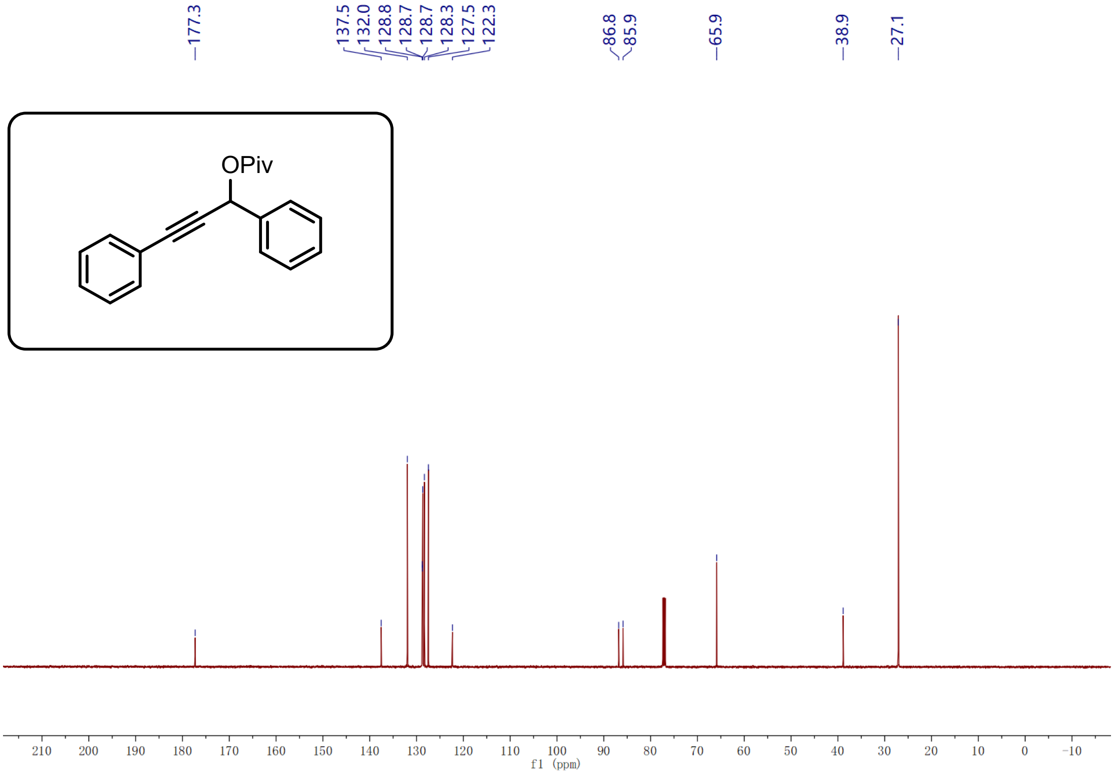

^{1}H NMR spectrum ($CDCl_3$) of **Et3Si-Bpin**

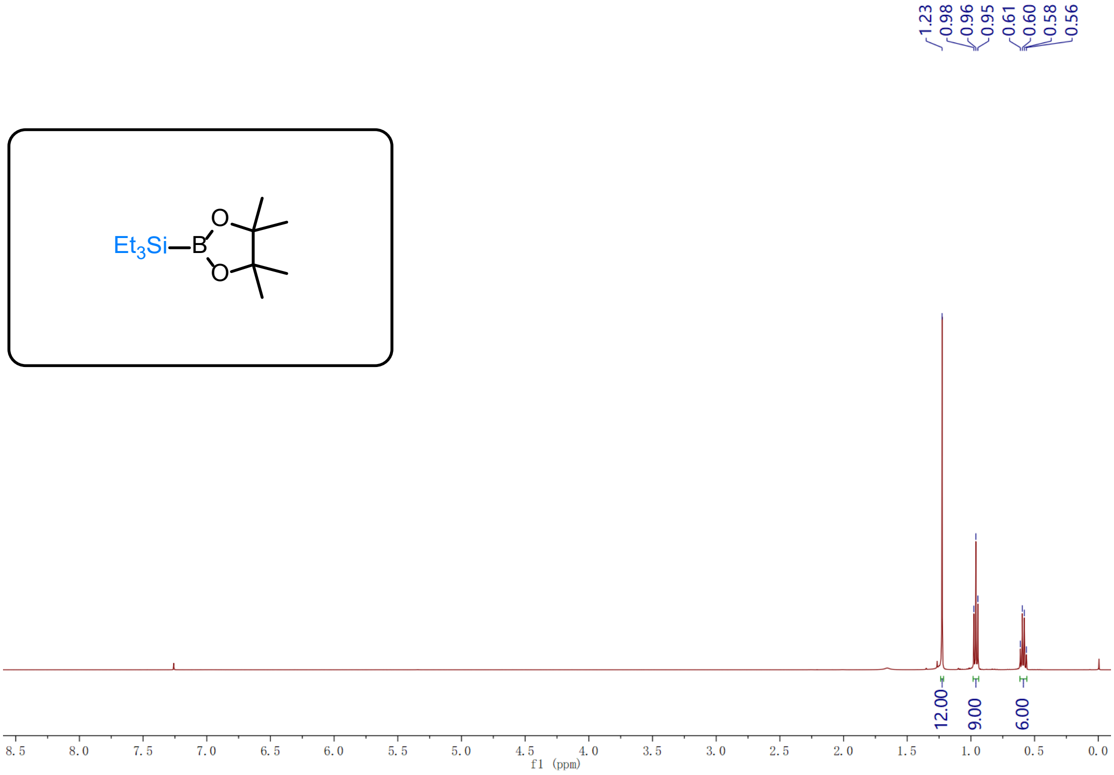

8.2 NMR spectra of products

^{1}H NMR spectrum ($CDCl_3$) of **3a**

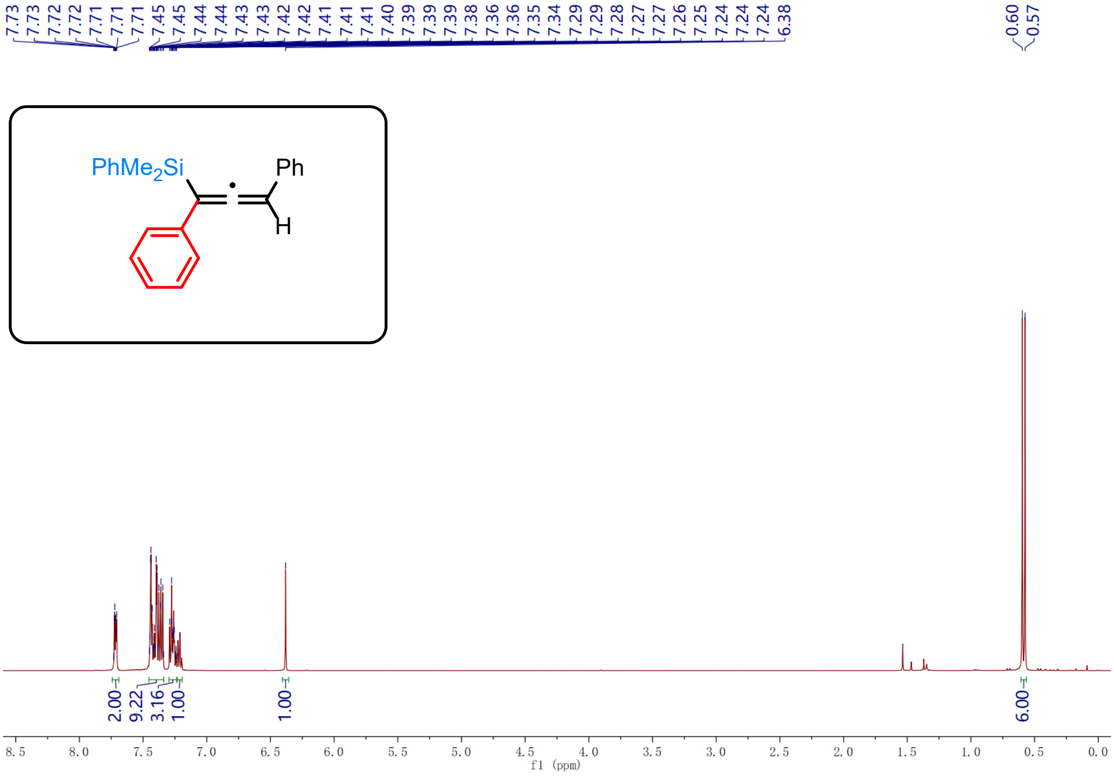

^{13}C NMR spectrum ($CDCl_3$) of **3a**

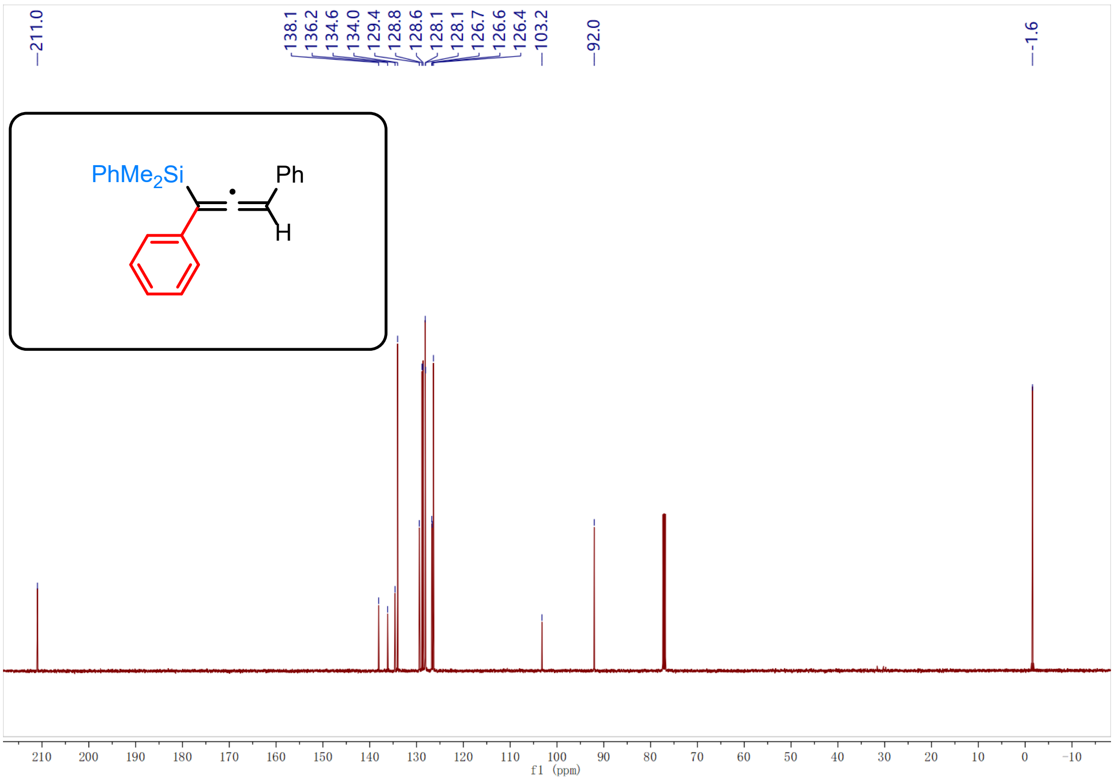

^{1}H NMR spectrum ($CDCl_3$) of **3b**

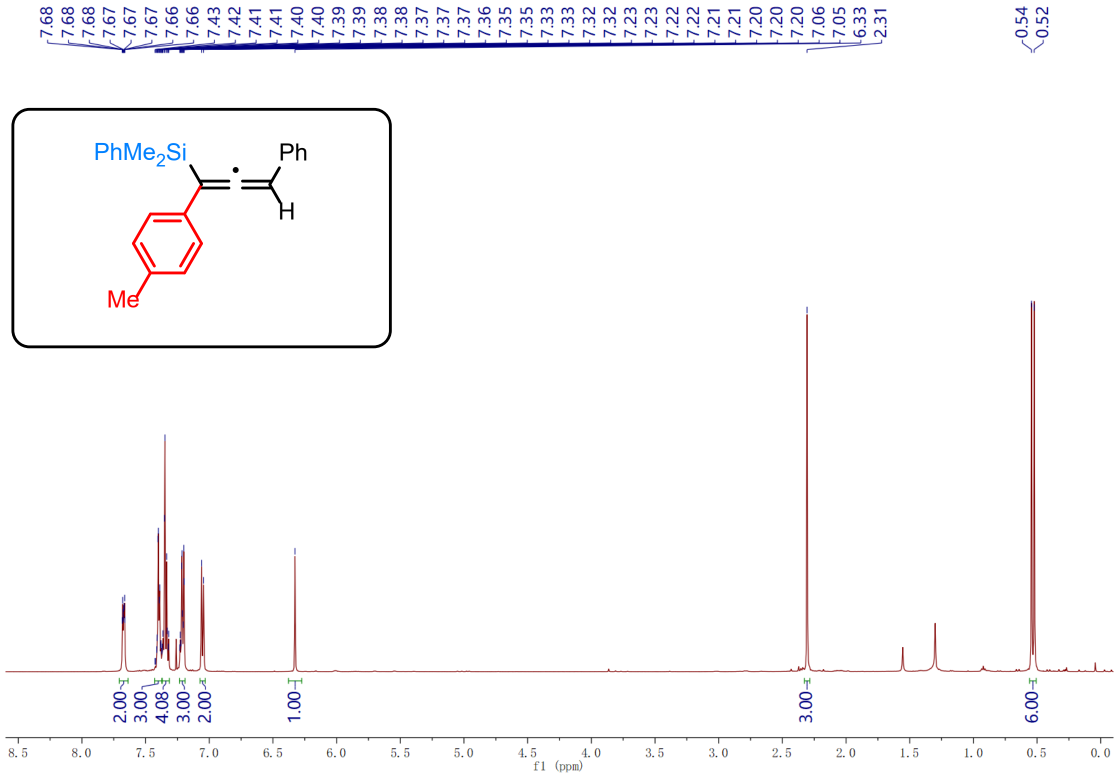

^{13}C NMR spectrum ($CDCl_3$) of **3b**

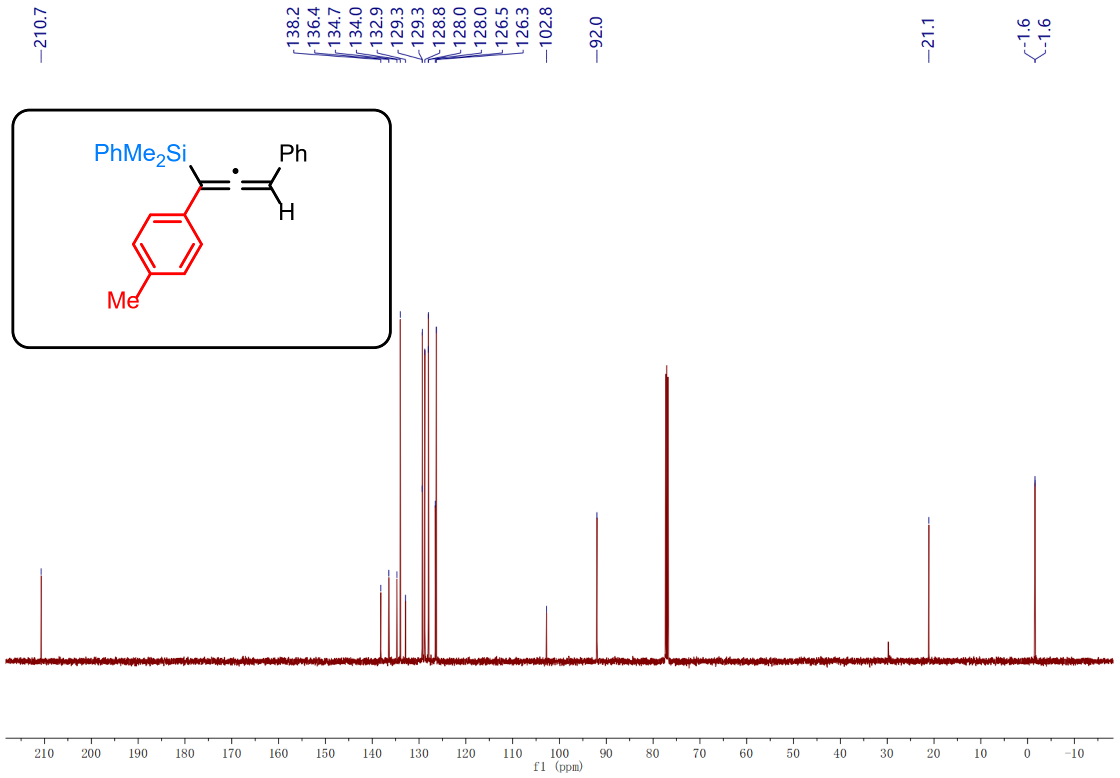

^{1}H NMR spectrum ($CDCl_3$) of **3c**

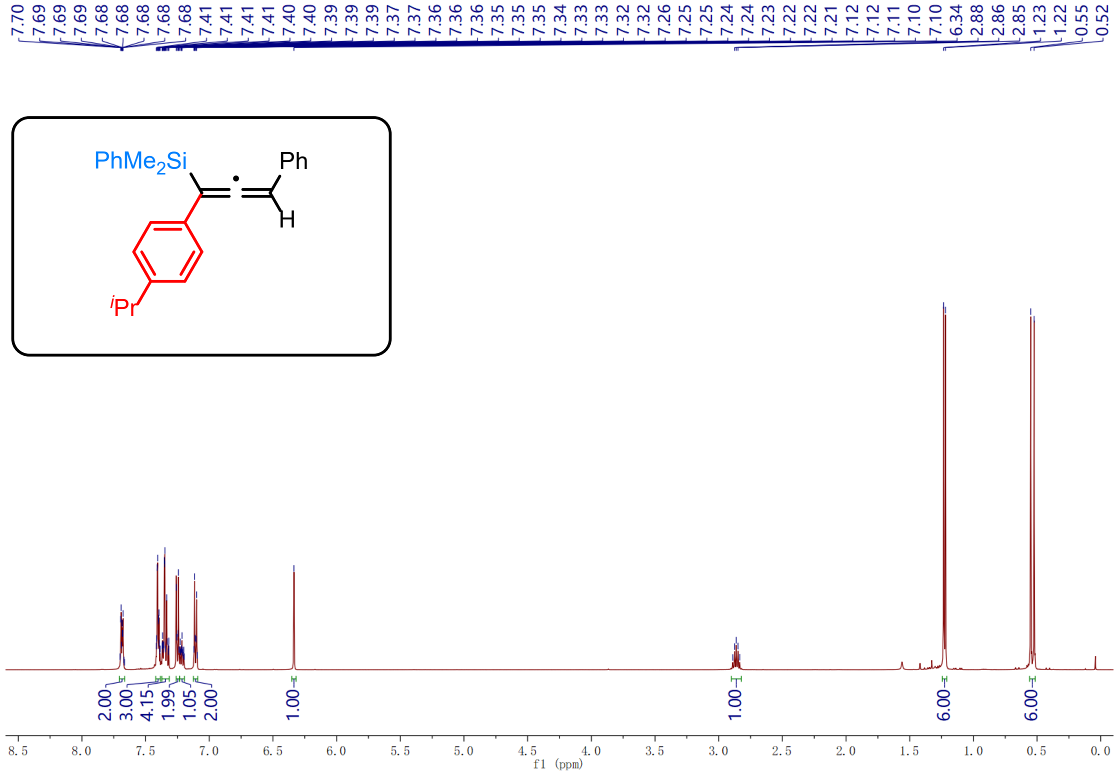

^{13}C NMR spectrum ($CDCl_3$) of **3c**

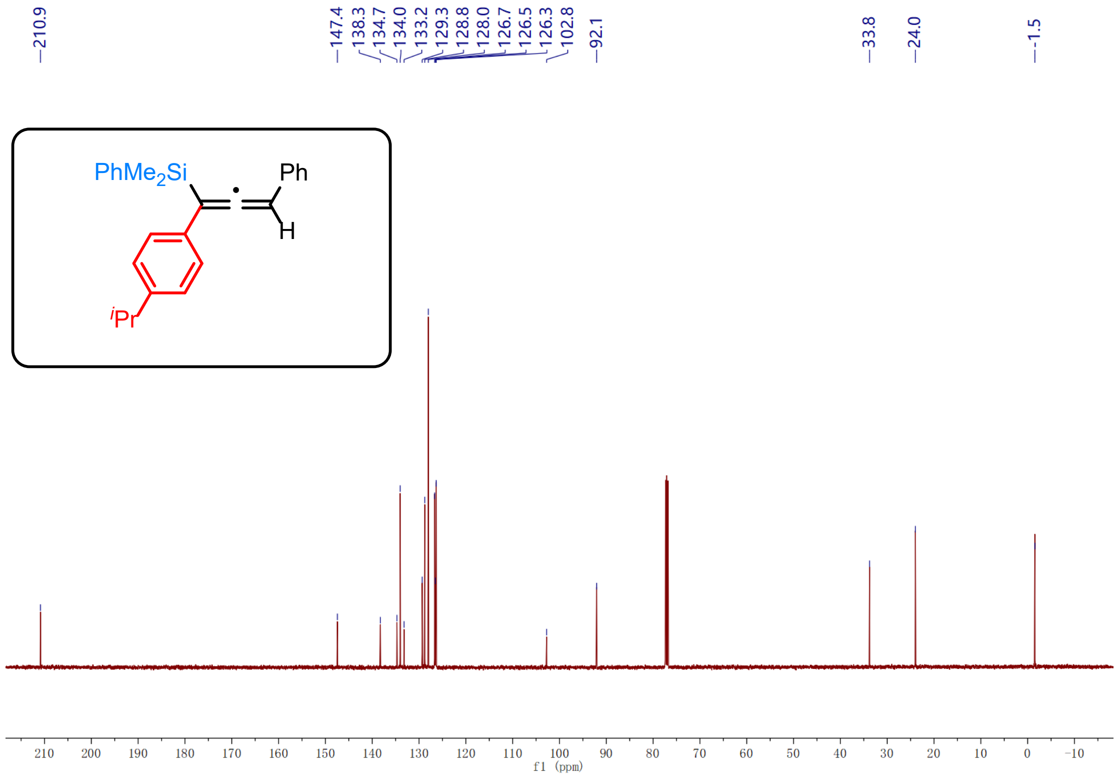

^{1}H NMR spectrum ($CDCl_3$) of **3d**

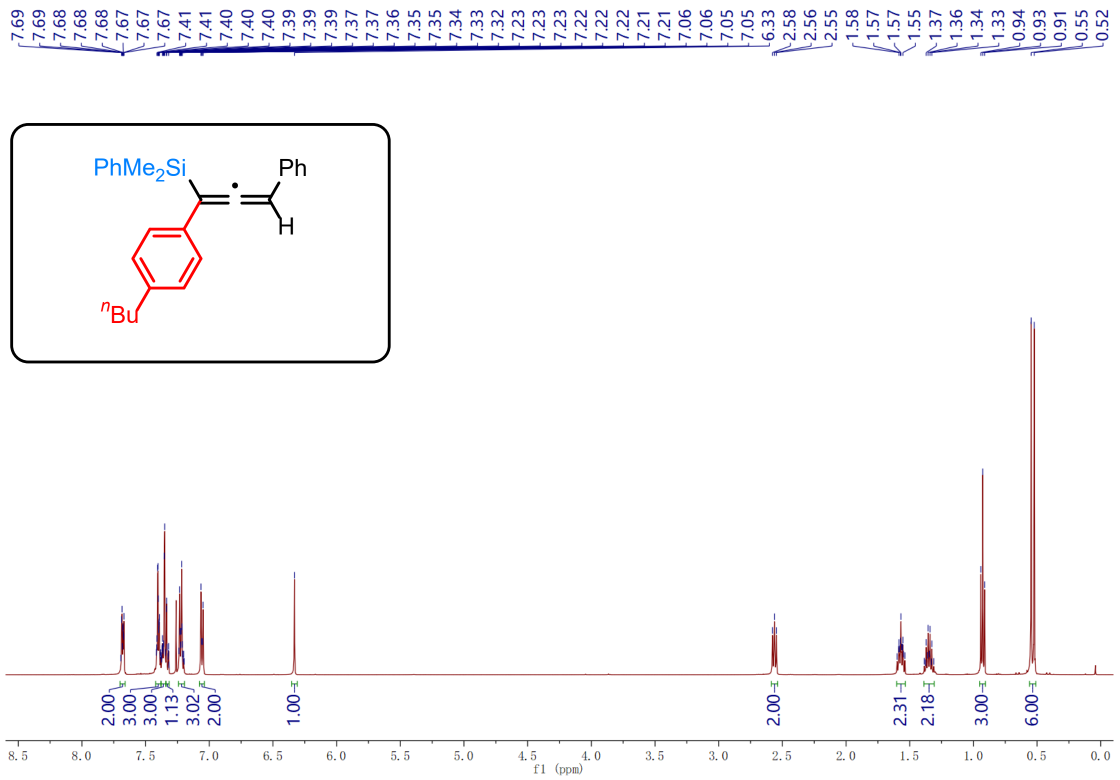

^{13}C NMR spectrum ($CDCl_3$) of **3d**

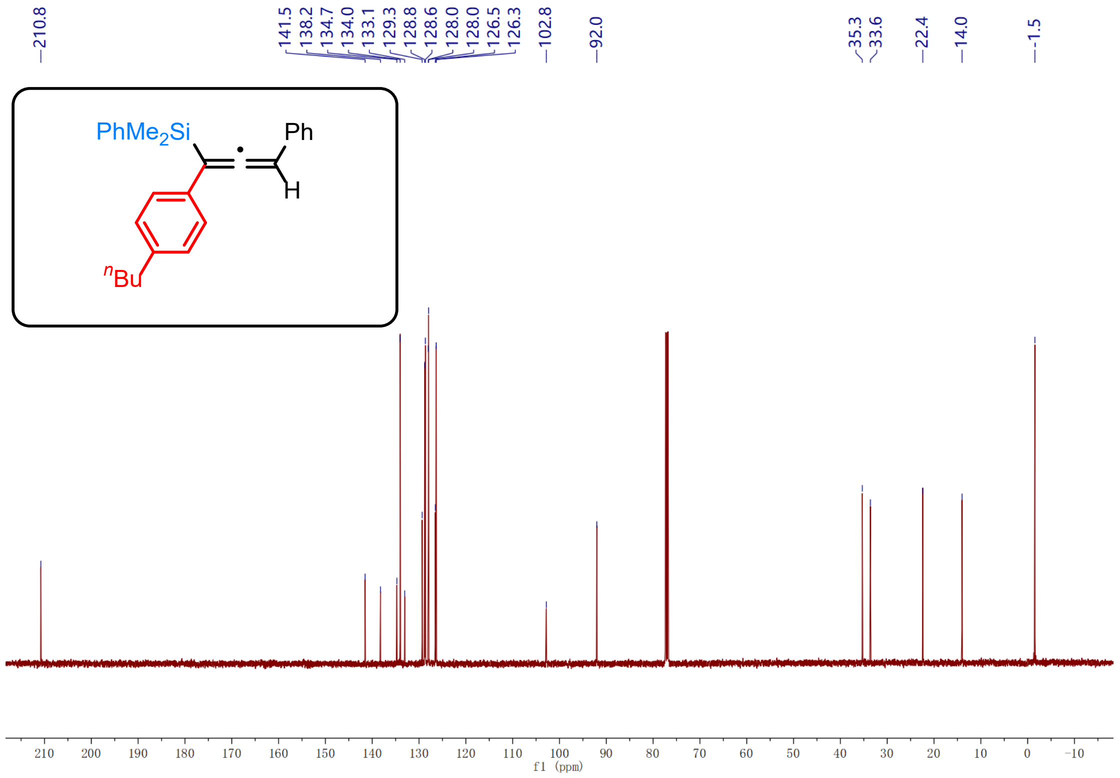

^{1}H NMR spectrum ($CDCl_3$) of **3e**

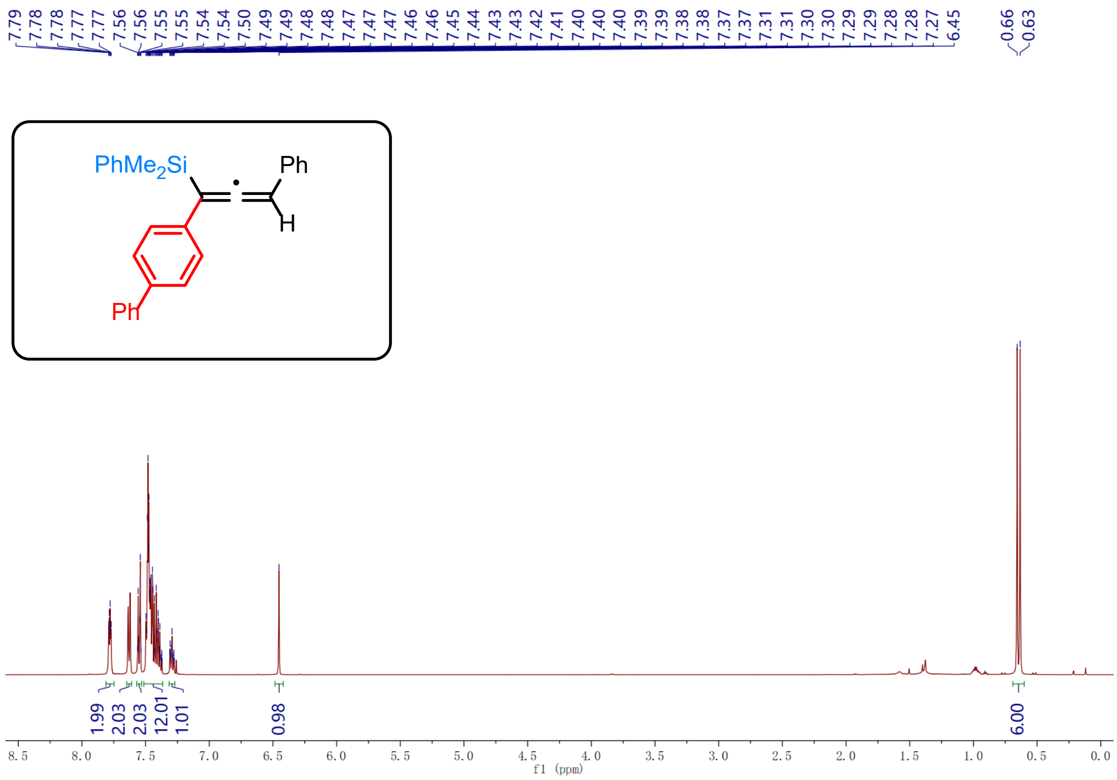

^{13}C NMR spectrum ($CDCl_3$) of **3e**

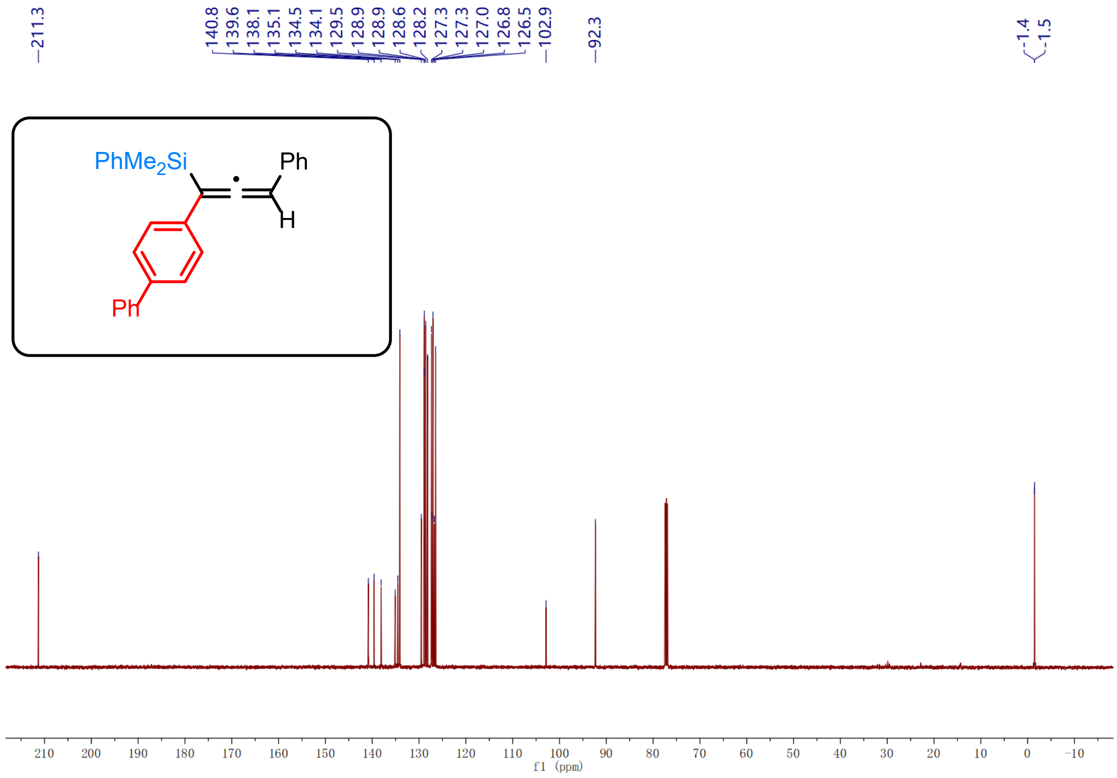

^{1}H NMR spectrum ($CDCl_3$) of **3f**

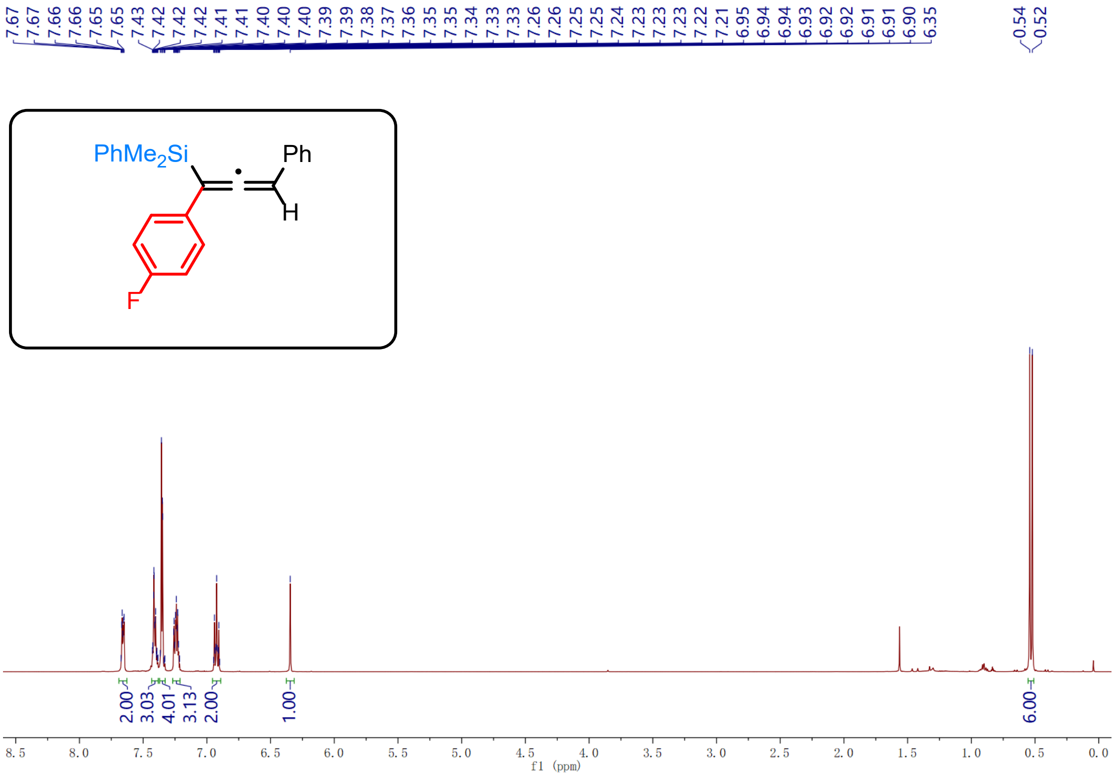

^{13}C NMR spectrum ($CDCl_3$) of **3f**

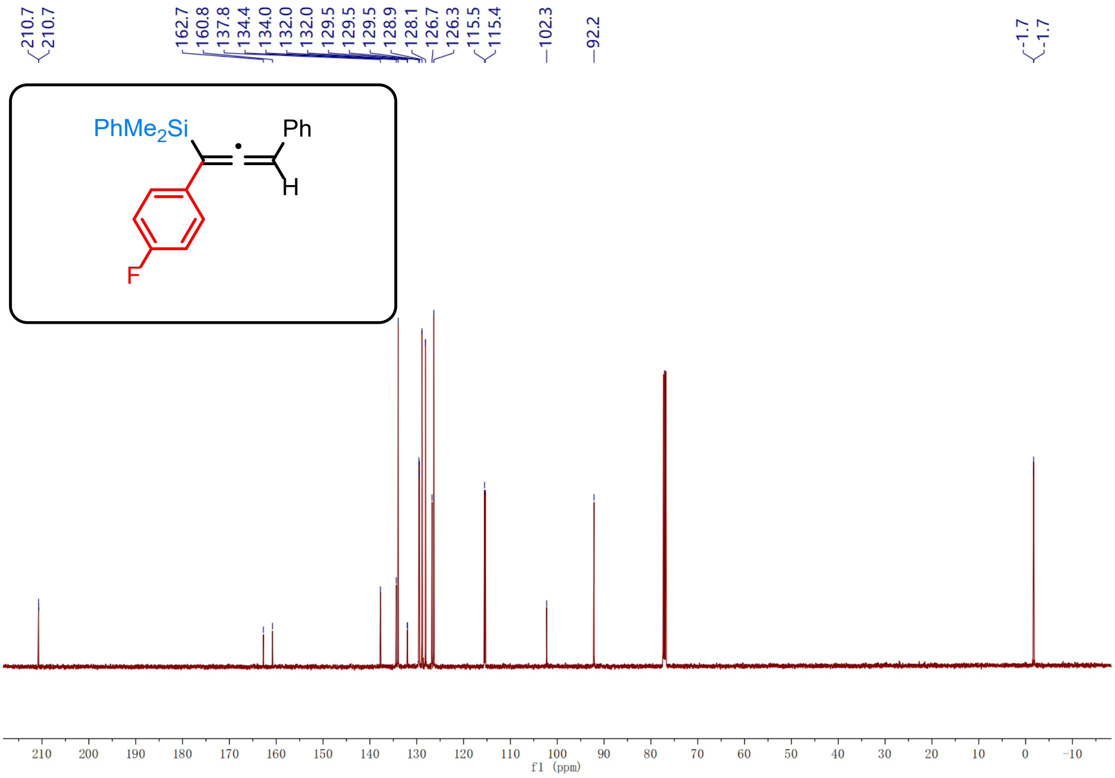

^{19}F NMR spectrum ($CDCl_3$) of 3**f**

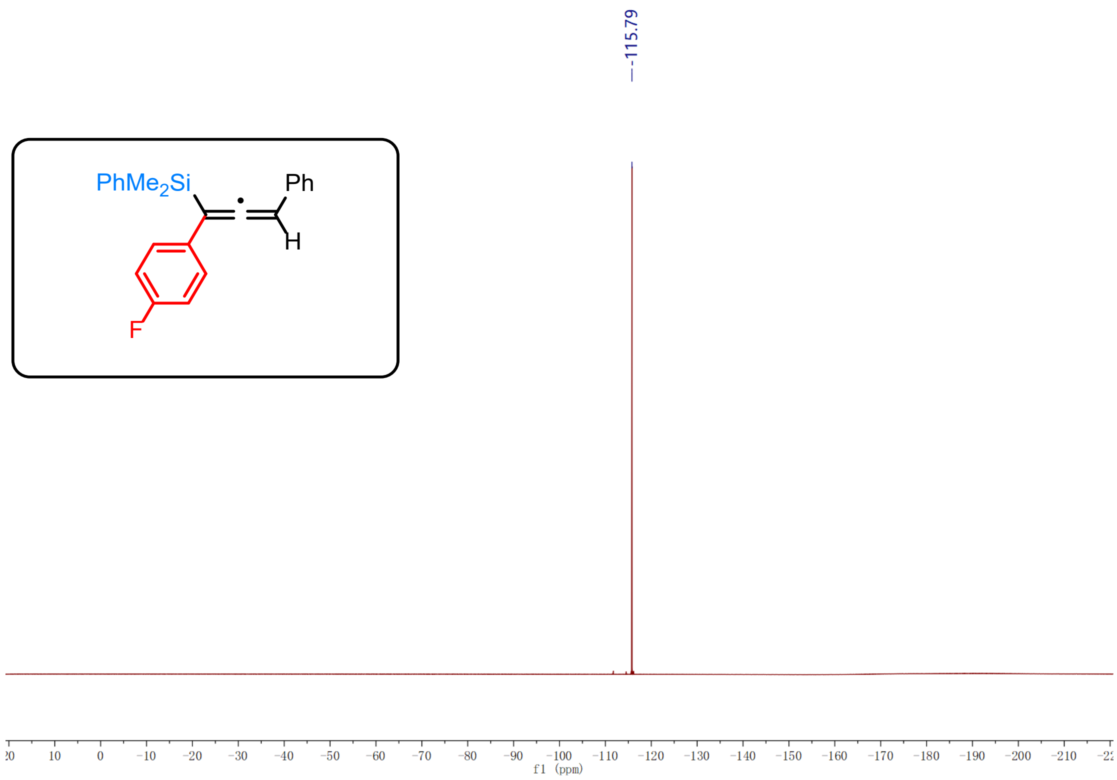

^{1}H NMR spectrum ($CDCl_3$) of **3g**

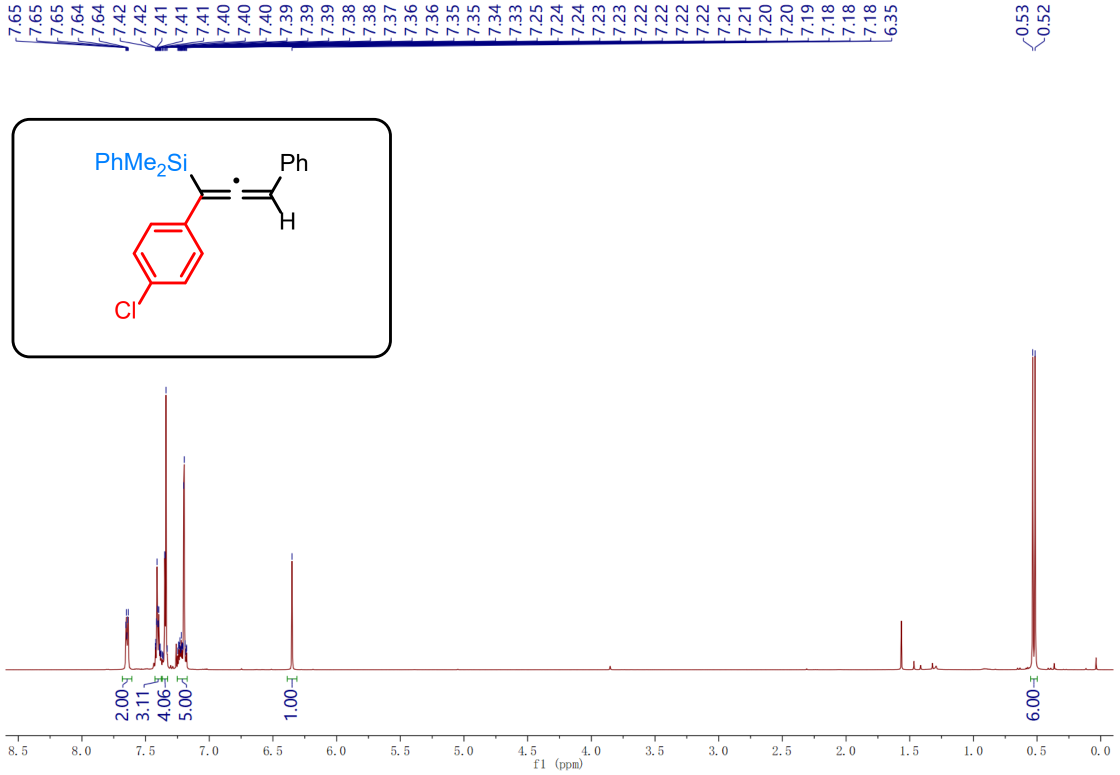

^{13}C NMR spectrum ($CDCl_3$) of **3g**

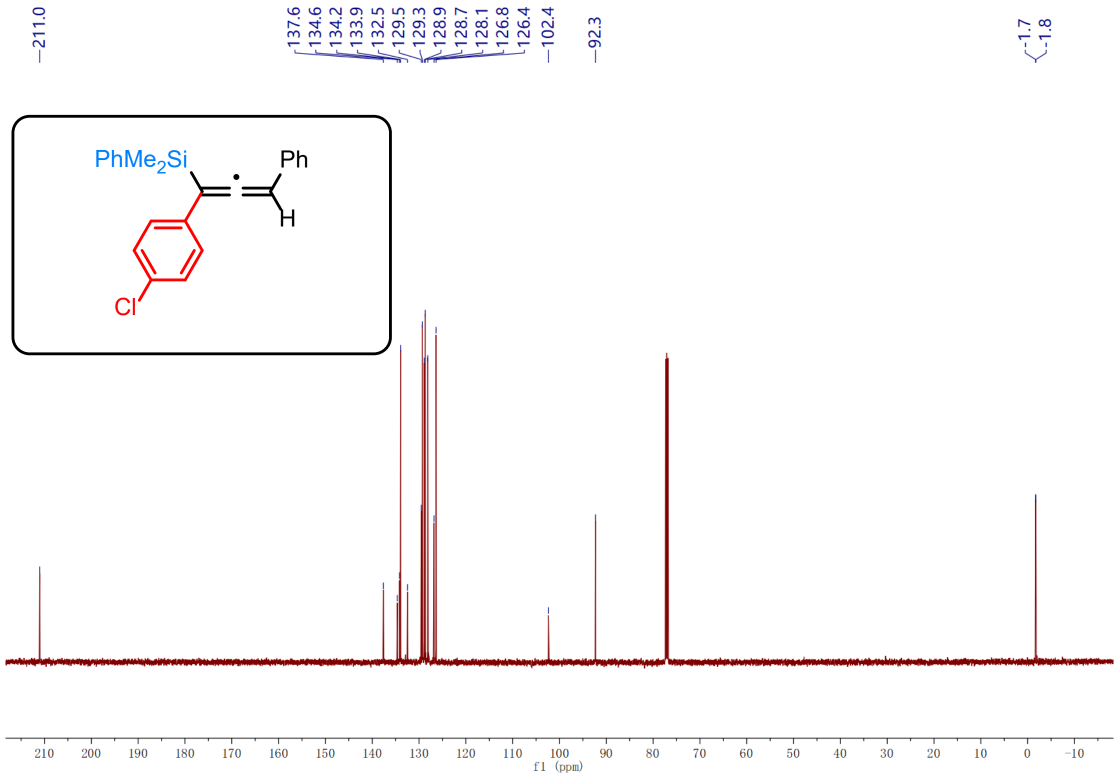

^{1}H NMR spectrum ($CDCl_3$) of **3h**

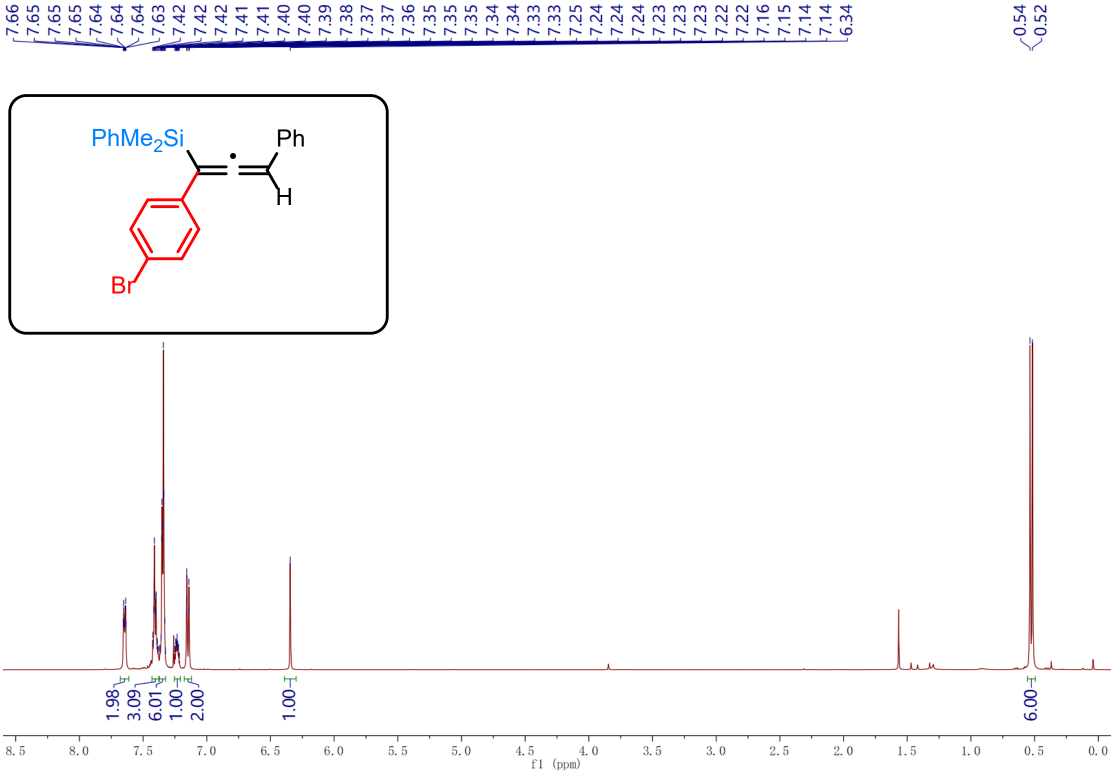

^{13}C NMR spectrum ($CDCl_3$) of **3h**

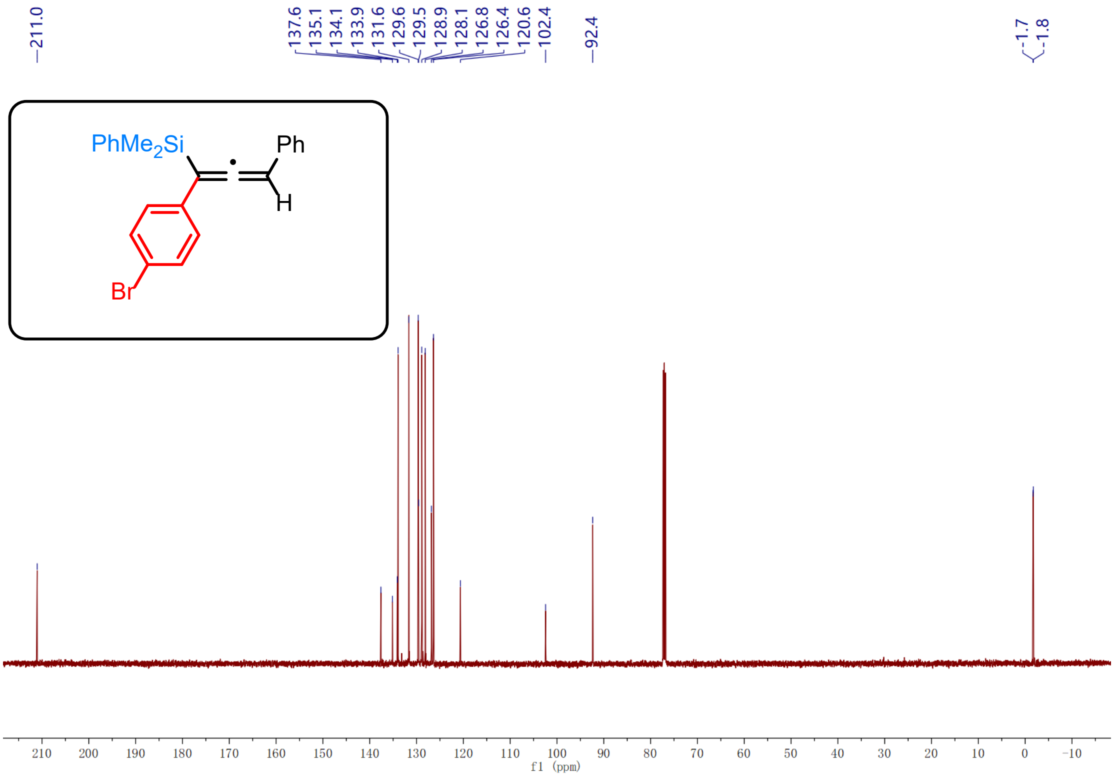

^{1}H NMR spectrum ($CDCl_3$) of **3i**

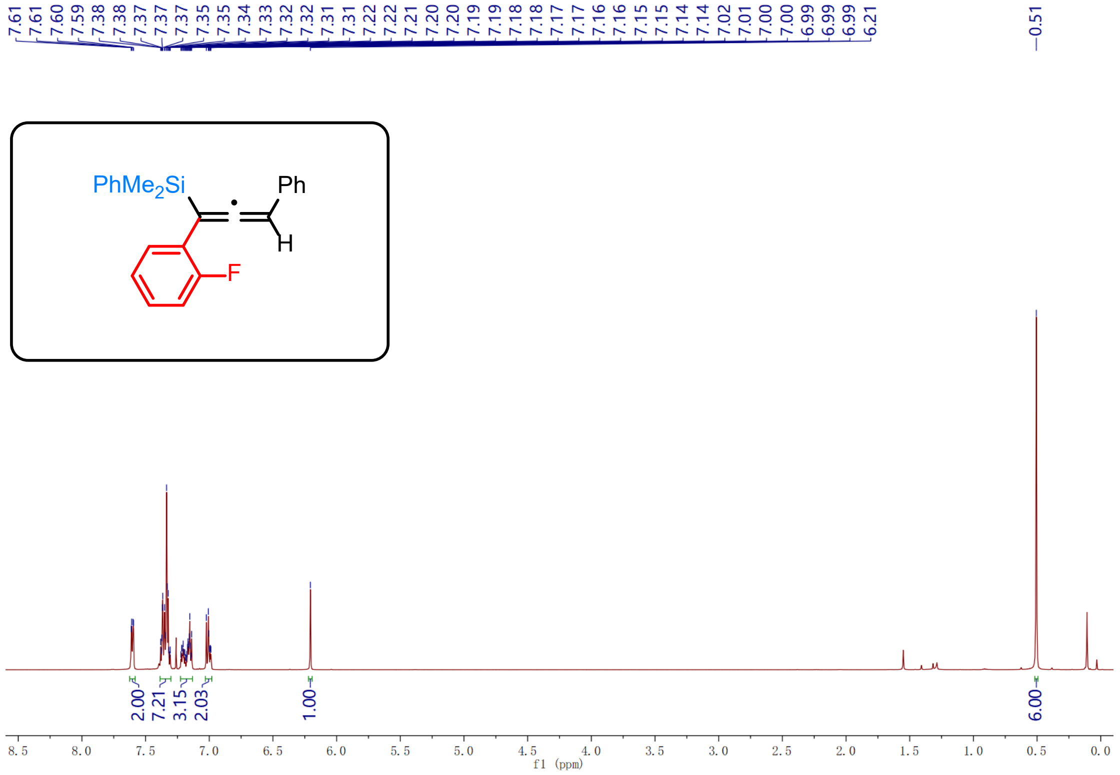

^{13}C NMR spectrum ($CDCl_3$) of **3i**

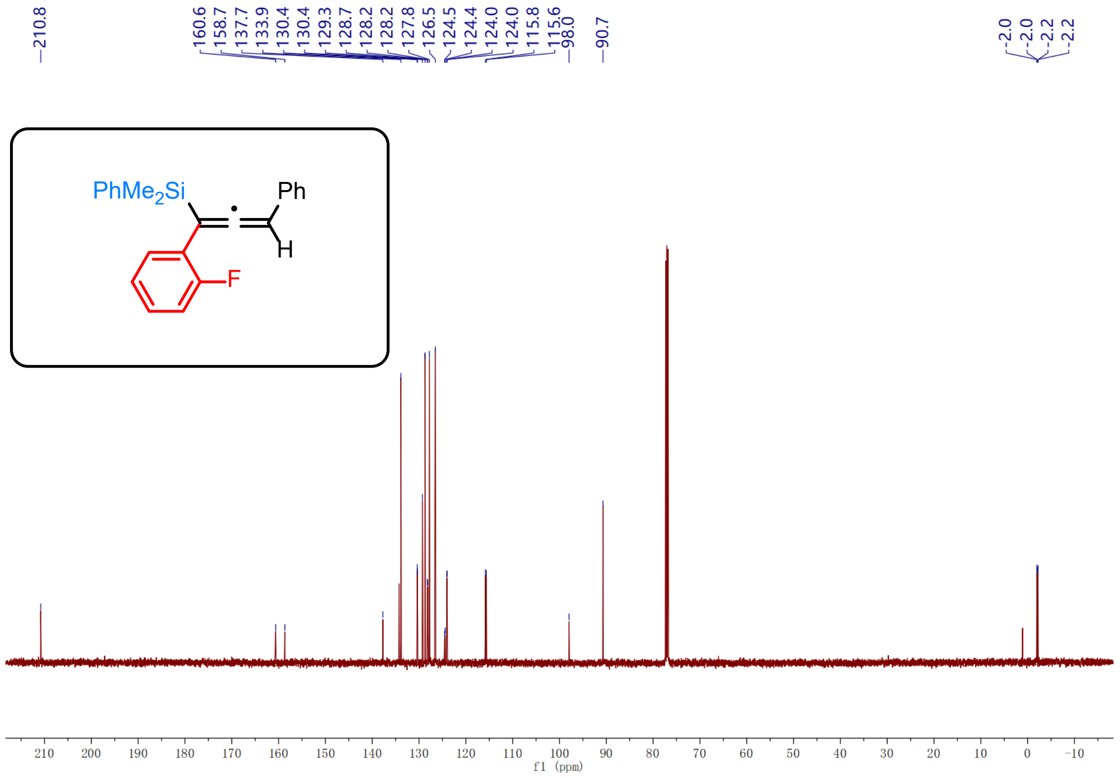

^{19}F NMR spectrum ($CDCl_3$) of **3i**

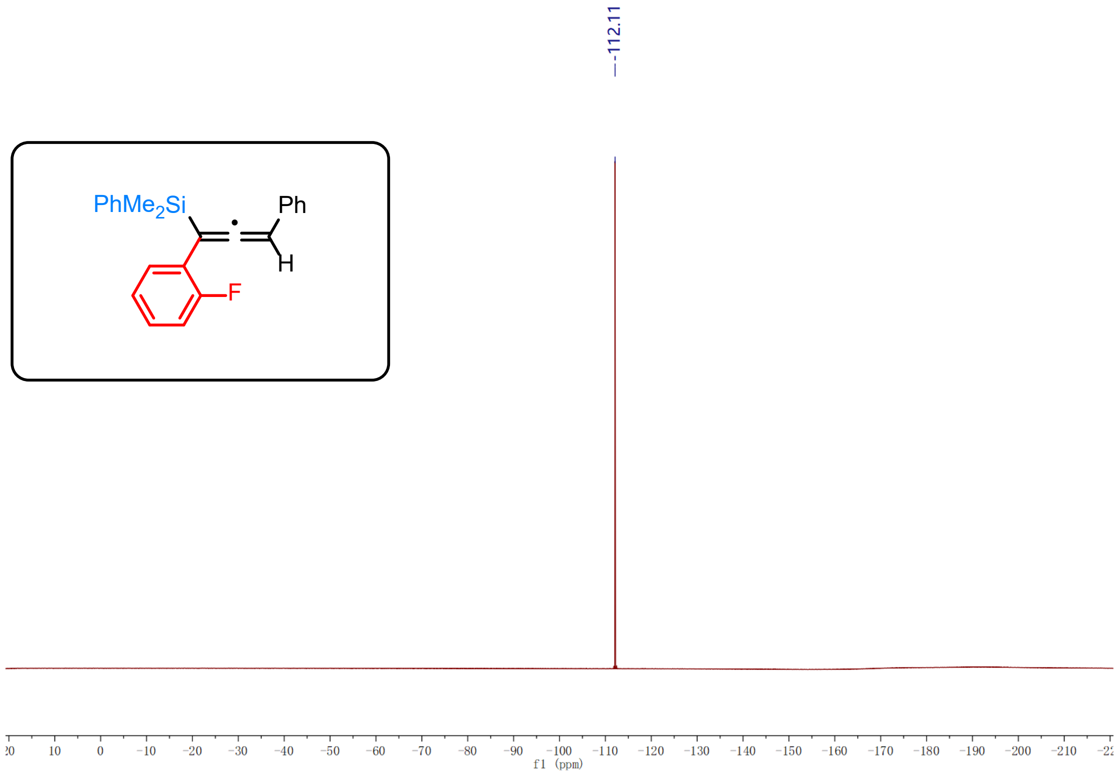

^{1}H NMR spectrum ($CDCl_3$) of **3j**

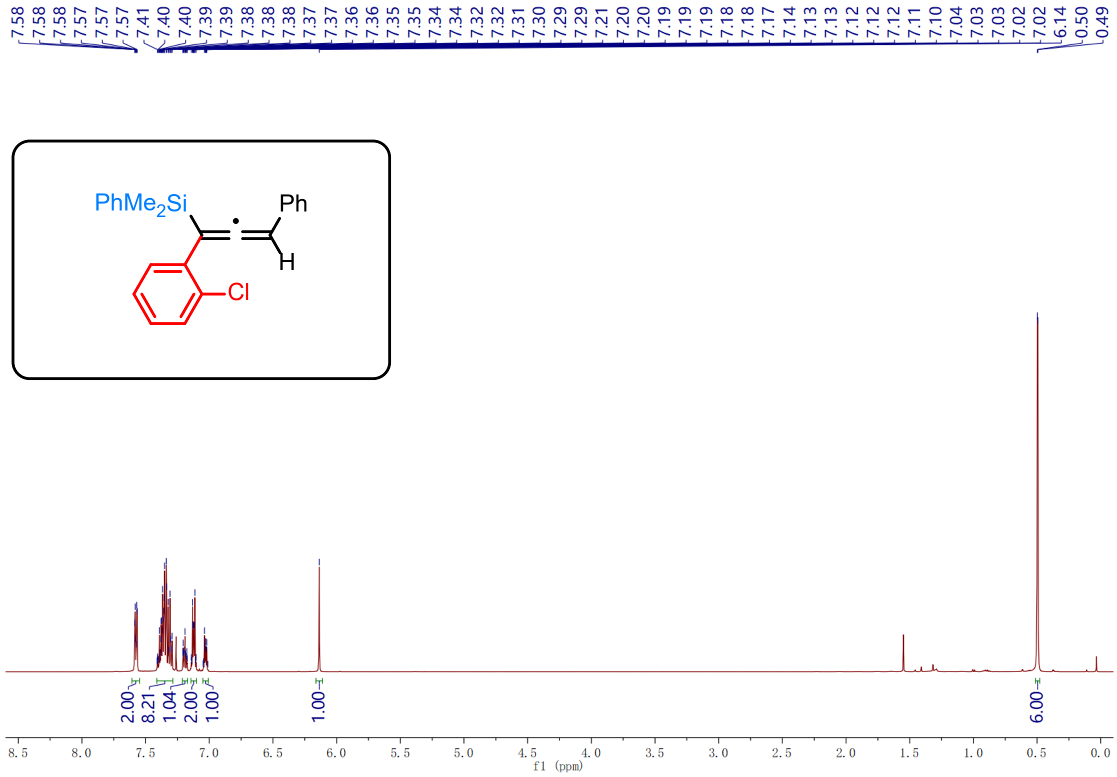

^{13}C NMR spectrum ($CDCl_3$) of **3j**

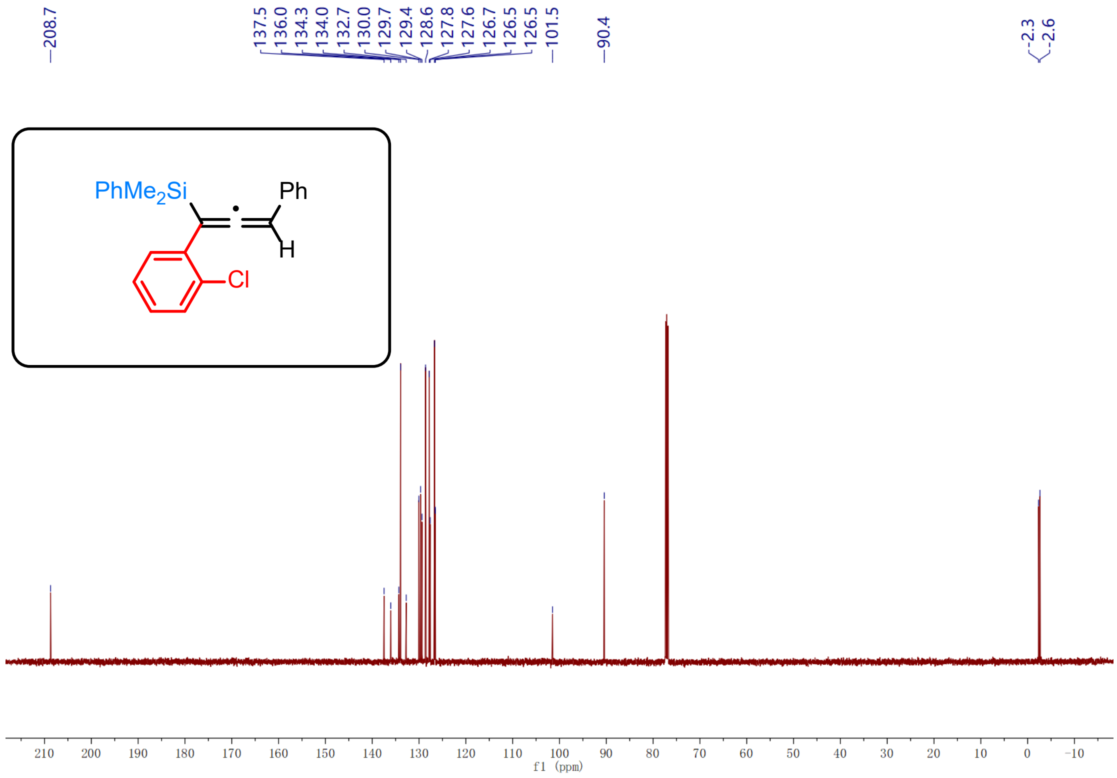

^{1}H NMR spectrum ($CDCl_3$) of **3k**

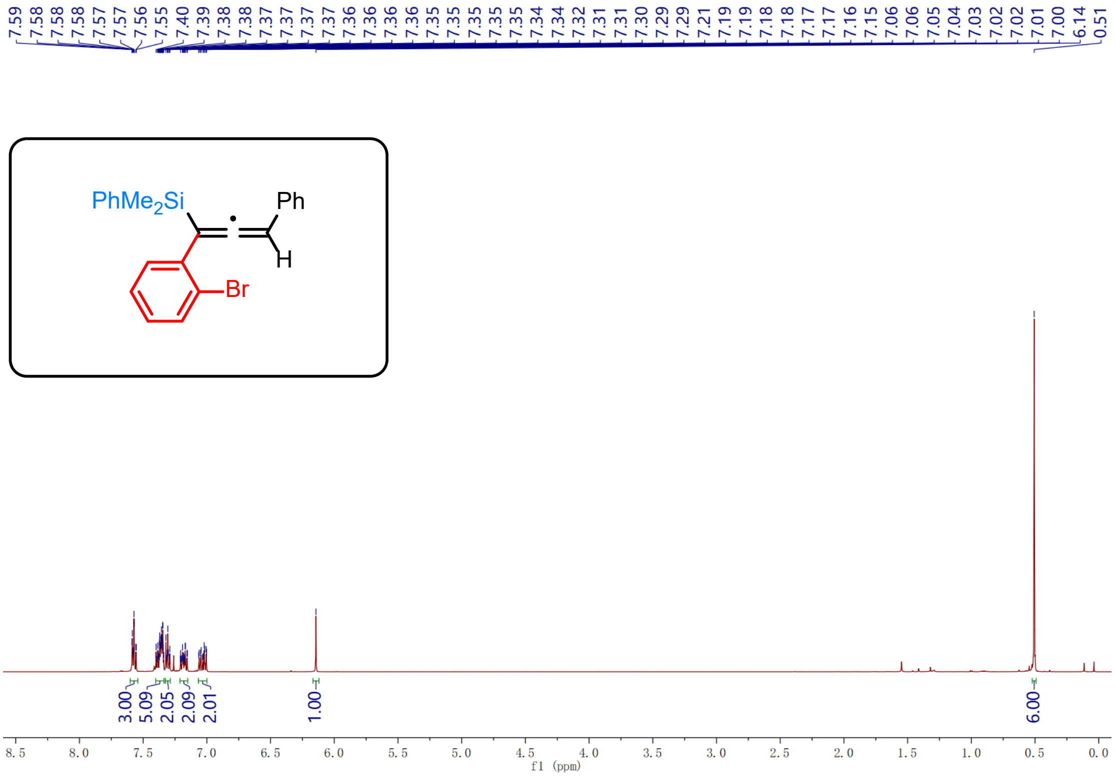

^{13}C NMR spectrum ($CDCl_3$) of **3k**

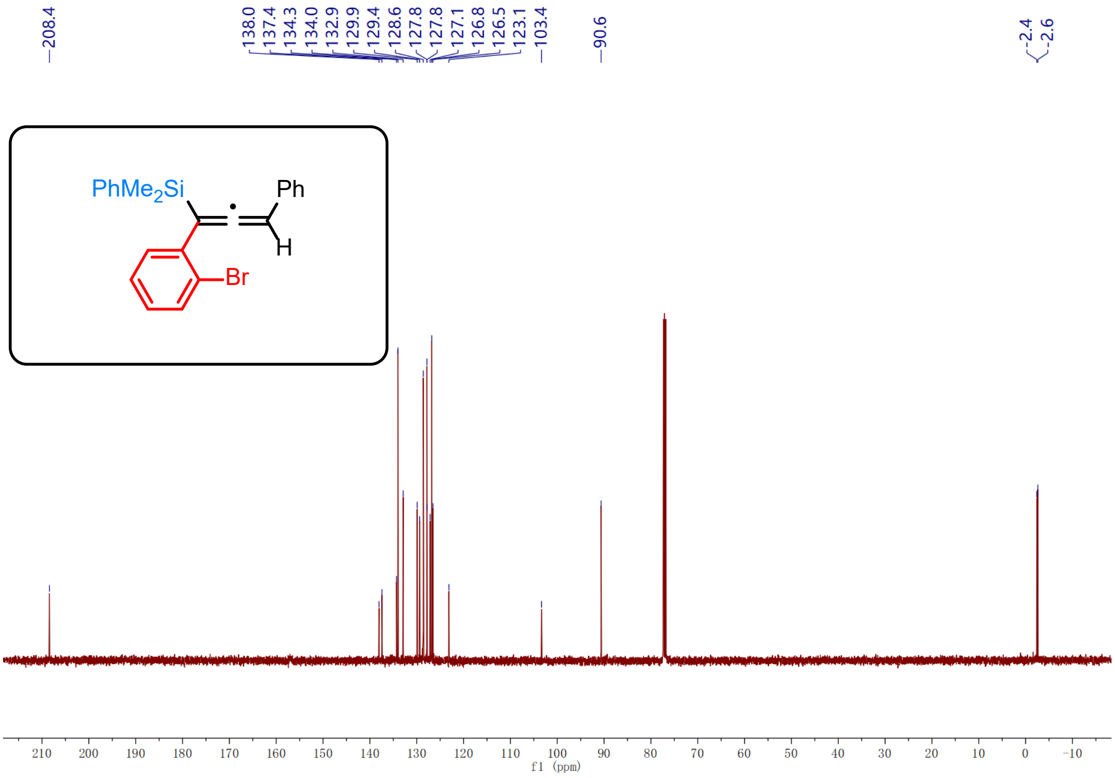

^{1}H NMR spectrum ($CDCl_3$) of **3l**

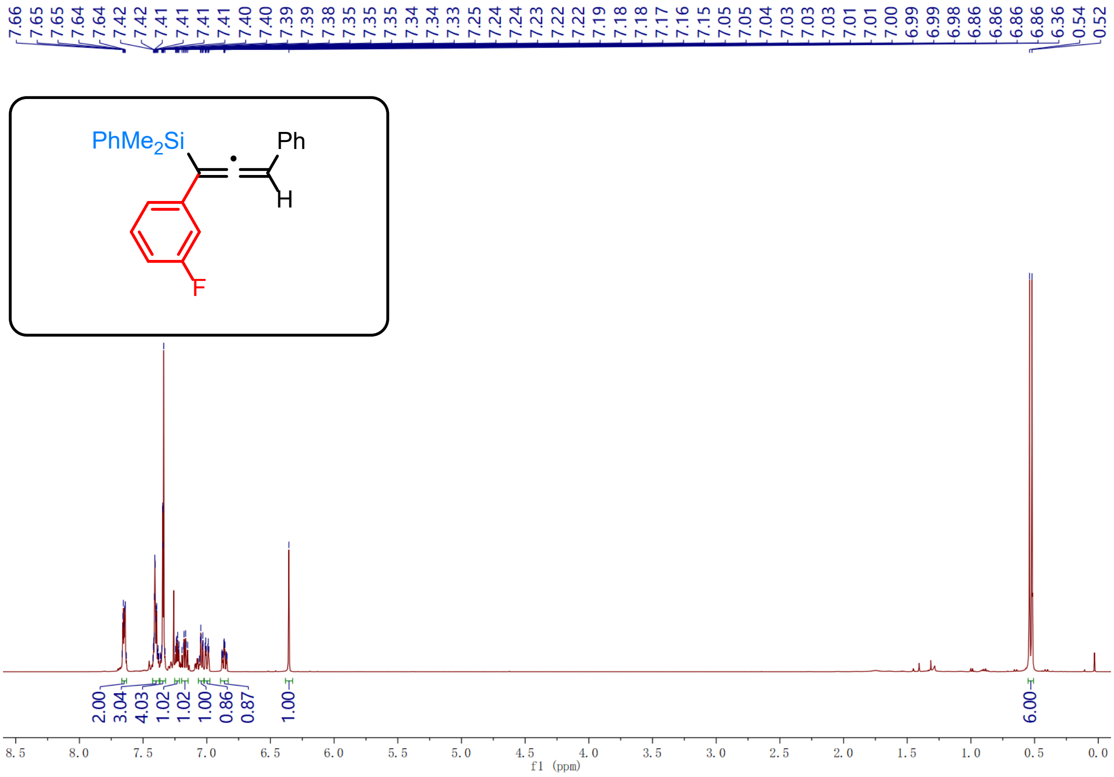

^{13}C NMR spectrum ($CDCl_3$) of **3l**

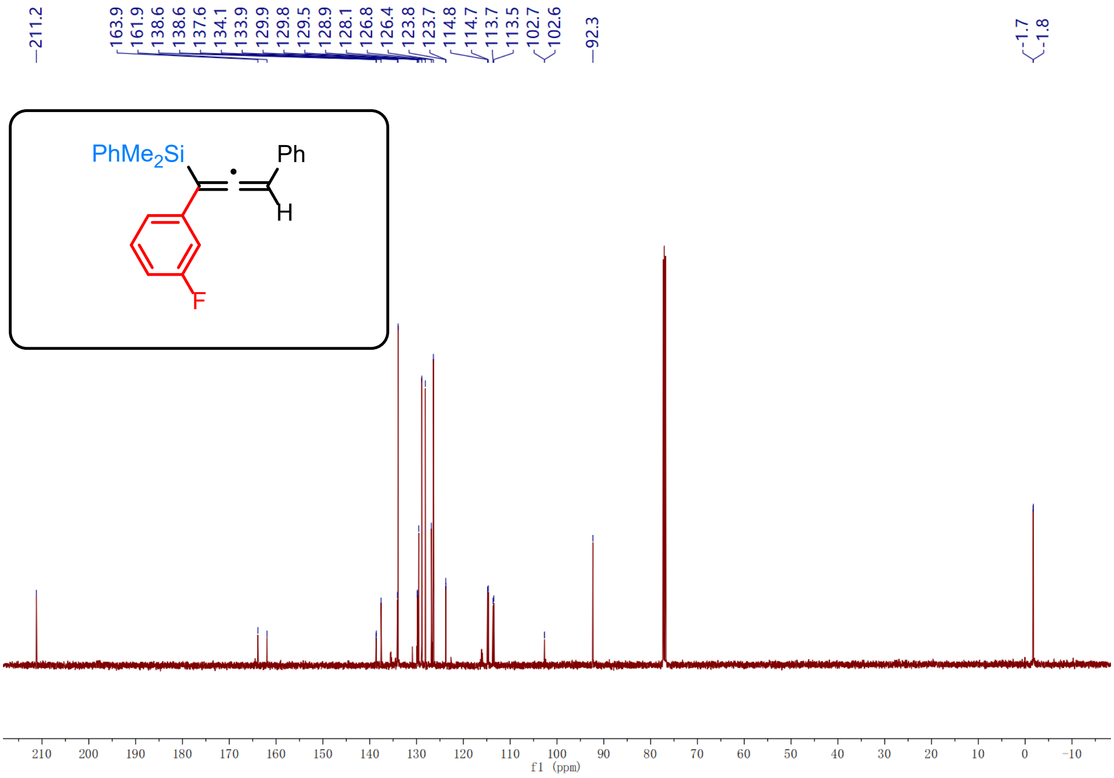

^{19}F NMR spectrum ($CDCl_3$) of **3l**

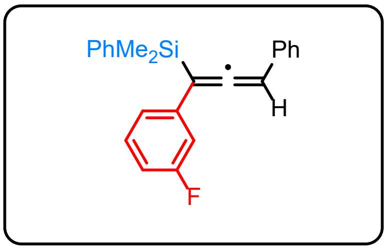

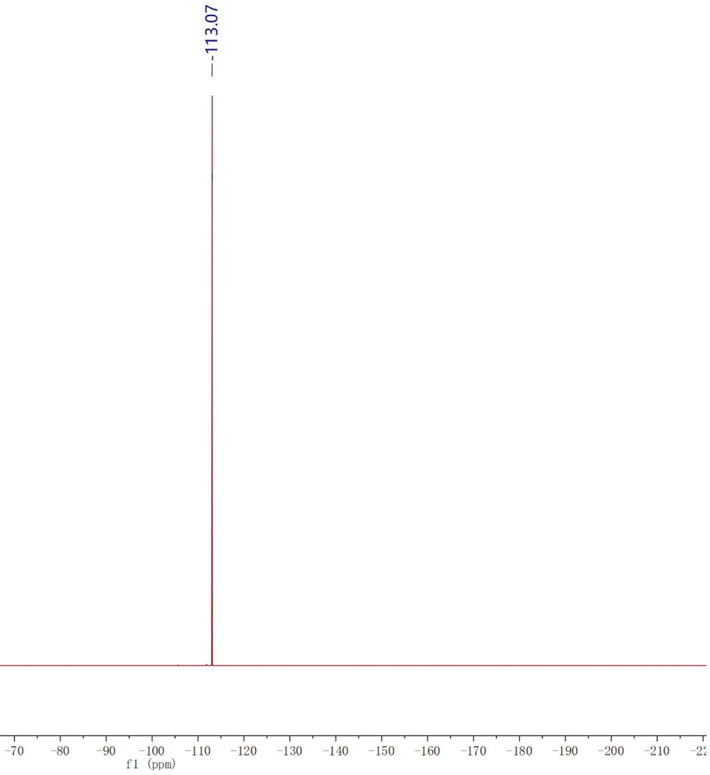

^{1}H NMR spectrum ($CDCl_3$) of **3m**

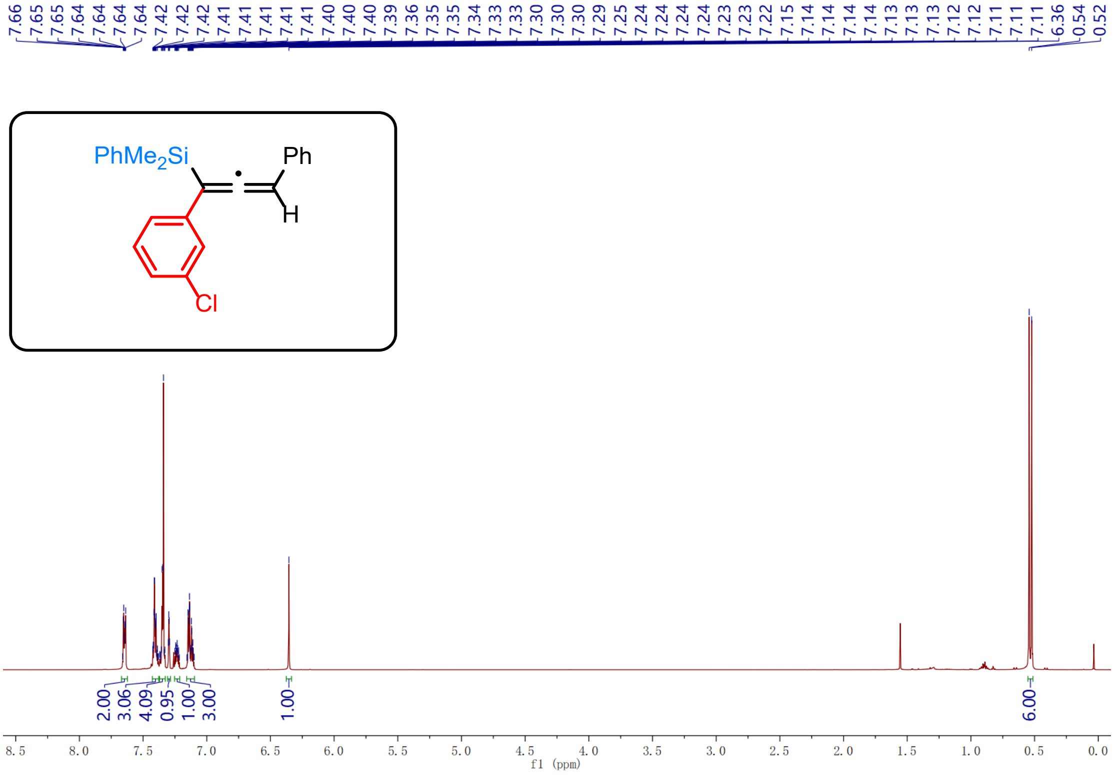

^{13}C NMR spectrum ($CDCl_3$) of **3m**

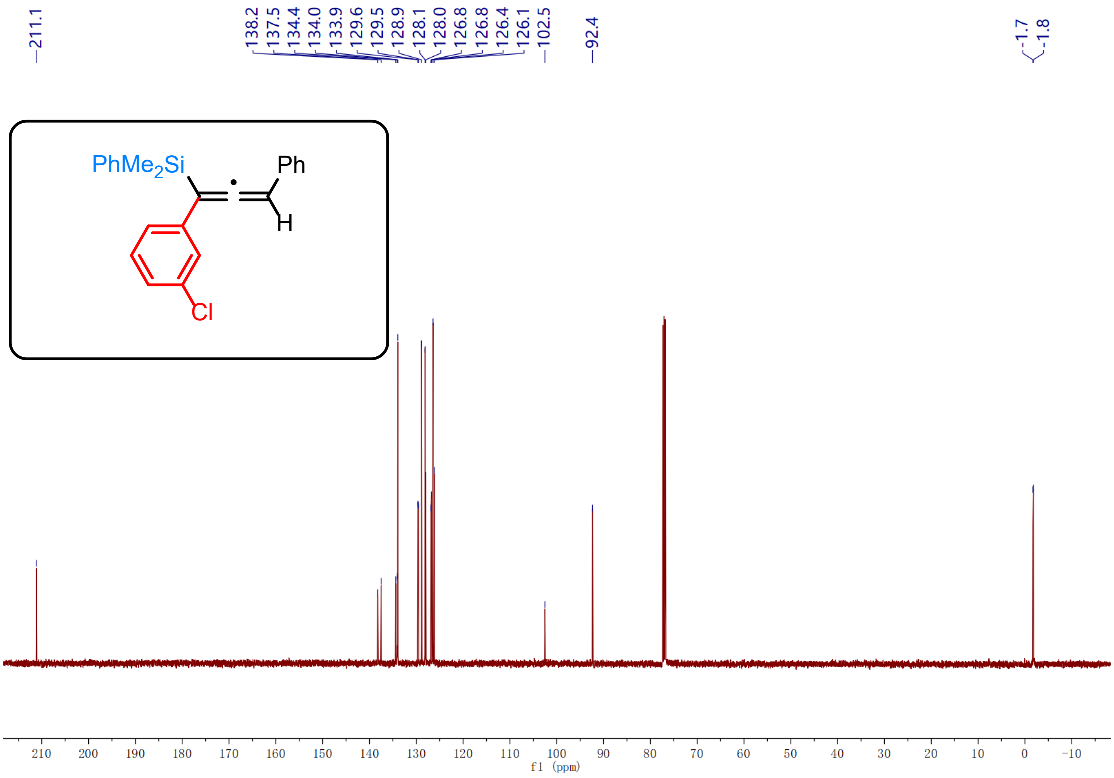

^{1}H NMR spectrum ($CDCl_3$) of **3n**

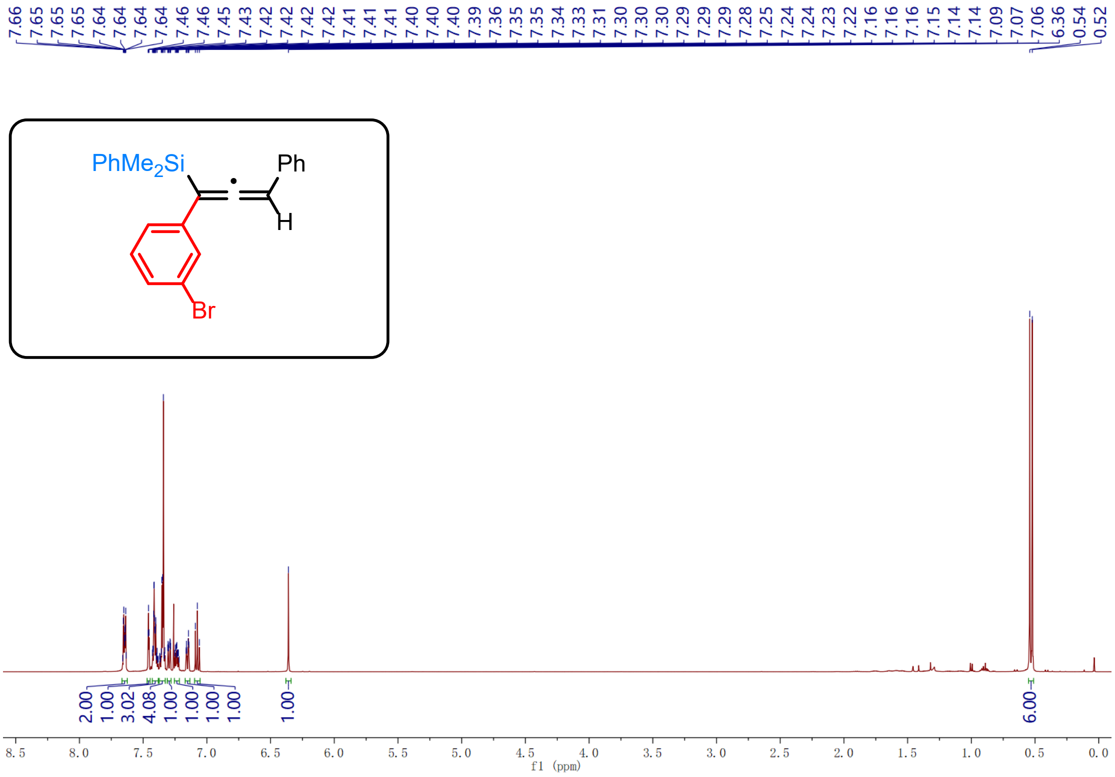

^{13}C NMR spectrum ($CDCl_3$) of **3n**

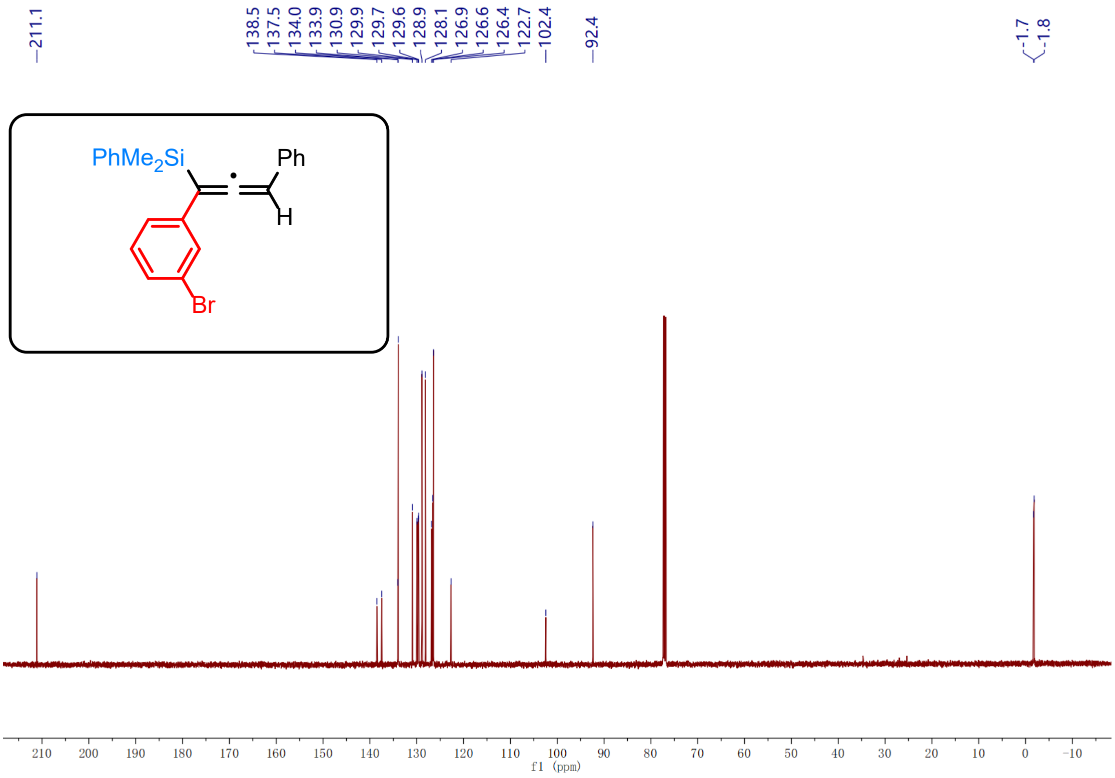

^{1}H NMR spectrum ($CDCl_3$) of **3o**

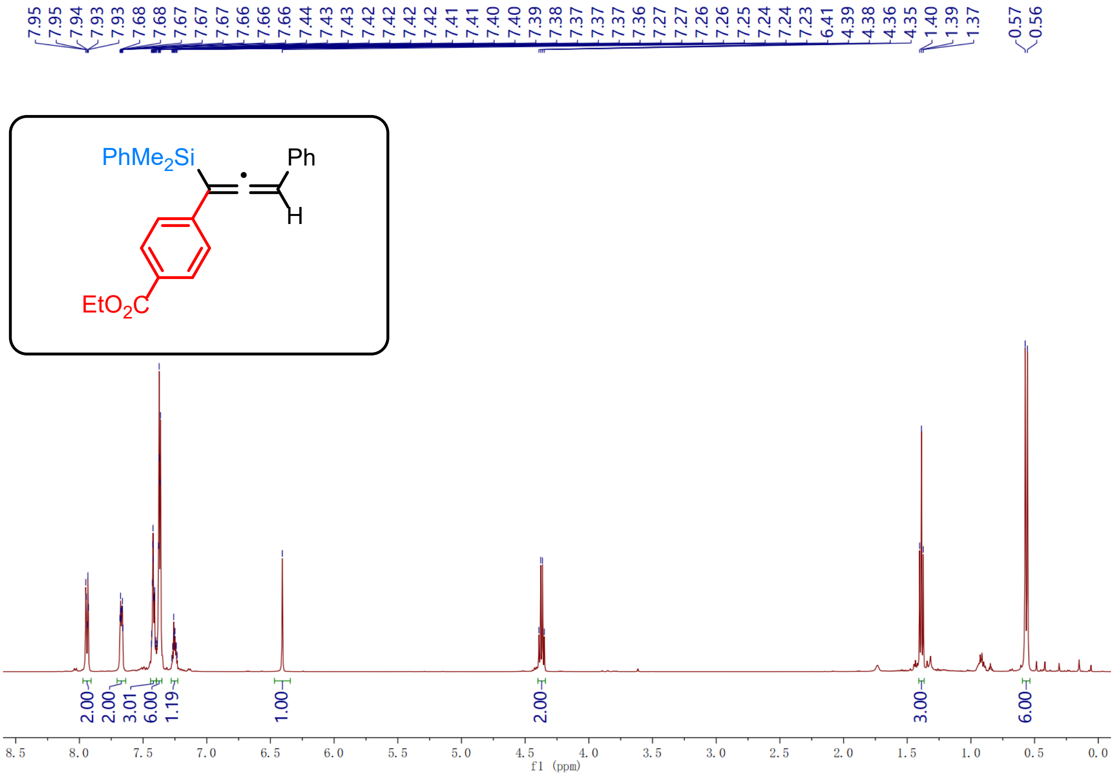

^{13}C NMR spectrum ($CDCl_3$) of **3o**

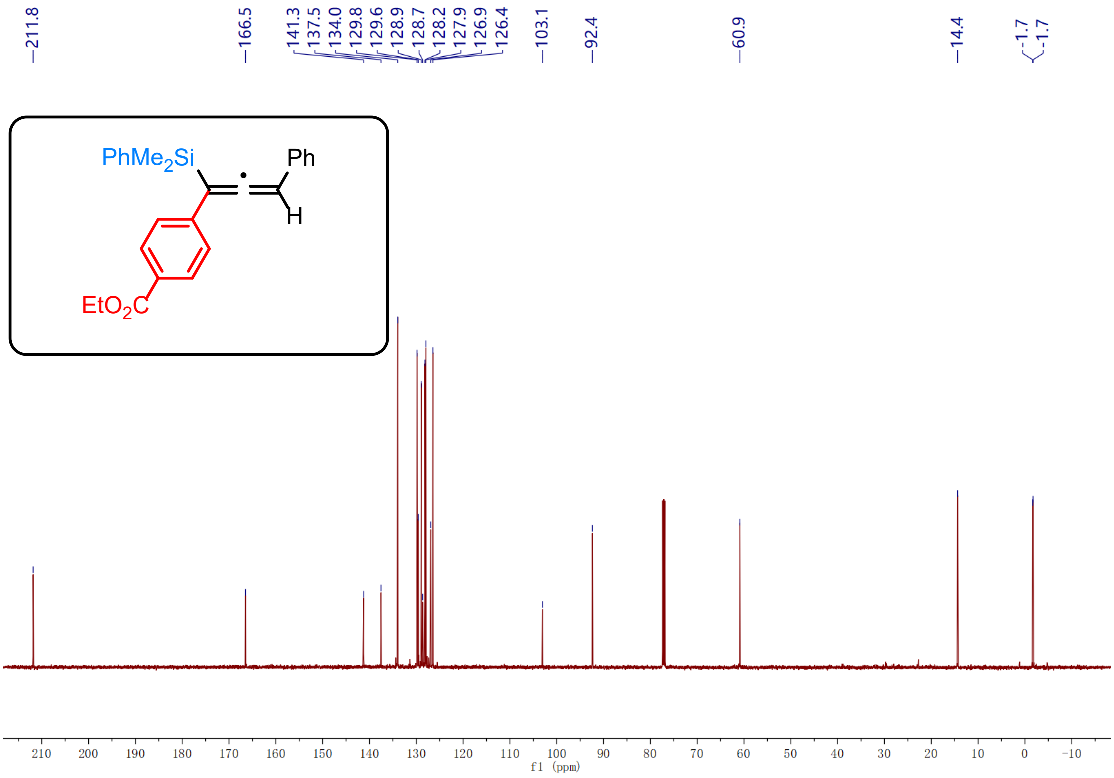

^{1}H NMR spectrum ($CDCl_3$) of **3p**

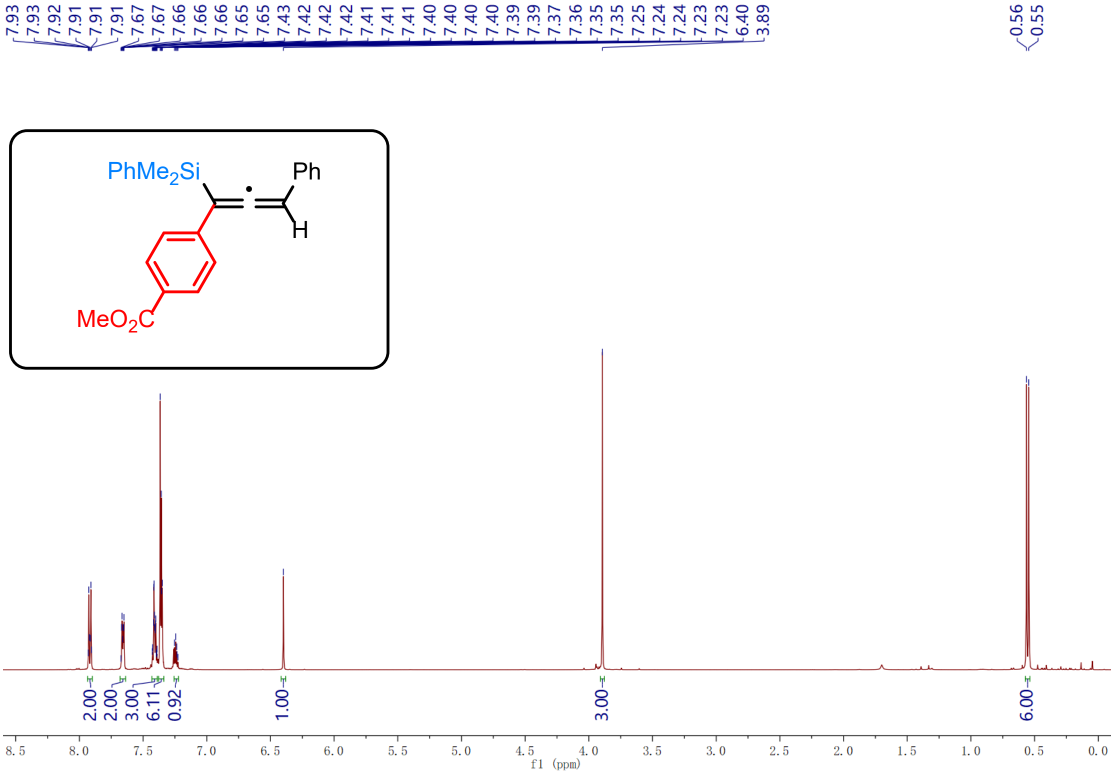

^{13}C NMR spectrum ($CDCl_3$) of **3p**

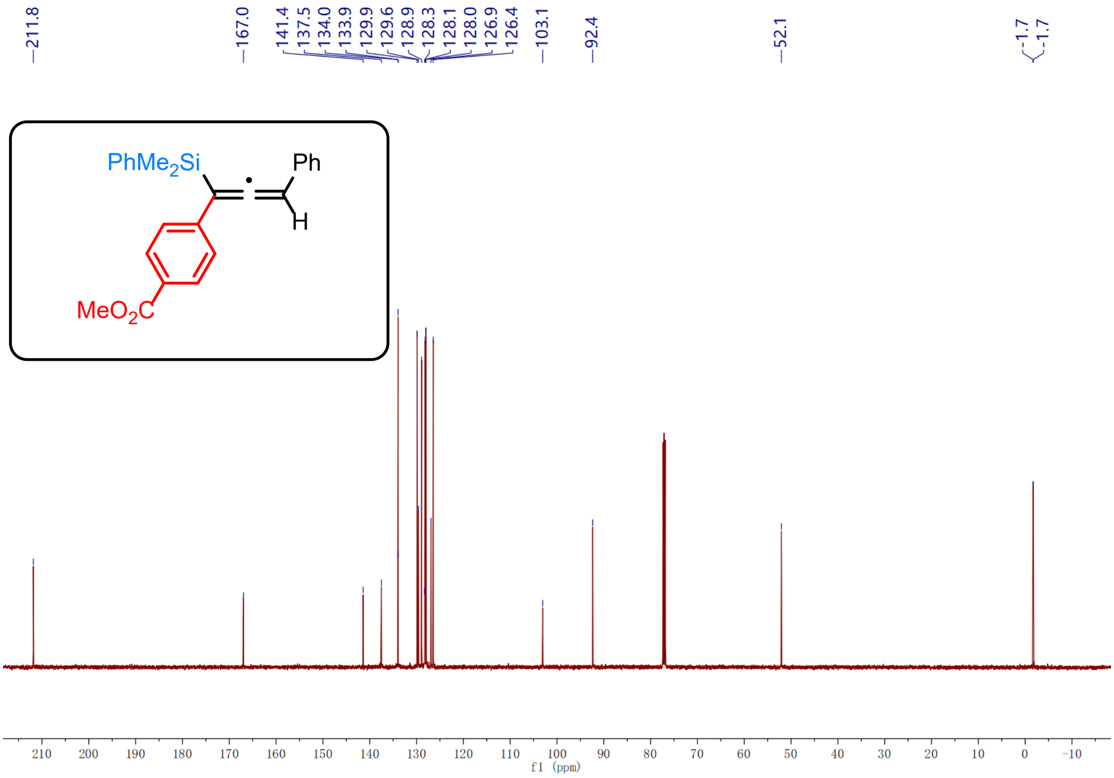

^{1}H NMR spectrum ($CDCl_3$) of **3q**

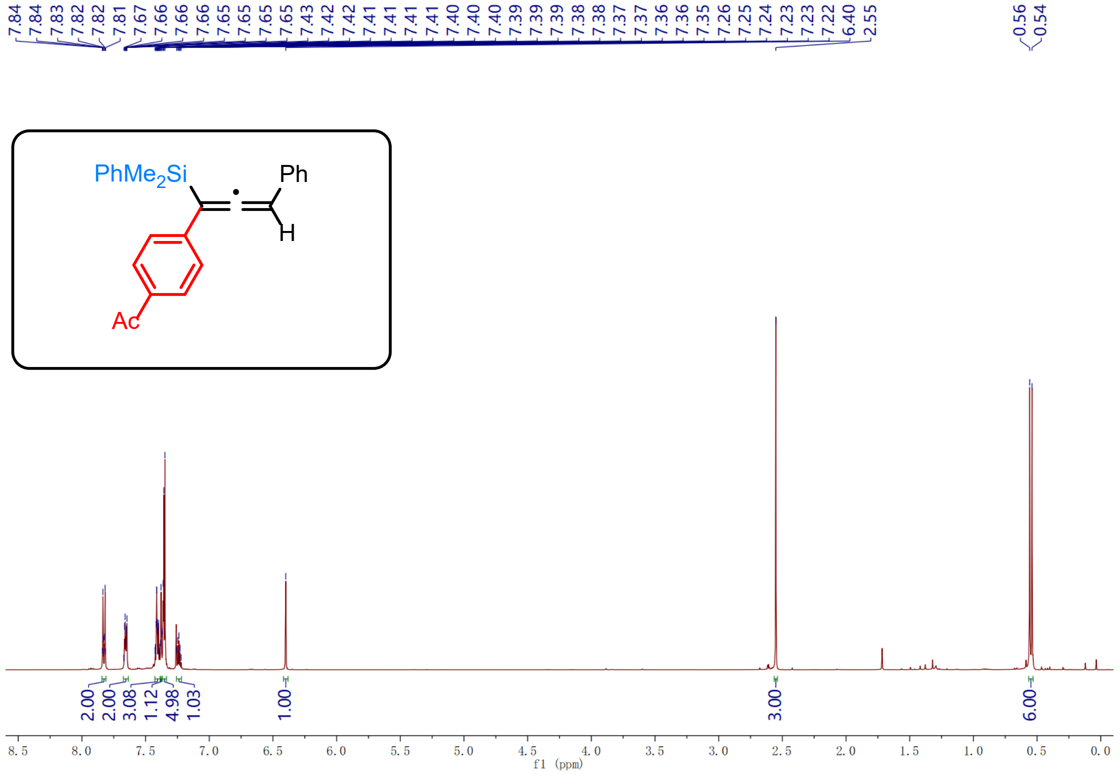

^{13}C NMR spectrum ($CDCl_3$) of **3q**

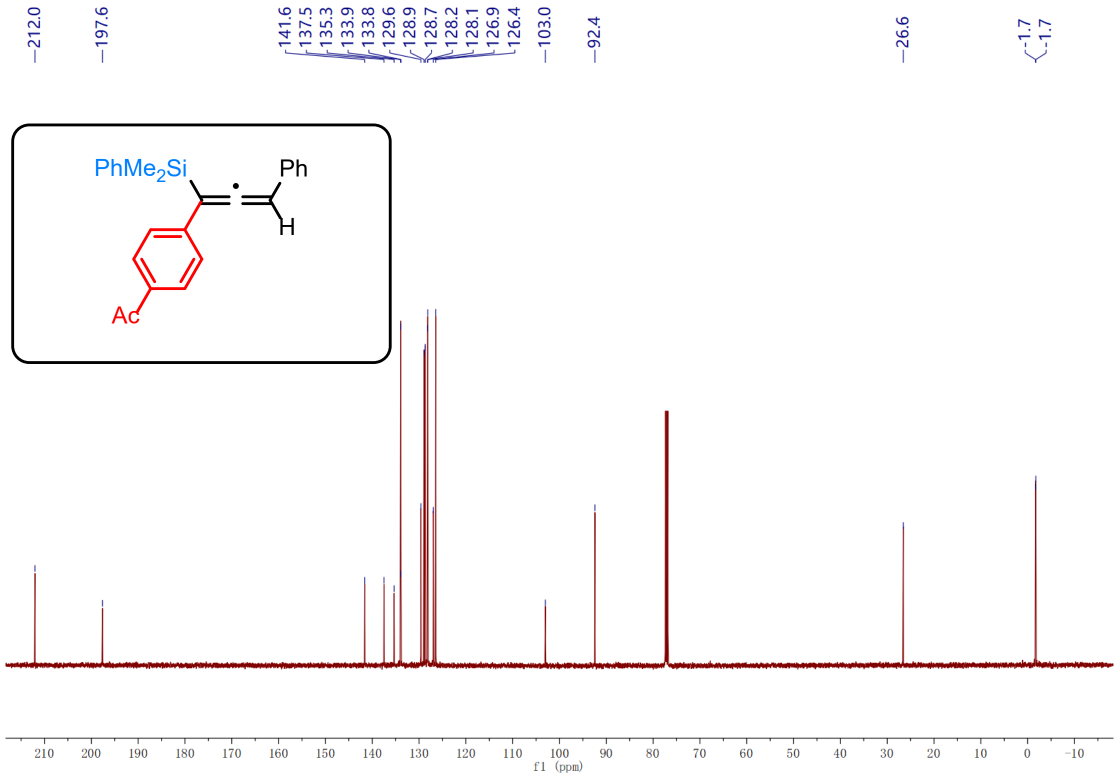

^{1}H NMR spectrum ($CDCl_3$) of **3r**

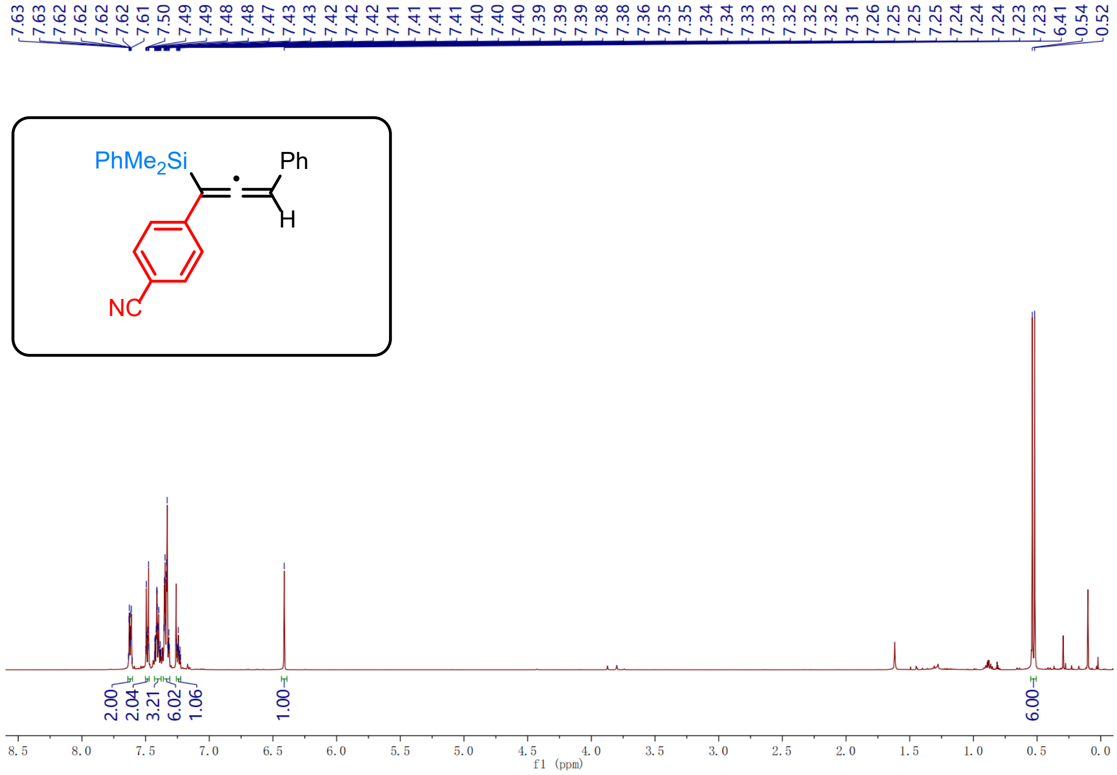

^{13}C NMR spectrum ($CDCl_3$) of **3r**

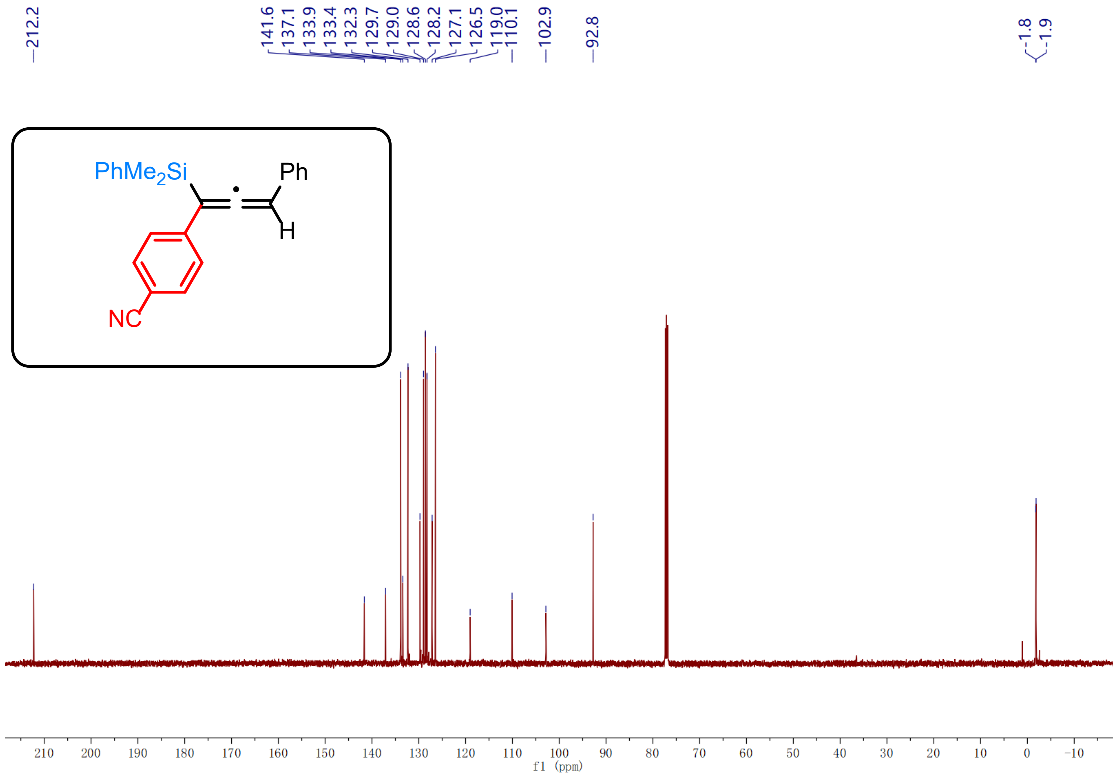

^{1}H NMR spectrum ($CDCl_3$) of **3s**

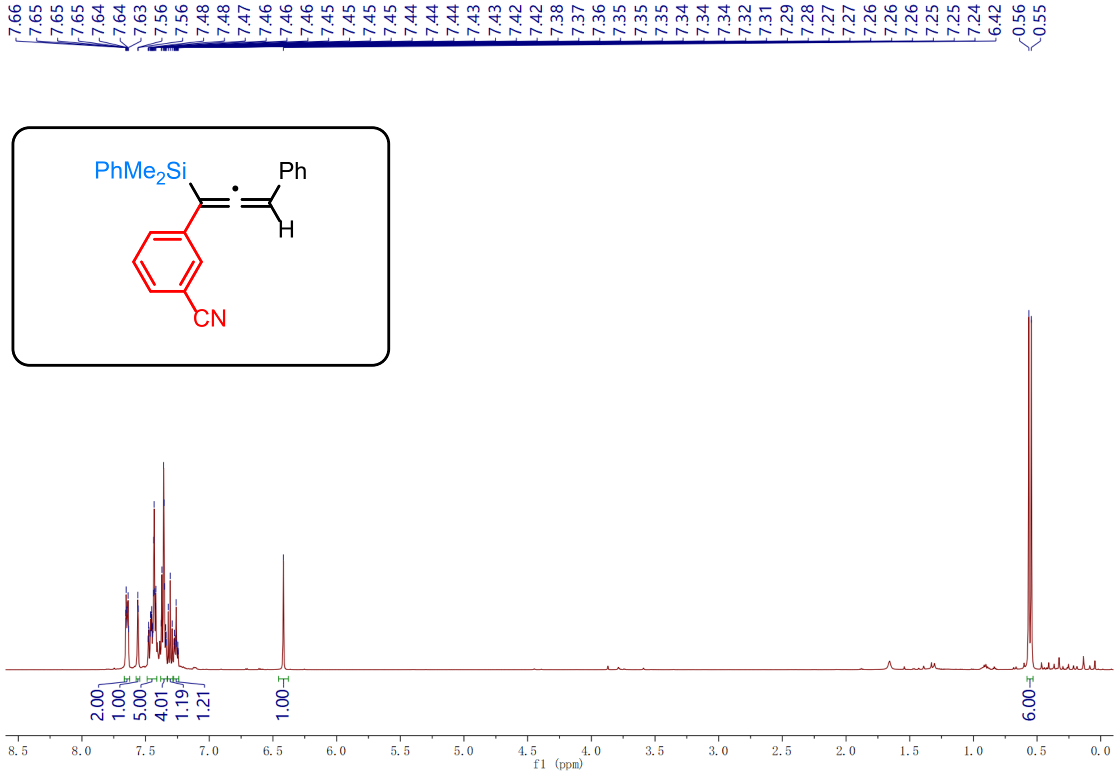

^{13}C NMR spectrum ($CDCl_3$) of **3s**

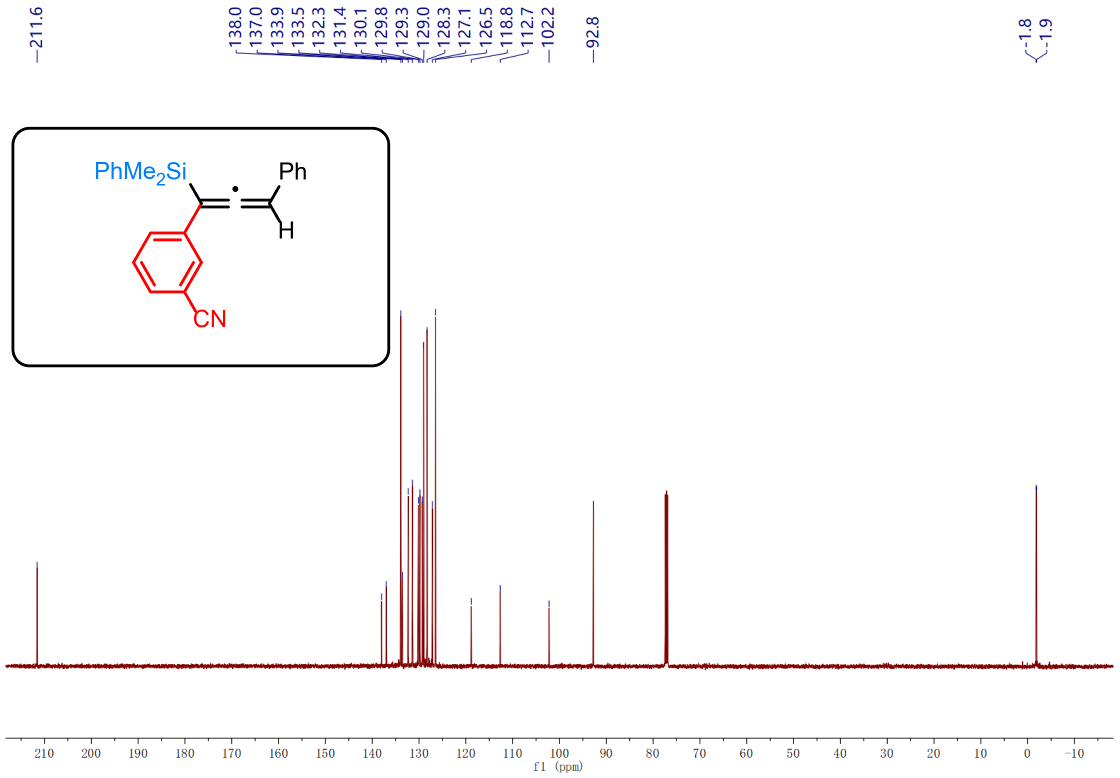

^{1}H NMR spectrum ($CDCl_3$) of **3t**

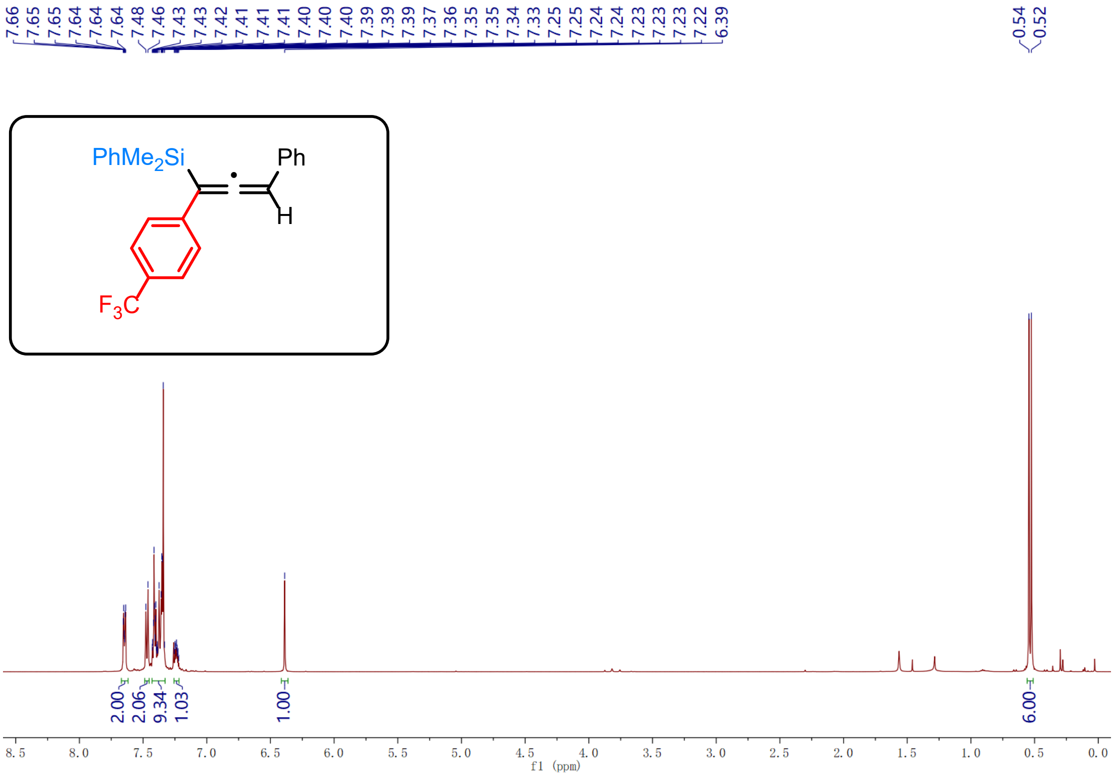

^{13}C NMR spectrum ($CDCl_3$) of **3t**

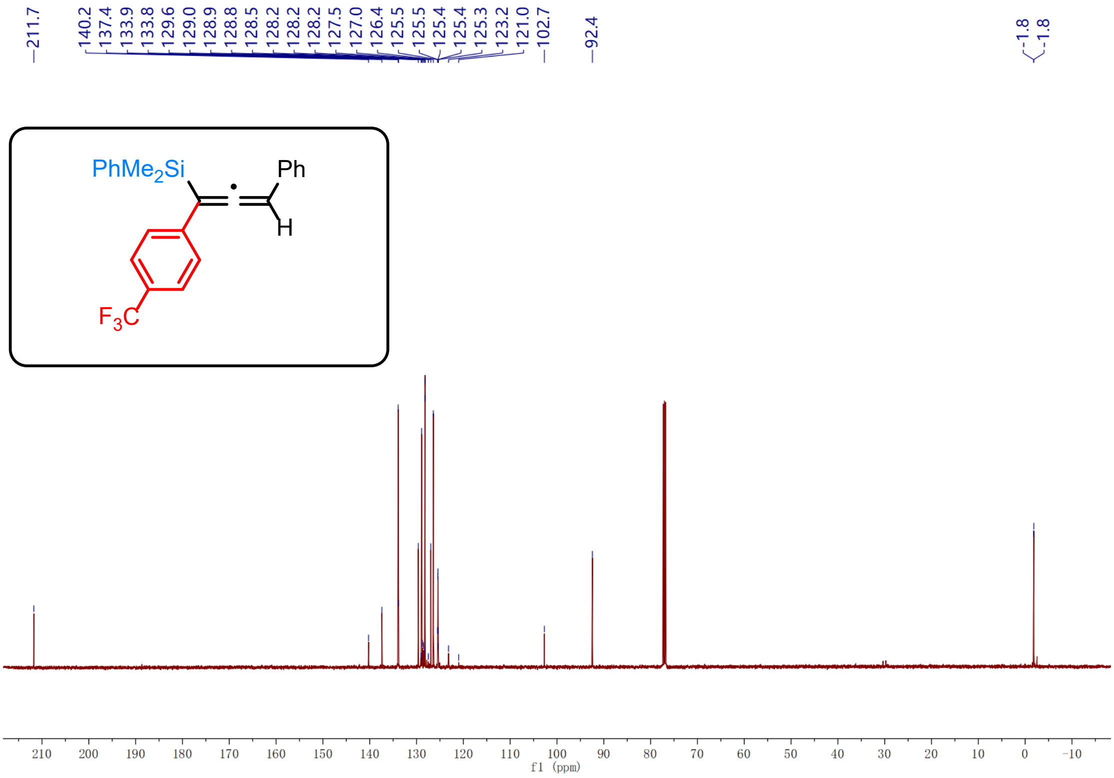

^{19}F NMR spectrum ($CDCl_3$) of **3t**

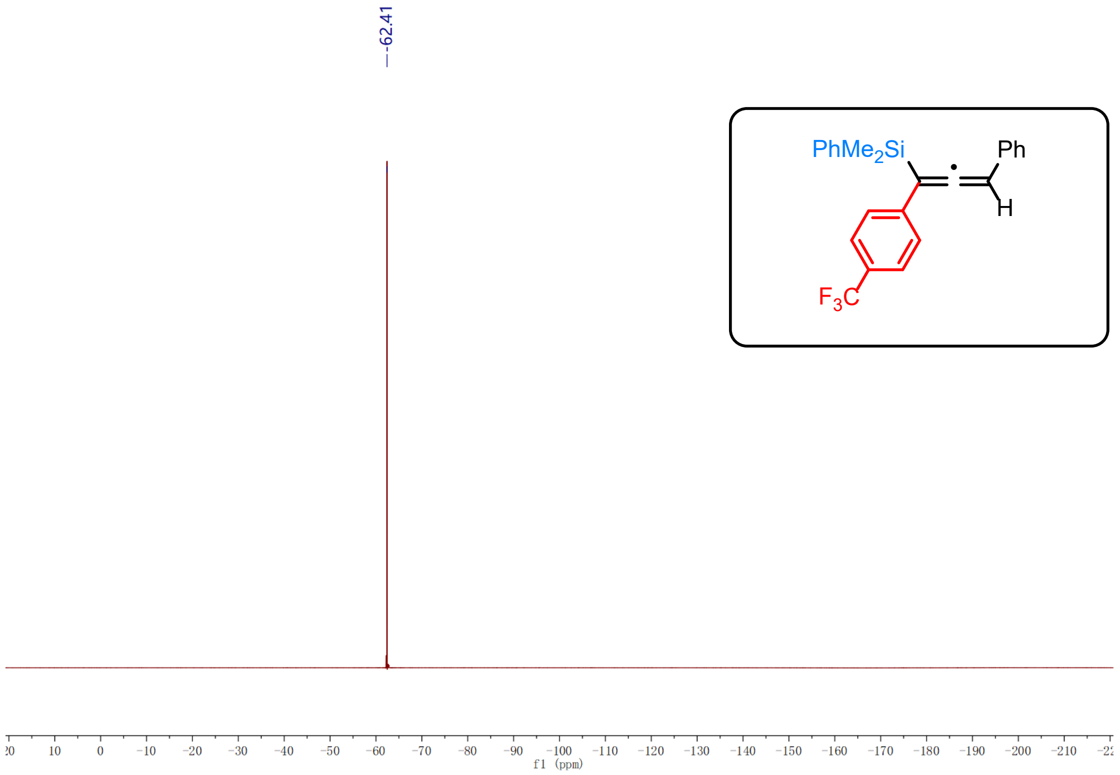

^{1}H NMR spectrum ($CDCl_3$) of **3u**

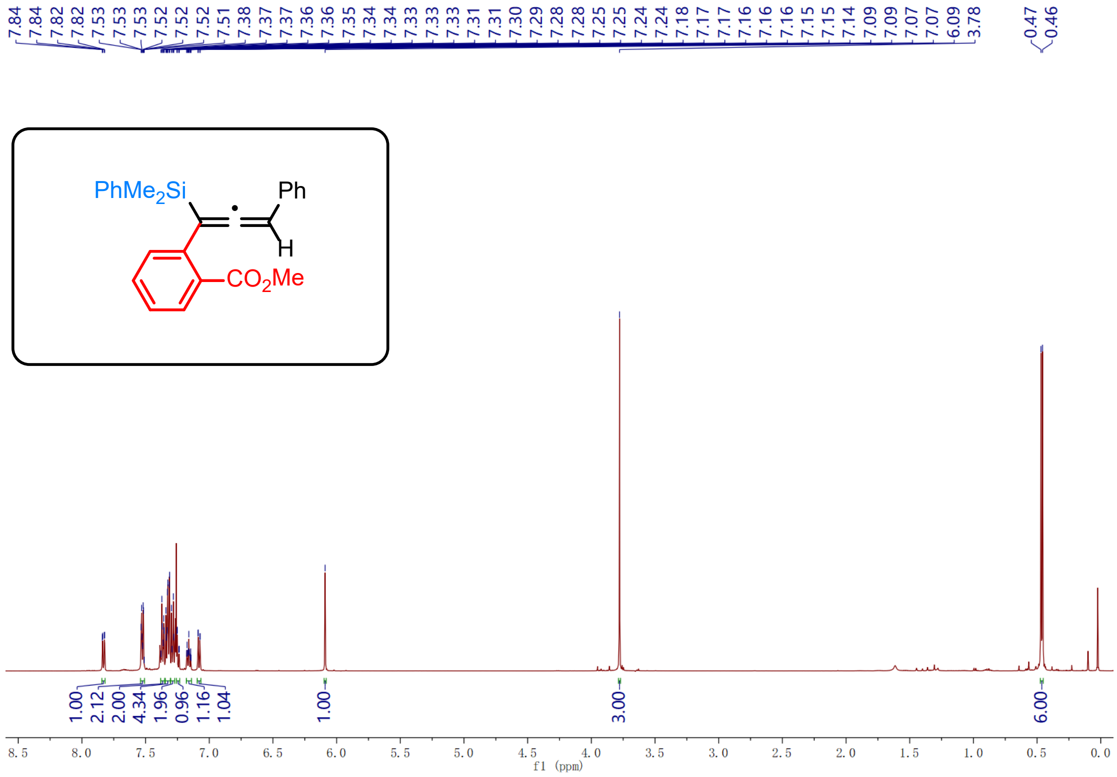

^{13}C NMR spectrum ($CDCl_3$) of **3u**

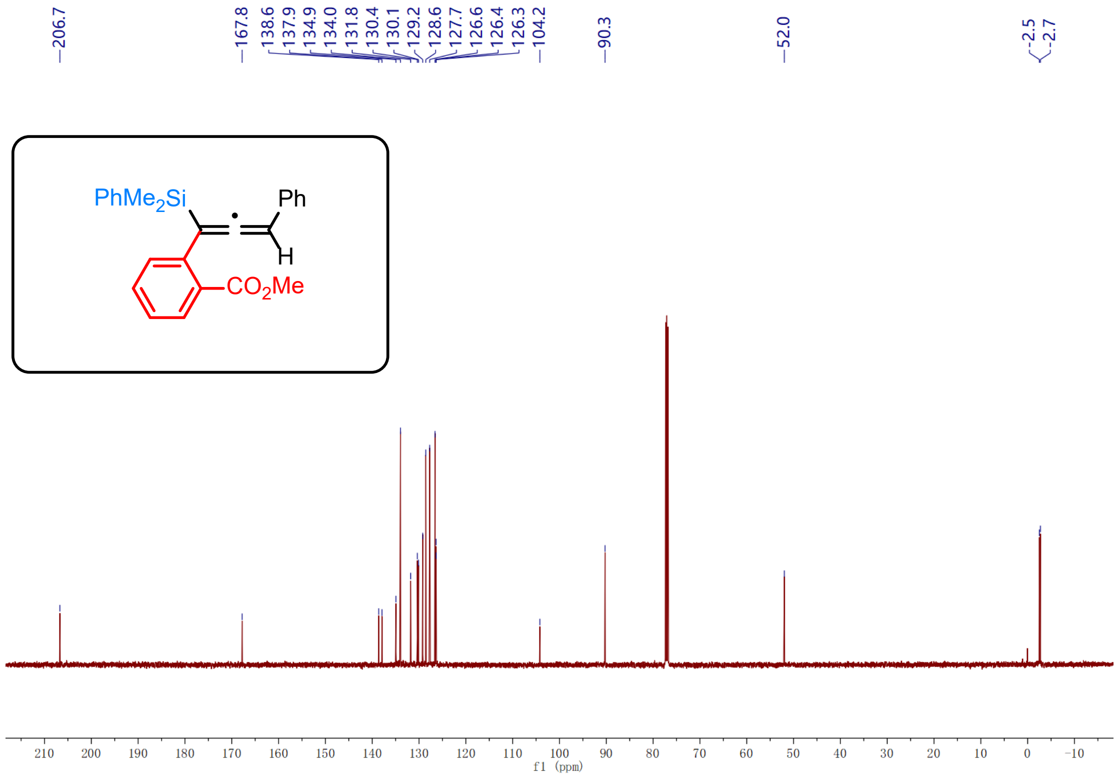

^{1}H NMR spectrum ($CDCl_3$) of **3v**

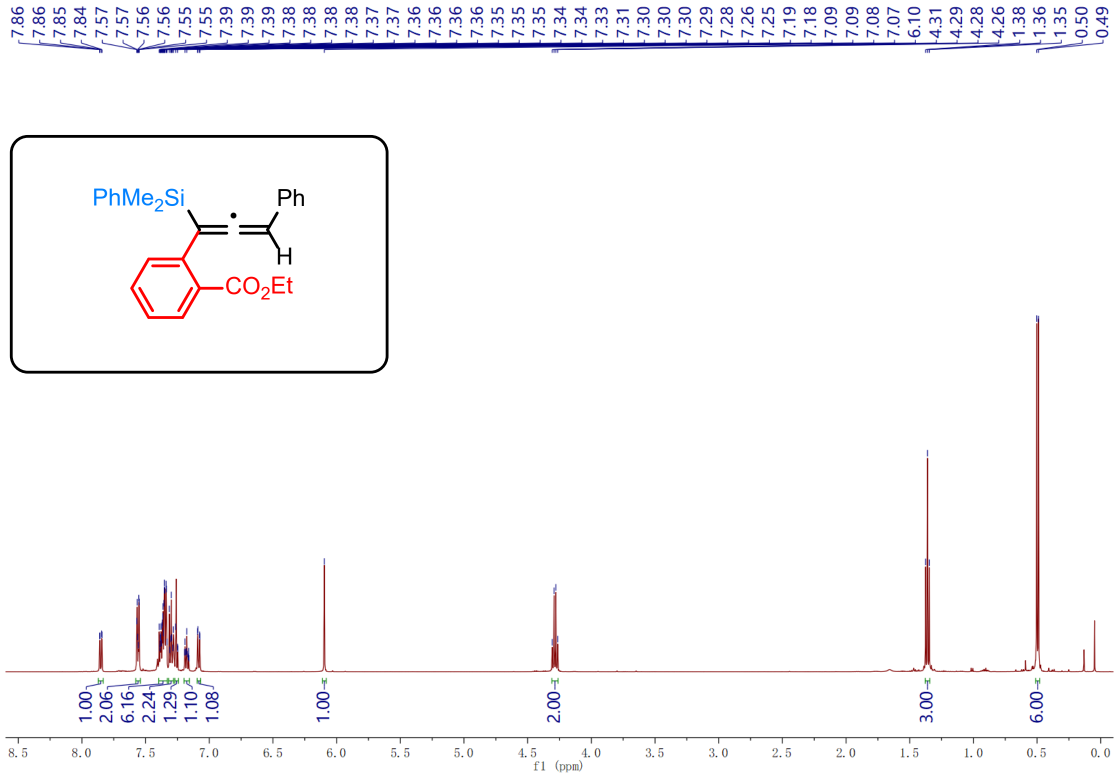

^{13}C NMR spectrum ($CDCl_3$) of **3v**

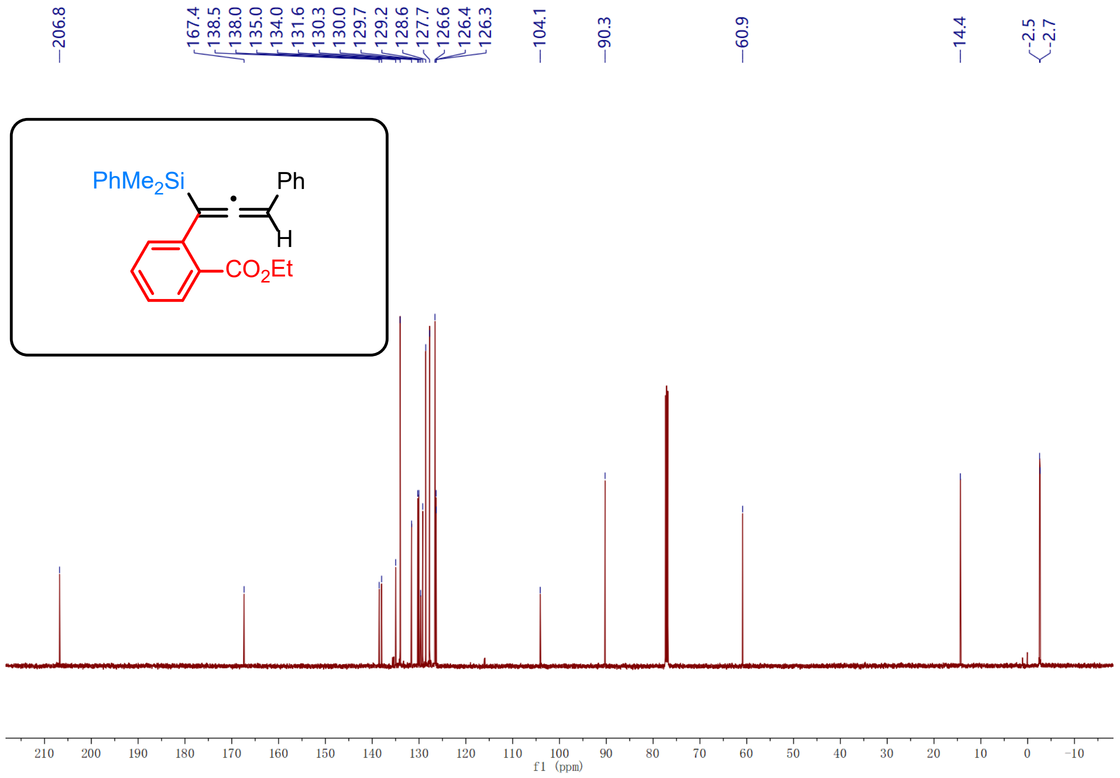

^{1}H NMR spectrum ($CDCl_3$) of **3w**

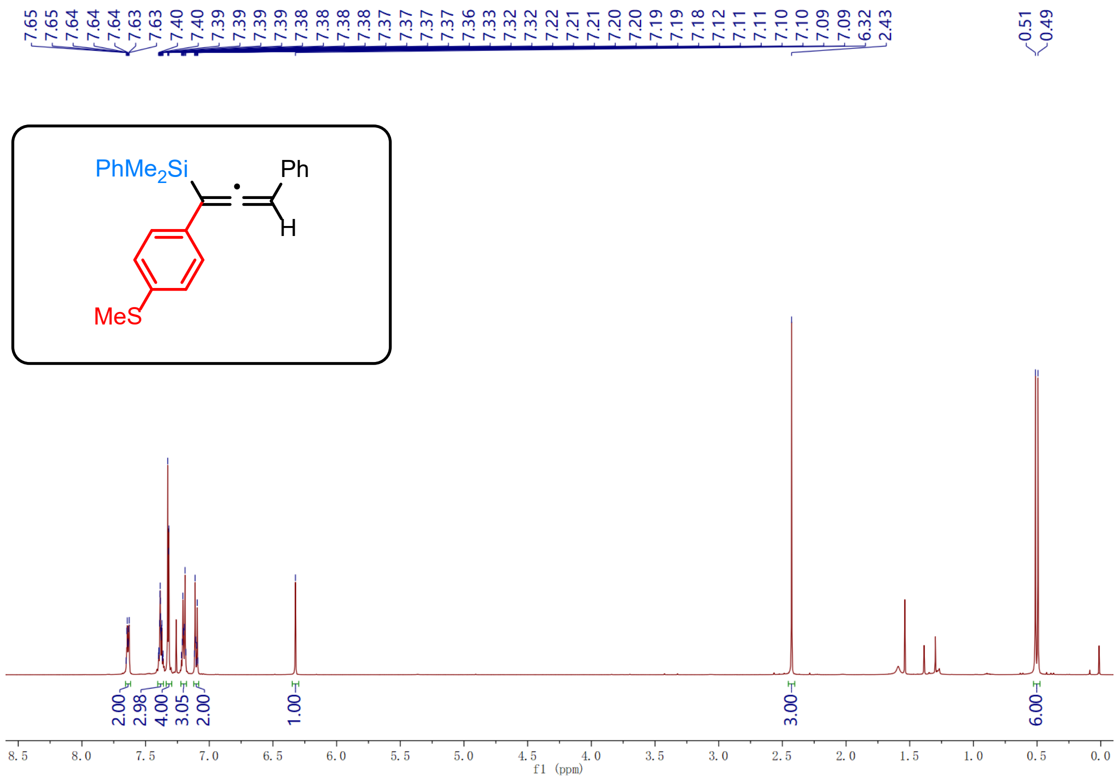

^{13}C NMR spectrum ($CDCl_3$) of **3w**

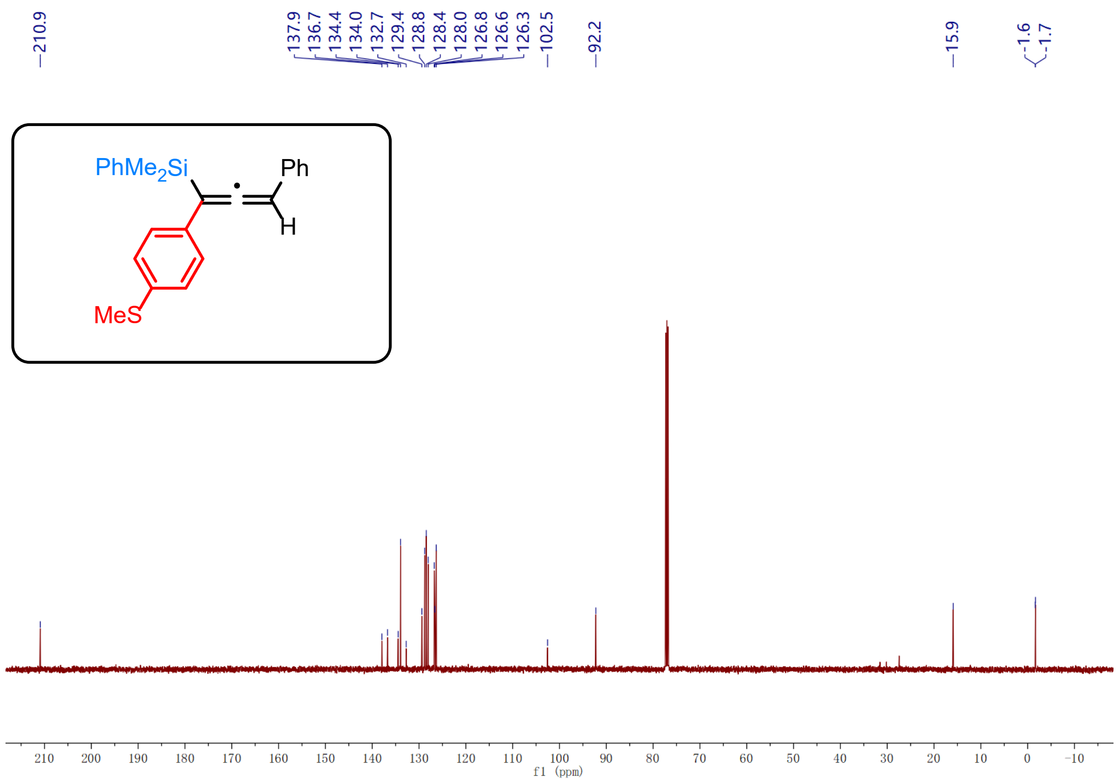

^{1}H NMR spectrum ($CDCl_3$) of **3x**

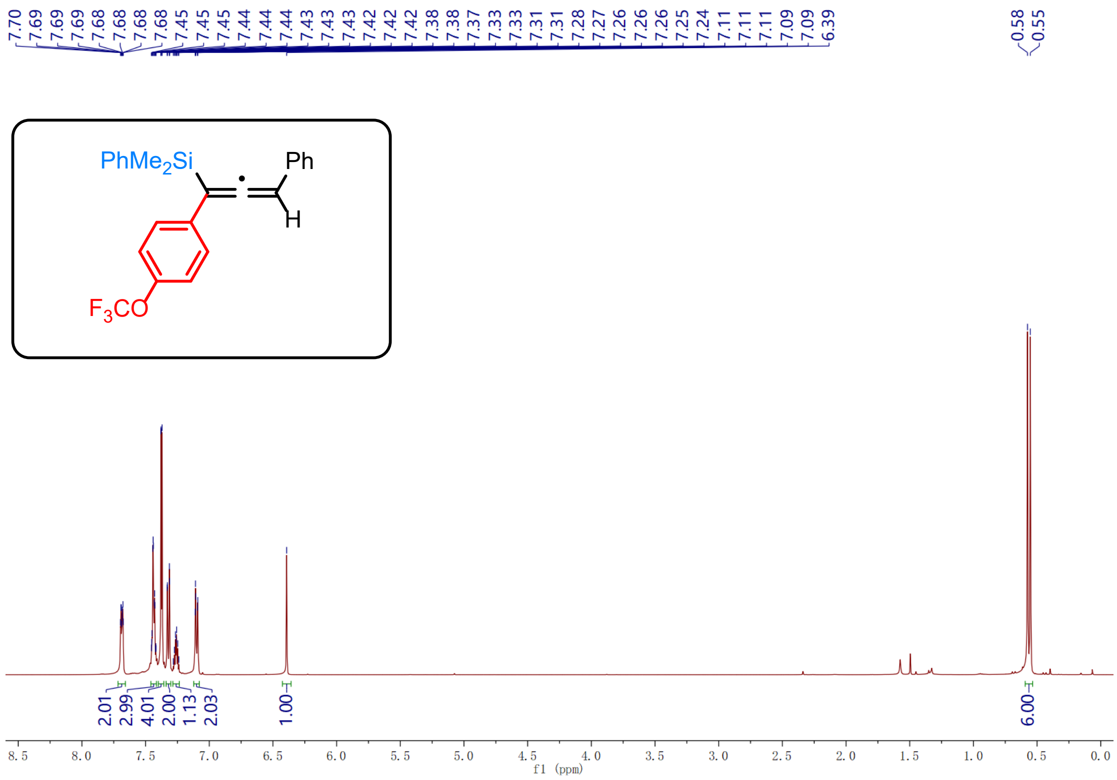

^{13}C NMR spectrum ($CDCl_3$) of **3x**

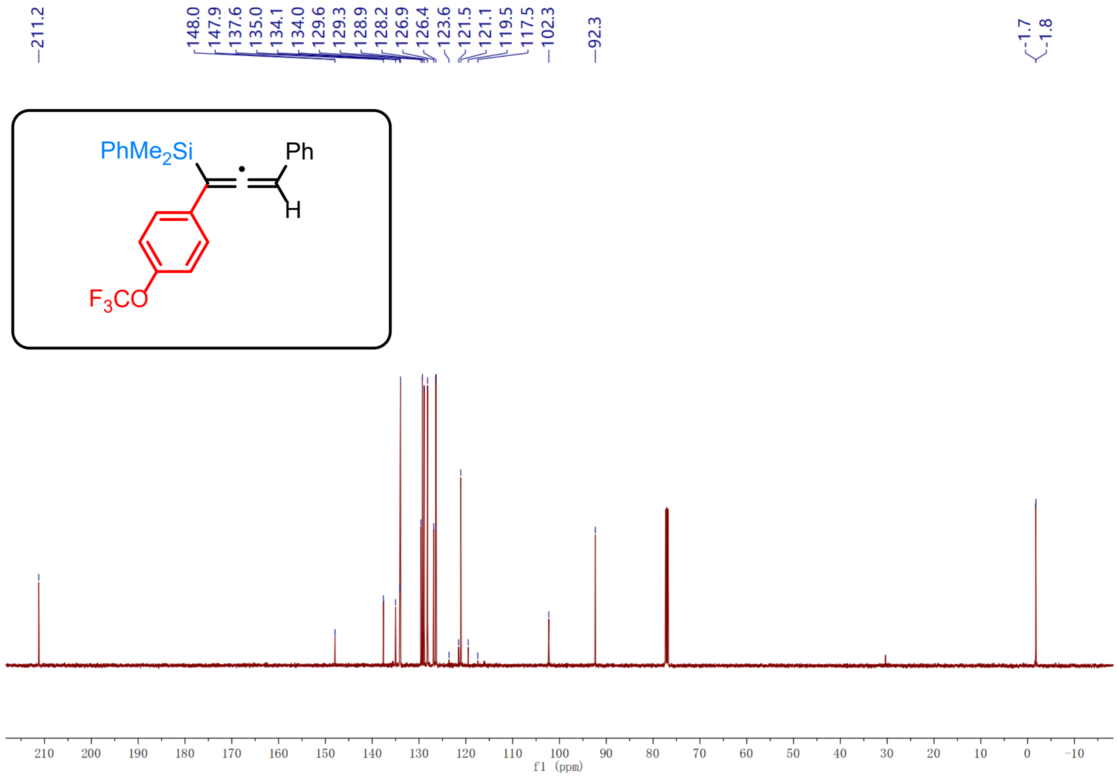

^{19}F NMR spectrum ($CDCl_3$) of **3x**

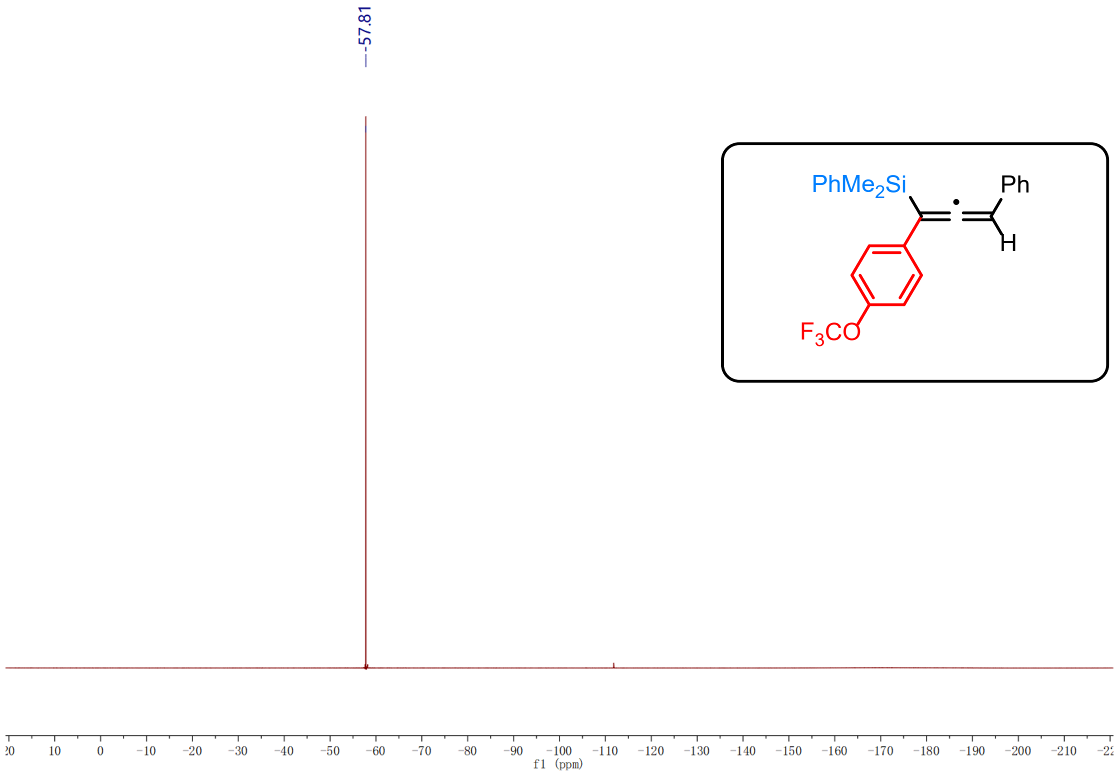

^{1}H NMR spectrum ($CDCl_3$) of **3y**

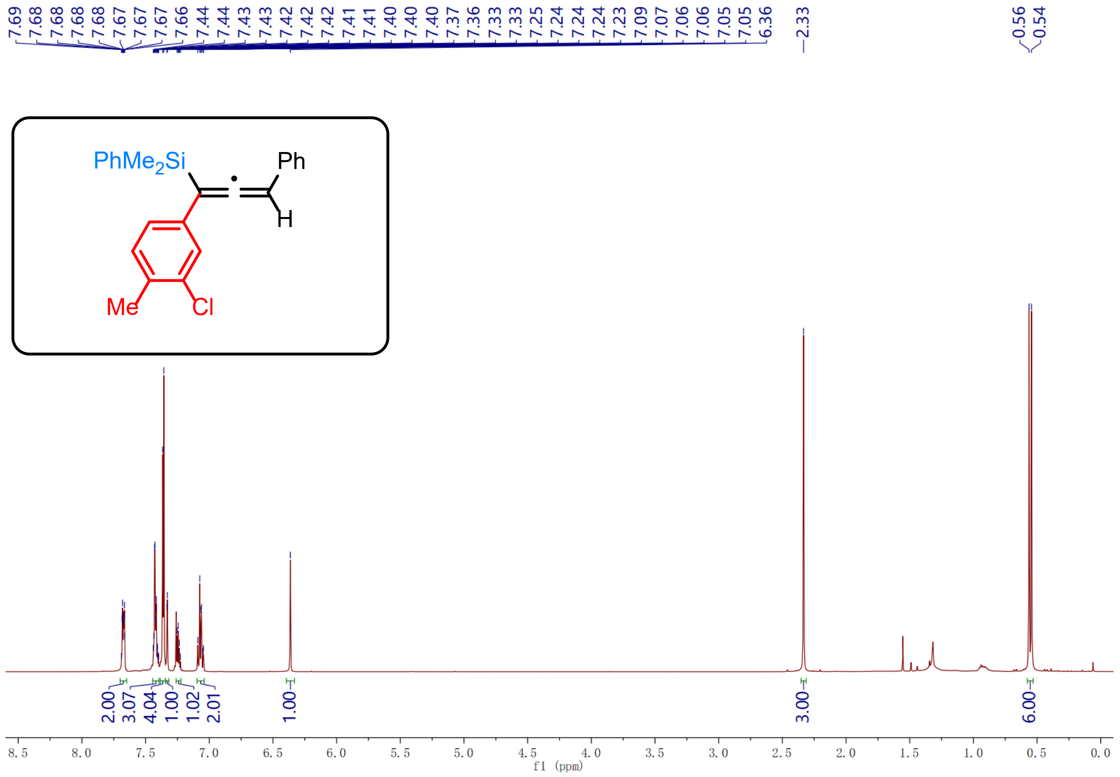

^{13}C NMR spectrum ($CDCl_3$) of **3y**

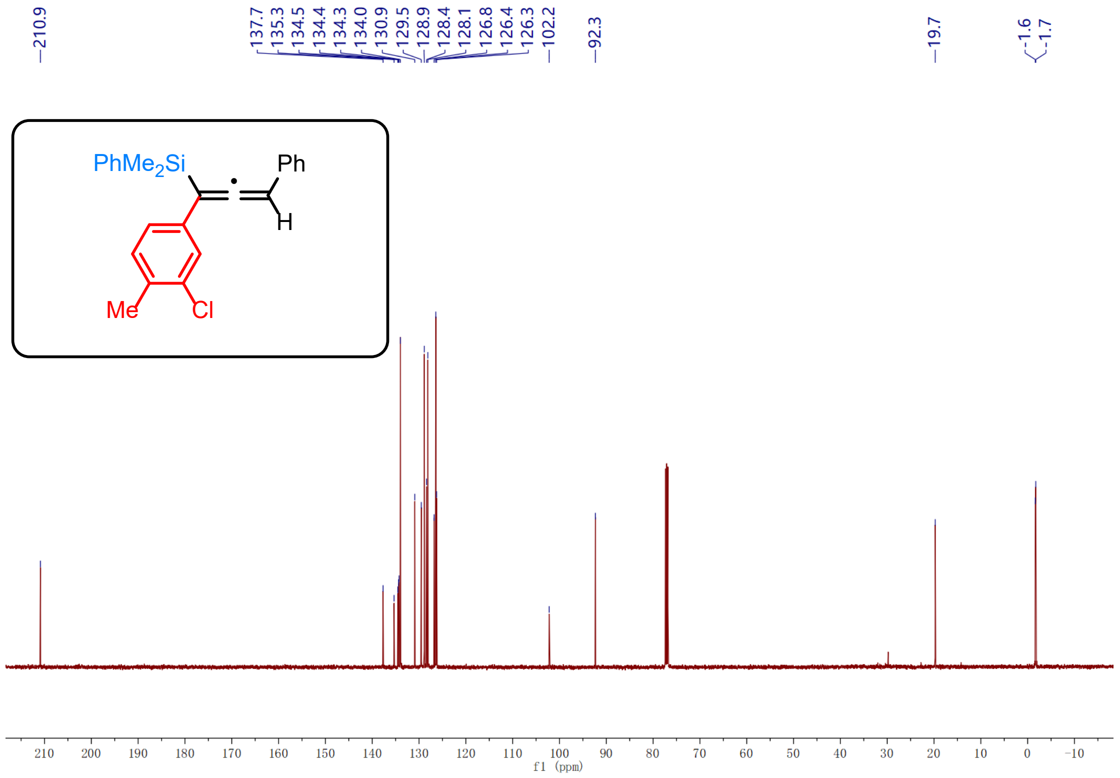

^{1}H NMR spectrum ($CDCl_3$) of **3z**

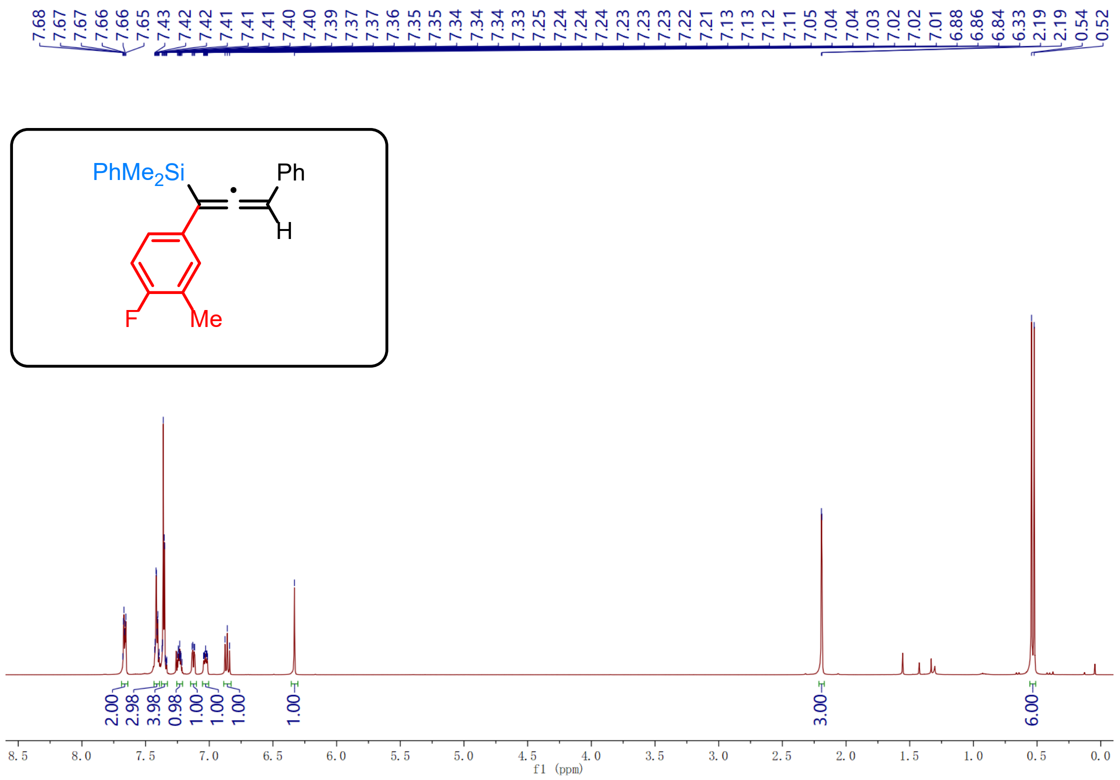

^{13}C NMR spectrum ($CDCl_3$) of **3z**

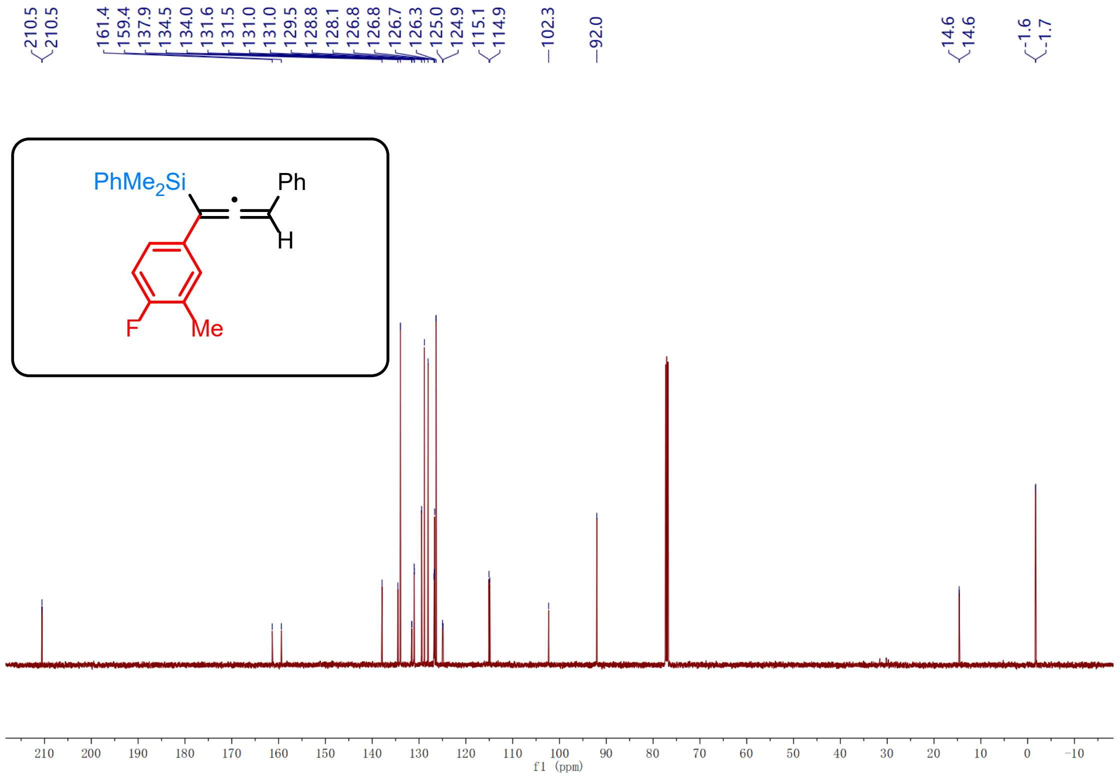

^{19}F NMR spectrum ($CDCl_3$) of **3z**

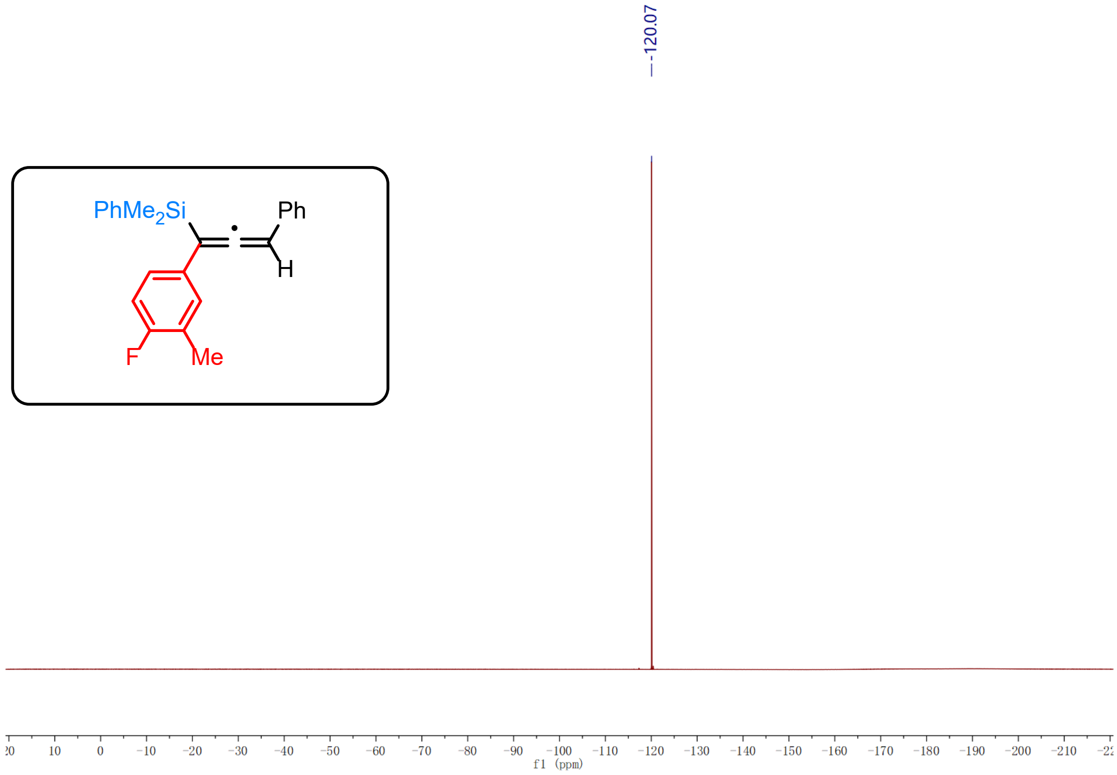

^{1}H NMR spectrum ($CDCl_3$) of **3aa**

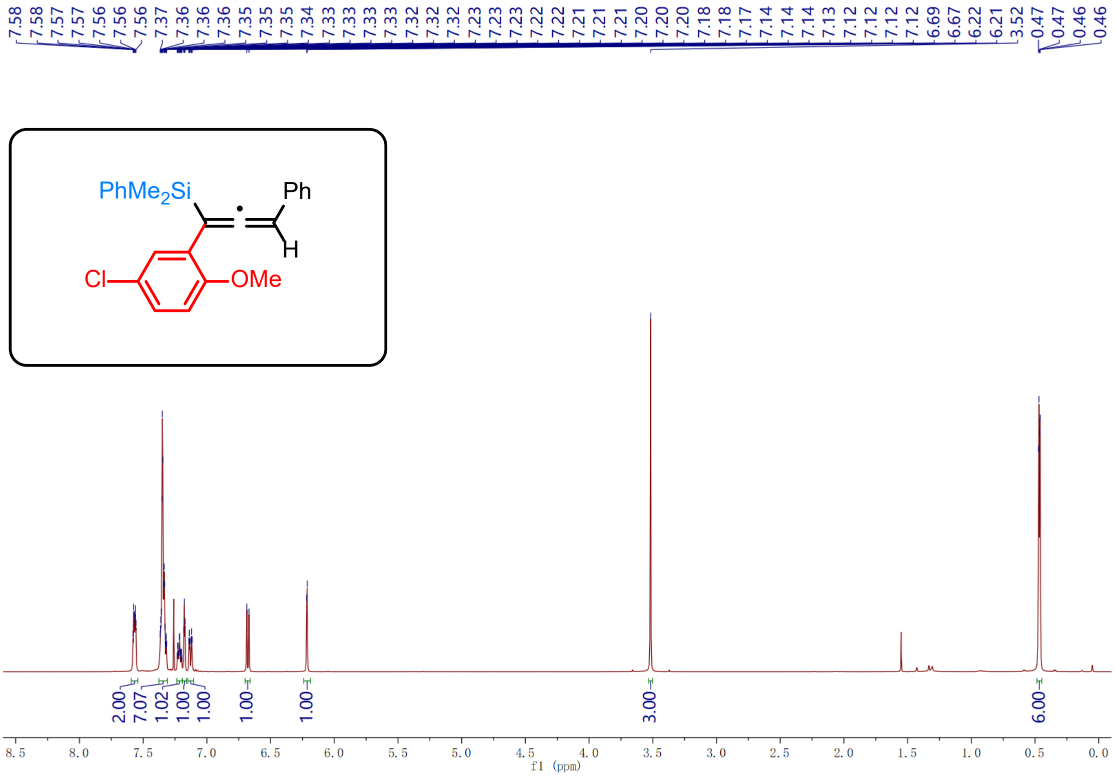

^{13}C NMR spectrum ($CDCl_3$) of **3aa**

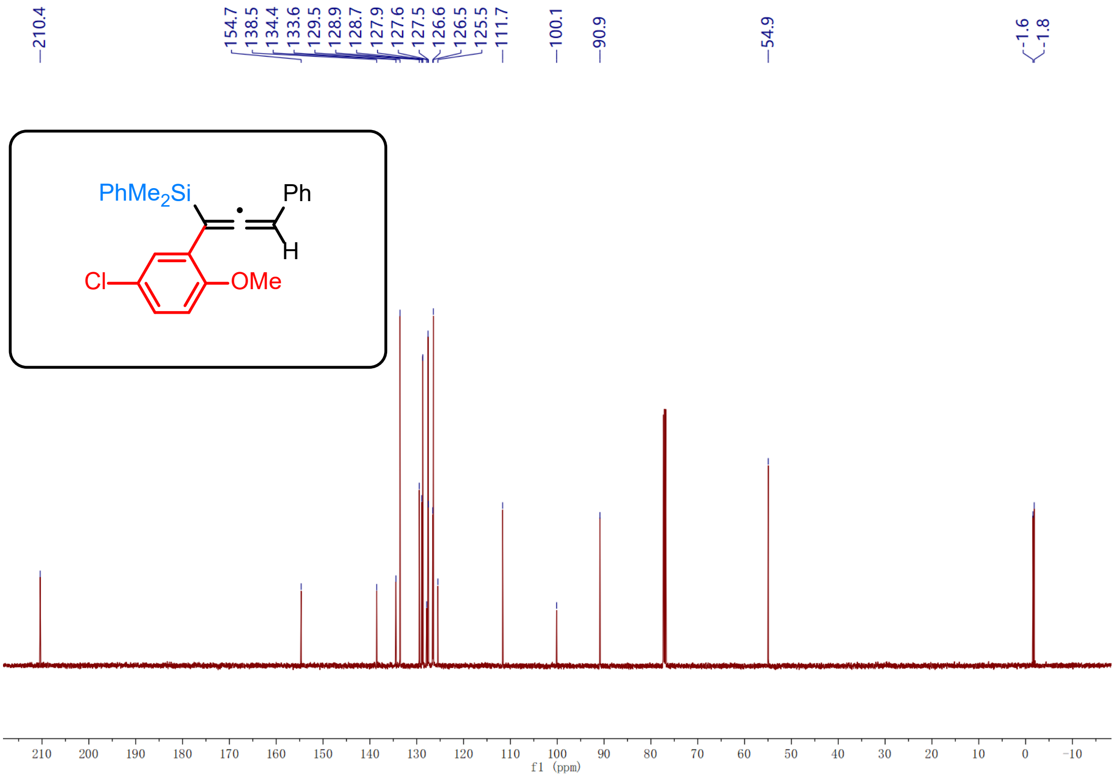

^{1}H NMR spectrum ($CDCl_3$) of **3ab**

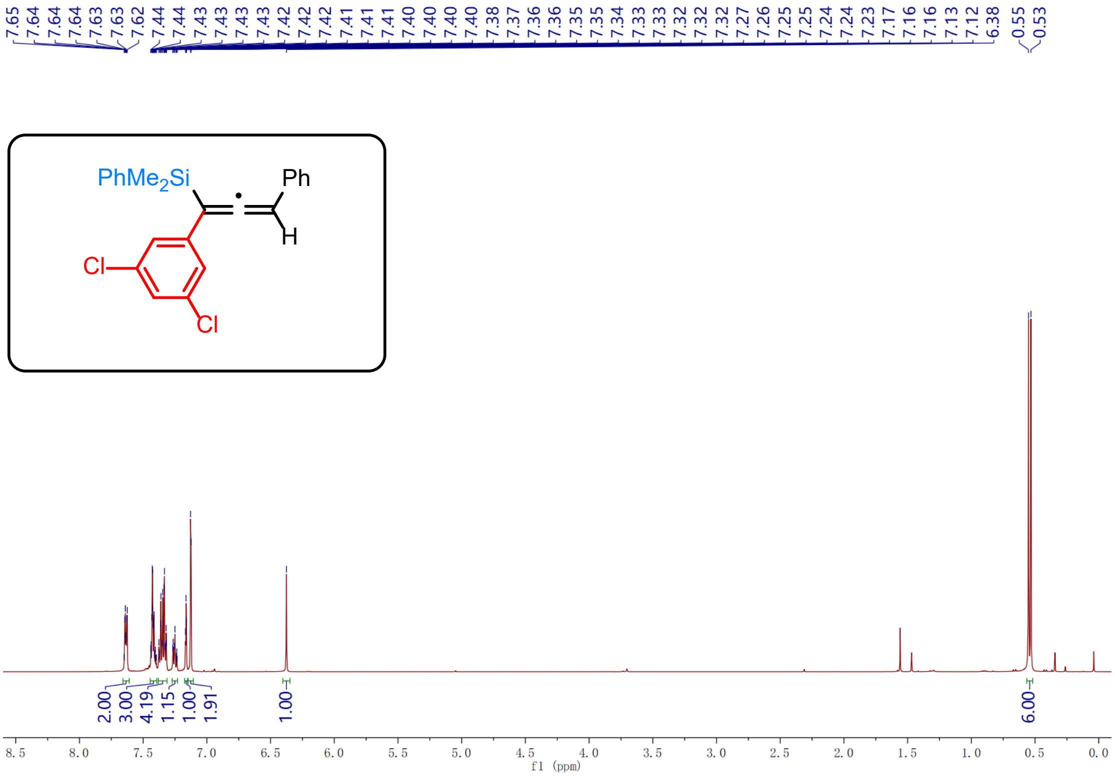

^{13}C NMR spectrum ($CDCl_3$) of **3ab**

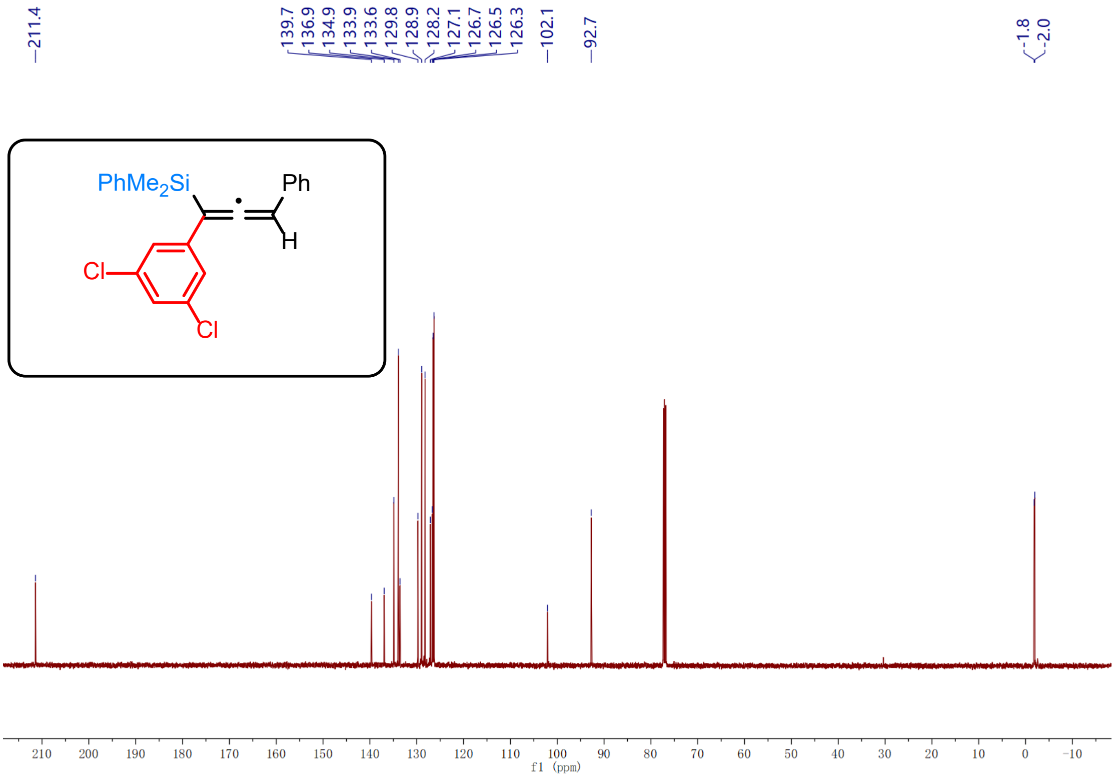

^{1}H NMR spectrum ($CDCl_3$) of **3ac**

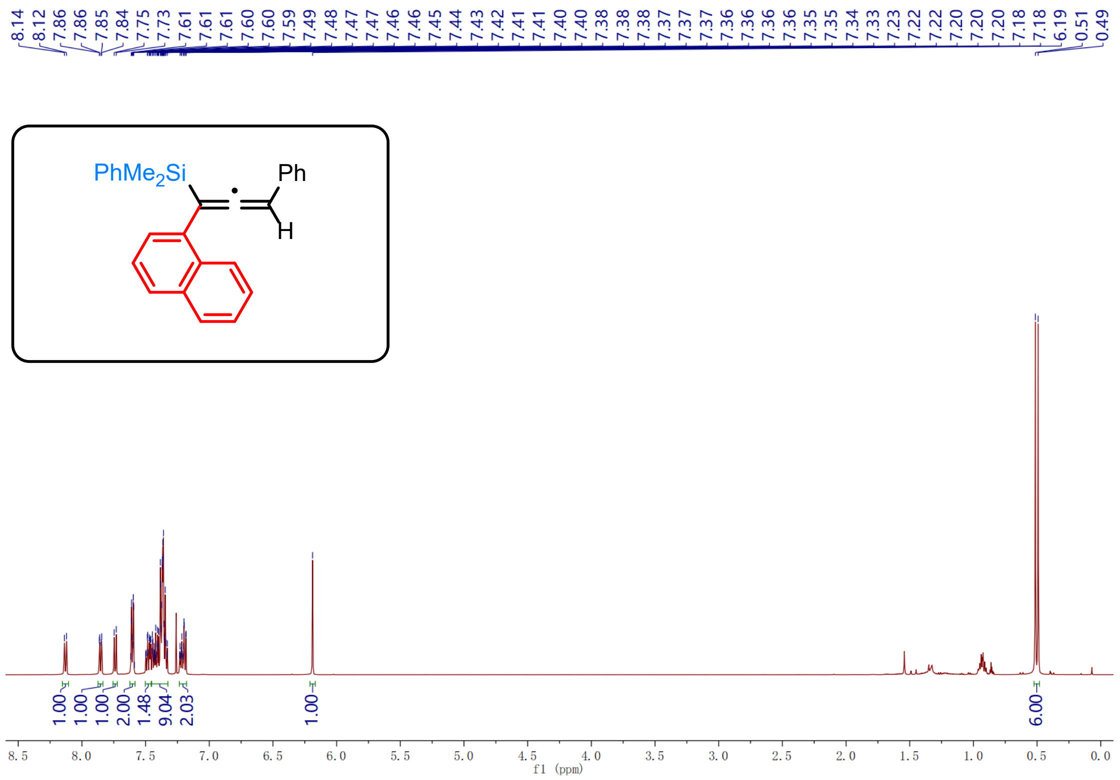

^{13}C NMR spectrum ($CDCl_3$) of **3ac**

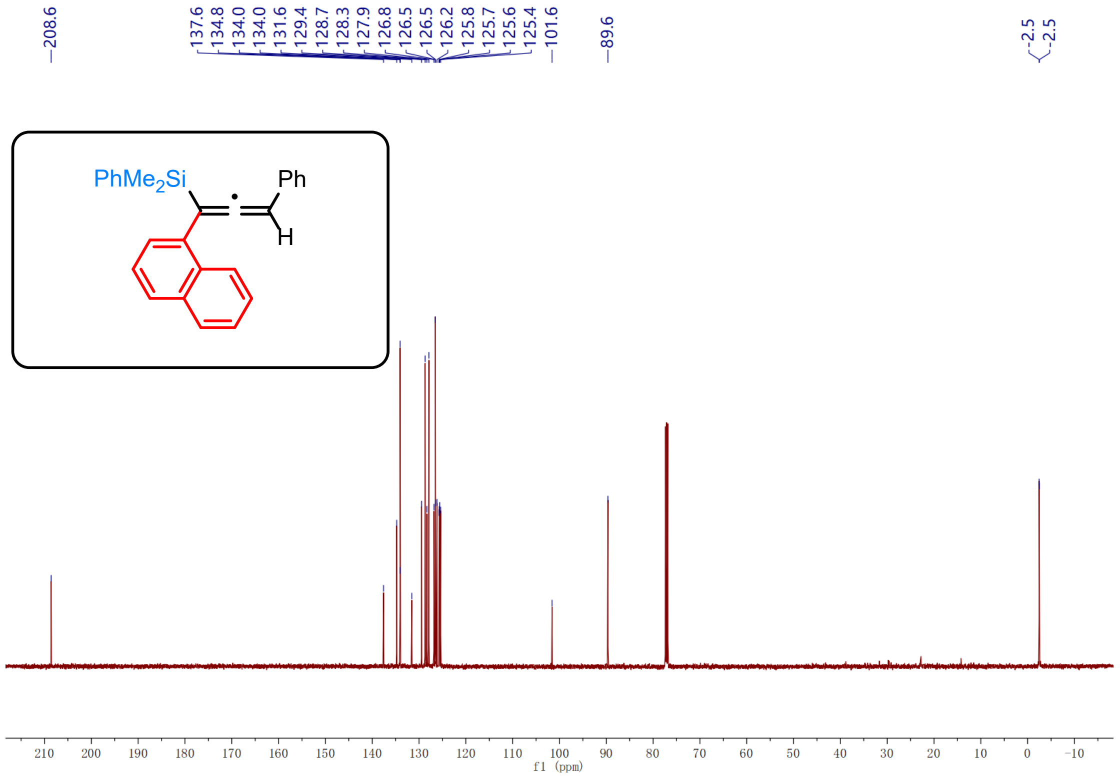

^{1}H NMR spectrum ($CDCl_3$) of **3ad**

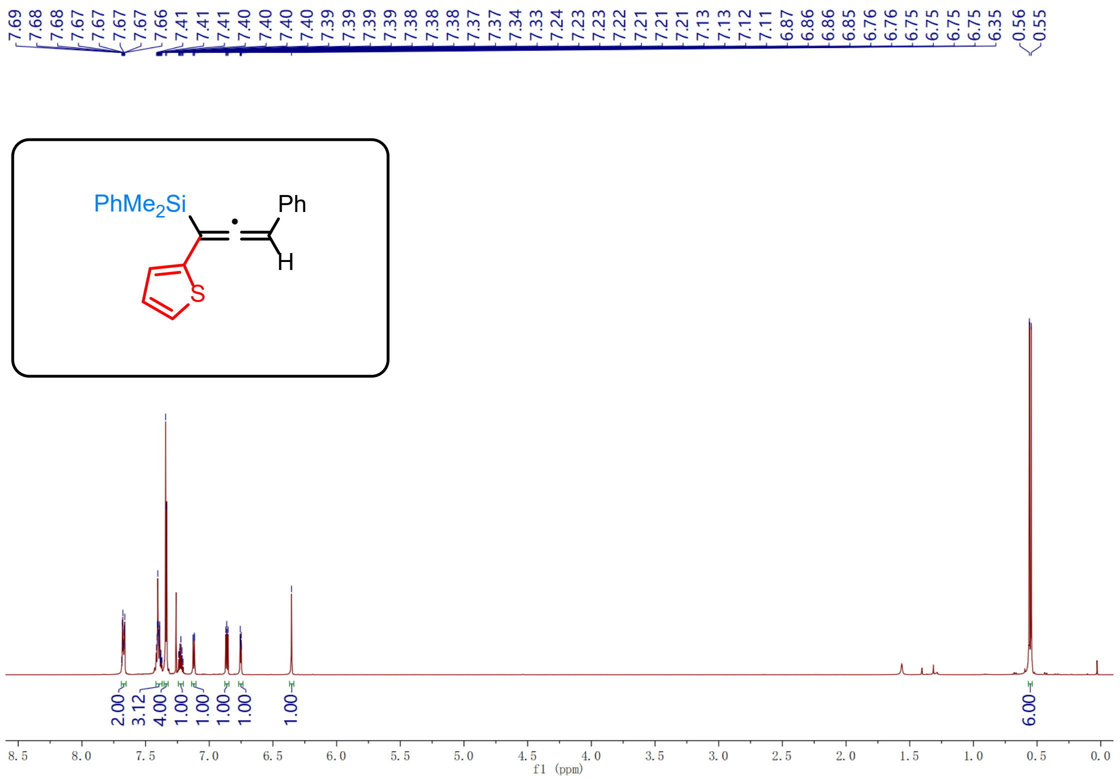

^{13}C NMR spectrum ($CDCl_3$) of **3ad**

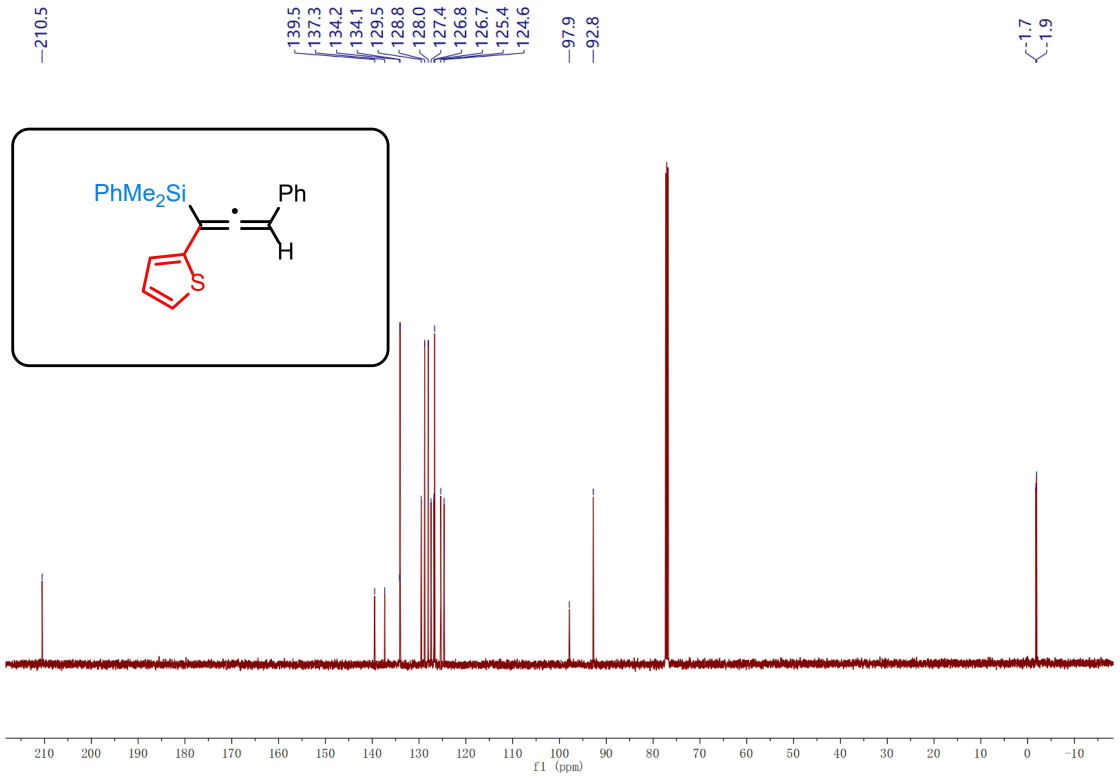

^{1}H NMR spectrum ($CDCl_3$) of **3ae**

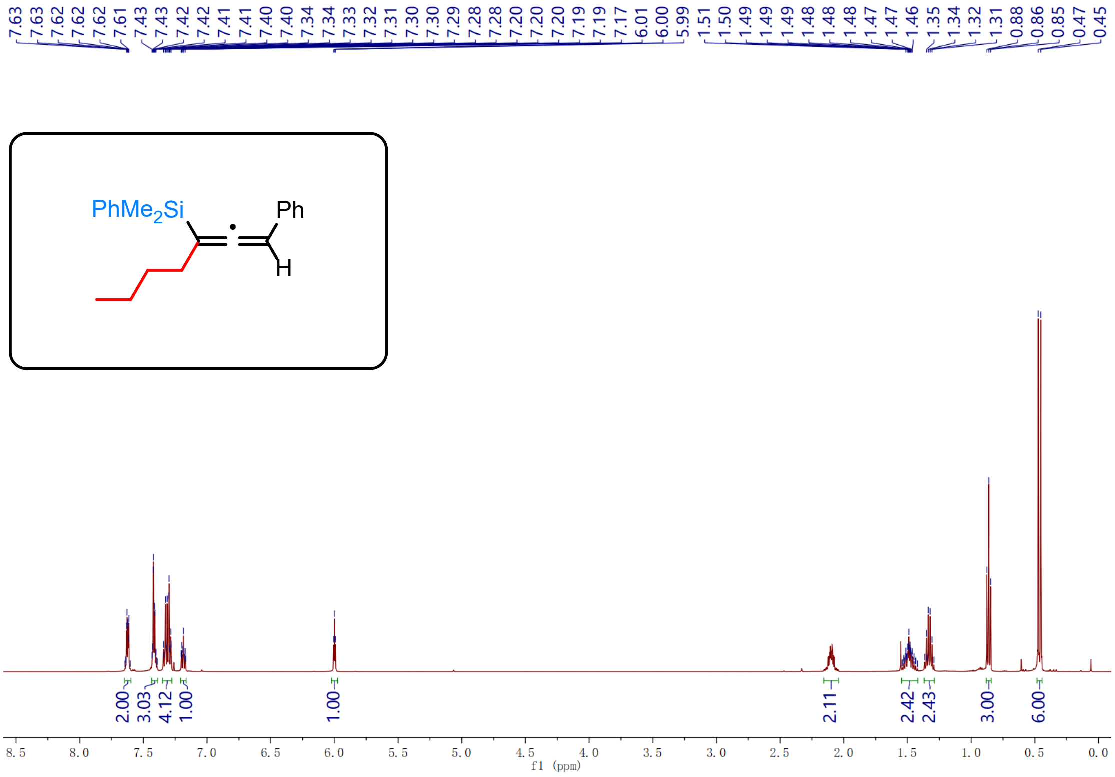

^{13}C NMR spectrum ($CDCl_3$) of **3ae**

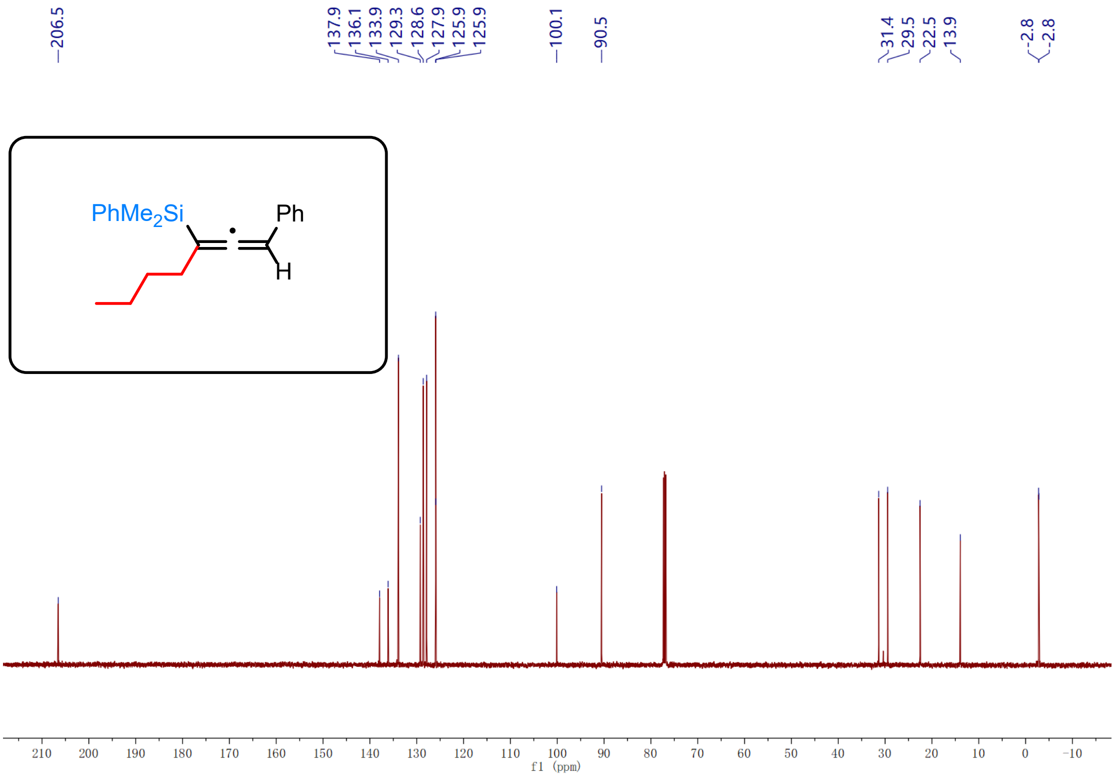

^{1}H NMR spectrum ($CDCl_3$) of **3af**

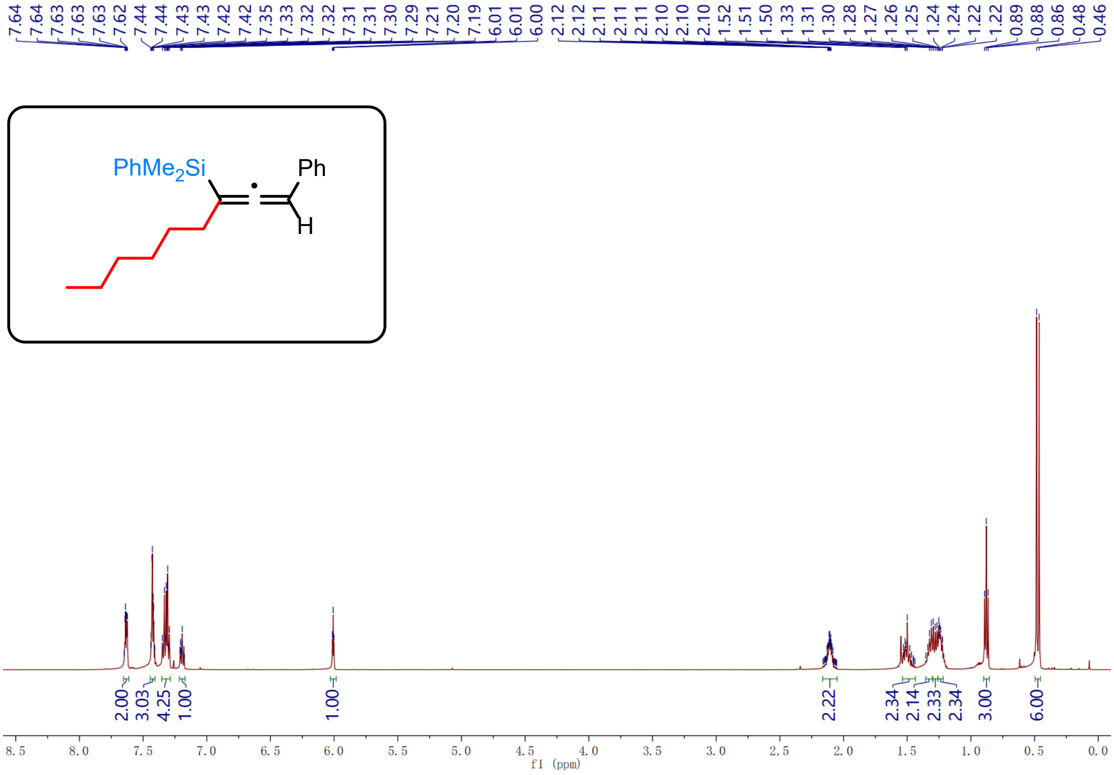

^{13}C NMR spectrum ($CDCl_3$) of **3af**

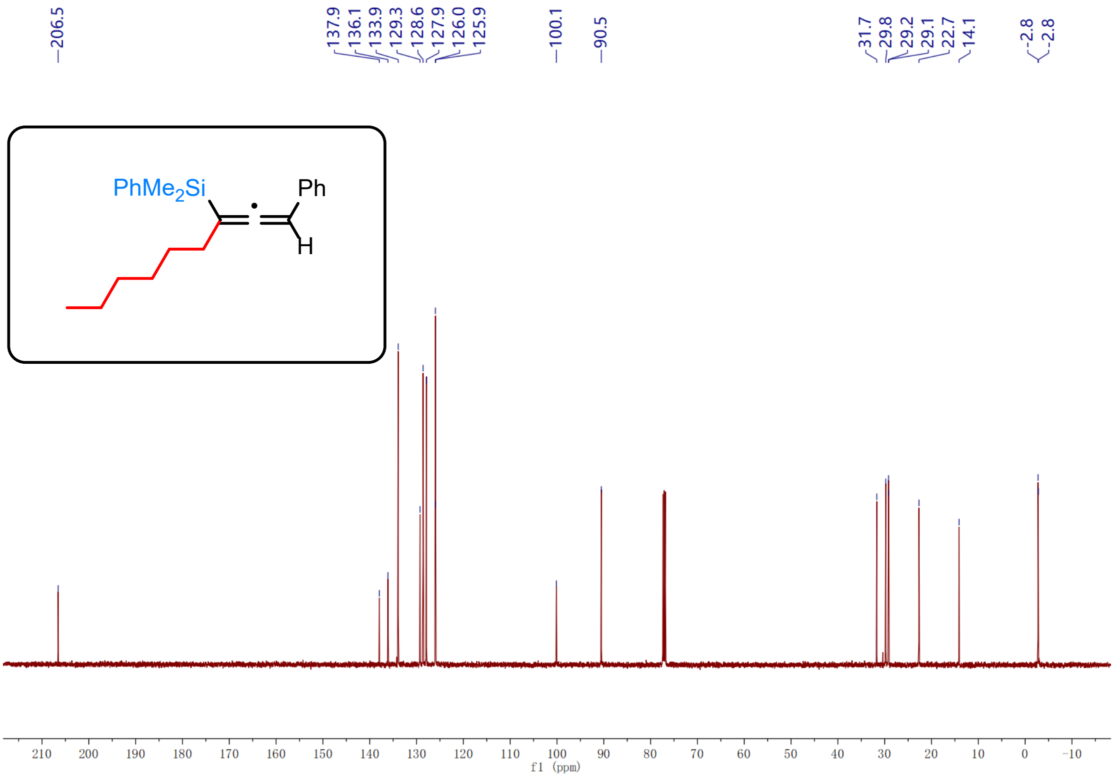

^{1}H NMR spectrum ($CDCl_3$) of **3ag**

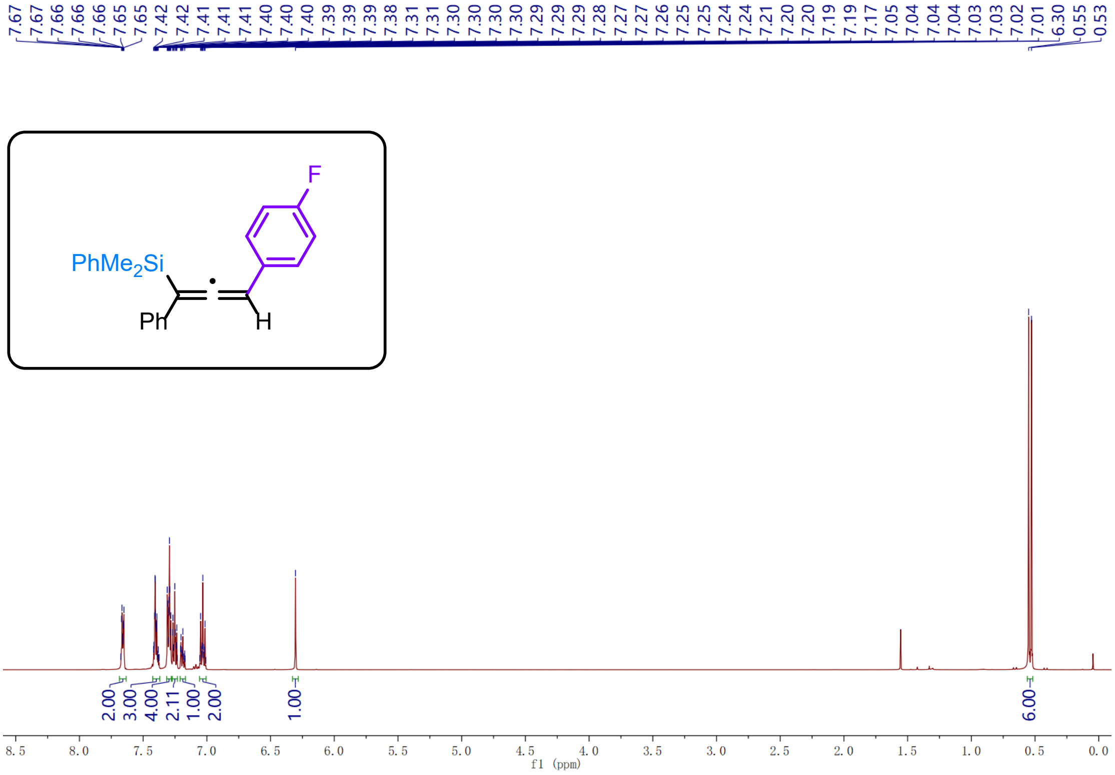

^{13}C NMR spectrum ($CDCl_3$) of **3ag**

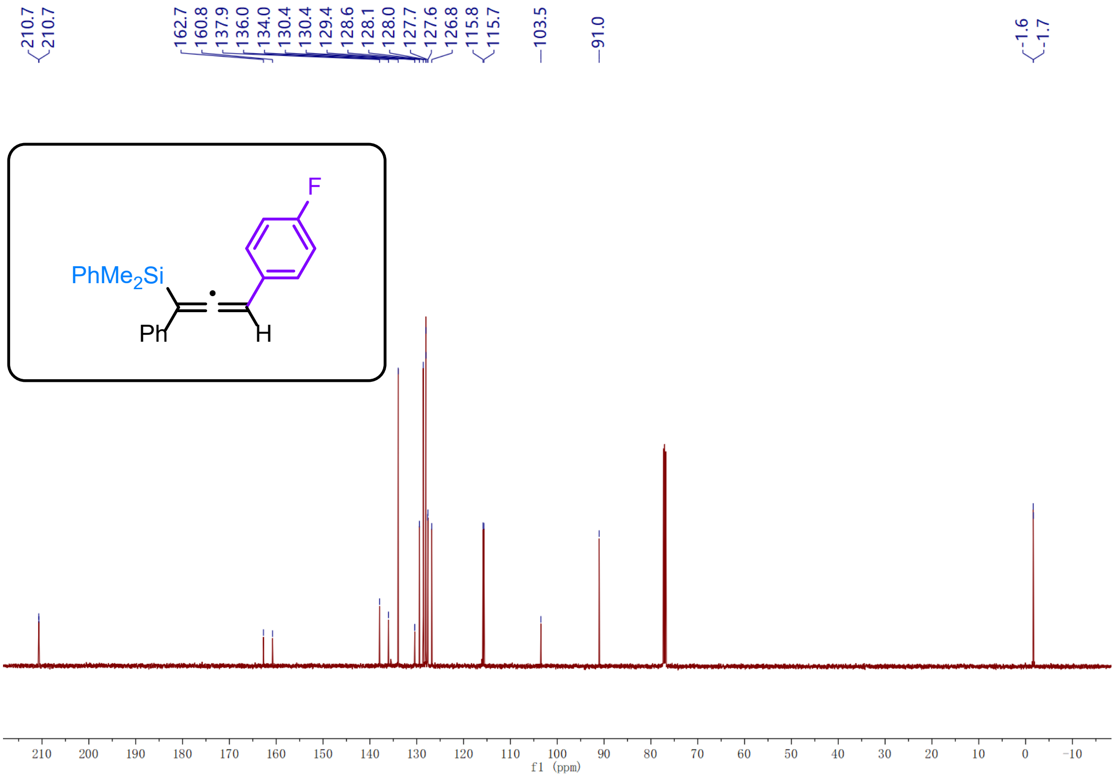

^{19}F NMR spectrum ($CDCl_3$) of **3ag**

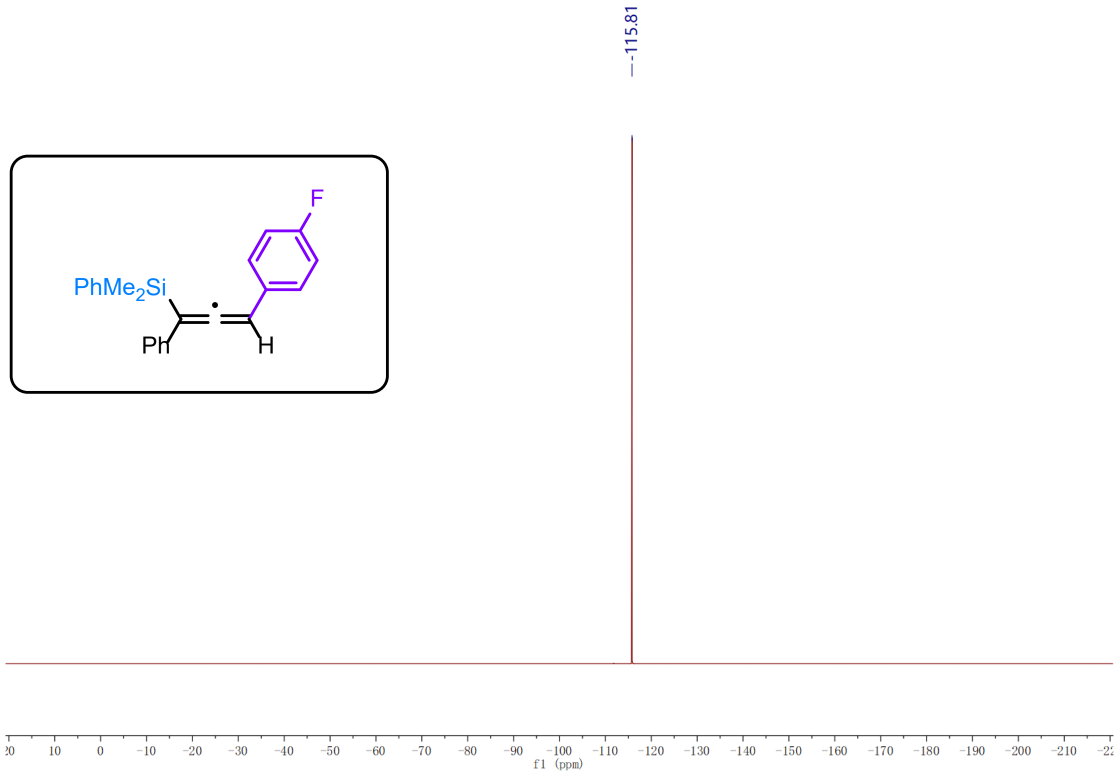

^{1}H NMR spectrum ($CDCl_3$) of **3ah**

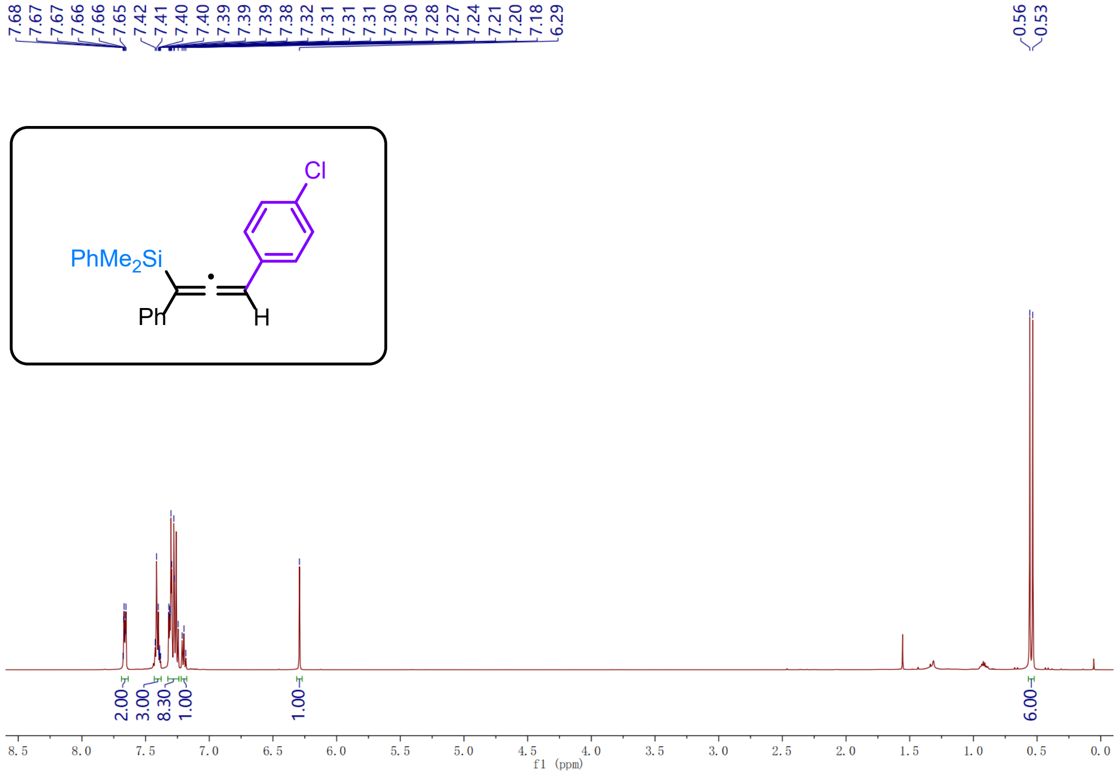

^{13}C NMR spectrum ($CDCl_3$) of **3ah**

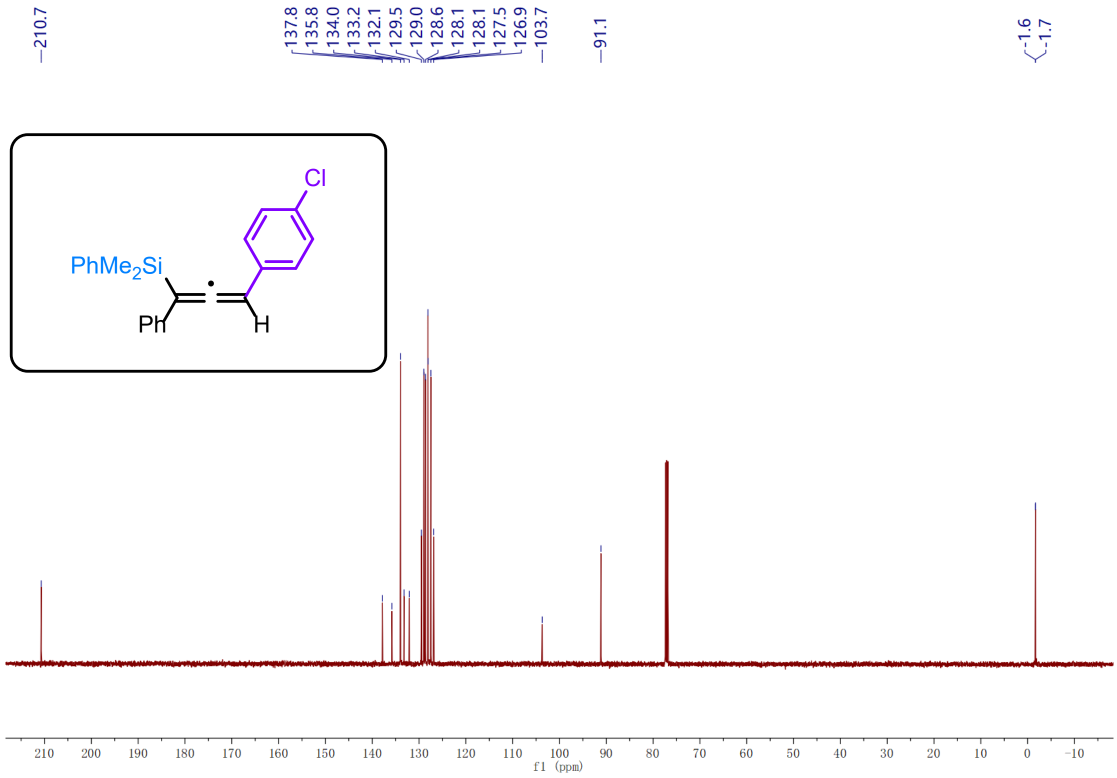

^{1}H NMR spectrum ($CDCl_3$) of **3ai**

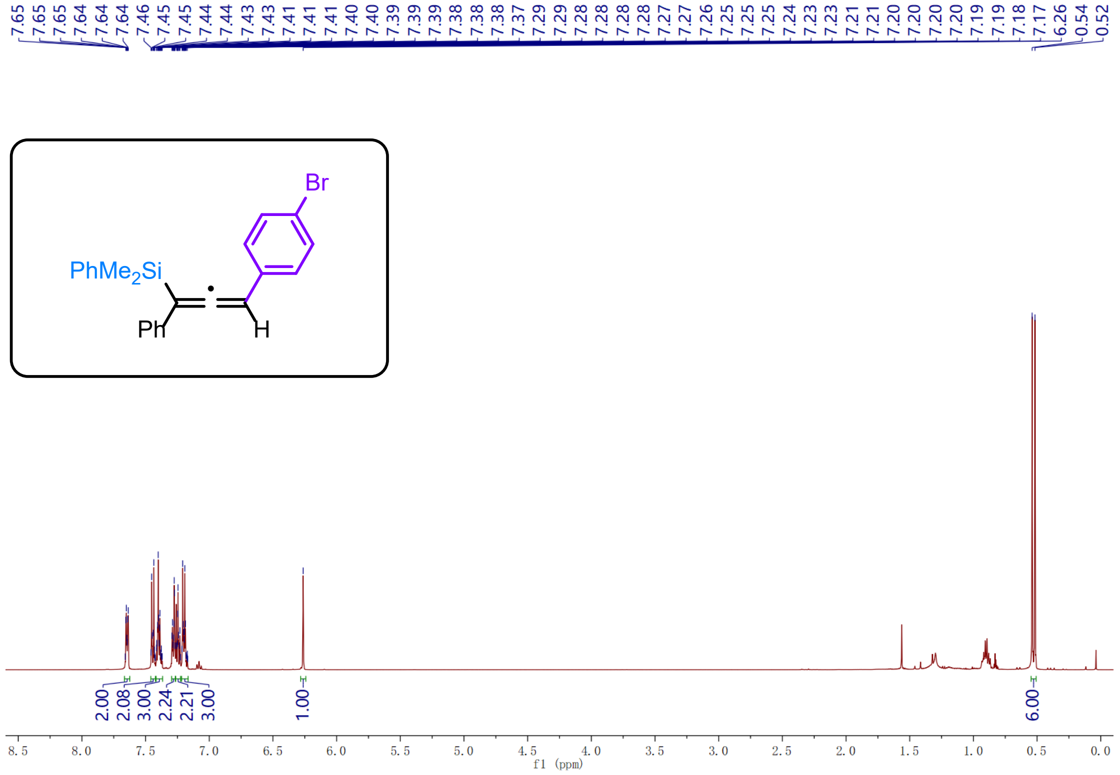

^{13}C NMR spectrum ($CDCl_3$) of **3ai**

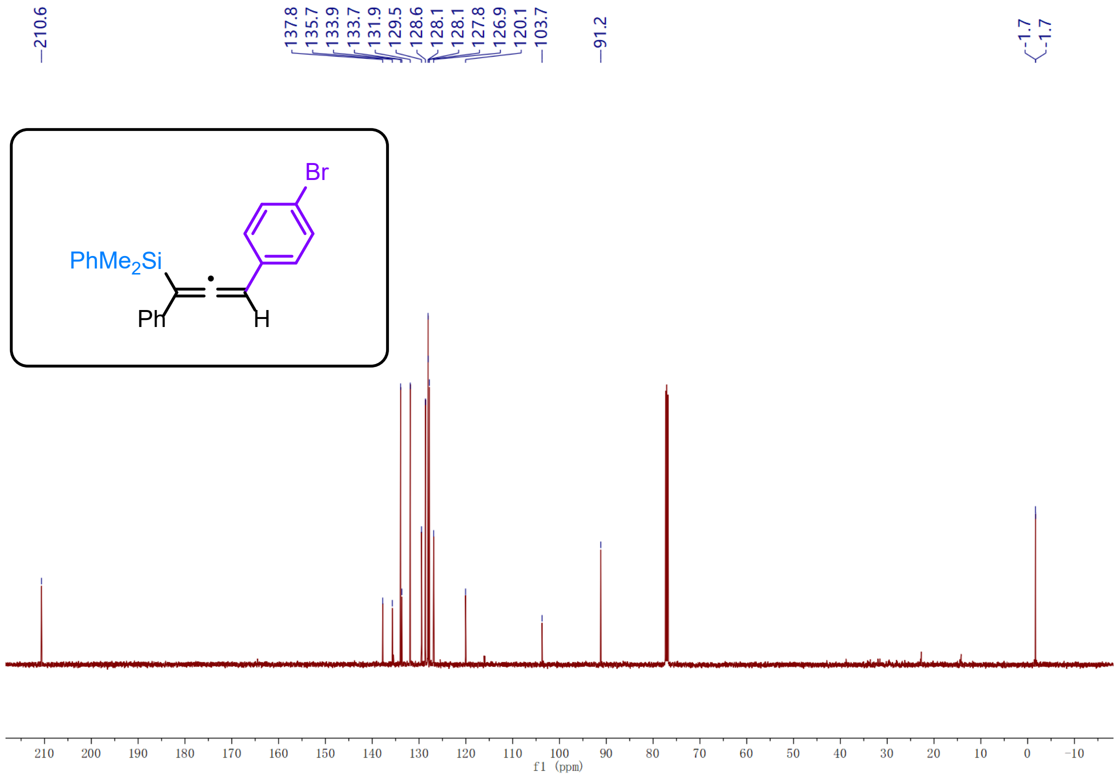

^{1}H NMR spectrum ($CDCl_3$) of **3aj**

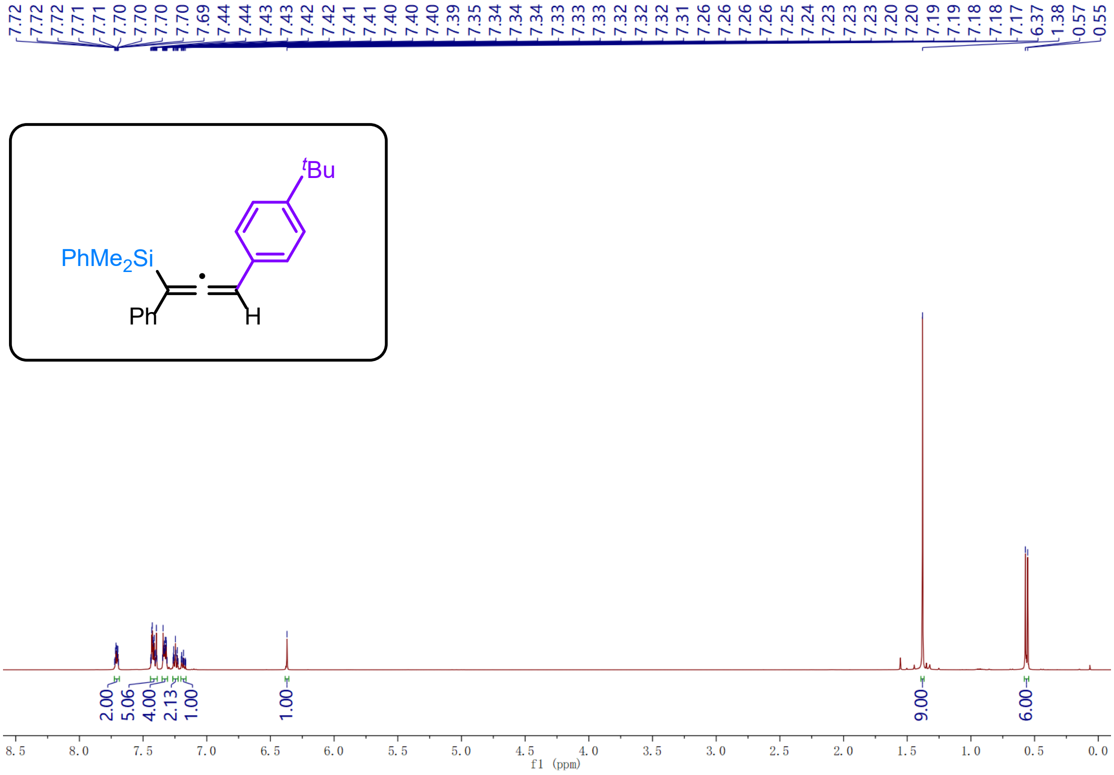

^{13}C NMR spectrum ($CDCl_3$) of **3aj**

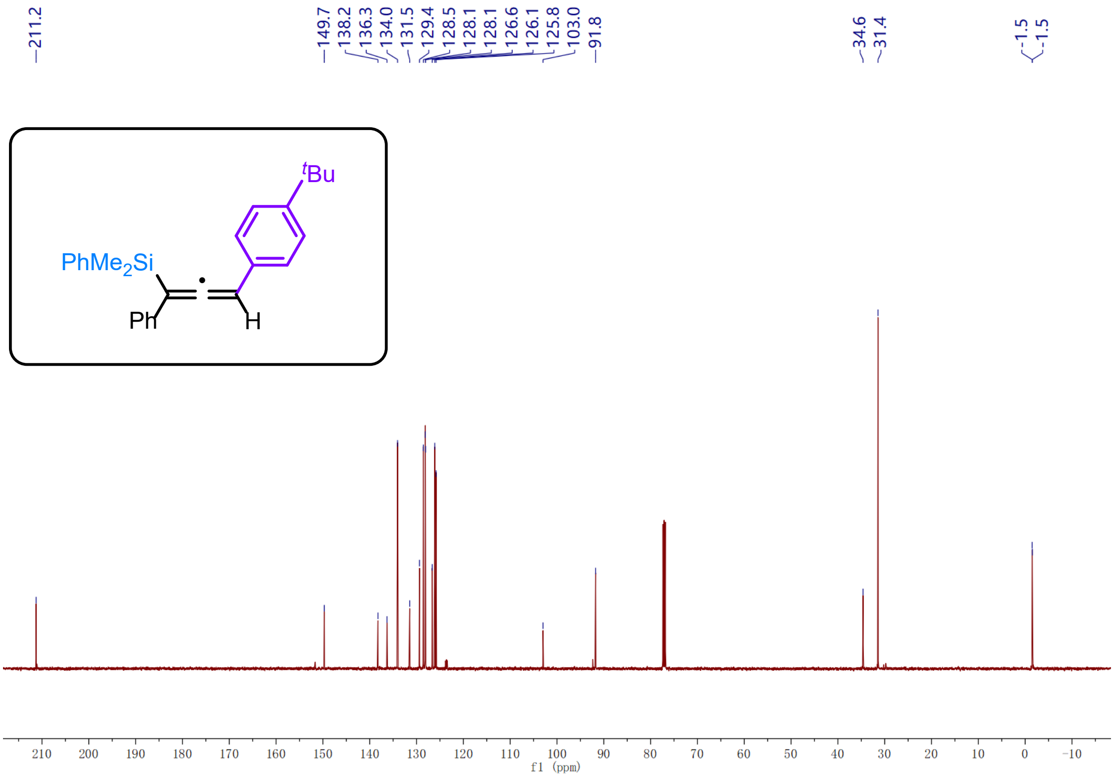

^{1}H NMR spectrum ($CDCl_3$) of **3ak**

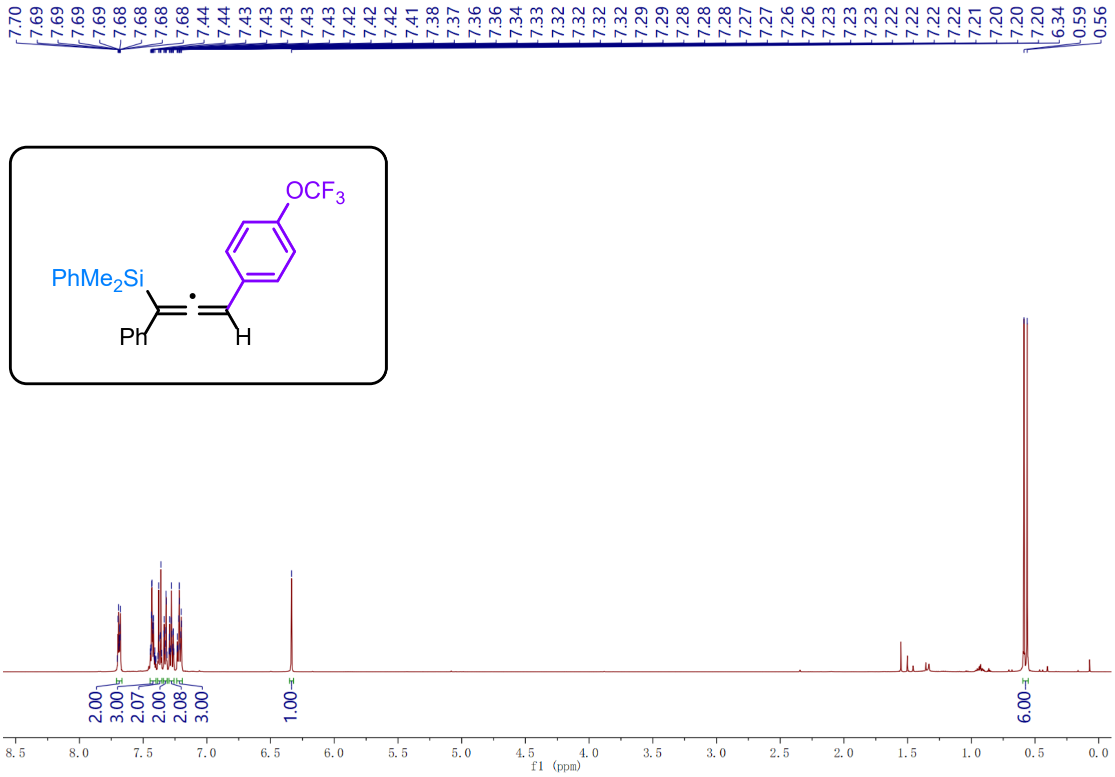

^{13}C NMR spectrum ($CDCl_3$) of **3ak**

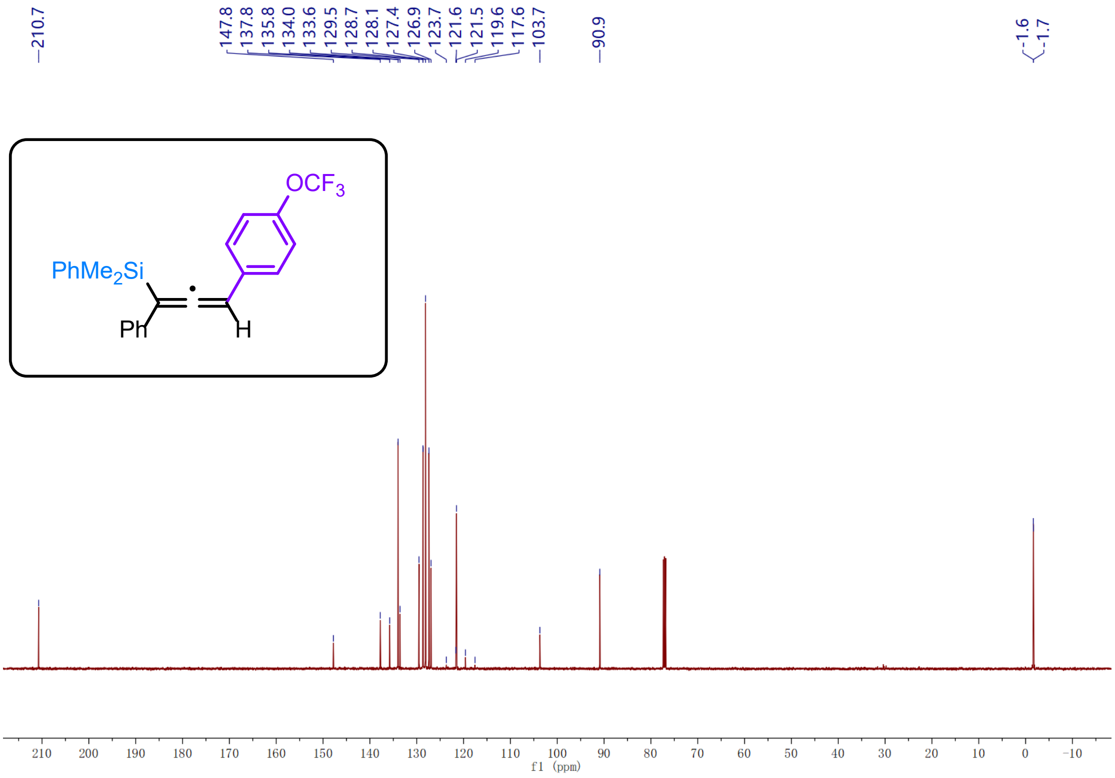

^{19}F NMR spectrum ($CDCl_3$) of **3ak**

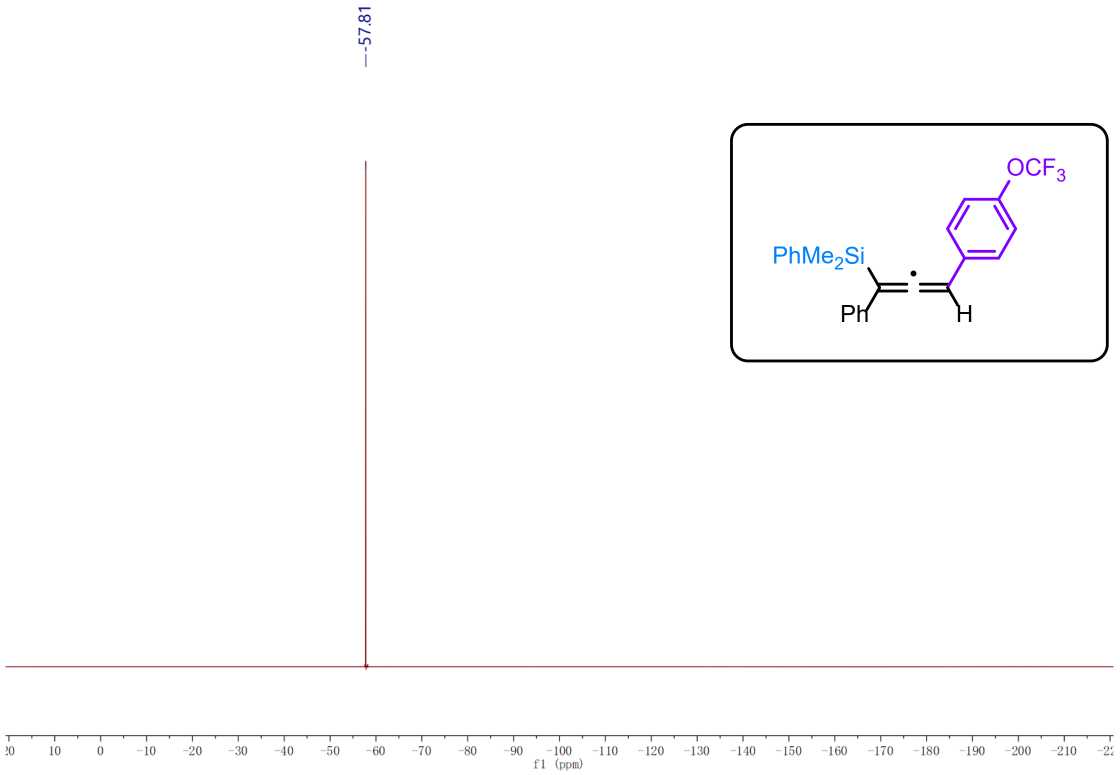

^{1}H NMR spectrum ($CDCl_3$) of **3al**

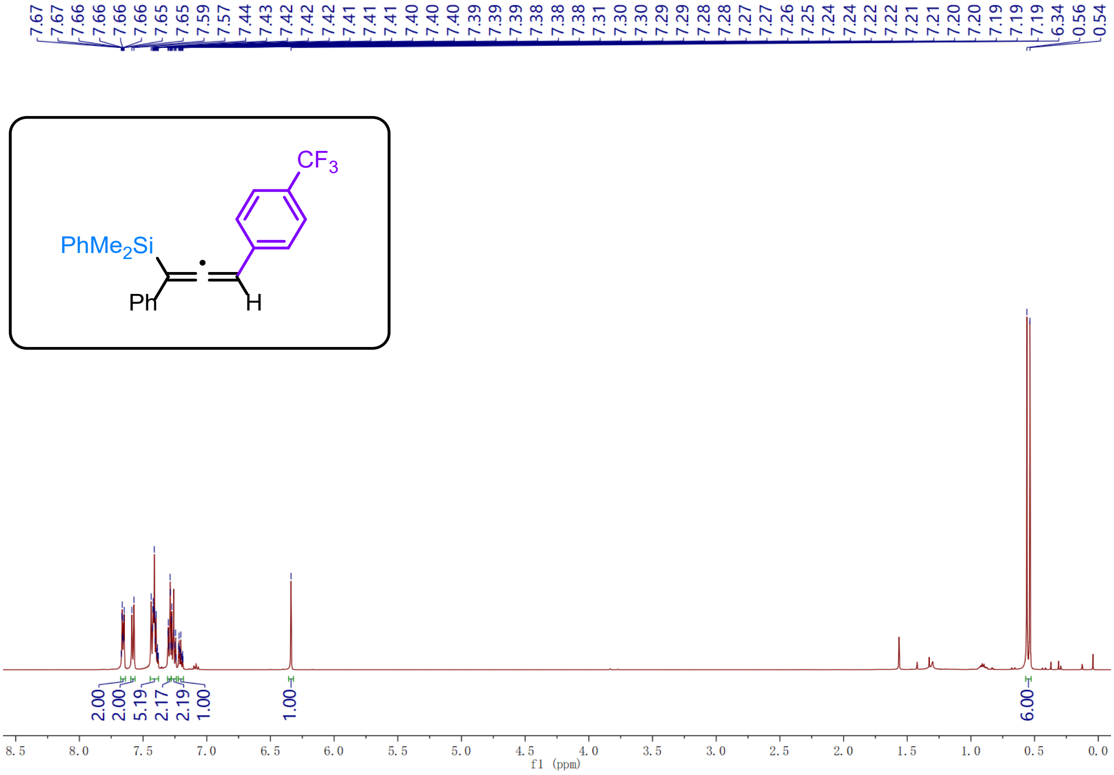

^{13}C NMR spectrum ($CDCl_3$) of **3al**

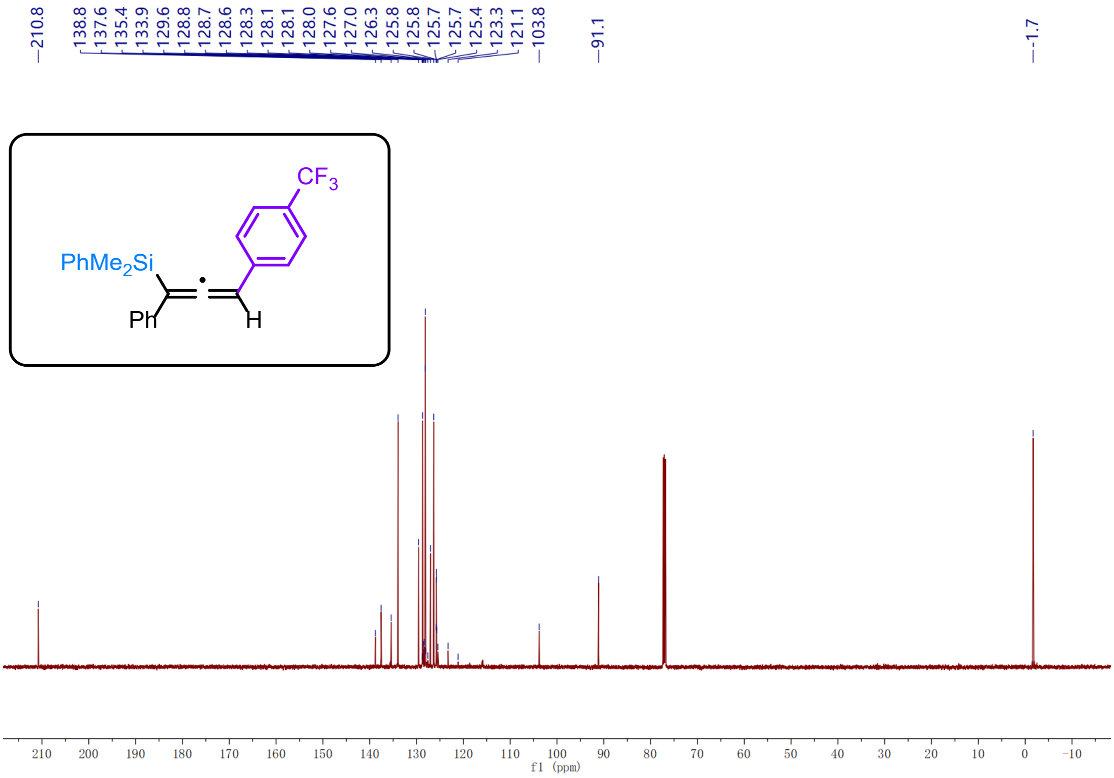

^{19}F NMR spectrum ($CDCl_3$) of **3al**

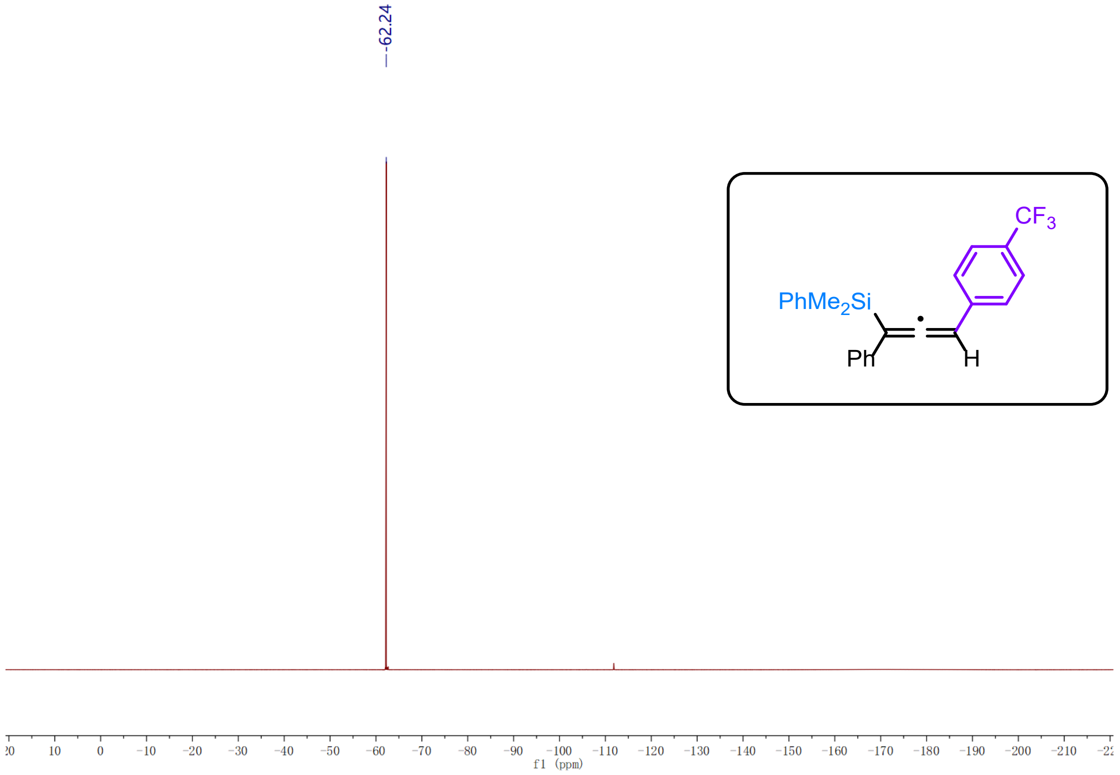

^{1}H NMR spectrum ($CDCl_3$) of **3am**

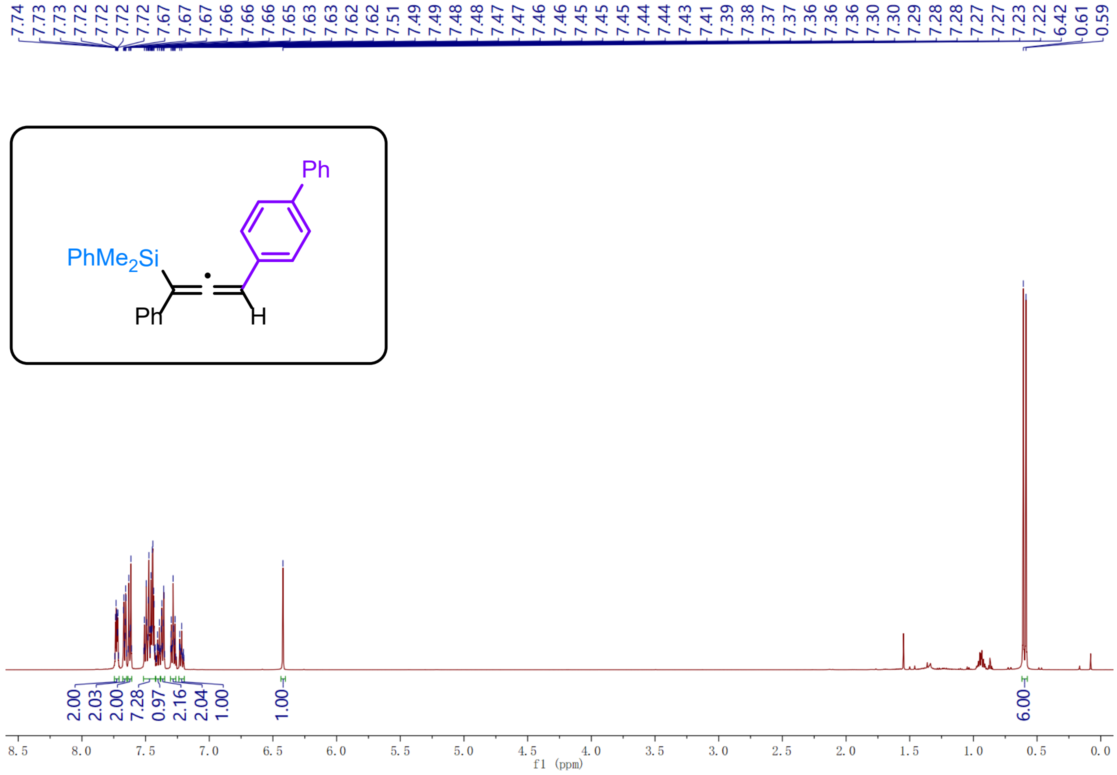

^{13}C NMR spectrum ($CDCl_3$) of **3am**

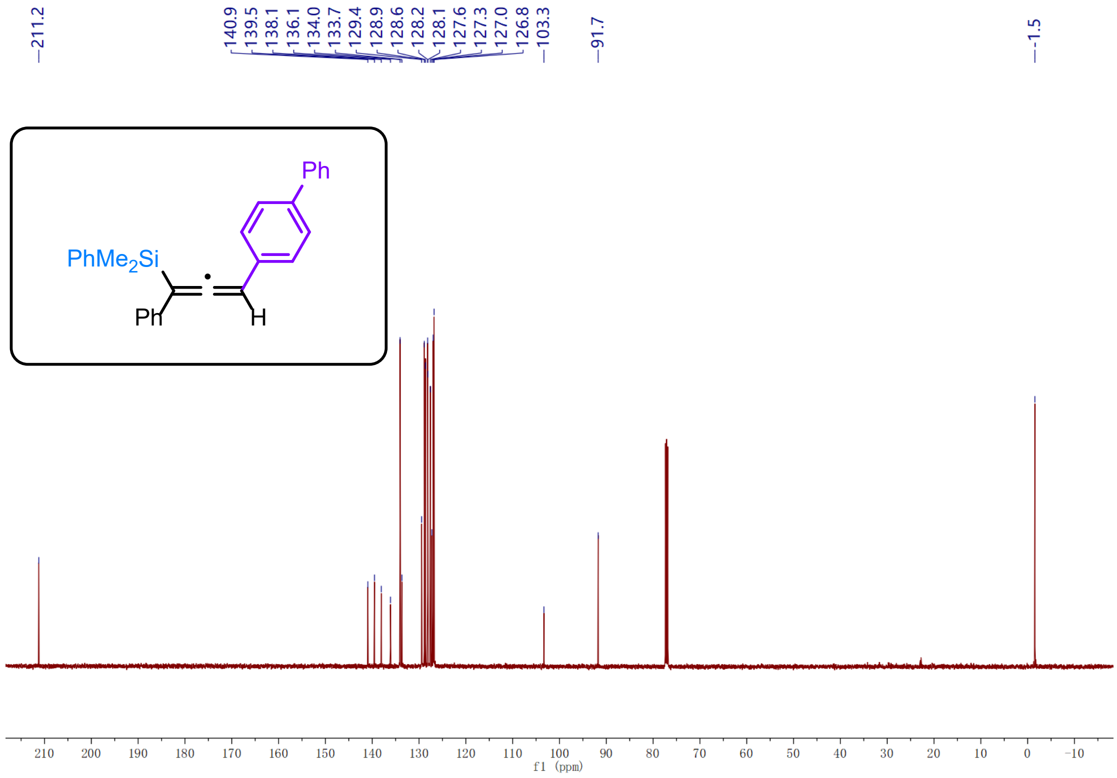

^{1}H NMR spectrum ($CDCl_3$) of **3an**

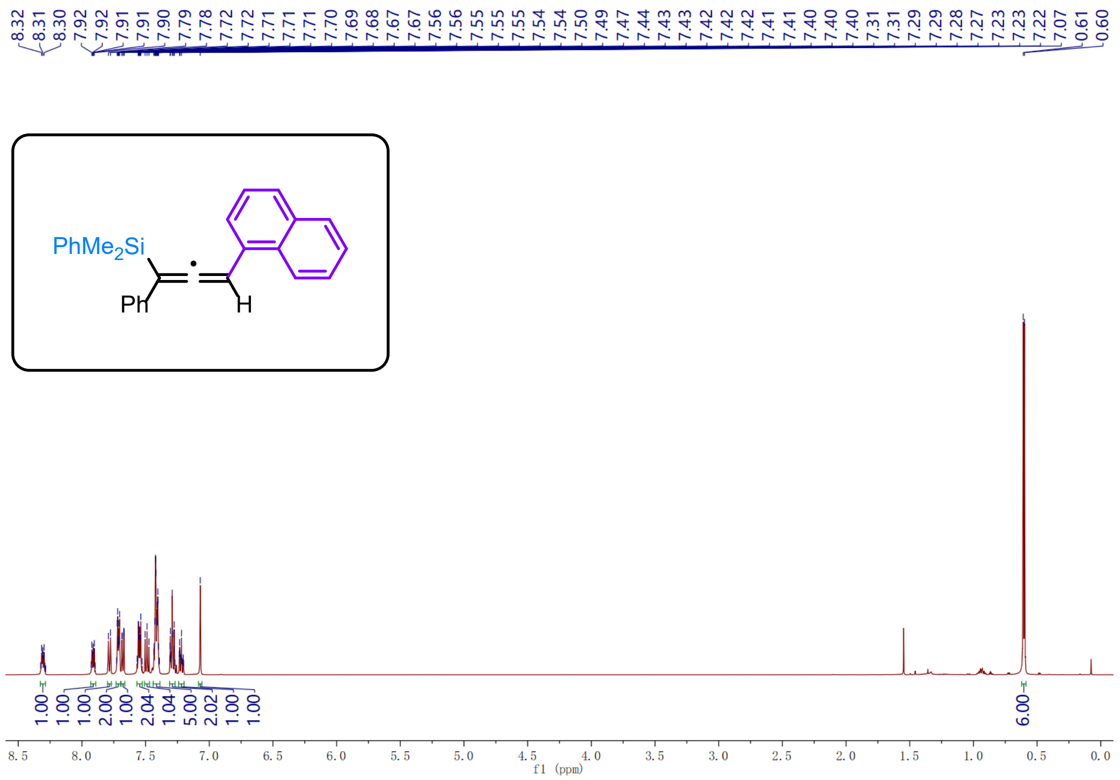

^{13}C NMR spectrum ($CDCl_3$) of **3an**

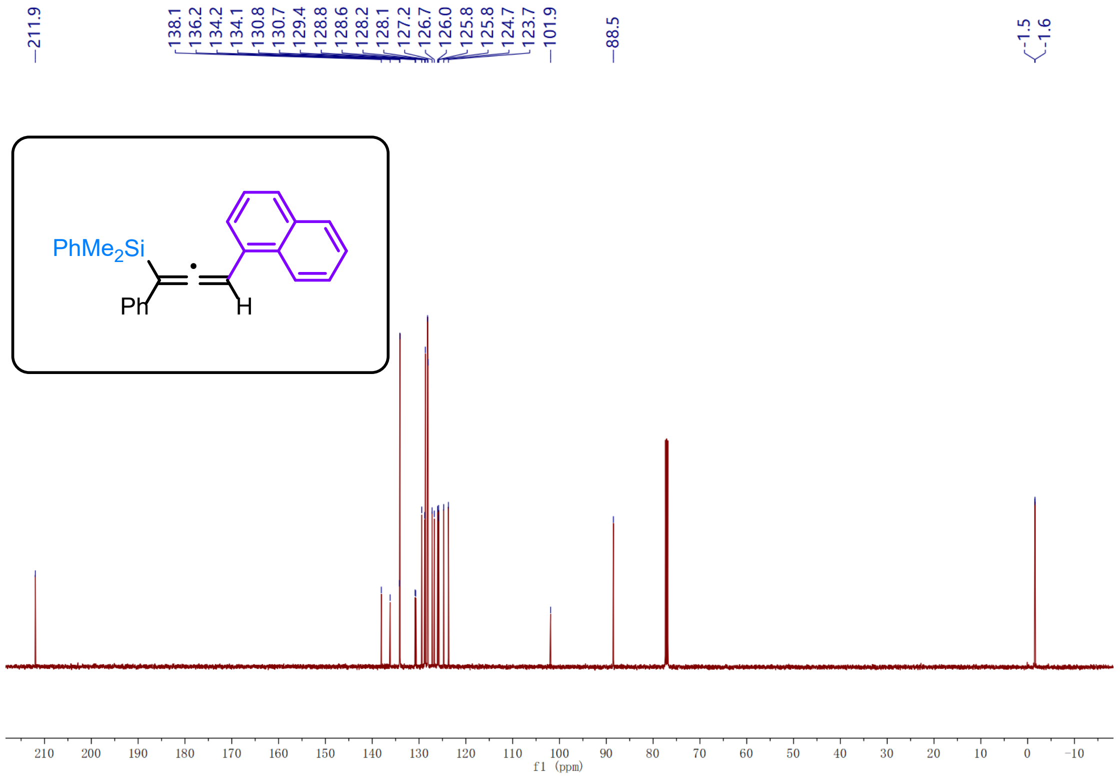

^{1}H NMR spectrum ($CDCl_3$) of **3ao**

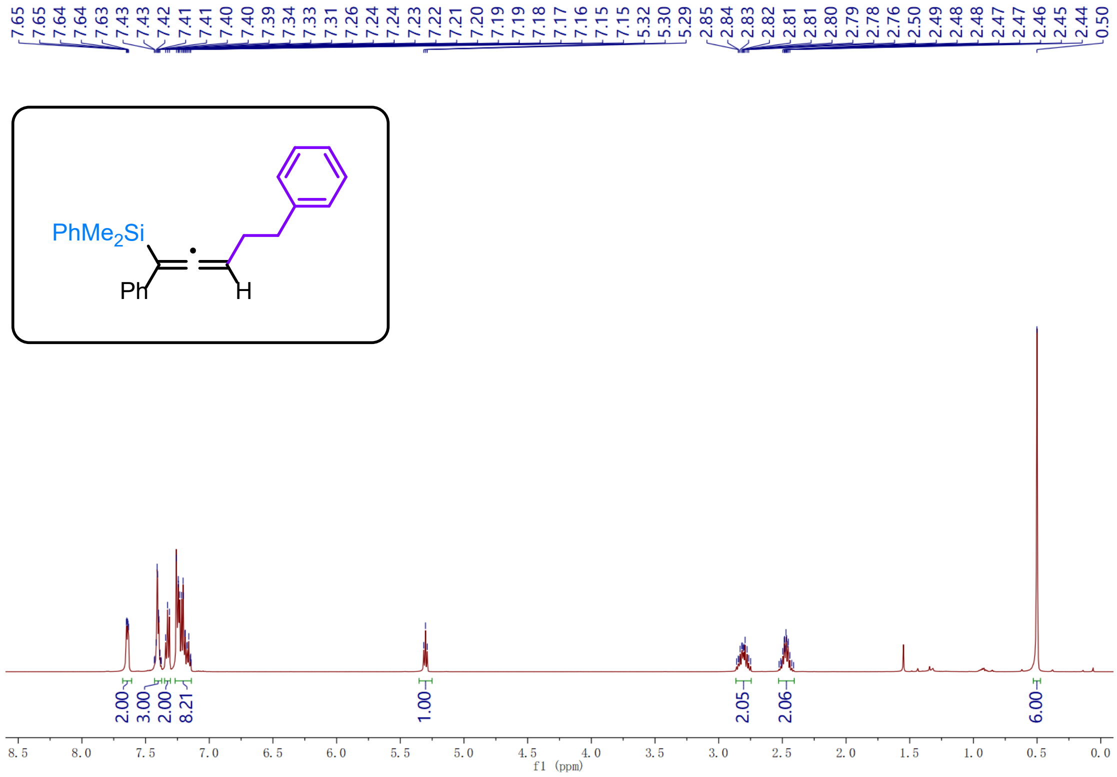

^{13}C NMR spectrum ($CDCl_3$) of **3ao**

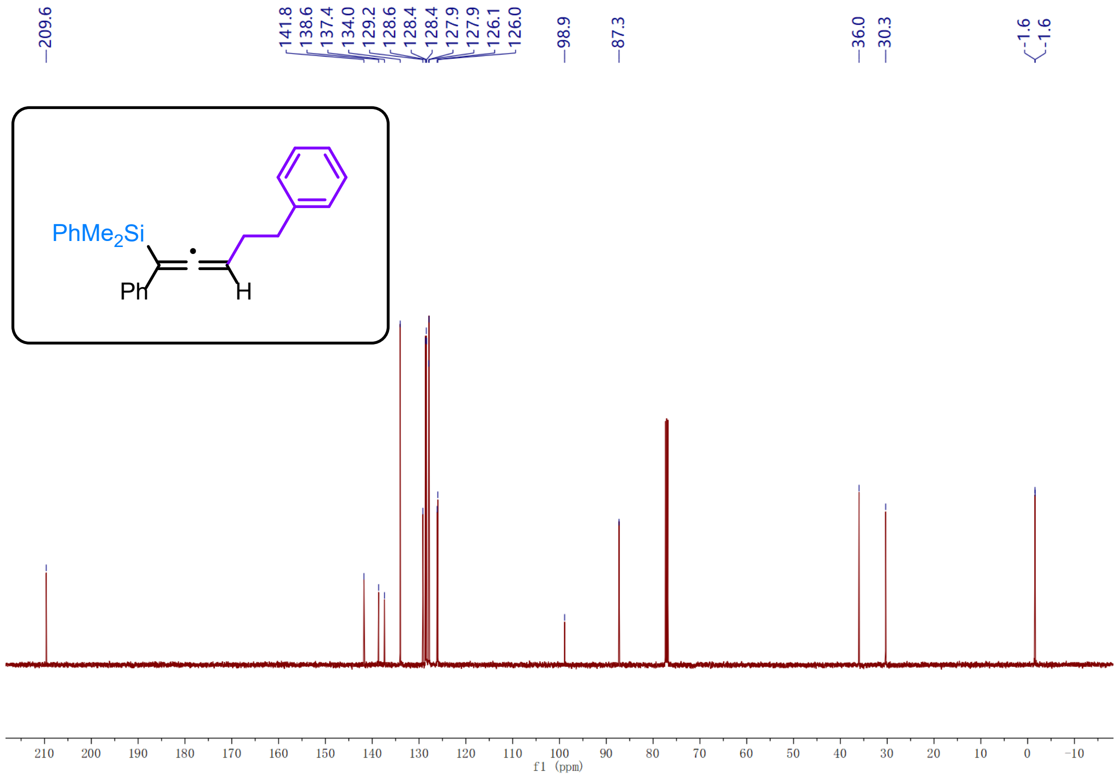

^{1}H NMR spectrum ($CDCl_3$) of **3ap**

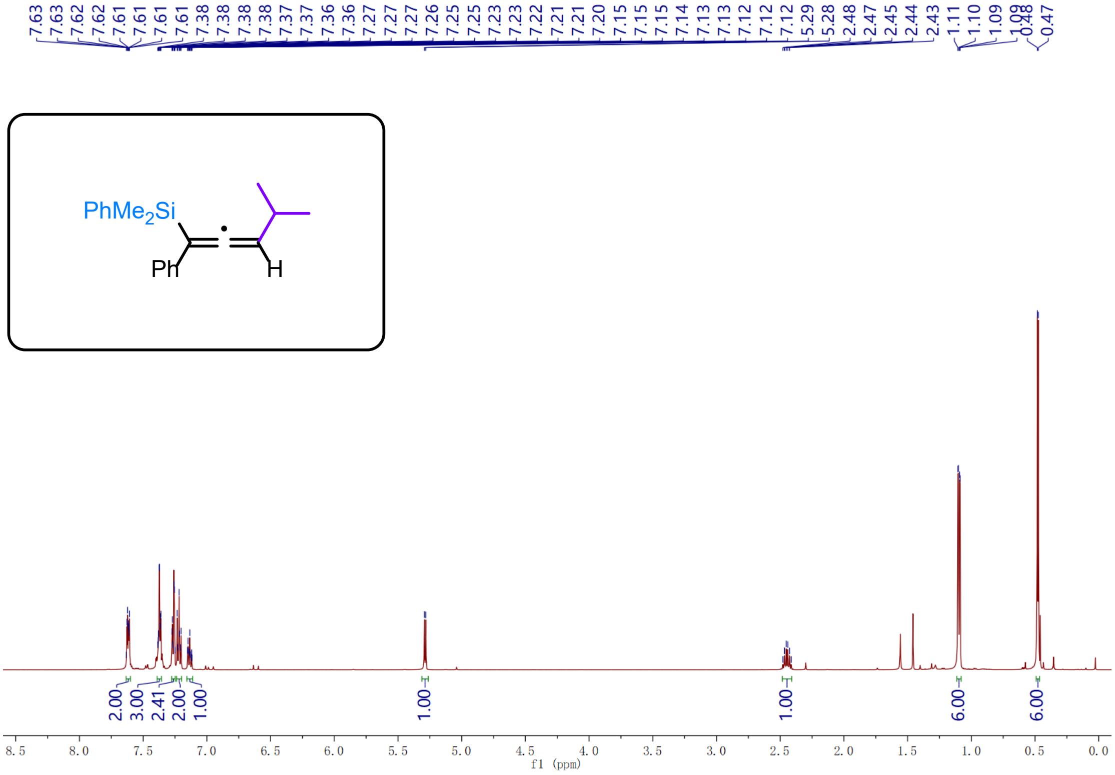

^{13}C NMR spectrum ($CDCl_3$) of **3ap**

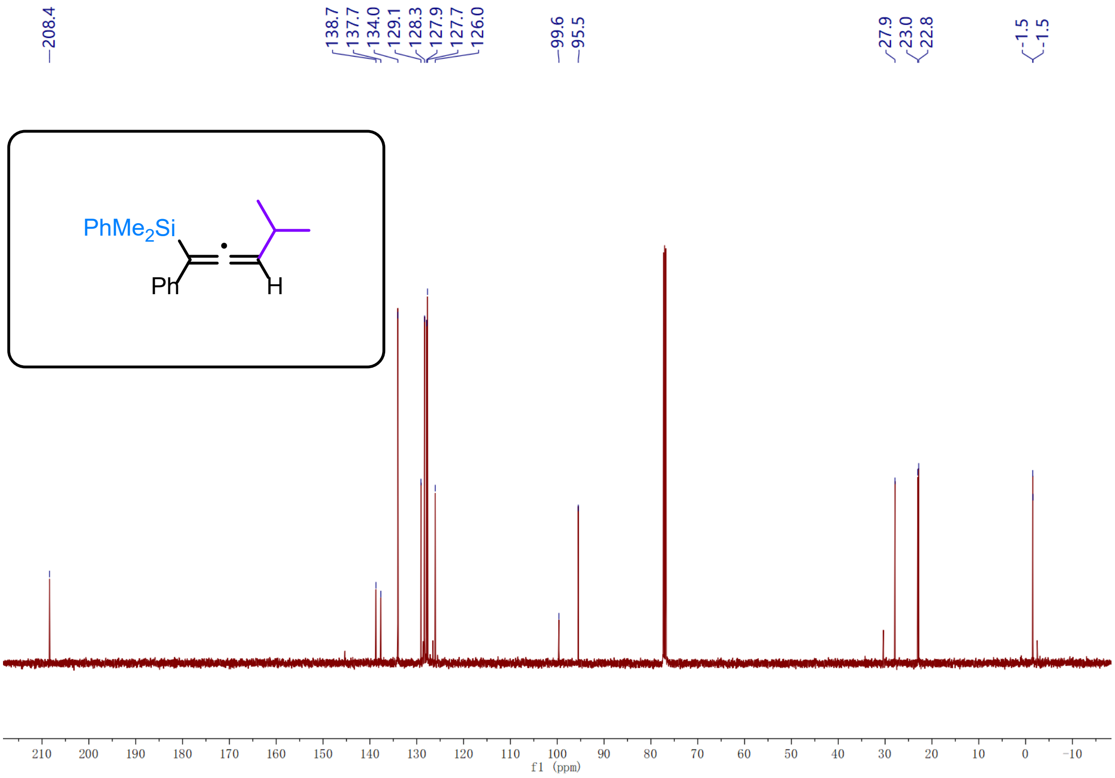

^{1}H NMR spectrum ($CDCl_3$) of **4**

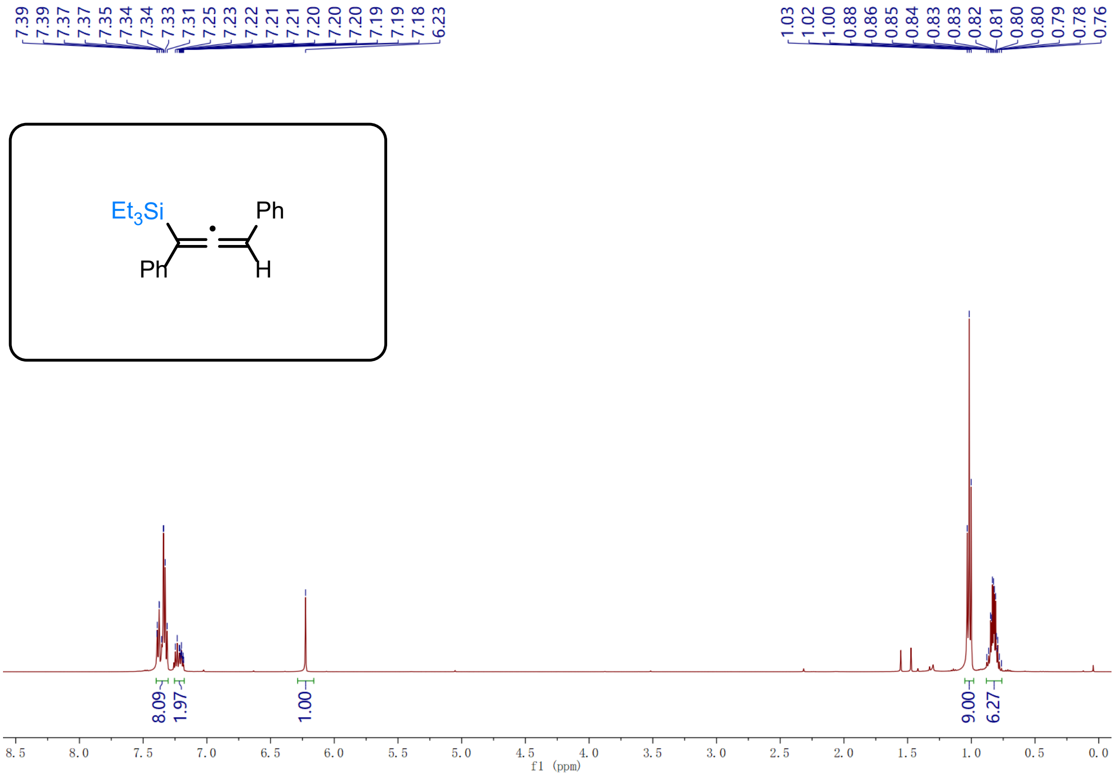

^{13}C NMR spectrum ($CDCl_3$) of **4**

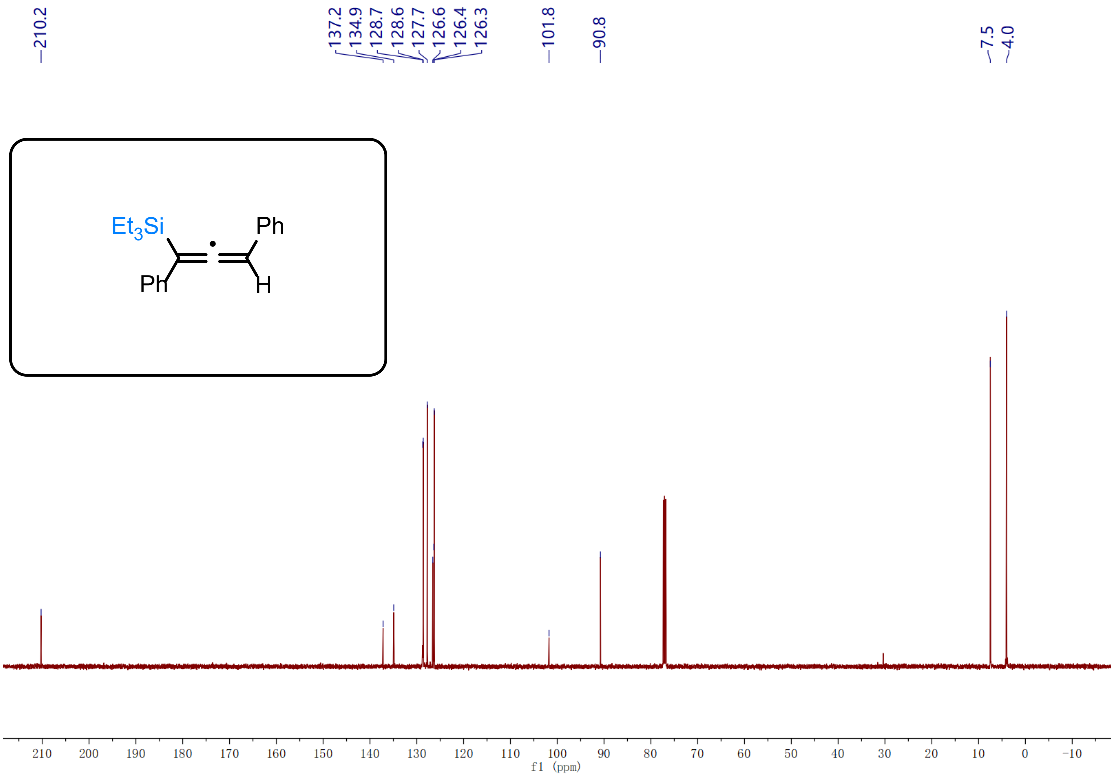

9. References

1. Zhou, Z.; Liu, G.; Chen, Y.; Lu, X., Cascade Synthesis of 3-Alkylidene Dihydrobenzofuran Derivatives via Rhodium(III)-Catalyzed Redox-Neutral C–H Functionalization/Cyclization. *Org. Lett.* **2015**, 17, 5874-5877.
2. Chang, X.; Zhang, J.; Peng, L.; Guo, C., Collective synthesis of acetylenic pharmaceuticals via enantioselective Nickel/Lewis acid-catalyzed propargylic alkylation. *Nat. Commun.* **2021**, 12, 299.
3. Li, R.; Ouyang, F.; Bai, Y.; Tang, R.; Yu, G.; Wei, B., Modular and Selective Access to Functionalized Alkynes and Allenes via the Intermediacy of Propargylic Acetates. *Org. Lett.* **2023**, 25, 2543-2547.
4. Okamoto, N.; Sueda, T.; Minami, H.; Miwa, Y.; Yanada, R., Regioselective Iodoazidation of Alkynes: Synthesis of α,α-Diazidoketones. *Org. Lett.* **2015**, 17, 1336-1339.
5. Su, Y.; Zhang, Y.; Akhmedov, N. G.; Petersen, J. L.; Shi, X., Ambient Intermolecular [2 + 2] Cycloaddition: An Example of Carbophilicity and Oxophilicity Competition in Au/Ag Catalysis. *Org. Lett.* **2014**, 16, 2478-2481.
6. Zhou, L.; Qiu, J.; Wang, C.; Zhang, F.; Yang, K.; Song, Q., Synthesis of α-Aminosilanes by 1,2-Metalate Rearrangement Deoxygenative Silylation of Aromatic Amides. *Org. Lett.* **2022**, 24, 3249-3253.
